# Supplementary material for: Unlocking full and fast conversion in photocatalytic carbon dioxide reduction for applications in radio-carbonylation
Source: Nat Commun. 2023 Jul 24;14:4451. doi: 10.1038/s41467-023-40136-w (PMC10366225; doi:10.1038/s41467-023-40136-w)
Supplement: Supplementary file 1 — Supplementary Information [file 41467_2023_40136_MOESM1_ESM.pdf]

Supplementary Information

Unlocking Full and Fast Conversion in Photocatalytic Carbon Dioxide Reduction for Applications in Radio-Carbonylation

Serena Monticelli,[1] Alex Talbot,[1] Philipp Gotico,[2] Fabien Caillé,[3] Olivier Loreau,[a] Antonio Del Vecchio,[1] Augustin Malandain,[1] Antoine Sallustrau,[1] Winfried Leibl,[2] Ally Aukauloo,[2,4] Frédéric Taran,[1] Zakaria Halime,[4,*] Davide Audisio[1,*]

1 Université Paris-Saclay, CEA, Service de Chimie Bio-organique et Marquage, DMTS, F-91191, Gif-sur-Yvette, France. E-mail: davide.audisio@cea.fr

2 Université Paris-Saclay, CEA, CNRS, Institute for Integrative Biology of the Cell, F-91191, Gif-sur-Yvette, France

3 Université Paris-Saclay, Inserm, CNRS, CEA, Laboratoire d’Imagerie Biomédicale Multimodale Paris-Saclay (BioMaps), F-91401 Orsay

4 Université Paris-Saclay, CNRS, Institut de chimie moléculaire et des matériaux d’Orsay, F-91400, Orsay, France. E-mail: zakaria.halime@universite-paris-saclay.fr

Table of contents

I. Supplementary Notes 4

Two-Chamber Glassware Characteristics 8

II. Supplementary Methods 12

General Procedure for [^{13}C]CO_2 Photoreduction Optimization in Two-Chamber Glassware Type 1 12

^{1}H-NMR yield determination of aminocarbonylated compounds [^{13}C]-1 using 1,3,5-trimethoxybenzene as Internal Standard (IS). 14

Pressure measurements of [^{13}C]CO_2 photoreduction 20

Full Experiment Pressure Profile 20

Recycling experiments 23

Proton Source Screening 25

Solvent and Light Intensity Screening 26

Electrochemical Characterization 28

Photo-irradiation Set-up 30

Product Analysis by Gas Chromatography 31

Kinetic Simulation 33

Preparation of starting materials 38

General Procedure 40

General Procedure for the [^{13}C]CO_2 photoreduction (GP0): 40

General Procedure for the 0.6 mmol [^{13}C]CO_2 photoreduction (GP0 bis): 42

General Procedure for the Aminocarbonylation (GP1):[15] 46

General Procedure for the Alkoxycarbonylation (GP2):[15] 46

General Procedure for the Carbonylative Suzuki Coupling (GP3):[16] 47

General Procedure for the Reductive Carbonylation (GP4a):[17] 47

General Procedure for the Reductive Carbonylation (GP4b):[17] 47

General Procedure for the Reductive Carbonylation (GP4c):[17] 48

General Procedure for the Carbonylative Sonogashira Coupling (GP5):[18] 48

General Procedure for the Carbon Isotope Exchange (GP6):[19] 49

Aminocarbonylation 50

Alkoxycarbonylation 56

Reductive Carbonylation 63

Carbonylative Suzuki Coupling 69

Carbon Isotope Exchange 75

Carbonylative Sonogashira Coupling 81

III. Supplementary Discussion 86

^{13}C Labeling of Pharmaceutical Relevant Compounds 86

Other Carbonylations Reactions with Aryl Chlorides and Alkyl Iodide 93

^{14}C Labeling of Pharmaceutically Relevant Compounds 96

^{11}C Radio-synthesis of amide **[^{11}C]1** 107

NMR Spectra 110

IV. Supplementary references 161

I. Supplementary Notes

***Reactants and solvents**:*

Unless otherwise noted, all reactions were carried out in oven-dried glassware.

Commercially available chemicals were purchased from ABCR, Acros Organics, Sigma-Aldrich, Alfa Aesar, Combi-Blocks, Carbolution, Fluorochem, and TCI Europe and used as received unless otherwise stated. The following solvents were dried by distillation over the drying agents indicated in parentheses: Tetrahydrofuran (Sodium), Dichloromethane (CaH_2). Additional anhydrous solvents were purchased from Acros Organics, Sigma Aldrich, Alfa Aesar and stored over molecular sieves under an argon atmosphere. Carbon-13 labeled CO_2 was purchased from Cisotec Inc./ Matheson ([^{13}C]CO_2 : 99.2 atom % ^{13}C, 7.9 atom % ^{18}O), and charged on TRITEC manifold system prior to utilization (RC TRITEC LTD. Teufen, Switzerland).

***Purifications**:*

Flash chromatography (FC) was performed on silica gel (Merck Kieselgel 60, grading 40-63 μm) or using automated Combiflash® Rf Teledyne ISCO with pre-packed column RediSep® Rf (grading 35-70 μm).

Analysis:

Reactions were monitored by TLC carried out on silica 0.25 mm (60 F254, Merck) using UV light as a visualizing agent. For staining, the TLC plates were dipped into a solution of basic aqueous permanganate (1 g $KMnO_4$, 6 g K_2CO_3 and 0.1 g KOH in 100 mL H_2O) and developed with a heat gun. Nuclear Magnetic Resonance (NMR) Spectroscopy: ^{1}H NMR (400 MHz), ^{13}C NMR (100 MHz) were measured on a Brucker Avance 400 MHz spectrometer. Chemical shifts are reported in parts per million (ppm) downfield from residual solvents peaks and coupling constants are reported as Hertz (Hz). Splitting patterns are designated as singlet (s), broad singlet (br. s), doublet (d), triplet (t), quartet (q), quintet (quint), multiplet (m). Splitting patterns that could not be interpreted or easily visualized are designated as multiplet (m).

Electrospray mass spectra were obtained using an ESI-Quadripole autopurify, Waters (pump: 2545, mass: ZQ2000) mass Spectrometer.

LC-MS spectra were recorded on a Waters Acquity UPLC® equipped PDA eλ Detector and SQ Detector 2, mobile phase A: H_2O + 0.1% formic acid, mobile phase B: acetonitrile + 0.1% formic acid.

High-resolution mass spectra (HRMS) were performed on a Bruker maXis mass spectrometer by the "Fédération de Recherche" ICOA/CBM (FR2708) platform (University of Orléans).

Infrared spectra (IR) were obtained on a Perkin Elmer UATR TWO FTIR spectrophotometer and are reported as wavenumbers (cm^{-1}).

Melting points (Mp) were obtained on a BÜCHI Melting Point B-545 and are reported in °C.

For Carbon-14 radiolabeling:

Carbon-14 labeled reagents and compounds were handled by personnel uniquely trained in working with radioactive materials and operating in specialized CEA laboratories.

Carbon-14 radioactivity was measured in Perkin Elmer Ultra Gold liquid scintillation cocktail with a PerkinElmer 3110TR liquid scintillation analyzer.

Radio-HPLC and HPLC-UV analysis was conducted with a Waters Alliance 2695 connected to an MS detector Waters ZQ 2000 and a Scintillation Analyzer Berthold 514. Alternatively, on a Waters Acquity UPLC® equipped PDA eλ Detector and SQ Detector 2, mobile phase A: H_2O + 0.1% formic acid, mobile phase B: acetonitrile + 0.1% formic acid and a Scintillation Analyzer Berthold 509.

Mass spectra (ESI) for the calculation of specific activities were performed on a Waters Micromass ZQ spectrometer,

- For compound ([^{14}C]1): column HSSC18 (1.8 µm, 2.5 x 50 mm) and a linear gradient of 5% B to 60% B over 10 min (mobile phase A = water with 0.1% formic acid; mobile phase B = acetonitrile with 0.1% formic acid; flow rate = 0.4 $mL.min^{-1}$; desolvation gas = nitrogen; capillary temperature = 40 °C).
- For compound ([^{14}C]40): column C18 (3.5 µm, 4.6 x 100 mm) and a linear gradient of 5% B to 100% B over 8 min (mobile phase A = water with 0.1% formic acid; mobile phase B = acetonitrile with 0.1% formic acid; flow rate = 1 $mL.min^{-1}$; desolvation gas = nitrogen; capillary temperature = 25 °C).

- For compound ([^{14}C]37): column C18 (3.5 µm, 4.6 x 100 mm) and a linear gradient of 5% B to 100% B over 24 min (mobile phase A = water with 0.1% formic acid; mobile phase B = acetonitrile with 0.1% formic acid; flow rate = 1 mL.min^{-1}; desolvation gas = nitrogen; capillary temperature = 25 °C).

For compound ([^{14}C]42): column C18 (3.5 µm, 4.6 x 100 mm) and a linear gradient of 5% B to 70% B over 24 min (mobile phase A = water with 0.1% formic acid; mobile phase B = acetonitrile with 0.1% formic acid; flow rate = 1 mL.min^{-1}; desolvation gas = nitrogen; capillary temperature = 25 °C).

[^{14}C]CO_2 (2.172 GBq mmol^{-1}) was generated using a [^{14}C]CO_2 manifold system (RC Tritec AG). Mass spectra (ESI) for the calculation of specific activities were obtained using a Waters Micromass ZQ spectrometer. Radiochemical purities were determined by Thin Layer Chromatography on TLC silica gel 60F254 glass plates (Merck) using a RITA scanner (Raytest) for the radioactive detection.

Additional Equipment:

- Kessil A160WE Tuna Blue, LED lamp® (40W) was used as source of blue light.
- Keller Leo record digital manometer® was adapted on specific double chamber COware SyTRACK H-Cap® for performing pressure measurement.

Safety instructions:

CAUTION: Carbon monoxide (CO) is a highly toxic gas

All the reactions in which carbon monoxide is used/formed must be performed in a well-ventilated fume-hood. To prevent uncontrolled release of gas and the possible exposure of the operator, for all experiments, operators were equipped with a *CO detector(s):*

Type: Dräger PAC® 6000 or 6500, personal CO gas detector

Safety Goggles. As a precaution measure, all photochemical reactions were performed under a well-ventilated fume hood, with light screen protection measures. In addition, operators were equipped with Light Eye Protection Goggles (LASER 2000 GmbH), when in proximity of a blue light source.

Two-Chamber Glassware Characteristics

All two-chamber glassware reactors were entirely glassblower-made to fit the RC-Tritec carboxylation manifold or adapted from commercial material (https://www.sytracks.com/). The specifications of the glassware are provided below.

The system was sealed using silicone/PTFE seals (Figure S4). in combination with the stabilizing discs each seal allows for a maximum of 2 piercings using 21G/Ø0.80 mm syringes or smaller.

Glassware under pressure - Warning!

As shown by the pressure experiments (see below), the reactions were not performed under gas pressure. Nonetheless, as a precaution, glass equipment should always be examined for damages to its surface, which may weaken its strength.

All experiments were performed within all laboratory safety procedures and always behind a shield and/or closed ventilated fume hood.

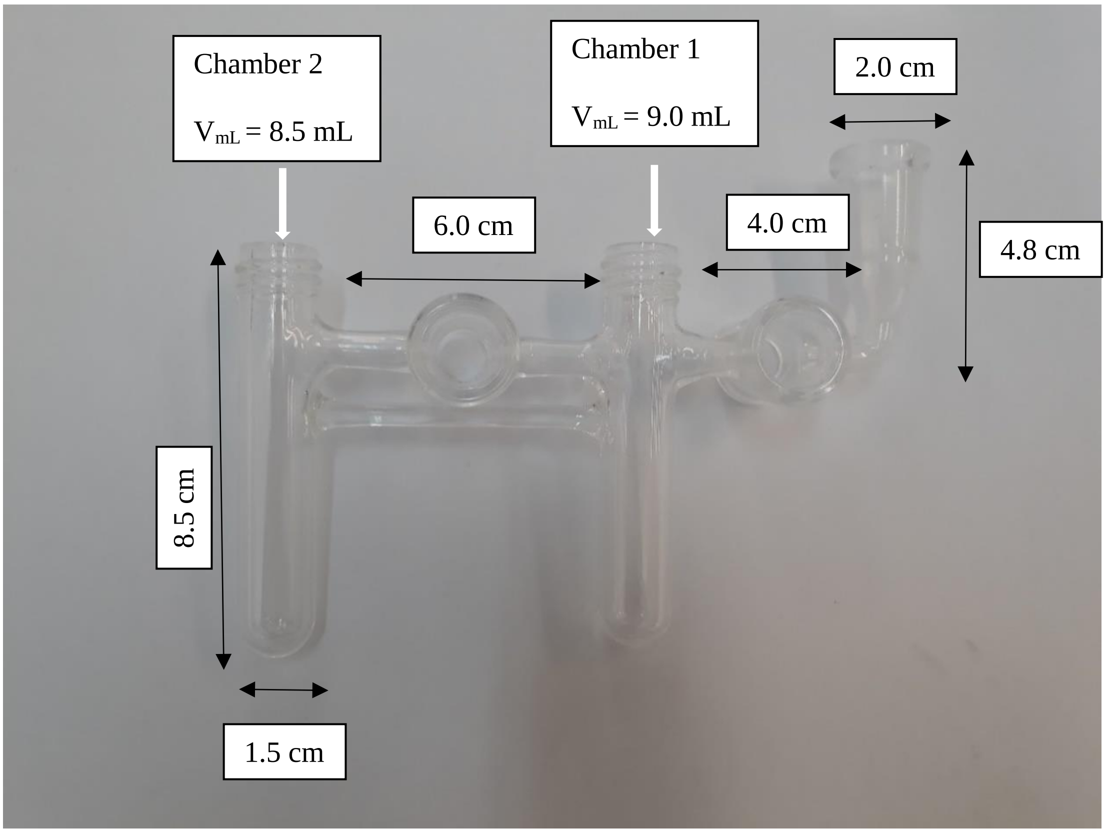

Supplementary Figure 1 Glassware Type 1

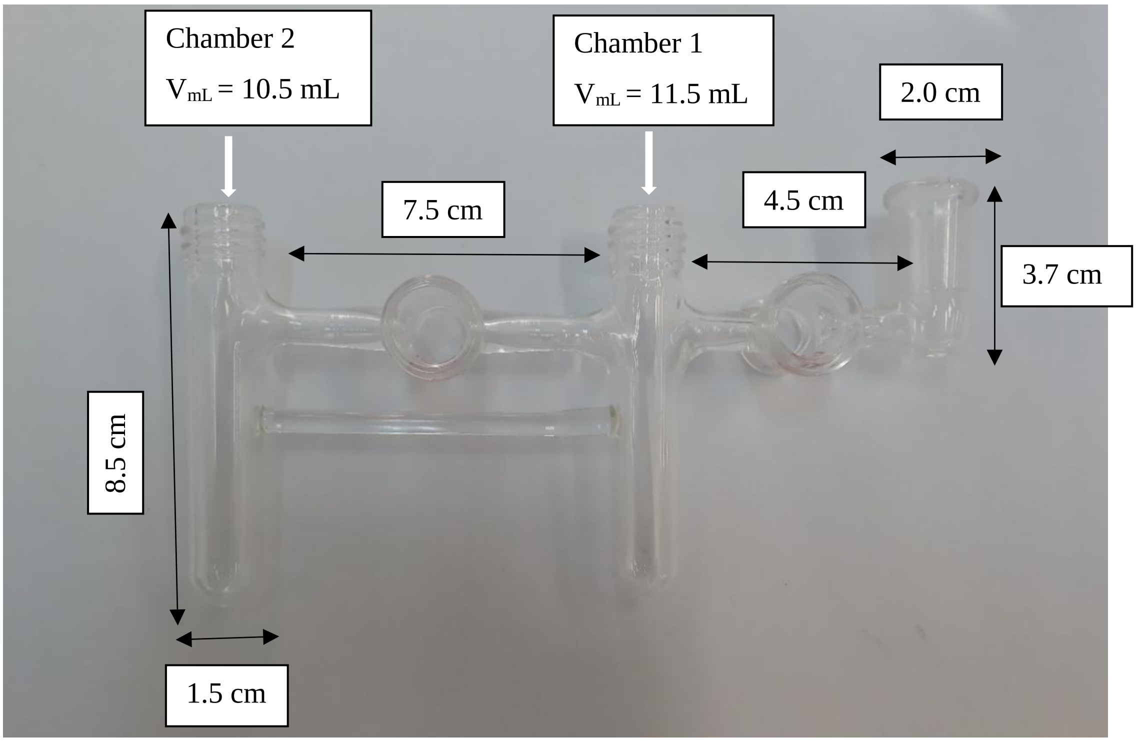

Supplementary Figure 2 Glassware Type 2

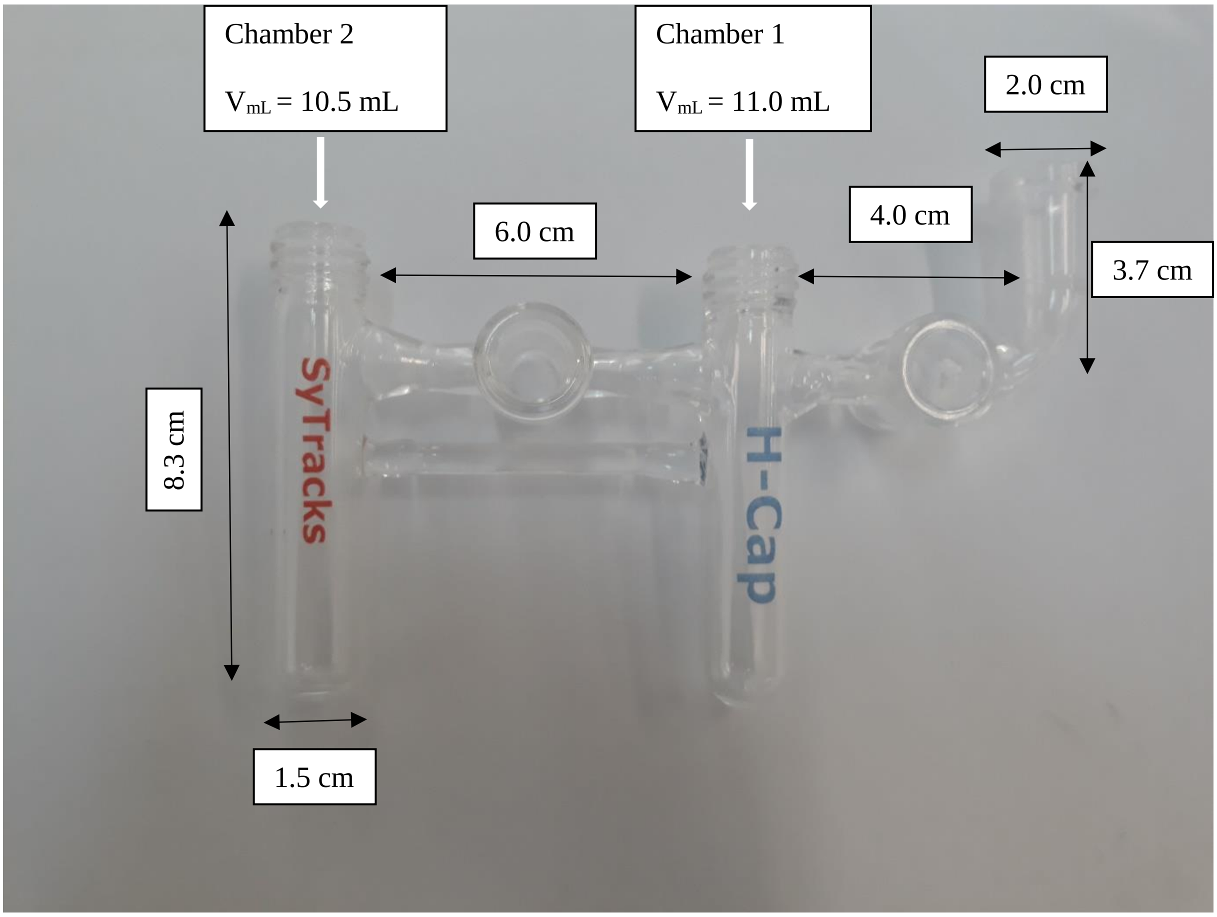

Supplementary Figure 3 Glassware Type 3 – Pressure studies

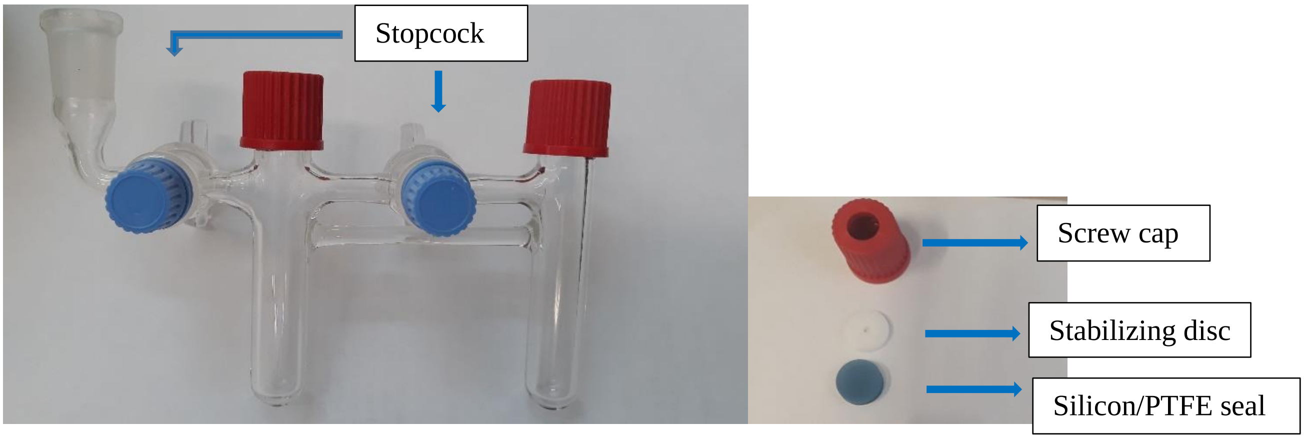

Supplementary Figure 4 Two-Chamber Glassware set-up

See : https://www.rctritec.com/en/tritium-handling-technology/c-14-manifold-system.html

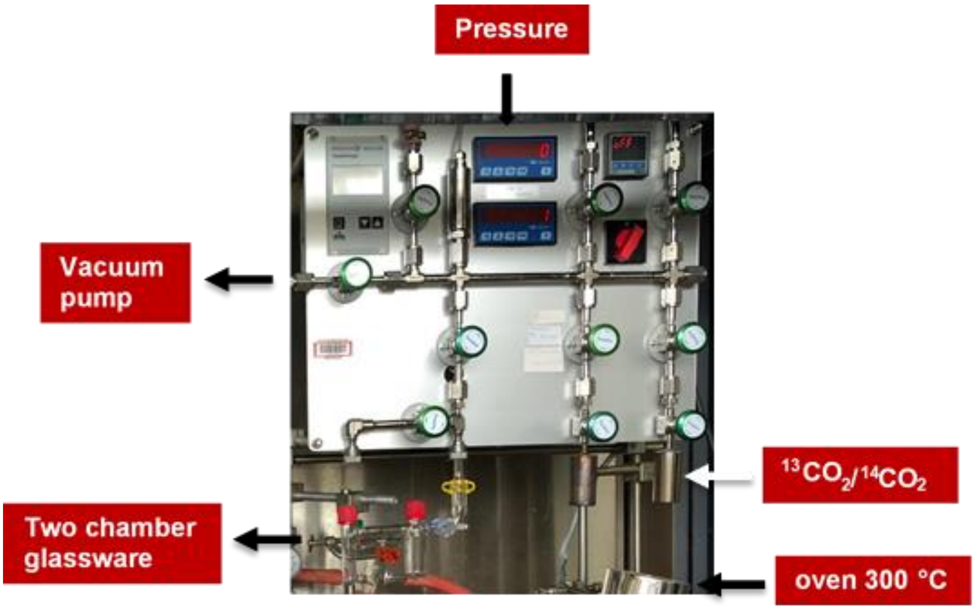

Supplementary Figure 5 RC Tritec® manifold system for [^{13}C]CO_2 and [^{14}C]CO_2 delivery

II. Supplementary Methods

General Procedure for [^{13}C]CO_2 Photoreduction Optimization in Two-Chamber Glassware Type 1

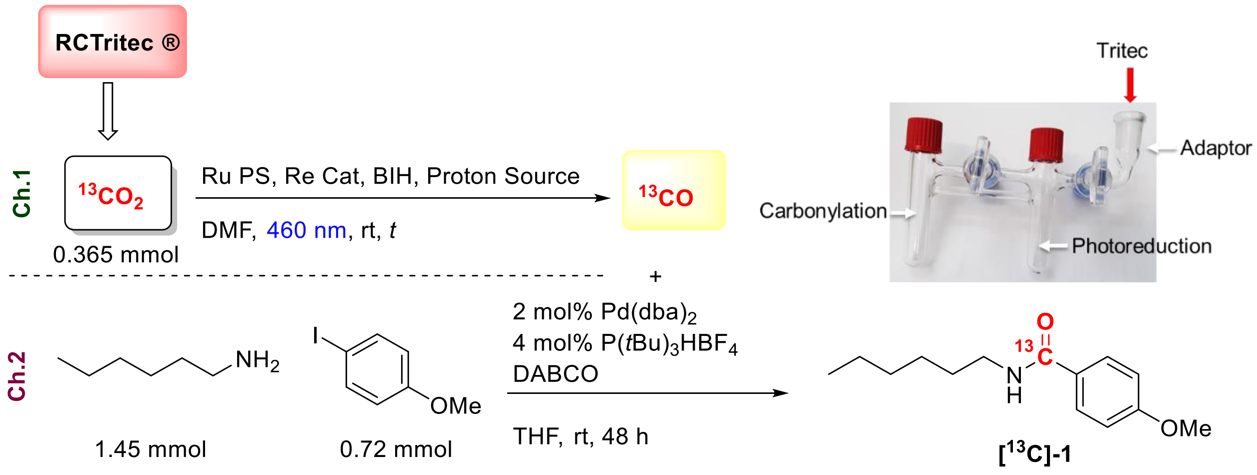

Supplementary Figure 5a Two-chamber reaction schemes

Chamber 1:

A solution of Ru(bpy)$_3$Cl$_2$.6H$_2$O stock solution, Re(CO)$_3$(bpy)Br stock solution, DMF, BIH (194 mg, 0.78 mmol), phosphine, proton source (H_2O, TEOA, phenol, TFE) were transferred into Chamber 1 with a Pasteur pipette glass. The chambers were sealed with a screwcap fitted with a Teflon® (Figure 4). The adaptor was then connected to the RC Tritec® system. The solution in Chamber 1 was frozen with a liquid nitrogen bath and the chambers were degassed with a *vacuum* pump connected with an RC Tritec manifold for 10 min. The stopcock was closed between the two chambers. **[^{13}C]CO_2** (365 μmol) was then loaded into Chamber 1 using the RC Tritec® system and the stopcock was closed between Chamber 1 and the adaptor. The loaded Chamber 1 was then disconnected from the RC Tritec® system and the solution was warmed to room temperature. Chamber 1 was placed *ca.* 2 cm away from a 40 W A160WE Tuna Blue Kessil® LED lamp and photo-irradiated with the lower light intensity (164 W m^{-2}) for 1 h.

Note: A stock solution of 1.34 mM Ru(bpy)$_3$Cl$_2$.6H$_2$O was prepared by dissolving 1 mg in 1 mL dimethylformamide (DMF). A stock solution of 1.98 mM Re(CO)$_3$(bpy)Br was prepared by dissolving 1 mg in 1 mL DMF.

Chamber 2:

A freshly prepared solution of $Pd(dba)_2$ (8.7 mg, 0.015 mmol), $P(\textit{t}Bu)_3HBF_4$ (8.7 mg, 0.030 mmol), 4-iodoanisole (169 mg, 0.724 mmol), *n*-hexylamine (0.19 mL, 1.45 mmol), and DABCO (162 mg, 1.45 mmol) in dry THF (3.0 mL) under argon is injected *via* syringe in Chamber 2 through the screwcap septum. The stopcock between the two chambers is open and the $[^{13}C]CO$ produced in Chamber 1 can react in the aminocarbonylation in Chamber 2. The Two-Chamber Glassware was then held at room temperature for 48 hours under constant stirring. After this time, the glassware is degassed through the *vacuum* pipe and the crude mixture is transferred from Chamber 2 in a round bottom flask. The solvent evaporated using a rotary evaporator and quantification of the carbonylated product was performed by ^1H-NMR (or by isolated yield where specified). $[^{13}C]CO_2$ is the limiting reagent.

^{1}H-NMR yield determination of aminocarbonylated compounds [^{13}C]-1 using 1,3,5-trimethoxybenzene as Internal Standard (IS).

The ratio between analyte and internal standard is:

$$rA/IS = (2.24/2)/(1/3) = 3.39$$

The sample contains 15 mg internal standard (1,3,5-trimethoxybenzene):

$$nIS = 15 \text{ mg}/168.19 \text{ mg/mmol} = 0.089 \text{ mmol}$$

Combining these two equations gives:

$$nA = 0.089 \times 3.39 = 0.302 \text{ mmol}$$

The sample in the crude reaction mixture in which the theoretical yield of analyte is expected to be 0.365 mmol, therefore the ^{1}H-NMR yield is:

$$\% = (0.302 \text{ mmol} / 0.365 \text{ mmol}) \times 100 = 83\%$$

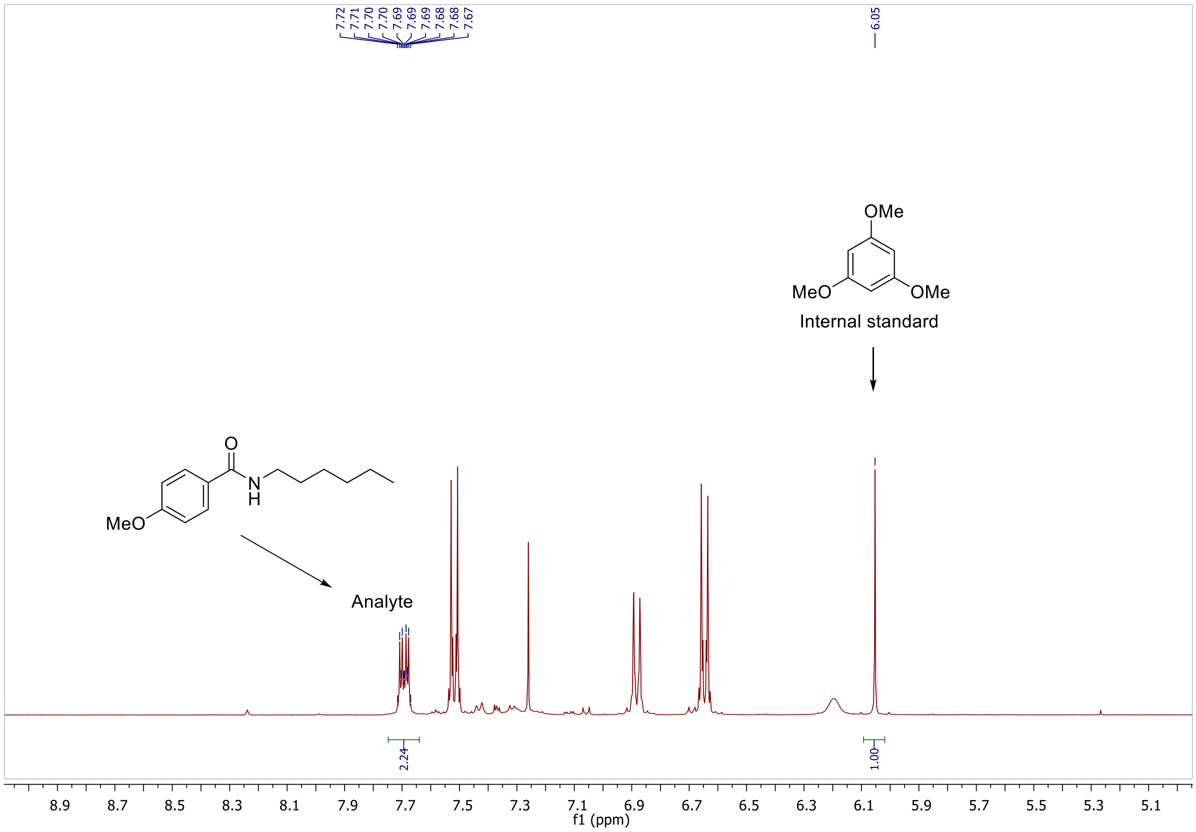

Supplementary Figure 5b *Internal standard yield determination*

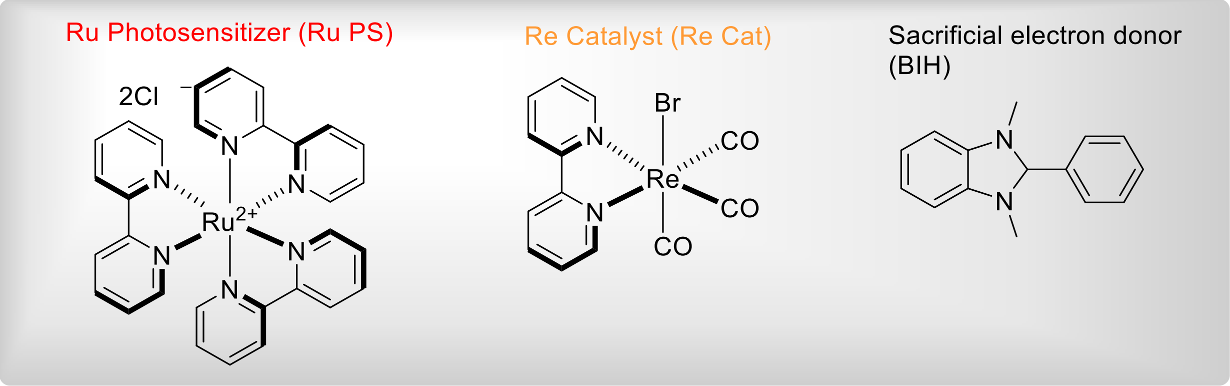

Supplementary Figure 6 Structures of Ru PS, Re Cat and BIH.

Entry	Ru PS µmol	Re Cat µmol	BIH mmol	Proton Source	PPh_3 mmol	Time (h)	^{1}H-NMR [^{13}C]1 Yield % [f]
**1**	0.54	0.54	0.78	H_2O[a]	-	21	35
**2**	0.54	0.54	0.78	TEOA[b]	-	21	32
**3**	0.54	0.54	0.78	TEOA[b]	0.36	21	37
**4**	1.62	1.62	0.78	TEOA[b]	0.36	21	42
**5**	1.62	1.62	0.78	TEOA[b]	0.36	1	44
**6**	0.54	0.54	0.78	phenol[b]	0.36	21	45
**7**	1.08	1.08	0.78	phenol[b]	0.36	21	69
**8**	1.08	1.08	0.78	phenol[b]	-	21	61
**9**	-	1.08	0.78	phenol[b]	0.36	21	75
**10**	1.08	1.08	0.78	-	0.36	21	6
**11**	1.08	-	0.78	phenol[b]	0.36	21	0
**12**	1.08	1.08	-	phenol[b]	0.36	21	0
**13**	1.62	1.62	0.78	phenol[b]	0.36	21	75
**14**	1.62	1.62	0.78	phenol[b]	0.36	15	72
**15**	1.62	1.62	0.78	phenol[b]	0.36	5	73
**16**	1.62	1.62	0.78	phenol[b]	0.36	1	70 (*isolated*)
**17**	1.62	1.62	0.78	phenol[b]	0.36	30 min	77
**18**	1.62	1.62	0.78	phenol[b]	0.36	5 min	14
**19**	-	1.62	0.78	phenol[b]	0.36	21	74 (*isolated*)

Entry	Ru PS µmol	Re Cat µmol	BIH mmol	Proton Source	PPh_3 mmol	Time (h)	^{1}H-NMR [^{13}C]1 Yield % ^{f}
**20**	-	1.62	0.78	phenol^{b}	-	21	64
**21**	-	2.16	0.78	phenol^{b}	0.36	21	58
**22**	2.16	2.16	0.78	phenol^{b}	0.36	21	44
**23**	1.62	1.62	0.78	phenol^{c}	0.36	1	85
**24**	1.62	1.62	0.78	phenol^{d}	0.36	1	57
**25**	1.62	1.62	0.78	phenol^{e}	0.36	1	36
**26**	1.62	1.62	0.78	phenol^{c}	0.18	1	80
**27**	**1.62**	**1.62**	**0.78**	**phenol**^{c}	**0.036**	**1**	**83**
**28**	-	1.62	0.78	phenol^{c}	0.036	1	40
**29**	1.62	1.62	-	phenol^{c}	0.036	1	0
**30**	1.62	-	0.78	phenol^{c}	0.036	1	0
**31**	1.62	1.62	0.78	-	0.036	1	10
**32**	1.62	1.62	0.78	phenol^{c}	-	1	45
**33**	**2.7**	**2.7**	0.78	phenol^{c}	-	1	55
**34**^{f}	1.62	1.62	0.78	phenol^{c}	0.036	1	0
**35**	1.62^{g}	1.62	0.78	phenol^{c}	0.036	1	40
**36**	1.62^{g,h}	-	0.78	phenol^{c}	0.036	1	3
**37**	1.62^{g}	-	0.78	phenol^{c}	0.036	1	3

Supplementary Table 1 [^{13}C]CO_2 Photoreduction Optimization. All experiments are performed in DMF. ^{a} 15.0 mmol; ^{b} 4.0 mmol; ^{c} 2.0 mmol; ^{d} 1.0 mmol; ^{e} 0.36 mmol; ^{f} ^{1}H-NMR yield are calculated using 1,3,5-trimethoxybenzene as Internal Standard. ^{f} without light irradiation. ^{g} 4CzIPN used instead of Ru PS; ^{h} Low intensity blue light irradiation (Kessil lamps)

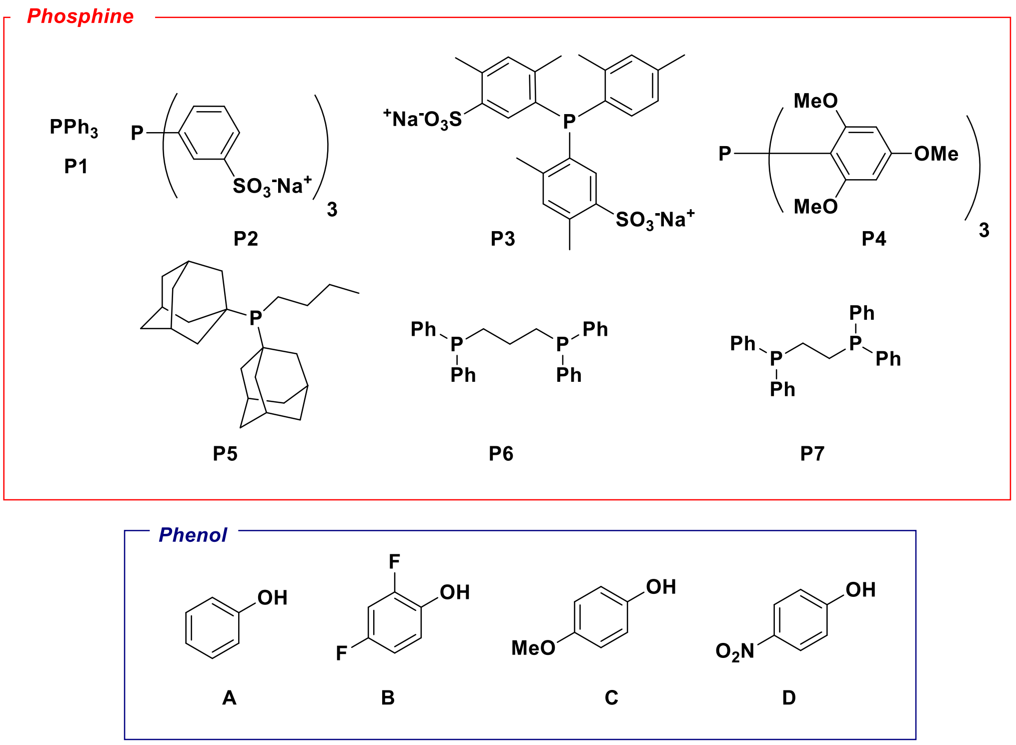

Supplementary Figure 7 Phosphines and phenols screened in the optimization. See Table S2 for details

Entry	Ru PS umol	Re Cat umol	BIH mmol	Proton Source^{a}	Phosphine^{b}	^{1}H-NMR Yield %^{c}
**1 (*Table S1*; Entry 27)**	1.62	1.62	0.78	**A**	**P1**	83 (75% isolated)
**2**	1.62	1.62	0.78	**A**	**P2**	82 (82% isolated)
**3**	1.62	1.62	0.78	**A**	**P3**	83
**4**	1.62	1.62	0.78	**A**	**P4**	67
**5**	1.62	1.62	0.78	**A**	**P5**	71
**6**	1.62	1.62	0.78	**A**	**P6**	46
**7**	1.62	1.62	0.78	**A**	**P7**	39
**8**	1.62	1.62	0.78	**B**	**P2**	82
**9**	1.62	1.62	0.78	**C**	**P2**	56
**10**	1.62	1.62	0.78	**D**	**P2**	0
**11**	1.62	1.62	0.78	**PhONa**	**P2**	0
**12**	-	1.62	0.78	**A**	**P2**	29
**13**	-	1.62	0.78	**A**	**-**	23

Supplementary Table 2 Screening of phosphines and phenols. All experiments are performed in DMF with an irradiation time of 1 h. [a] 2.0 mmol; [b] 0.036 mmol; [c] ^{1}H-NMR yields are calculated using 1,3,5-trimethoxybenzene as Internal Standard.

Acquisition Parameter

Source Type	ESI	Ion Polarity	Positive	Set Nebulizer	0.6 Bar
Scan Begin	50 m/z	Set Capillary	4500 V	Set Dry Heater	200 °C
Scan End	2500 m/z	Set Collision Cell RF	1800.0 Vpp	Set Dry Gas	7.0 l/min

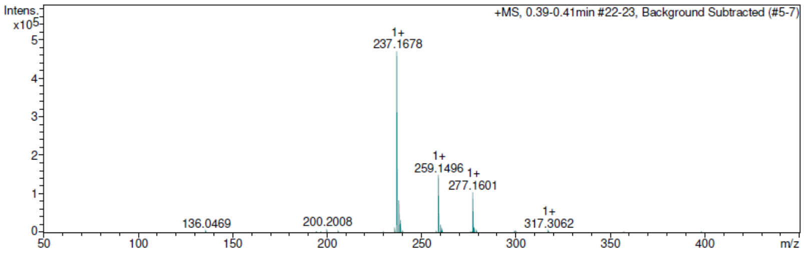

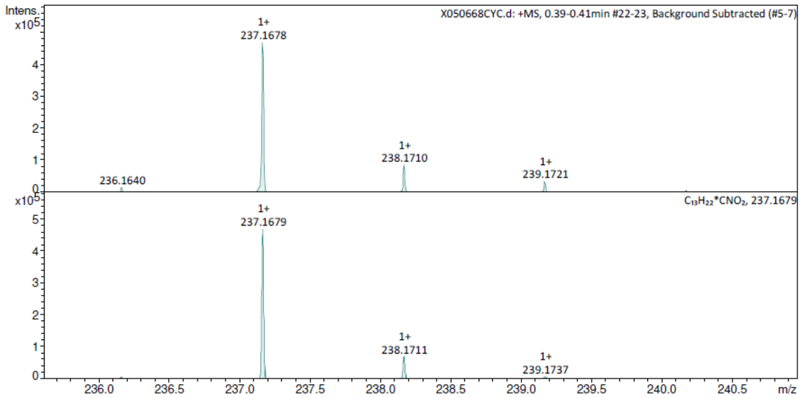

Isotopic Purity

X050668CYC - SM-108-1

Theoretical Natural Isotopic Distribution

Isotope	m/z	C0	C1	C2	C3	C4	C5
M	236	100					
M + 1	237	15.837	100				
M + 2	238	1.582	14.755				
M + 3	239	0.119	1.423				
M + 4	240	0.007	0.103				
M + 5	241		0.006				
M + 6	242						
M + 7	243						
M + 8	244						
M + 9	245						
M + 10	246						
M + 11	247						

Isotopomer	m/z	Area	Natural Isotope Correction	Corrected Area	Isotopic Enrichment Relative %	Total Labelled Atoms
0	236	214	0	214	2.67	0.00
1	237	7849	34	7815	97.33	97.33
2	238	1242	1157	85	1.06	2.13
3	239	500	111	389	4.84	14.52
4	240	84	8	76	0.95	3.78
5	241	0	0	0	-0.01	-0.03
6	242	0	0	0	0.00	0.00
7	243	0	0	0	0.00	0.00
8	244	0	0	0	0.00	0.00
9	245	0	0	0	0.00	0.00
10	246	0	0	0	0.00	0.00
11	247	0	0	0		
			Total	8029	100.00	97.33 Overall Isotopic Purity %

Supplementary Figure 8 Isotopic Enrichment of Reference Compound **[^{13}C]-1** (*Table S1;* Entry 16*)*

Pressure measurements of [^{13}C]CO_2 photoreduction

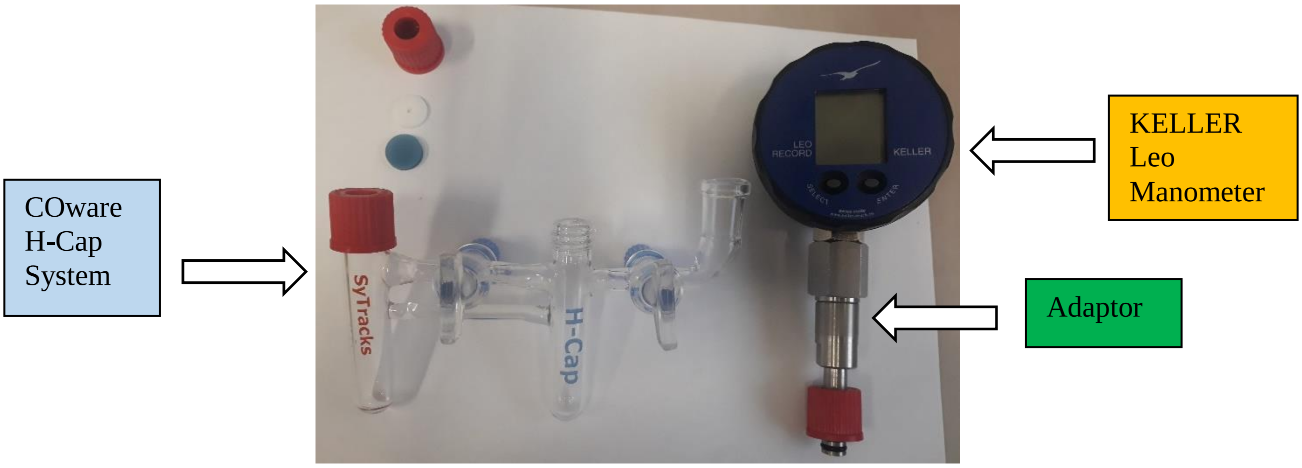

Supplementary Figure 9a Real-time pressure measurement set-up

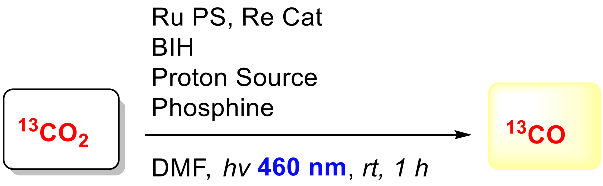

Supplementary Figure 9b CO_2 to CO reduction

Full Experiment Pressure Profile

For [^{13}C]CO_2 pressure measurements, **Glassware Type 3** (Figure S3) was used. The pressure measurement has been performed using the optimized condition for Chamber 1 (Entry 2, Table S2). A solution of $Ru(bpy)_3Cl_2.6H_2O$ stock solution (1.33 mL, 1.33 mg, 1.78 µmol), $Re(CO)_3(bpy)Br$ stock solution (0.9 mL, 0.9 mg, 1.78 µmol), DMF (0.89 mL), BIH (194 mg, 0.87 mmol), phosphine P**2** (22.5 mg, 39.6 µmol), **phenol** (2.22 mmol) is transferred in Chamber 1. Chamber 2 was sealed with a screwcap fitted with a Teflon® seal and a real-time pressure LEO manometer was connected to Chamber 1. The adaptor was then connected to the RC Tritec® system. The solution in Chamber 1 is frozen with a liquid nitrogen bath and the chambers are degassed with *vacuum* pump connected with RC Tritec manifold for 10 min. The stopcock is closed between the two chambers. [^{13}C]CO_2 (400 µmol) is then loaded into Chamber 1 using the RC Tritec® system and the stopcock is closed between Chamber 1 and the adaptor.

The Two-Chamber Glassware is then disconnected from the RC Tritec® system and the solution in Chamber 1 is warmed to room temperature. When the pressure inside of Chamber 1 is stable then Chamber 1 is placed *ca.* 2 cm away from a 40 W A160WE Tuna Blue Kessil® LED lamp and photo-irradiated with the lower light intensity (164 W m^{-2}) for 1 h.

Note: A stock solution of 1.34 mM $Ru(bpy)_3Cl_2.6H_2O$ was prepared by dissolving 1 mg in 1 mL dimethylformamide (DMF) or acetonitrile (ACN) where specified. A stock solution of 1.98 mM $Re(CO)_3(bpy)Br$ was prepared by dissolving 1 mg in 1 mL DMF or ACN where specified.

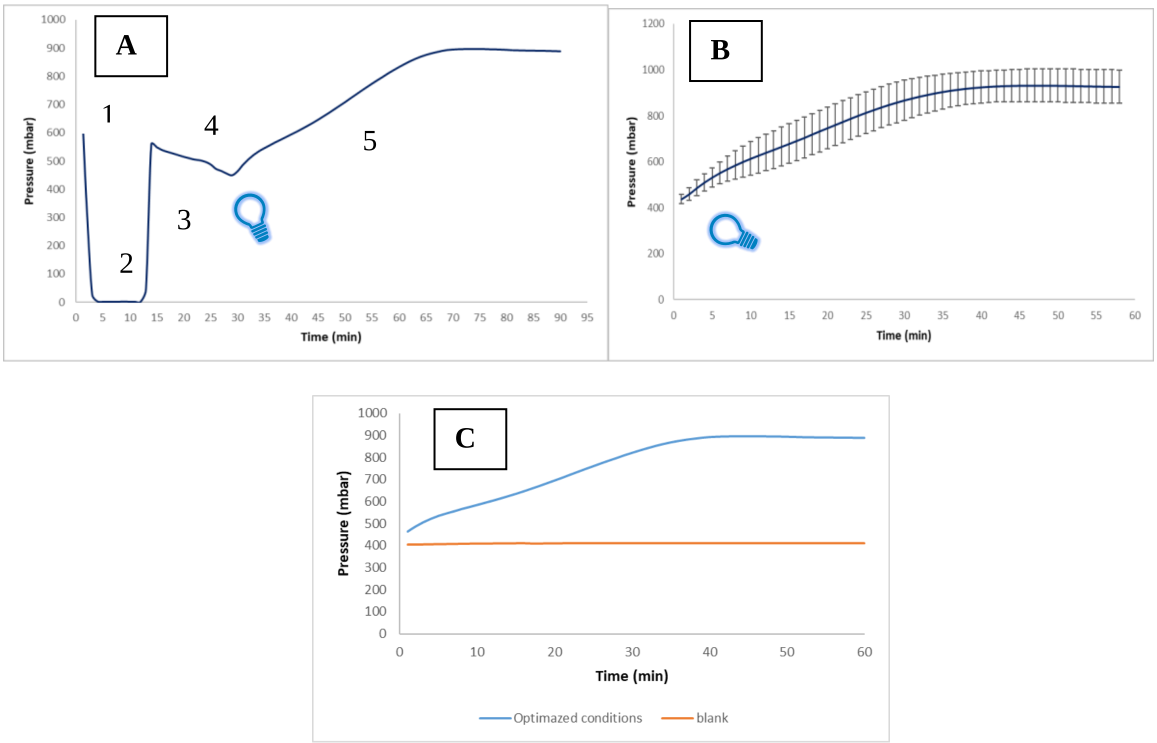

***Supplementary Figure 10* A**. Full pressure profile of $[^{13}C]CO_2$ photo-reduction using LEO manometer: 1) *vacuum* 2) $[^{13}C]CO_2$ loading 3) warming to room temperature 4) stabilization 5) photo-reduction. Carbonylation reaction yields 72% of compound **[^{13}C]-1**; **B**. $[^{13}C]CO_2$ photo-reduction monitoring using LEO manometer (with standard deviation, 5 values); **C.** Optimized condition (Table S2; Entry 2) *Vs* blank (condition: $[^{13}C]CO_2$ - DMF - 1 h irradiation)

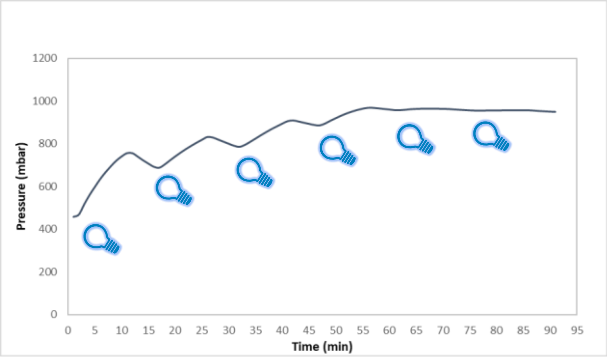

Supplementary Figure 11 On-Off experiment. Chamber 1 was placed ca. 2 cm away from a 40 W A160WE Tuna Blue Kessil® LED lamp and photo-irradiated with the lower light intensity (164 W m^{-2}). Six cycles of 10 min irradiation followed by 5 min of dark. After the 6th irradiation, a plateau is reached.

Recycling experiments

1) The pressure measurement has been performed using the optimized condition for Chamber 1 (Table S2, Entry 2). A solution of $Ru(bpy)_3Cl_2.6H_2O$ stock solution (1.33 mL, 1.33 mg, 1.78 µmol), of $Re(CO)_3(bpy)Br$ stock solution (0.9 mL, 0.9 mg, 1.78 µmol), DMF (0.89 mL), BIH (194 mg, 0.87 mmol), phosphine **P2** (22.5 mg, 39.6 µmol), **phenol** (2.22 mmol) is transferred in Chamber 1. Chamber 2 was sealed with a screwcap fitted with a Teflon® seal and a real-time pressure LEO manometer is connected to Chamber 1. The adaptor is then connected to the RC Tritec® system. The solution in Chamber 1 is frozen with a liquid nitrogen bath and the chambers are degassed with *vacuum* pump connected with RC Tritec manifold for 10 min. The stopcock was closed between the two chambers. $[^{13}C]CO_2$ (400 µmol) is then loaded into Chamber 1 using the RC Tritec® system and the stopcock was closed between Chamber 1 and the adaptor. The loaded Chamber 1 was then removed from the RC Tritec® system and the solution is warmed to room temperature. When the pressure inside of Chamber 1 is stable then Chamber 1 is placed *ca.* 2 cm away from a 40 W A160WE Tuna Blue Kessil® LED lamp and photo-irradiated with the lower light intensity (164 W m^{-2}) for 1 h. After the first irradiation, Chamber 1 is degassed into a *vacuum* pipe and BIH (194 mg, 0.87 mmol) is added to the solution. The adaptor is then reconnected to the RC Tritec® system. The solution in Chamber 1 is frozen with a liquid nitrogen bath and the chambers are degassed with *vacuum* pump connected with RC Tritec manifold for 10 min. The stopcock is closed between the two chambers. $[^{13}C]CO_2$ (400 µmol) is loaded into Chamber 1 using the RC Tritec® system and the stopcock is closed between Chamber 1 and the adaptor. The two-chamber glassware is then disconnected from the RC Tritec® system and the solution is warmed to room temperature. Chamber 1 is photo-irradiated in the second photocatalytic reaction for 1 h. The pressure does not increase (there is no CO production) in presence of additional BIH and $[^{13}C]CO_2$. The solution cannot be recycled by adding fresh BIH and $[^{13}C]CO_2$.

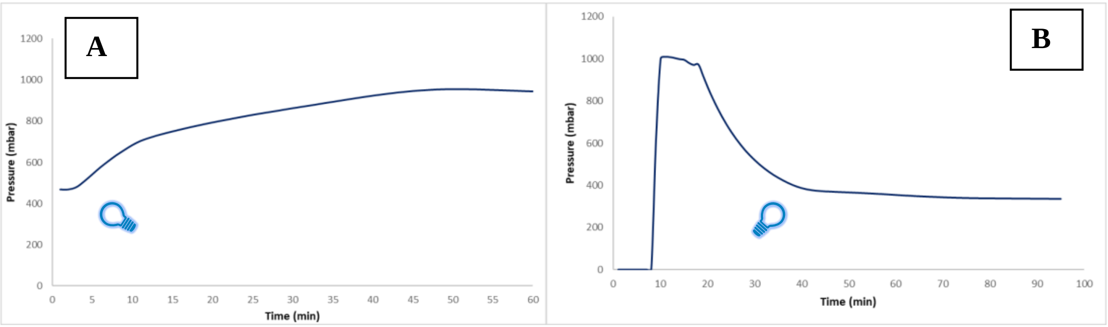

Supplementary Figure 12 **A**. First irradiation **B**. Second irradiation *via* addition of BIH and $[^{13}C]CO_2$. Note: There is no $[^{13}C]CO$ production.

2) The pressure measurement has been performed using the optimized condition for Chamber 1 (Table S2, Entry 2). A solution of $Ru(bpy)_3Cl_2.6H_2O$ stock solution (1.33 mL, 1.33 mg, 1.78 μmol), of $Re(CO)_3(bpy)Br$ stock solution (0.9 mL, 0.9 mg, 1.78 μmol), DMF (0.89 mL), BIH (194 mg, 0.87 mmol), phosphine P**2** (22.5 mg, 39.6 μmol), **phenol** (2.22 mmol) is transferred in Chamber 1. Chamber 2 is sealed with a screwcap fitted with a Teflon® seal and a real-time pressure LEO manometer is connected to Chamber 1. The adaptor is then connected to the RC Tritec® system. The solution in Chamber 1 is frozen with a liquid nitrogen bath and the chambers are degassed with *vacuum* pump connected with RC Tritec manifold for 10 min. The stopcock is closed between the two chambers. $[^{13}C]CO_2$ (400 μmol) is then loaded into Chamber 1 using the Tritec® system and the stopcock is closed between Chamber 1 and the adaptor. The Two-Chamber Glassware is then disconnected from the RC Tritec® system and the solution is warmed to room temperature. When the pressure inside of Chamber 1 is stable then Chamber 1 is placed *ca.* 2 cm away from a 40 W A160WE Tuna Blue Kessil® LED lamp and photo-irradiated with the lower light intensity (164 W m^{-2}) for 1 h. After the first irradiation, Chamber 1 is degassed and 16 mM solution of $Ru(bpy)_3Cl_2.6H_2O$ (0.11 mL, 1.33 mg, 1.78 μmol) is added to the solution. The adaptor was then reconnected to the RC Tritec® system. The solution in Chamber 1 is frozen with a liquid nitrogen bath and the chambers were degassed with *vacuum* pump connected with RC Tritec manifold for 10 min. The stopcock is closed between the two chambers. $[^{13}C]CO_2$ (400 μmol) is loaded into chamber 1 using the RC Tritec® system and the stopcock is closed between Chamber 1 and the adaptor. The Two-Chamber Glassware is then disconnected from the RC Tritec® system and the solution is warmed to room temperature. This chamber is photo-irradiated in the second photocatalytic reaction for 1 h. The pressure does not increase (there is no $[^{13}C]CO$ production) in presence of additional Ru PS. The solution cannot be recycled by adding fresh Ru PS.

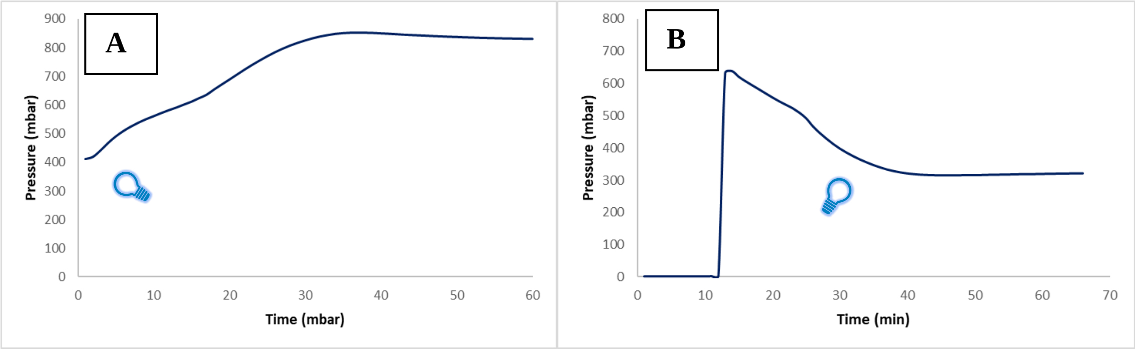

Supplementary Figure 13 **A.** First irradiation **B.** Second irradiation *via* addition of Ru PS and $[^{13}C]CO_2$. Note: There is no $[^{13}C]CO$ production.

Proton Source Screening

The pressure measurement has been performed using the optimized condition for Chamber 1 (Table S2 Entry 2). A solution of Ru(bpy)$_3$Cl$_2$.6H$_2$O stock solution (1.33 mL, 1.33 mg, 1.78 µmol), of Re(CO)$_3$(bpy)Br stock solution (0.9 mL, 0.9 mg, 1.78 µmol), DMF (0.89 mL), BIH (194 mg, 0.87 mmol), phosphine **P2** (22.5 mg, 39.6 µmol), **proton source** (2.22 mmol) is transferred in Chamber 1. Chamber 2 is sealed with a screwcap fitted with a Teflon® seal and a real-time pressure LEO manometer is connected to Chamber 1. The adaptor is then connected to the RC Tritec® system. The solution in Chamber 1 is frozen with a liquid nitrogen bath and the chambers are degassed with *vacuum* pump connected with RC Tritec manifold for 10 min. The stopcock is closed between the two chambers. [^{13}C]CO_2 (400 µmol) is then loaded into Chamber 1 using the RC Tritec® system and the stopcock was closed between Chamber 1 and the adaptor. The Two-Chamber Glassware is then disconnected from the RC Tritec® system and the solution is warmed to room temperature. When the pressure inside of Chamber 1 is stable then Chamber 1 is placed *ca.* 2 cm away from a 40 W A160WE Tuna Blue Kessil® LED lamp and photo-irradiated with the lower light intensity (164 W m^{-2}) for 1 h.

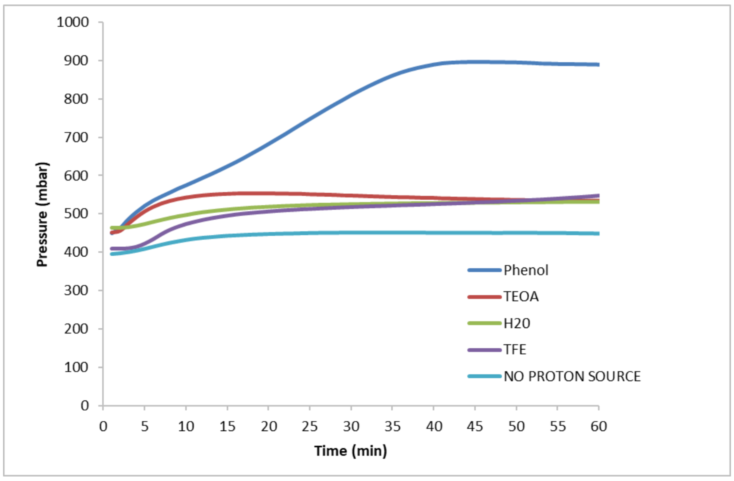

Supplementary Figure 14 Proton source screening in DMF at low light intensity using KELLER LEO manometer

Solvent and Light Intensity Screening

The pressure measurement has been performed using the optimized condition for Chamber 1 (Entry 27 Table S1). A suspension of $Ru(bpy)_3Cl_2.6H_2O$ stock solution (1.33 mL, 1.33 mg, 1.78 µmol), $Re(CO)_3(bpy)Br$ stock solution (0.9 mL, 0.9 mg, 1.78 µmol), DMF or ACN (0.89 mL), BIH (194 mg, 0.87 mmol), phosphine **P2** (22.5 mg, 39.6 µmol), **phenol** (2.22 mmol) is transferred in Chamber 1. The Chamber 2 was sealed with a screwcap fitted with a Teflon® seal and a real-time pressure LEO manometer was connected to the Chamber 1. The adaptor was then connected to the RC Tritec® system. The solution in Chamber 1 is frozen with a liquid nitrogen bath and the chambers are degassed with *vacuum* pump connected with RC Tritec manifold for 10 min. The stopcock is closed between the two chambers. $[^{13}C]CO_2$ (400 µmol) is then loaded into Chamber 1 using the RC Tritec® system and the stopcock is closed between Chamber 1 and the adaptor. The Two-Chamber Glassware is then disconnected from the RC Tritec® system and the solution is warmed to room temperature. When the pressure inside Chamber 1 is stable then Chamber 1 was placed *ca.* 2 cm away from a 40 W A160WE Tuna Blue Kessil® LED lamp and photo-irradiated for 1 h with the specified light intensity.

Phosphine **P2** has low solubility in acetonitrile. Reagents are mixed for a few minutes with a mini shaker. Do not sonicate for solubilizing (this will affect the reaction outcome).

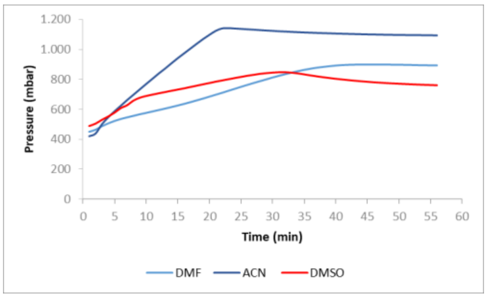

Supplementary Figure 15 Solvent effect at lower light intensity (164 W m^{-2}). Carbonylation reaction yields 72% of compound **[^{13}C]-1** in DMF and 82% in ACN**.**

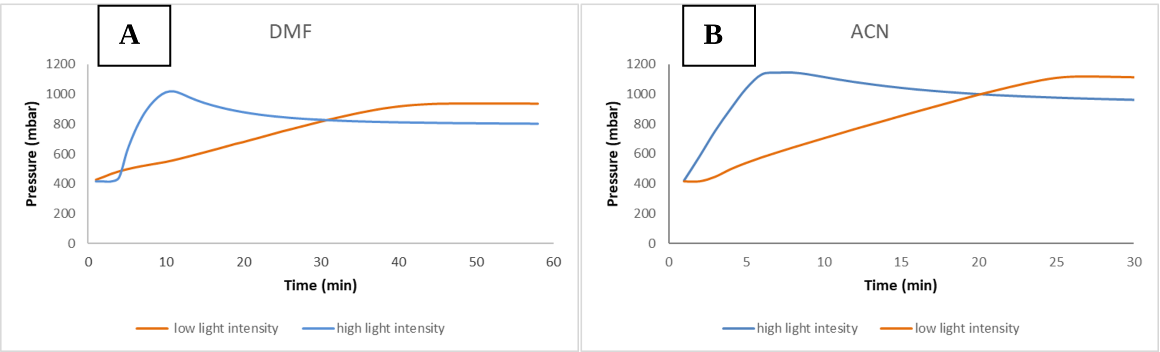

Supplementary Figure 16 **A**. Light intensity effect in DMF (low: 164 W m^{-2}, high 925 W m^{-2}). Carbonylation reaction yields 71% of compound **[^{13}C]-1** with higher light intensity; **B.** Light intensity effect in ACN. Carbonylation reaction yields 84% of compound **[^{13}C]-1** with higher light intensity.

Electrochemical Characterization

Cyclic voltammetry measurements were performed in an electrochemical cell composed of a glassy carbon (3 mm diameter) working electrode, Ag/AgNO$_3$ (10^{-2} M) reference electrode, and a platinum wire counter electrode. Dimethylformamide (DMF) was mainly used as solvent and solutions of samples were prepared at a concentration of 1 mM. Tetrabutylammonium hexafluorophosphate (Bu$_4$NPF$_6$) was used as supporting electrolyte and its concentration was maintained at a hundred-fold excess compared to the sample. Different additives (e.g. water, phenol, phosphine) were added and the solution was purged with inert argon gas (or carbon dioxide gas) and the cyclic voltammograms were measured at a scan rate of 100 mV/s.

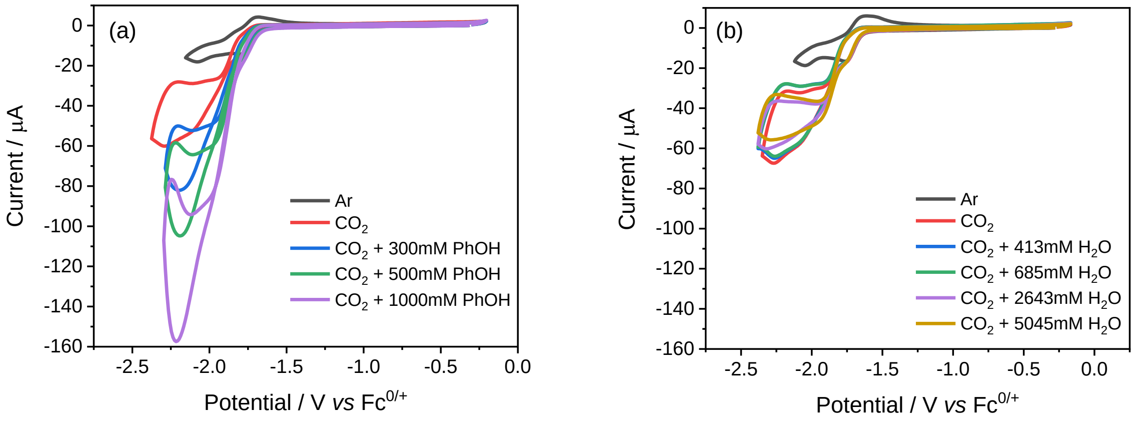

Supplementary Figure 17 Cyclic voltammograms with 1 mM Re catalyst in Ar- and CO_2-purged dimethylformamide solution at increasing concentrations of (a) phenol or (b) water. Comparison shows higher catalytic CO_2 reduction currents with phenol.

For the Re catalytic system, which undergoes a two-electron, two-proton ECEC mechanism, a catalytic rate constant (k_{cat}) can be estimated from the catalytic current (i_{cat}) of the cyclic voltammograms, normalized by the peak current in the absence of the substrate (i_p), using the following equation:[1,21]

$$\frac{i_{cat}}{i_p} = \frac{1}{0.446}\sqrt{\frac{RTnk_{cat}}{n'Fv}}$$

where R is the universal gas constant, T is the temperature, F is Faraday's constant, v is the scan rate, n is the number of unique electron transfer processes that occur at the electrode per catalyst (n = 2), and n' is the catalyst equivalents required per turnover (n' = 1). This equation was used to estimate and differentiate the effect of phenol and water on the electrocatalytic activities of the Re catalyst in CO_2-

purged solution at a scan rate of 100 mV/s. This method of estimation of k_{cat} was also recently recommended when dealing with Re-bpy-based catalysts, as it gives comparable results, rather than using the well-known foot-of-the-wave analysis (FOWA) employed for iron porphyrin catalysts.[3]

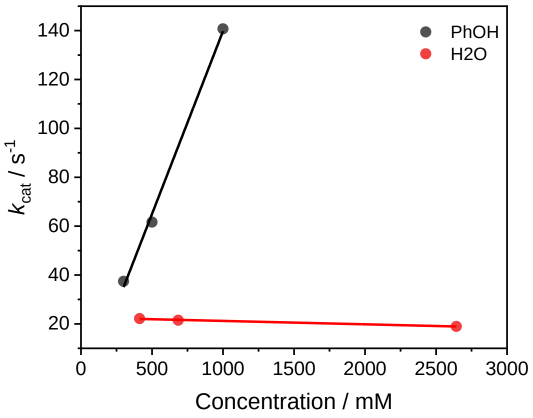

Supplementary Figure 18 Calculated rate constant (k_{cat}) for the electro-catalytic reduction of CO_2 by 1 mM Re catalyst in CO_2-purged dimethylformamide solution at increasing concentrations of (a) phenol or (b) water. Comparison shows higher catalytic CO_2 reduction rate with the use of phenol than water.

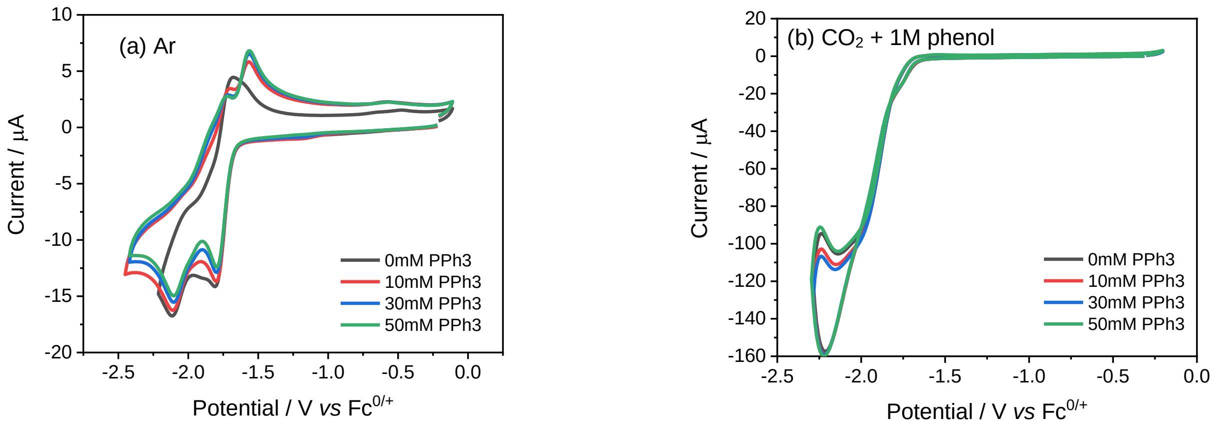

Supplementary Figure 19 Effect of increasing concentrations of triphenylphosphine (PPh_3) additive on the cyclic voltammograms with 1 mM Re catalyst in (a) Ar-purged dimethylformamide solution and (b) CO_2-purged dimethylformamide solution containing 1 M phenol. Comparison shows no significant effect of the PPh_3 additive in the reduction potentials of the Re catalyst, nor in the electrocatalytic reduction activity of the catalyst. This indicates that phosphines do not play a role in the catalytic redox cycle of Re.

Photo-irradiation Set-up

To excite only the ruthenium photosensitizer's metal-to-ligand charge transfer (MLCT) band at 460 nm and to prevent the sample from excessive heating, blue LED lamps were used. Two light sources were utilized in the experiments: (a) SugarCube® high intensity LED fiber optic illuminator with a blue light output centered at 463 nm with a full width at half maximum (FWHM) characteristic of about 50 nm, and (b) Kessil® A160WE Tuna Blue Aquarium Light. The irradiance from the LED sources to the photocatalytic cell was measured using a digital solar meter at different distances from the light source and the data are shown in Figure S20 and Table S3.

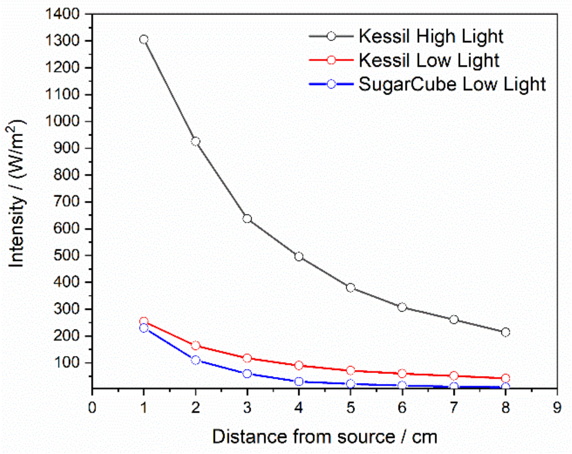

Supplementary Figure 20 Measured irradiance of the two blue LED lamps (SugarCube and Kessil) used in the photocatalytic experiments as a function of distance from the light source.

**Distance / cm**	**Irradiance / W m^{-2}**		
	**SugarCube (Intensity I)**	**Kessil Low Light Intensity (Min)**	**Kessil High Light Intensity (Max)**
1	230	254	1306
2	110	164	925
3	59	117	637
4	30	90	496
5	21	71	380
6	15	60	307
7	12	51	261
8	9	42	214

Supplementary Table 3 Irradiance data at various distances from the Blue LED lamps.

Product Analysis by Gas Chromatography

Analysis of gaseous products during the photocatalytic experiment was performed by withdrawing 50 μL gas aliquots from the headspace of the photocatalytic cell with a gas-tight syringe and injecting it into a gas chromatograph (GC - TraceGC Ultra, ThermoScientific). The GC is equipped with a 30 m molecular sieve porous layer open tubular (PLOT) column having an internal diameter of 0.53 mm, helium carrier gas, and a thermal conductivity detector (TCD). Peak separation between air (O_2+N_2), CO, and CO_2 were achieved by programming the oven temperature from an initial 100 °C, ramped by 10 °C/min until a final temperature of 180 °C, with a carrier gas flow rate of 4 ml/min. Air (O_2+N_2) was detected at 1.94 min, CO was detected at 2.07 min, and CO_2 was detected at 6.36 min. A splitless injector line was utilized to maximize the injection volume and become sensitive to possible trace amounts. A calibration curve relating peak area (or height) and concentration were established by injecting known amounts of the pure gases and determining their GC response (Figure S21).

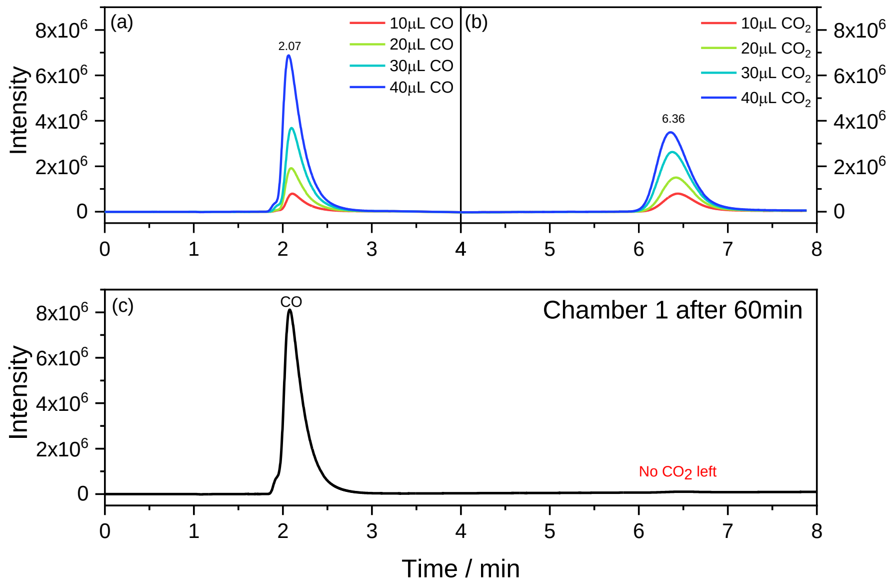

Supplementary Figure 21 Gas chromatograms showing the retention time and calibration of (a) carbon monoxide and (b) carbon dioxide. (c) The headspace analysis of the photocatalytic experiment after 60 min of photo-irradiation, showing only CO gas production**.**

To further determine the purity of the photocatalytic reduction of CO_2 to CO, the headspace sample was analyzed with a different column, which is more sensitive to the separation of H_2, O_2, N_2, and CO gases, as shown in Figure S22. The same oven temperature program, detector, carrier gas and gas flow rates

were maintained but the column was switched to Rt-Msieve 5A (molecular sieve fused silica PLOT) column having a smaller internal diameter of 0.32 mm. With this column, H_2 can be detected at 3.63 min, O_2 at 4.32 min, N_2 at 5.00 min, and CO at 6.82 min.

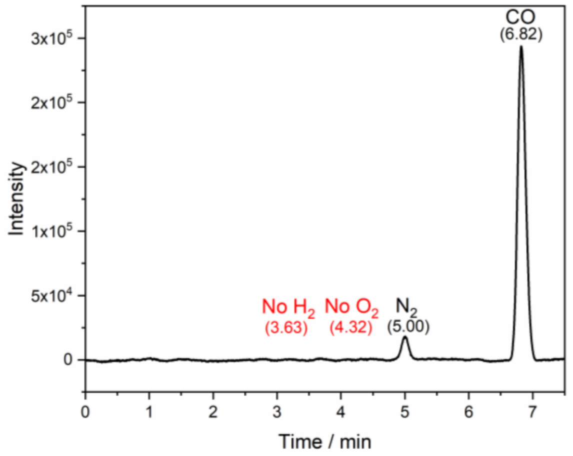

Supplementary Figure 22 Gas chromatogram of the headspace analysis of the photocatalytic experiment after 60 min of photo-irradiation, showing only CO gas production, and no H_2 production.

Kinetic Simulation

The pressure measurements during the photo-irradiation of the catalytic system were simulated using a model-based fitting software, *Kintek Global Kinetic Explorer*,[4,5] to gain insights into the reaction steps involved in the photocatalytic cycle and to rationalize the observed differences when using phenol or water as an additive.

The experimental conditions are as follows:

(i) Using phenol: The first chamber of the two-chamber system was utilized for this control experiment. Chamber 1 contains 0.57 mM Ru, 0.57 mM Re, 279 mM BIH, 12.7 mM sodium phosphinidynetris(benzene sulfonate) and 712 mM phenol in dimethylformamide. Isotopically labeled $[^{13}C]CO_2$ (400 µmol) was introduced into the system using a RC Tritec® equipment.[6] This was irradiated using a Kessil lamp (low light intensity), at a distance of 3 cm (irradiance of 117 W m^{-2}).

(ii) Using H_2O: The first chamber of the two-chamber system was utilized for this control experiment. Chamber 1 contains 0.57 mM Ru, 0.57 mM Re, 279 mM BIH, 12.7 mM sodium phosphinidynetris(benzene sulfonate) and 712 mM water in dimethylformamide. Isotopically labeled $[^{13}C]CO_2$ (400 µmol) was introduced into the system using a RC Tritec® equipment.[6] This was irradiated using a Kessil lamp (low light intensity), at a distance of 3 cm (irradiance of 117 W m^{-2}).

The proposed photocatalytic cycle, upon which the simulation was based, is shown in Scheme 3 in the manuscript and reiterated in Figure S23 below. The initial reaction steps (1, 2, 3, 4, A, B, C) have been mechanistically probed by transient absorption studies in our previous report,[6] and are in good agreement with accepted literature knowledge.[7] The proceeding steps in the catalytic cycle (5, 6, 7, 8, 10, 11) were based on reported mechanistic DFT calculations.[8,9] Though other pathways are proposed within the catalytic cycle (*e. g.*, electron transfer first *vs* protonation first of the $ReCO_2H$ intermediate, Reaction 7 *vs* 8), the one presented and chosen for the model-based fitting was the one thermodynamically more accessible.

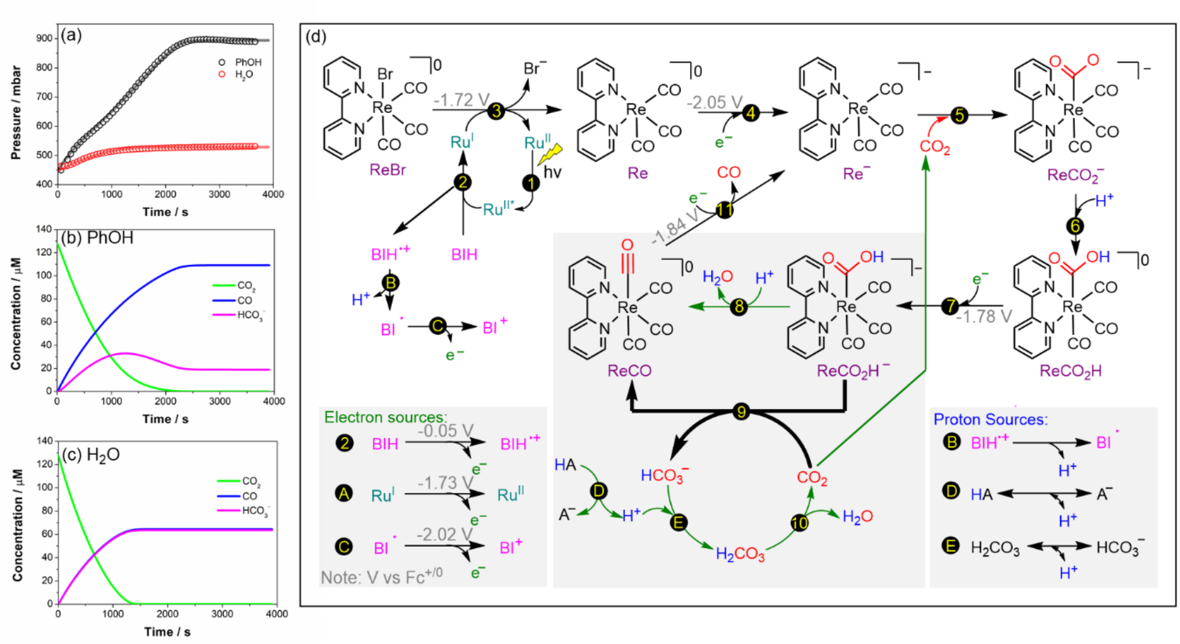

Supplementary Figure 23 Proposed photocatalytic cycle for the bimolecular system consisting of a ruthenium photosensitizer, rhenium catalyst, and BIH as electron donor. Effect of water as proton source is indicated in thick black arrows accumulating bicarbonate while the changes when phenol is used is indicated in green arrows.

Interconversion between pressure and concentration of gases

The translation of the observed pressure reading with the concentration of the gases is established by taking into account the solubility of the gases in the solvent, the respective headspace and solution volumes, and the equilibration due to pressure changes.

Solubility of CO_2 in DMF (mol L^{-1} atm^{-1})[10,11]: $S_{CO2} = 0.187$
Solubility of CO in DMF (mol L^{-1} atm^{-1})[12]: $S_{CO} = 0.01$

Volume of solution (L): $V_{sol} = 3.12 \times 10^{-3}$
Volume of headspace (L): $V_{HS} = 6.88 \times 10^{-3}$

Concentration (M) of any gas at 1 bar, 298 K: $C_g = 40 \times 10^{-3}$
Effective initial concentration of CO_2 (M): $C_{CO2} = 40 \times 10^{-3}$ M * (1 L / 3.12×10^{-3} L) = 0.128
*Assuming all injected CO_2 goes into the solution

Amount of gas in solution as a function of solubility and pressure: $n_{sol} = S * V_{sol} * P$
Amount of gas in headspace as a function of pressure: $n_{HS} = V_{HS} * P * C_g$
Total amount of gas in solution and headspace: $n_{tot} = n_{sol} + n_{HS}$
Total amount of CO_2 (mol) used in the experiment: $n_{tot} = 360 \times 10^{-6}$

For CO_2:
$$P_{CO2}(\text{total}) = \frac{n_{tot}}{V_{sol} \times S_{CO2} + V_{HS} \times C_g} = 0.419$$
$n_{CO2,sol} = 2.446 \times 10^{-3}$
$n_{CO2,HS} = 1.154 \times 10^{-4}$

For CO:
$$P_{CO,tot} = \frac{n_{tot}}{V_{sol} \times S_{CO} + V_{HS} \times C_g} = 1.175$$
$n_{CO,sol} = 3.666 \times 10^{-5}$
$n_{CO,HS} = 3.233 \times 10^{-4}$

Parameter p relating CO concentration and pressure in the fit (assuming 100% conversion of CO_2 to CO):
$p = P_{CO,tot} / C_{CO2} = 9.164$ (for bar, M) = 9.164×10^{-3} (for mbar, µM)

Parameter q relating CO_2 concentration and pressure in the fit:
$q = P_{CO2,tot} / C_{CO2} = 3.27$ (for bar, M) = 3.27×10^{-3} (for mbar, µM)

As such the pressure was fitted with the relation: $P_{fit} = C_{CO}*p + C_{CO2}*q + \text{offset}$
Fitting parameter results (mbar, µM) agree with experimental parameters: $p = 8.57 \times 10^{-3}$, q = 3 .642 × 10^{-3}, offset = 20.46

When using PhOH as additive: $P_{CO} = 0.893$ corresponding to 76% conversion
When using H_2O as additive: $P_{CO} = 0.531$ corresponding to 45% conversion

Entry	Reaction	$k_{forward}$	$k_{reverse}$
1	Ru^{II} + light $\rightarrow$ Ru^{II*}	2.01×10^{-1} s^{-1}	-
2	Ru^{II*} + BIH $\rightarrow$ Ru^{I} + $BIH^{\bullet+}$	1×10^{9} M^{-1} s^{-1}	-
C	$BIH^{\bullet+}$ $\rightarrow$ $BI^{\bullet}$ + H^{+}	35700 s^{-1}	-
1B	Ru^{II} + $BI^{\bullet}$ $\rightarrow$ Ru^{I} + BI^{+}	0.450×10^{9} M^{-1} s^{-1}	-
3A	ReBr + Ru^{I} $\rightarrow$ Re + Ru^{II}	0.422×10^{9} M^{-1} s^{-1}	1×10^{9} M^{-1} s^{-1}
3B	ReBr + $BI^{\bullet}$ $\rightarrow$ Re + BI^{+}	0.450×10^{9} M^{-1} s^{-1}	-
4A	Re + Ru^{I} $\rightarrow$ Re^{-} + Ru^{II}	0.188×10^{9} M^{-1} s^{-1}	1×10^{9} M^{-1} s^{-1}
4B	Re + $BI^{\bullet}$ $\rightarrow$ Re^{-} + BI^{+}	0.450×10^{9} M^{-1} s^{-1}	-
5	Re^{-} + CO_2 $\rightarrow$ $ReCO_2^{-}$	0.000511×10^{9} M^{-1} s^{-1}	1×10^{9} M^{-1} s^{-1}
6	$ReCO_2^{-}$ + H^{+} $\rightarrow$ $ReCO_2H$	1×10^{9} M^{-1} s^{-1}	-
7A	$ReCO_2H$ + Ru^{I} $\rightarrow$ $ReCO_2H^{-}$ + Ru^{II}	1×10^{9} M^{-1} s^{-1}	0.225×10^{9} M^{-1} s^{-1}
7B	$ReCO_2H$ + $BI^{\bullet}$ $\rightarrow$ $ReCO_2H^{-}$ + BI^{+}	0.45×10^{9} M^{-1} s^{-1}	-
8	$ReCO_2H^{-}$ + H^{+} $\rightarrow$ ReCO + H_2O	0.0134×10^{9} M^{-1} s^{-1}	1×10^{9} M^{-1} s^{-1}
9	$ReCO_2H^{-}$ + CO_2 $\rightarrow$ ReCO + HCO_3^{-}	0.00497×10^{9} M^{-1} s^{-1}	1×10^{9} M^{-1} s^{-1}
10	CO_2 + H_2O $\rightarrow$ H_2CO_3	0.00193×10^{9} M^{-1} s^{-1}	1.6×10^{9} s^{-1}
Ei	H_2CO_3 $\rightarrow$ H^{+} + HCO_3^{-}	21700 s^{-1}	1×10^{9} M^{-1} s^{-1}
Eii[13]	HCO_3^{-} + BI^{+} $\rightarrow$ $HCO_3^{-}BI^{+}$	$(1.05 \times 10^{-11}) \times 10^{9}$ M^{-1} s^{-1}	-
D	AH $\rightarrow$ A^{-} + H^{+}	0.0662 s^{-1}	1×10^{9} M^{-1} s^{-1}
11Ai	ReCO + Ru^{I} $\rightarrow$ $ReCO^{-}$ + Ru^{II}	1×10^{9} M^{-1} s^{-1}	0.00364×10^{9} M^{-1} s^{-1}
11Bi	ReCO + $BI^{\bullet}$ $\rightarrow$ $ReCO^{-}$ + BI^{+}	0.450×10^{9} M^{-1} s^{-1}	-
11ii	$ReCO^{-}$ $\rightarrow$ Re^{-} + CO	0.137 s^{-1}	-

Supplementary Table 4 Simulated rate constants from the reaction-model-based kinetic simulation of the pressure data.

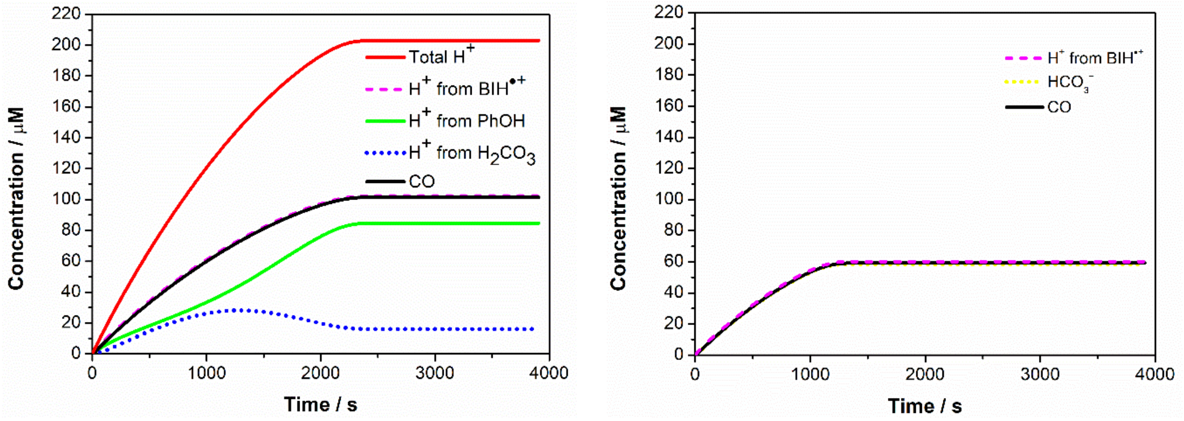

Supplementary Figure 24 Concentration profiles for the total protons (red), as well as their relative distribution from phenol (green), $BIH^{\bullet+}$ (magenta) and H_2CO_3 (blue), are utilized for the catalytic production of CO (black) when using phenol (left) or water (right) as an additive. Protons are needed to capture one of the oxygens of CO_2: if this oxygen goes to water, two protons are needed ($CO_2 + 2e^{-} + 2H^{+} \rightarrow CO + H_2O$); if it ends up in bicarbonate, only one proton is needed per CO, plus one equivalent of CO_2 ($2CO_2 + 2e^{-} + H^{+} \rightarrow CO + HCO_3^{-}$). When using phenol as an additive (left), the total amount of protons utilized for the reaction is coming from the deprotonation of $BIH^{\bullet+}$ (magenta) and is complemented by the protons coming from phenol itself, and to some extent, from the formed carbonic

acid (once water is formed during the cycle). In the case of using water (right), only one proton is needed per CO, and this comes from the deprotonation of $BIH^{\bullet+}$ (magenta), and an equivalent amount of bicarbonate is formed (yellow).

Preparation of starting materials

Re(CO)$_3$(bpy)Br (Re Cat)

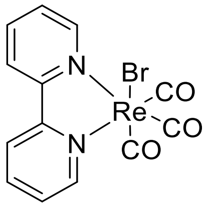

$C_{13}H_8BrN_2O_3Re$
MW: 506.33 g.mol^{-1}
Yield: 92%
yellow solid

Re(CO)$_5$Br (0.345 g, 0.85 mmol) and 2,2’ -bipyridine (0.136 g, 0.87 mmol) was dissolved in toluene (50 mL) and heated to 115 °C. The reaction mixture was stirred under reflux for 2 h. Afterward, the solution was removed from the heat source and cooled down by placing it in an ice bath. The product precipitated from the solution as a yellow powder and it was filtered off. The pure product was obtained with 92% overall yield (0.326 g).[14]

^{1}H NMR (200 MHz, CD$_3$CN) δ 7.62 (t, 2H), 8.18 (t, 2H), 8.41 (d, 2H), 9.00 (d, 2H).
HRMS (ESI) ***m/z*** $C_{13}H_8BrN_2O_3Re$ [M+Na$^+$]$^+$ calcd 528.9168, found 528.9143.

Synthesis of BIH:

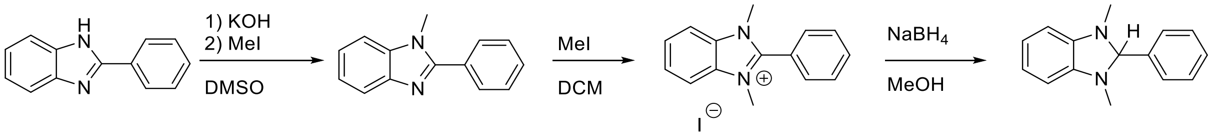

1-methyl-2-phenyl-1H-benzo[d]imidazole

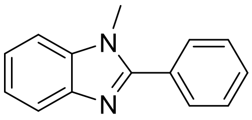

$C_{14}H_{12}N_2$
MW: 208.26 g.mol^{-1}
Yield: 90%
beige solid

KOH (5.8 g, 103 mmol) was dissolved in 100 mL of DMSO and stirred for 30 minutes. A solution of 2-phenyl-1H-benzo[d]imidazole (10 g, 51.4 mmol) in DMSO was added and the resulting was stirred for 1 h. Methyliodide (4 mL, 61.7 mmol) was added and the mixture was stirred for 45 minutes. The reaction mixture was then poured into a solution of KOH in H_2O and Et_2O and the organic layer was decanted.

The process was then repeated another time. The organic phase was washed with water, brine, water, dried over $MgSO_4$ and evaporated to give the corresponding compound (9.64 g, 90%).

^{1}H NMR (400 MHz, DMSO-d_6) δ 7.88 – 7.83 (m, 2H), 7.71 – 7.66 (m, 1H), 7.63 – 7.60 (m, 1H), 7.60 – 7.54 (m, 3H), 7.33 – 7.23 (m, 2H), 3.88 (s, 3H).

1,3-dimethyl-2-phenyl-1H-benzo[d]imidazol-3-ium iodide

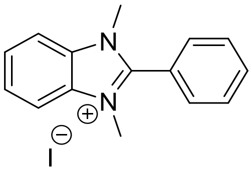

$C_{15}H_{15}IN_2$
MW: 350.2 g.mol^{-1}
Yield: 80%
beige solid

1-methyl-2-phenyl-1H-benzo[d]imidazole (9.64 g, 46.0 mmol) was dissolved in 100 mL of DCM under stirring, MeI (11.5 mL, 185 mmol) was added and the mixture was stirred at 30 °C for 19 h. The solvent was then removed under reduced pressure. Et_2O was added and the suspension was filtered to give the pure compound (12.87 g, 80%).

^{1}H NMR (400 MHz, DMSO-d_6) δ 8.17 – 8.11 (m, 2H), 7.93 – 7.89 (m, 2H), 7.88 – 7.82 (m, 1H), 7.82 – 7.75 (m, 4H), 3.90 (s, 6H).

1,3-dimethyl-2-phenyl-2,3-dihydro-1H-benzo[d]imidazole

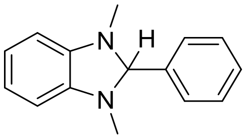

$C_{15}H_{16}N_2$
MW: 224.3 g.mol^{-1}
Yield: 95%
beige solid

To a suspension of 1,3-dimethyl-2-phenyl-1H-benzo[d]imidazol-3-ium iodide (12.87 g, 36.8 mmol) in 180 mL of dry MeOH, was added at 0 °C $NaBH_4$ (3.5 g, 92 mmol) by portion. The mixture was allowed to warm up to room temperature and stirred for 1 h. The solvent was removed under reduced pressure and the residue was dissolved in water and extracted twice with Et_2O, dry over $MgSO_4$ and evaporated. The solid was then recrystallized from EtOH : H_2O (2:1) to give the crystalline product (7.86 g, 95%).

^{1}H NMR (400 MHz, $CDCl_3$) δ 7.62 – 7.52 (m, 2H), 7.48 – 7.36 (m, 3H), 6.77 – 6.70 (m, 2H), 6.48 – 6.38 (m, 2H), 4.89 (s, 1H), 2.57 (s, 6H).

Synthetic Procedure and Analytical Data

General Procedure

General Procedure for the [^{13}C]CO_2 photoreduction (GP0):

- **Glassware Type 1**

A suspension of $Ru(bpy)_3Cl_2.6H_2O$ stock solution (1.20 mL), $Re(CO)_3(bpy)Br$ stock solution (0.81 mL), ACN (0.8 mL), BIH (175 mg, 0.78 mmol), phosphine P**2** (20.5 mg, 0.036 mmol), phenol (188 mg, 2.0 mmol) were transferred into Chamber 1 with a Pasteur pipette. The chambers were sealed with a screwcap fitted with a Teflon®. The adaptor was then connected to the RC Tritec® system. The solution in Chamber 1 was frozen with a liquid nitrogen bath and the chambers were degassed with *vacuum* pump connected with RC Tritec manifold for 10 min. The stopcock was closed between the two chambers. [**^{13}C]CO_2** (365 μmol) was then loaded into Chamber 1 using the RC Tritec® system and the stopcock was closed between Chamber 1 and the adaptor. The loaded Two-Chamber Glassware was then disconnected from the RC Tritec® system and the suspension was warmed to room temperature. Chamber 1 was placed *ca.* 2 cm away from a 40 W A160WE Tuna Blue Kessil® LED lamp and photo-irradiated with the lower light intensity for 1 h. The **[^{13}C]CO** produced is then used in the carbonylation reaction in Chamber 2.

- **Glassware Type 2**

A suspension of $Ru(bpy)_3Cl_2.6H_2O$ stock solution (1.46 mL), $Re(CO)_3(bpy)Br$ stock solution (0.99 mL), ACN (0.98 mL), BIH (213 mg, 0.95 mmol), phosphine P**2** (25.0 mg, 0.044 mmol), phenol (230 mg, 2.44 mmol) were transferred into Chamber 1 with a Pasteur pipette. The chambers were sealed with a screwcap fitted with a Teflon®. The adaptor was then connected to the RC Tritec® system. The solution in Chamber 1 was frozen with a liquid nitrogen bath and the chambers were degassed with *vacuum* pump connected with RC Tritec manifold for 10 min. The stopcock was closed between the two chambers. **[^{13}C]CO_2** (445 μmol) was then loaded into Chamber 1 using the RC Tritec® system and the stopcock was closed between Chamber 1 and the adaptor. The loaded Two-Chamber Glassware was

then disconnected from the RC Tritec® system and the suspension was warmed to room temperature. Chamber 1 was placed *ca.* 2 cm away from a 40 W A160WE Tuna Blue Kessil® LED lamp and photo-irradiated with the lower light intensity for 1 h.

The **[^{13}C]CO** produced is then used in the carbonylation reaction in Chamber 2.

Note: *A* stock solution of 1.34 mM Ru(bpy)$_3$Cl$_2$.6H$_2$O was prepared by dissolving 1 mg in 1 mL dimethylformamide (DMF) or in acetonitrile (ACN). A stock solution of 1.98 mM Re(CO)$_3$(bpy)Br was prepared by dissolving 1 mg in 1 mL DMF or in ACN. Phosphine P**2** has low solubility in acetonitrile. The reagents are mixed for a few minutes with a mini shaker maker.

Do not sonicate for solubilizing (this will affect the reaction outcome).

During the revision process, a Reviewer asked to evaluate the effect of increasing the equivalents of [^{13}C]CO_2 the yield of the carbonylation reaction.
We have selected amide **[^{13}C]6** and ketone **[^{13}C]21**, that were previously isolated in 53% and 34% yield. The reactions were performed using 600 µmol of [^{13}C]CO_2 in chamber 1 (catalyst loading was increased accordingly, seen procedures reported right afterwards), while in chamber B (carbonylative reaction) the conditions were identical.
Under higher stoichiometry of CO the yields were increased in both case to 65% and 88%.

General Procedure for the 0.6 mmol [^{13}C]CO_2 photoreduction (GP0 bis):

- **Glassware Type 1** : this procedure was used for amide **[^{13}C]6**

A suspension of Ru(bpy)$_3$Cl$_2$.6H$_2$O stock solution (2.40 mL), Re(CO)$_3$(bpy)Br stock solution (1.6 mL), BIH (350 mg, 1.56 mmol), phosphine P**2** (41.0 mg, 0.072 mmol), phenol (376 mg, 4.0 mmol) were transferred into Chamber 1 with a Pasteur pipette. The chambers were sealed with a screwcap fitted with a Teflon®. The adaptor was then connected to the RC Tritec® system. The solution in Chamber 1 was frozen with a liquid nitrogen bath and the chambers were degassed with *vacuum* pump connected with RC Tritec manifold for 10 min. The stopcock was closed between the two chambers. [**^{13}C]CO_2** (600 µmol) was then loaded into Chamber 1 using the RC Tritec® system and the stopcock was closed between Chamber 1 and the adaptor. The loaded Two-Chamber Glassware was then disconnected from the RC Tritec® system and the suspension was warmed to room temperature. Chamber 1 was placed *ca.* 2 cm away from a 40 W A160WE Tuna Blue Kessil® LED lamp and photo-irradiated with the lower light intensity for 1 h. The **[^{13}C]CO** produced is then used in the carbonylation reaction in Chamber 2.

- **Glassware Type 2** : this procedure was used for ketone **[^{13}C]21**

A suspension of Ru(bpy)$_3$Cl$_2$.6H$_2$O stock solution (3.0 mL), Re(CO)$_3$(bpy)Br stock solution (2 mL), BIH (426 mg, 1.9 mmol), phosphine P**2** (50.0 mg, 0.088 mmol), phenol (460 mg, 4.88 mmol) were transferred into Chamber 1 with a Pasteur pipette. The chambers were sealed with a screwcap fitted with a Teflon®. The adaptor was then connected to the RC Tritec® system. The solution in Chamber 1 was frozen with a liquid nitrogen bath and the chambers were degassed with *vacuum* pump connected with RC Tritec manifold for 10 min. The stopcock was closed between the two chambers. **[^{13}C]CO$_2$** (600 μmol) was then loaded into Chamber 1 using the RC Tritec® system and the stopcock was closed between Chamber 1 and the adaptor. The loaded Two-Chamber Glassware was then disconnected from the RC Tritec® system and the suspension was warmed to room temperature. Chamber 1 was placed *ca.* 2 cm away from a 40 W A160WE Tuna Blue Kessil® LED lamp and photo-irradiated with the lower light intensity for 1 h. The **[^{13}C]CO** produced is then used in the carbonylation reaction in Chamber 2.

Note: *A* stock solution of 1.34 mM Ru(bpy)$_3$Cl$_2$.6H$_2$O was prepared by dissolving 1 mg in 1 mL dimethylformamide (DMF) or in acetonitrile (ACN). A stock solution of 1.98 mM Re(CO)$_3$(bpy)Br was prepared by dissolving 1 mg in 1 mL DMF or in ACN. Phosphine P**2** has low solubility in acetonitrile. The reagents are mixed for a few minutes with a mini shaker maker.

Do not sonicate for solubilizing (this will affect the reaction outcome).

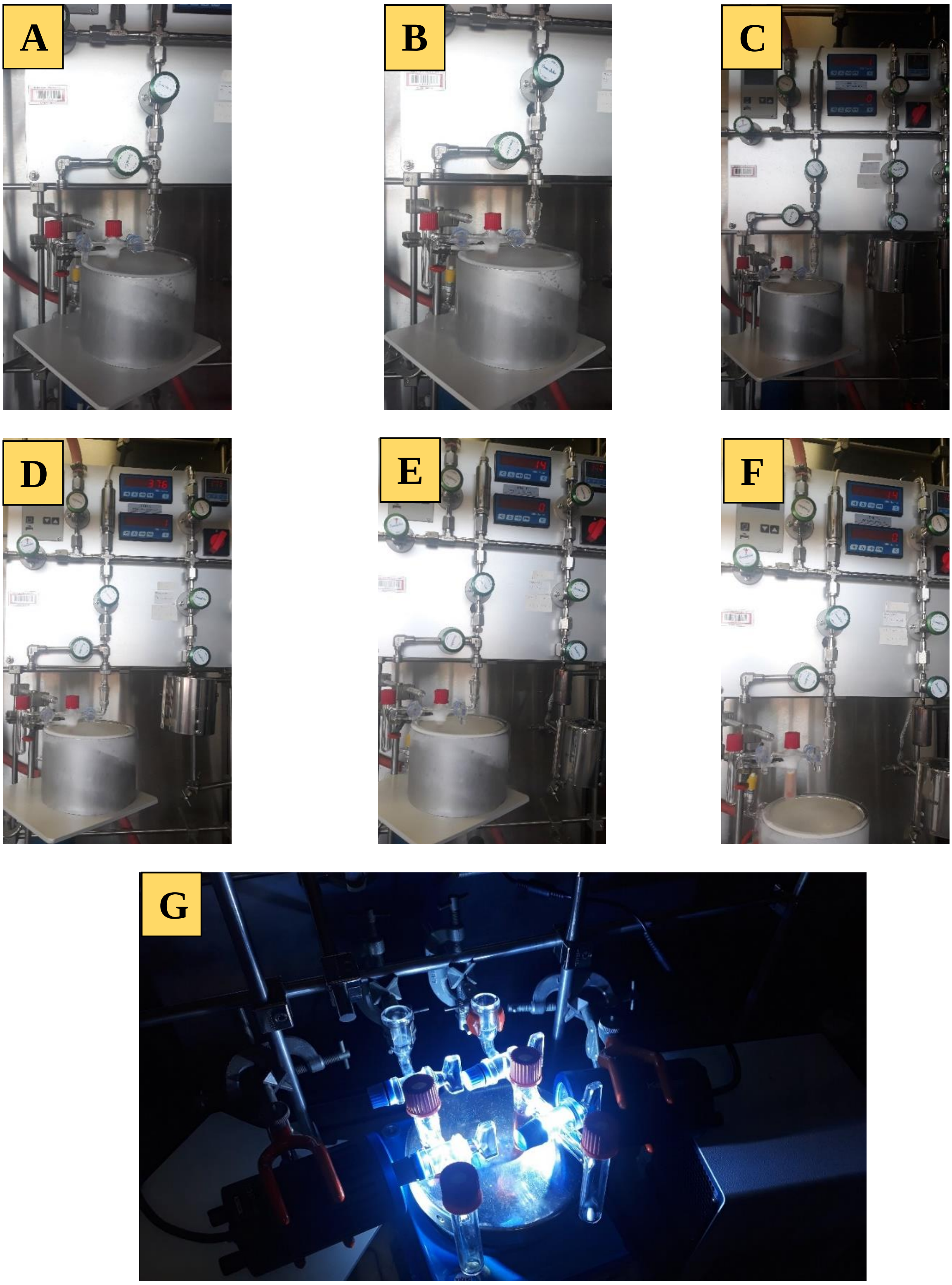

Supplementary Figure 25 **A** Two-chamber glassware is connected with RC TRITEC, the solution in chamber 1 is frozen with N_2 bath and a *vacuum* is created with RC TRITEC pump. **B**. *Vacuum* is created in chamber 2 **C.** Stopcock between chambers 1 and 2 is closed **D.** $[^{13}C]CO_2$ is charged into TRITEC by warming the cartridge with the oven at 300 °C until reaching the pressure value correspondent to the amount of $[^{13}C]CO_2$ desired (376 mbar = 0.376 mmol of $[^{13}C]CO_2$). **E.** Trapping of $[^{13}C]CO_2$ into Chamber 1 and the stopcock between chamber 1 and RC TRITEC connection is closed **F.** N_2 bath is removed and the solution is allowed to go at room temperature **G.** The two-chamber glassware is disconnected from the RC TRITEC and chamber 1 is photo-irradiated.

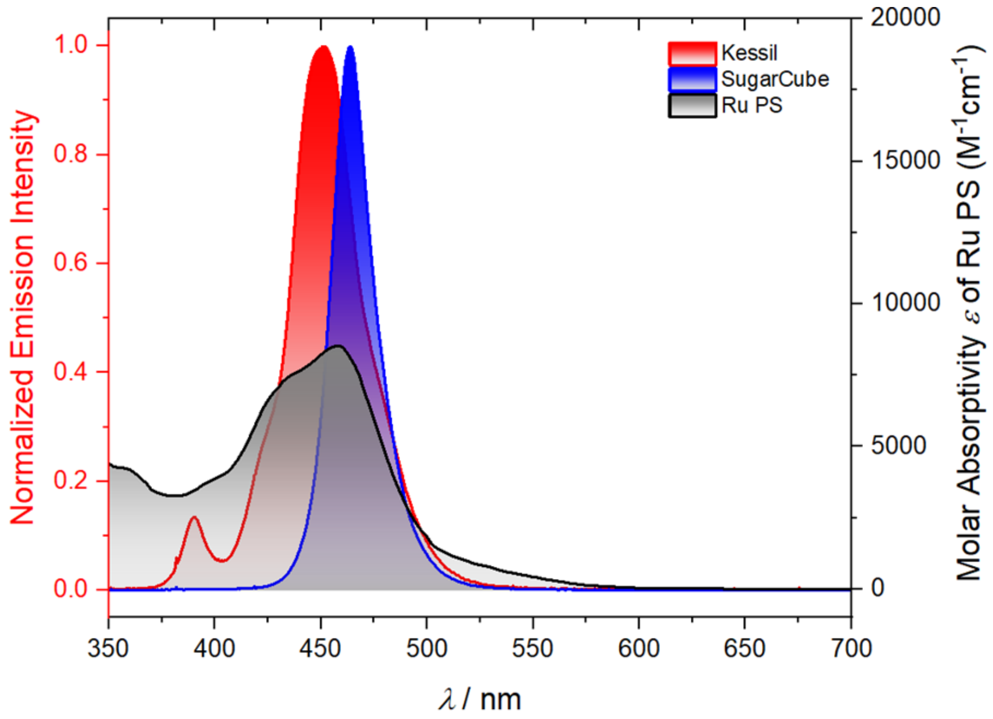

Supplementary Figure 26 : *Normalized emission spectra of the Kessil A160WE Tuna Blue, LED lamp® (red) and SugarCube (blue, used in previous study, see: ChemPhotoChem* ***2018****, 2, 715) LED lamps, as well as the molar absorptivity profile of the ruthenium photosensitizer (black).*

General Procedure for the Aminocarbonylation (GP1):[15]

Glassware Type 1 (Figure S1) was used unless otherwise specified. To a solution of aryl iodide (0.724 mmol), Pd(dba)$_2$ (8.7 mg, 0.015 mmol, 2 mol%), P(*t*Bu)$_3$HBF$_4$ (8.7 mg, 0.030 mmol, 4 mol%), DABCO (162 mg, 1.45 mmol) in dry THF (3 mL) is added amine (1.448 mmol) under argon. The solution is then transferred *via* syringe (previously removing the argon from the syringe) into Chamber 2 of the Two-Chamber Glassware (*note*: this chamber is under *vacuum*). Stopcock between Chamber 1 (where [^{13}C]CO is previously produced by photoreduction following **GP0, page S40**) and Chamber 2 is then open. The solution is stirred for 48 h at room temperature under [^{13}C]CO atmosphere. The [^{13}C]CO is then evacuated from the Two-Chamber Glassware, the solution transferred into a round bottom flask and the solvent removed under reduced pressure. The crude is purified by chromatography on silica gel to give the pure compound.

Yields are calculated on the [^{13}C]CO$_2$ loaded in Chamber 1 (limiting reagent, 0.365 mmol).

General Procedure for the Alkoxycarbonylation (GP2):[15]

Glassware Type 1 (Figure S1) was used unless otherwise specified. Aryl halide (0.3 mmol), Pd(dba)$_2$ (8.7 mg, 0.015 mmol, 5 mol%), CataCXium A (10.7 mg, 0.030 mmol, 10 mol%), NaHCO$_3$ (75.6 mg, 0.9 mmol), DMAP (9.1 mg, 0.075 mmol) and alcohol (0.6 mmol) are loaded into Chamber 2 under argon and toluene (1.5 mL) is injected through the septum. Stopcock between Chamber 1 (where [^{13}C]CO is previously produced by photoreduction following **GP0, page S40**)* and Chamber 2 is then open. The solution is stirred for 20 h at 80 °C under [^{13}C]CO atmosphere. The [^{13}C]CO is then evacuated from the Two-Chamber Glassware, the solution transferred into a round bottom flask and the solvent removed under reduced pressure. The crude is purified by chromatography on silica gel to give the pure compound. *1.2 equivalent of [^{13}C]CO$_2$ (0,365 mmol) are loaded in Chamber 1.

General Procedure for the Carbonylative Suzuki Coupling (GP3):[16]

Glassware Type 2 (Figure S2) was used unless otherwise specified. Aryl halide (0.25 mmol), $PdCl_2$ (1.33 mg, 0.0075 mmol, 3 mol%), PPh_3 (3.93 mg, 0.015 mmol, 6 mol%), K_2CO_3 (103.5 mg, 0.75 mmol), aryl boronic acid (0.375 mmol) are loaded into chamber 2 under argon and anisole (2.5 mL) is injected through the septum. Stopcock between Chamber 1 (where [^{13}C]CO is previously produced by photoreduction following **GP0, page S40**)* and Chamber 2 is then open. The solution is stirred for 18 h at 80 °C under [^{13}C]CO atmosphere. The [^{13}C]CO is then evacuated from the Two-Chamber Glassware, the solution transferred into a round bottom flask and the solvent removed under reduced pressure. The crude is purified by chromatography on silica gel to give the pure compound.

*1.8 equivalent of [^{13}C]CO_2 (0,445 mmol) are loaded in Chamber 1.

General Procedure for the Reductive Carbonylation (GP4a):[17]

Glassware Type 1 (Figure S1) was used unless otherwise specified. Aryl halide (0.2 mmol), $Pd(dba)_2$ (5.7 mg, 0.01 mmol, 5 mol%), PCy_3HBF_4 (3.68 mg, 0.01 mmol), potassium formate (33.6 mg, 0.4 mmol), TBAI (22 mg, 0.06 mmol) are loaded into Chamber 2 under argon and acetonitrile (1.5 mL) is injected through the septum. Stopcock between Chamber 1 (where [^{13}C]CO is previously produced by photoreduction following **GP0, page S40**)* and Chamber 2 is then open. The solution is stirred for 18 h at 80 °C under [^{13}C]CO atmosphere. The [^{13}C]CO is then evacuated from the Two-Chamber Glassware, the solution transferred into a round bottom flask and the solvent removed under reduced pressure. The crude is purified by chromatography on silica gel to give the pure compound.

*1.8 equivalent of [^{13}C]CO_2 (0.365 mmol) are loaded in Chamber 1.

General Procedure for the Reductive Carbonylation (GP4b):[17]

Glassware Type 1 (Figure S1) was used unless otherwise specified. Aryl halide (0.1 mmol), $Pd(dba)_2$ (2.9 mg, 0.005 mmol, 5 mol%), PCy_3HBF_4 (1.9 mg, 0.005 mmol, 5 mol%), potassium formate (17 mg, 0.2 mmol), TBAI (11 mg, 0.03 mmol) are loaded into Chamber 2 under argon and acetonitrile (1.5 mL)

is injected through the septum. Stopcock between Chamber 1 (where [^{13}C]CO is previously produced by photoreduction following **GP0, page S40**)* and Chamber 2 is then open. The solution is stirred for 18 h at 80 °C under [^{13}C]CO atmosphere. The [^{13}C]CO is then evacuated from the Two-Chamber Glassware, the solution transferred into a round bottom flask and the solvent removed under reduced pressure. The crude is purified by chromatography on silica gel to give the pure compound.

*3.6 equivalent of [^{13}C]CO_2 (0.365 mmol) are loaded in Chamber 1.

General Procedure for the Reductive Carbonylation (GP4c):[17]

Glassware Type 1 (Figure S1) was used unless otherwise specified. Aryl halide (0.6 mmol), $Pd(dba)_2$ (17 mg, 0.03 mmol, 5 mol%), PCy_3HBF_4 (11 mg, 0.03 mmol, 5 mol%), potassium formate (101 mg, 1.2 mmol), TBAI (66 mg, 0.18 mmol) are loaded into chamber 2 under argon and acetonitrile (3.5 mL) is injected through the septum. Stopcock between Chamber 1 (where [^{13}C]CO is previously produced by photoreduction following **GP0, page S40**) and Chamber 2 is then open. The solution is stirred for 18 h at 80 °C under [^{13}C]CO atmosphere. The [^{13}C]CO is then evacuated from the Two-Chamber Glassware, the solution transferred into a round bottom flask and the solvent removed under reduced pressure. The crude is purified by chromatography on silica gel to give the pure compound.

The yield is calculated on the [^{13}C]CO_2 used (limiting reagent, 0.365 mmol).

General Procedure for the Carbonylative Sonogashira Coupling (GP5):[18]

Glassware Type 1 (Figure S1) was used unless otherwise specified. Aryl halide (0.2 mmol), $PdCl_2$ (1.77 mg, 0.01 mmol, 5 mol%), XantPhos (5.79 mg, 0.01 mmol, 5 mol%), base (0.6 mmol), and corresponding terminal alkyne (0.3 mmol) are loaded into Chamber 2 under argon and dioxane (1 mL) is injected through the septum. Stopcock between Chamber 1 (where [^{13}C]CO is previously produced by photoreduction following **GP0, page S40**) and Chamber 2 is then open. The solution is stirred for 16 h at 75 °C under [^{13}C]CO atmosphere. The [^{13}C]CO is then evacuated from the Two-Chamber Glassware, the solution transferred into a round bottom flask and the solvent removed under reduced pressure. The crude is purified by chromatography on silica gel to give the pure compound.

*1.8 equivalent of [^{13}C]CO_2 (0.365 mmol) are loaded in Chamber 1.

General Procedure for the Carbon Isotope Exchange (GP6):[19]

Glassware Type 1 (Figure S1) was used unless otherwise specified. A solution of acyl Chloride (0.2 mmol), $Pd_2(dba)_3$ (9.1 mg, 0.01 mmol, 5 mol%), P(*o*-tol)$_3$ (12.2 mg, 0.04 mmol, 20 mol%), in dry toluene (1 mL) is transferred *via* syringe (previously removing the argon from the syringe) into Chamber 2 of the Two Chamber Glassware (*note*: this chamber is under *vacuum*). Stopcock between Chamber 1 (where [^{13}C]CO is previously produced by photoreduction following **GP0, page S40**)* and Chamber 2 is then open. The solution is stirred for 18 h at 75 °C under [^{13}C]CO atmosphere. The [^{13}C]CO is then evacuated from the Two-Chamber Glassware and the reaction is quenched with NaOH 1M. The basic aqueous phase is acidified with HCl 1M and extracted with EtOAc to give the pure acid.

*1.8 equivalent of [^{13}C]CO_2 (0.365 mmol) are loaded in Chamber 1.

Aminocarbonylation

[^{13}C] N-hexyl-4-methoxybenzamide ([^{13}C]1)

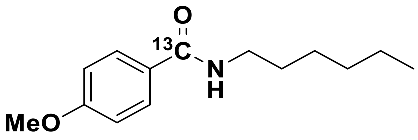

$C_{13}{}^{13}CH_{21}NO_2$
MW: 236.32 g.mol^{-1}
Yield: 82%
Light brown solid

The [^{13}C] *N*-hexyl-4-methoxybenzamide (**[^{13}C]1**) was prepared accordingly to the **GP1**, using Pd(dba)$_2$ (8.7 mg, 0.015 mmol, 2 mol%), P(*t*Bu)$_3$HBF$_4$ (8.7 mg, 0.030 mmol, 4 mol%), 4-iodoanisole (169 mg, 0.724 mmol), *n*-hexylamine (0.19 mL, 1.45 mmol), and DABCO (162 mg, 1.45 mmol) in dry THF (3.0 mL) under argon. The solution is injected *via* syringe in chamber 2. The reaction mixture was stirred at room temperature for 48 hours then the solvent was evaporated under *vacuum*. The crude was purified by FC on SiO_2 gel (Eluent Hept : EtOAc 80:20), providing the compound (**[^{13}C]1**) as light brown solid (69 mg, 82% yield, [^{13}C]CO_2 = 0.359 mmol); mp 63-65 °C.

^{1}H NMR (400 MHz, CDCl$_3$) δ 7.74 – 7.70 (m, 2H), 6.93 – 6.90 (m, 2H), 6.06 (br.s, 1H), 3.84 (s, 3H), 3.45 – 3.39 (m, 2H), 1.61 – 1.58 (m, 2H), 1.38 – 1.29 (m, 6H), 0.89 (t, *J* = 7.0 Hz, 3H).
^{13}C NMR (101 MHz, CDCl$_3$) δ 167.1 (^{13}C labeled), 162.1, 128.7 (d, *J* = 2.7 Hz, 2C), 127.2 (d, *J* = 66.0 Hz), 113.7 (d, *J* = 4.5 Hz, 2C), 55.5, 40.1, 31.6, 29.8, 26.8, 22.7, 14.1.
HRMS (ESI) *m/z* calcd for $C_{13}{}^{13}CH_{21}NO_2$ $[M+H]^+$: 237.1677; found: 237.1678.
IR (cm^{-1}): 3326, 2957, 2937, 2914, 2844, 1599, 1570, 1522, 1498, 1462, 1302, 1246, 1181, 1105, 1029, 843, 752, 609.

[^{13}C] N-benzylnicotinamide ([^{13}C]2)

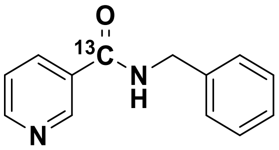

$C_{12}{}^{13}CH_{12}N_2O$
MW: 213.24 g.mol^{-1}
Yield: 57%
Pale yellow solid

The [^{13}C] *N*-benzylnicotinamide (**[^{13}C]2**) was prepared accordingly to the **GP1**, using Pd(dba)$_2$ (8.7 mg, 0.015 mmol, 2 mol%), P(*t*Bu)$_3$HBF$_4$ (8.7 mg, 0.030 mmol, 4 mol%), 3-iodopyridine (148 mg, 0.724 mmol), benzylamine (0.16 mL, 1.45 mmol), and DABCO (162 mg, 1.45 mmol) in dry THF (3.0 mL) under argon. The solution is injected *via* syringe in Chamber 2. The reaction mixture was stirred at room temperature for 48 hours then the solvent was evaporated under *vacuum*. The crude was purified by FC on SiO_2 gel (Eluent DCM : MeOH 95 : 5), providing the compound (**[^{13}C]2**) as pale yellow solid (44 mg, 57% yield, [^{13}C]CO_2 = 0.362 mmol); mp 74-77 °C.

^{1}H NMR (400 MHz, $CDCl_3$) δ 8.95 (m, 1H), 8.68 (dd, J = 4.7, 1.1 Hz, 1H), 8.13 (m, 1H), 7.41 – 7.27 (m, 6H), 6.69 (br.s, 1H), 4.65 (dd, J = 5.6, 3.2 Hz, 2H).
^{13}C NMR (100 MHz, $CDCl_3$) δ 165.6 (^{13}C labeled), 152.2, 148.0 (d, J = 3.3 Hz), 137.9 (d, J = 1.4 Hz), 135.4 (d, J = 1.7 Hz), 130.2 (d, J = 65.4 Hz), 128.9 (2C), 128.0 (2C), 127.8, 123.6 (d, J = 3.2 Hz), 44.2.
HRMS (ESI) *m/z* calcd for $C_{12}{}^{13}CH_{12}N_2O$ $[M+H]^+$: 214.1057; found: 214.1055.
IR (cm^{-1}): 3281, 3030, 2920, 1600, 1590, 1530, 1282, 821, 736, 696, 602, 523, 458.

[^{13}C] 1-(4-(morpholine-4-carbonyl)phenyl)ethan-1-one ([^{13}C]3)

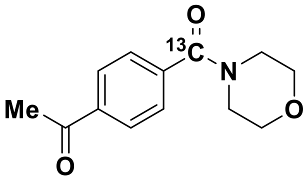

$C_{12}{}^{13}CH_{15}NO_3$
MW: 234.26 g.mol^{-1}
Yield: 72%
White solid

The [^{13}C] 1-(4-(morpholine-4-carbonyl)phenyl)ethan-1-one (**[^{13}C]3**) was prepared accordingly to the **GP1**, using Pd(dba)$_2$ (8.7 mg, 0.015 mmol, 2 mol%), P(*t*Bu)$_3$HBF$_4$ (8.7 mg, 0.030 mmol, 4 mol%), 4-iodoacetophenone (178 mg, 0.724 mmol), morpholine (0.13 mL, 1.45 mmol), and DABCO (162 mg, 1.45 mmol) in dry THF (3.0 mL) under argon. The solution is injected *via* syringe in Chamber 2. The reaction mixture was stirred at room temperature for 48 hours then the solvent was evaporated under *vacuum*. The crude was purified by FC on SiO_2 gel (Eluent EtOAc : MeOH 98:2), providing the compound (**[^{13}C]3**) as white solid (61 mg, 72% yield, [^{13}C]CO_2 = 0.365 mmol); mp 121-123 °C.

^{1}H NMR (400 MHz, $CDCl_3$) δ 8.01 - 7.98 (m, 2H), 7.51 - 7.47 (m, 2H), 3.78 (br.s, 4H), 3.61 (br.s, 2H), 3.39 (br.s, 2H), 2.61 (s, 3H).
^{13}C NMR (100 MHz, $CDCl_3$) δ 197.3, 169.3 (^{13}C labeled), 139.7 (d, J = 66.1 Hz), 137.9, 128.6 (d, J = 4.3 Hz, 2C), 127.3 (d, J = 2.1 Hz, 2C), 66.8 (2C), 48.1, 42.5, 26.8.
HRMS (ESI) *m/z* calcd for $C_{12}{}^{13}CH_{15}NO_3$ [M+H]$^+$: 235.1159; found: 235.1158.
IR (cm^{-1}): 2919, 1680, 1592, 1413, 1401, 1357, 1272, 1250, 1112, 1009, 832, 583, 540.

[^{13}C] *N*-cyclohexyl-4-fluorobenzamide ([^{13}C]4)

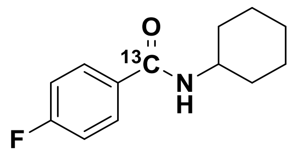

$C_{12}{}^{13}CH_{16}FNO$
MW: 222.27 g.mol^{-1}
Yield: 32%
Pale yellow solid

The [^{13}C] *N*-cyclohexyl-4-fluorobenzamide (**[^{13}C]4**) was prepared accordingly to the **GP1**, using Pd(dba)$_2$ (8.7 mg, 0.015 mmol, 2 mol%), P(*t*Bu)$_3$HBF$_4$ (8.7 mg, 0.030 mmol, 4 mol%), 4-iodofluorobenzene (161 mg, 0.724 mmol), cyclohexylamine (0.16 mL, 1.45 mmol), and DABCO (162 mg, 1.45 mmol) in dry THF (3.0 mL) under argon. The solution is injected *via* syringe in Chamber 2. The reaction mixture was stirred at room temperature for 48 hours then the solvent was evaporated under *vacuum*. The crude was purified by FC on SiO_2 gel (Eluent Hept : EtOAc 85:15), providing the compound (**[^{13}C]4**) as pale yellow solid (26 mg, 32% yield, [^{13}C]CO_2 = 0.365 mmol); mp 142-144 °C.

^{1}H NMR (400 MHz, $CDCl_3$) δ 7.78 – 7.73 (m, 2H), 7.10 – 7.06 (m, 2H), 5.97 (br.s, 1H), 3.99 – 3.90 (m, 1H), 2.03 – 1.99 (m, 2H), 1.77 – 1.72 (m, 2H), 1.47 – 1.34 (m, 2H), 1.27 – 1.13 (m, 4H).
^{13}C NMR (100 MHz, $CDCl_3$) δ 165.7 (^{13}C labeled), 161.42 (d, *J* = 298.6 Hz), 131.3 (dd, *J* = 65.3, 3.1 Hz), 129.2 (dd, *J* = 8.8, 2.6 Hz, 2C), 115.6 (dd, *J* = 21.8, 4.5 Hz, 2C), 48.9, 33.4 (2C), 25.7, 25.0 (2C).
^{19}F NMR (376 MHz, $CDCl_3$) δ -108.7.
HRMS (ESI) *m/z* calcd for $C_{12}{}^{13}CH_{16}FNO$ $[M+H]^+$: 223.1324; found: 223.1322.
IR (cm^{-1}): 3334, 2921, 2851, 1602, 1575, 1521, 1497, 1318, 1228, 1158, 851, 756, 651, 624, 579.

[^{13}C] N-(adamantan-1-yl)-2-(trifluoromethyl)benzamide ([^{13}C]5)

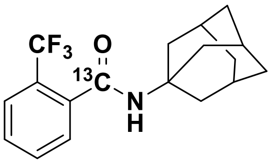

$C_{17}{}^{13}CH_{20}F_3NO$
MW: 324.35 g.mol^{-1}
Yield: 53%
White solid

The [^{13}C] *N*-(adamantan-1-yl)-2-(trifluoromethyl)benzamide (**[^{13}C]5**) was prepared accordingly to the **GP1**, using Pd(dba)$_2$ (8.7 mg, 0.015 mmol, 2 mol%), P(*t*Bu)$_3$HBF$_4$ (8.7 mg, 0.030 mmol, 4 mol%), 2-iodobenzotrifluoride (197 mg, 0.724 mmol), 1-adamanthylamine (219 mg, 1.45 mmol), and DABCO (162 mg, 1.45 mmol) in dry THF (3.0 mL) under argon. The solution is injected *via* syringe in Chamber 2. The reaction mixture was stirred at room temperature for 48 hours then the solvent was evaporated under *vacuum*. The crude was purified by FC on SiO_2 gel (Eluent Hept : EtOAc 90 : 10), providing the compound (**[^{13}C]5**) as white solid (63 mg, 53% yield, [^{13}C]CO_2 = 0.366 mmol); mp 196-198 °C.

^{1}H NMR (400 MHz, CDCl$_3$) δ 7.65 (d, *J* = 7.7 Hz, 1H), 7.58 – 7.47 (m, 3H), 5.40 (s, 1H), 2.11 (br. s, 9H), 1.75 – 1.68 (m, 6H).
^{13}C NMR (100 MHz, CDCl$_3$) δ 166.9 (^{13}C labeled), 137.2 (dd, *J* = 62.9, 2.1 Hz), 132.1 (d, *J* = 3.0 Hz), 129.5, 128.7 (d, *J* = 1.3 Hz), 127.0 (q, *J* = 31.7 Hz), 126.5 – 126.1 (m), 123.8 (q, *J* = 273.7 Hz), 53.1, 41.4 (3C), 36.4 (3C), 29.6 (3C).
^{19}F NMR (376 MHz, CDCl$_3$) δ -58.5.
HRMS (ESI) *m/z* calcd for $C_{17}{}^{13}CH_{20}F_3NO$ $[M+H]^+$: 325.1603; found: 325.1603.
IR (cm^{-1}): 3269, 2905, 2847, 1597, 1535, 1311, 1118, 1034, 764, 687, 606.

[^{13}C] (1H-indol-5-yl)(4-methylpiperidin-1-yl)methanone ([^{13}C]6)

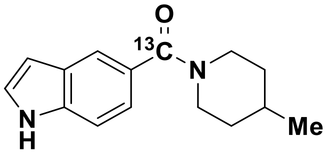

$C_{14}{}^{13}CH_{18}N_2O$
MW: 243.31 g.mol^{-1}
Yield: 53%
Light brown solid

The [^{13}C] (1H-indol-5-yl)(4-methylpiperidin-1-yl)methanone (**[^{13}C]6**) was prepared accordingly to the **GP1** (**Glassware Type 3** (Figure S3) was used), using Pd(dba)$_2$ (9.5 mg, 0.016 mmol, 2 mol%), P(*t*Bu)$_3$HBF$_4$ (9.5 mg, 0.032 mmol, 4 mol%), 5-iodoindole (193 mg, 0.796 mmol), 4-methylpiperidine (0.19 mL, 1.59 mmol), and DABCO (178 mg, 1.59 mmol) in dry THF (3.3 mL) under argon. The solution is injected *via* syringe in Chamber 2. The reaction mixture was stirred at room temperature for 48 hours then the solvent was evaporated under *vacuum*. The crude was purified by FC on SiO$_2$ gel (Eluent Hept : EtOAc 60 :40), providing the compound (**[^{13}C]6**) as light brown solid (51 mg, 53% yield, [^{13}C]CO$_2$ = 0.400 mmol); mp 143-145 °C.

The [^{13}C] (1H-indol-5-yl)(4-methylpiperidin-1-yl)methanone (**[^{13}C]6**) was also prepared using 0.6 mmol of [^{13}C]CO following the **GP0bis** in chamber 1 and **GP3** in chamber 2. The crude was purified by FC on SiO$_2$ gel (Eluent Hept : EtOAc from 95:5 to 80:20), providing the compound (**[^{13}C]6**) as light brown solid (95 mg, 65% yield, [^{13}C]CO$_2$ = 0.600 mmol).

^{1}H NMR (400 MHz, CDCl$_3$) δ 9.54 (s, 1H), 7.69 (m, 1H), 7.21 – 7.14 (m, 2H), 7.12 – 7.08 (m, 1H), 6.51 – 6.46 (m, 1H), 4.71 (br.s, 1H), 3.91 (br. s, 1H), 2.89 (s, 2H), 1.82 – 1.45 (m, 3H), 1.20 (br. s, 2H), 0.97 (d, *J* = 6.4 Hz, 3H).
^{13}C NMR (100 MHz, CDCl$_3$) δ 172.3 (^{13}C labeled), 136.6, 127.3 (d, *J* = 4.9 Hz), 127.3 (d, *J* = 66.9 Hz), 125.9, 120.9 (d, *J* = 2.6 Hz), 119.7 (d, *J* = 2.3 Hz), 111.4 (d, *J* = 4.5 Hz), 102.5, 48.6 (br. s), 42.9 (br. s), 34.3 (br. d, 2C), 31.3, 21.9.
HRMS (ESI) *m/z* calcd for $C_{14}{}^{13}CH_{18}N_2O$ [M+H]$^+$: 244.1529; found: 244.1525.
IR (cm^{-1}): 3141, 2955, 2906, 1551, 1434, 1311, 1240, 1085. 966, 895, 812, 775, 742, 577.

Alkoxycarbonylation

[13C] adamantan-1-ylmethyl 4-butoxybenzoate ([13C]7)

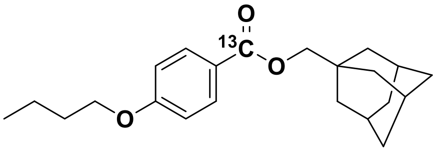

$C_{21}{}^{13}CH_{30}O_3$
MW: 343.47 g.mol^{-1}
Yield: 28%
Yellow oil

The [^{13}C] adamantan-1-ylmethyl 4-butoxybenzoate (**[^{13}C]7**) was prepared accordingly to the **GP2**, using Pd(dba)$_2$ (8.6 mg, 0.015 mmol, 5 mol%), cataCXium® A (10.7 mg, 0.030 mmol, 10 mol%), DMAP (9.1 mg, 0.075 mmol,), $NaHCO_3$ (75.6 mg, 0.900 mmol), 1-bromo-4-butoxybenzene (0.051 mL, 0.3 mmol), 1-adamantanemethanol (0.079 mL, 0.6 mmol) under argon. The reaction mixture was heated to 80 °C for 20 hours then the solvent was evaporated under *vacuum*. The crude was purified by FC on SiO_2 gel (Eluent Hept : EtOAc from 20/0 to 18/2, Rf: 0.4), providing the compound (**[^{13}C]7**) as a yellow oil (29 mg, 28% yield).

^{1}H NMR (400 MHz, CDCl$_3$) δ 8.01 – 7.98 (m, 2H), 6.91 (d, J = 8.6 Hz, 2H), 4.02 (t, J = 6.5 Hz, 2H), 3.89 (d, J = 2.4 Hz, 2H), 2.01 (m, 2H), 1.81-1.67 (m, 9H), 1.63 (d, J = 2.4 Hz, 6H), 1.50 (dd, J = 15.0, 7.5 Hz, 2H), 0.98 (t, J = 7.4 Hz, 3H).

^{13}C NMR (101 MHz, CDCl$_3$) δ 166.7 (^{13}C labeled), 163.0 (d, J = 0.6 Hz), 131.6 (d, J = 2.7 Hz, 2C), 122.9 (d, J = 77.1 Hz), 114.2 (d, J = 4.8 Hz, 2C), 74.3 (d, J = 2.4 Hz), 68.0, 39.6 (3C), 37.1 (3C), 33.7 (d, J = 2.1 Hz), 31.3, 28.2 (3C), 19.3, 14.0.

HRMS (ESI) *m/z* calcd for $^{13}CC_{21}H_{30}O_3$ $[M+H]^+$: 366.2122; found: 366.2120; $^{13}CC_{21}H_{30}O_3$ [M-[adamantine-CH$_2$]+H]$^+$ calcd : 149.1325; found 149.1324.

IR (cm^{-1}): 2904, 1672, 1606, 1248, 1165, 1096.

[^{13}C] 2-(piperidin-1-yl)ethyl 2,3-dihydrobenzo[b][1,4]dioxine-6-carboxylate ([^{13}C]8)

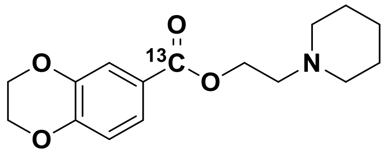

$C_{15}{}^{13}CH_{21}NO_4$
MW: 292.35 g.mol^{-1}
Yield: 35%
Yellow oil

The [^{13}C] 2-(piperidin-1-yl)ethyl 2,3-dihydrobenzo[b][1,4]dioxine-6-carboxylate (**[^{13}C]8**) was prepared accordingly to the **GP2**, using Pd(dba)$_2$ (8.6 mg, 0.015 mmol, 5 mol%), cataCXium® A (10.7 mg, 0.030 mmol, 10 mol%), DMAP (9.1 mg, 0.075 mmol), $NaHCO_3$ (75.6 mg, 0.9 mmol), 4-bromo-1,2-(ethylenedioxy)benzene (0.040 mL, 0.300 mmol), 1-(2-hydroxyethyl)piperidine (0.079 mL, 0.6 mmol) under argon. The reaction mixture was heated to 80 °C for 20 hours then the solvent was evaporated under *vacuum*. The crude light brown oil was purified by FC on SiO_2 gel (Eluent EtOAc : MeOH 95/5, Rf: 0.3), providing the compound (**[^{13}C]8**) as a yellow oil (31 mg, 35% yield).

^{1}H NMR (400 MHz, CDCl$_3$) δ 7.55 – 7.53 (m, 2H), 6.87 (dd, J = 9.0, 1.1 Hz, 1H), 4.41 (td, J = 6.1, 3.0 Hz, 2H), 4.32 – 4.28 (m, 2H), 4.27 – 4.23 (m, 2H), 2.74 (t, J = 6.1 Hz, 2H), 2.53 – 2.50 (m, 4H), 1.60 (dt, J = 11.1, 5.6 Hz, 4H), 1.46 – 1.41 (m, 2H).
^{13}C NMR (101 MHz, CDCl$_3$) δ 166.1 (^{13}C labeled), 147.9, 143.2 (d, J = 6.3 Hz), 123.6 (d, J = 76.8 Hz), 123.5 (d, J = 2.5 Hz), 119.1 (d, J = 3.0 Hz), 117.2 (d, J = 5.3 Hz), 64.7, 64.2, 62.7, 57.4, 54.9 (2C), 26.0 (2C), 24.2.
LC-MS (ESI) m/z $C_{15}{}^{13}CH_{21}NO_4$ $[M+H]^+$ 293.5.
IR (cm^{-1}): 2933, 1671, 1585, 1505, 1430, 1289, 1218, 1184, 1064, 1096, 887, 749.

[^{13}C] 2-(benzofuran-3-yl)ethyl 4-cyanobenzoate ([^{13}C]9)

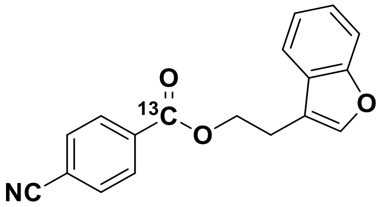

$C_{17}{}^{13}CH_{13}NO_3$
MW: 292.31 g.mol^{-1}
Yield: 27%
White solid

The [^{13}C] 2-(benzofuran-3-yl)ethyl 4-cyanobenzoate (**[^{13}C]9**) was prepared accordingly to the **GP2**, using $Pd(dba)_2$ (8.6 mg, 0.015 mmol, 5 mol%), cataCXium® A (10.7 mg, 0.030 mmol, 10 mol%), DMAP (9.1 mg, 0.075 mmol), $NaHCO_3$ (75.6 mg, 0.9 mmol), 4-Bromobenzonitrile (54.6 mg, 0.3 mmol), 2-benzo[b]furan-3-ylethanol (0.081 mL, 0.600 mmol) under argon. The reaction mixture was heated to 80 °C for 20 hours then the solvent was evaporated under *vacuum*. The crude light brown oil was purified by FC on SiO_2 gel (Eluent Hept : EtOAc 8/2, Rf: 0.32), providing the compound (**[^{13}C]9**) as white solid (23.3 mg, 27% yield).

^{1}H NMR (400 MHz, $CDCl_3$) δ 8.12 – 8.078 (m, 2H), 7.75 – 7.71 (m, 2H), 7.61 (ddd, J = 7.6, 1.4, 0.7 Hz, 1H), 7.54 (m, 1H), 7.49 (dt, J = 8.2, 0.9 Hz, 1H), 7.32 (ddd, J = 8.2, 7.2, 1.5 Hz, 1H), 7.28 – 7.24 (m, 1H), 4.66 (td, J = 6.8, 3.0 Hz, 2H), 3.18 (td, J = 6.8, 1.0 Hz, 2H).
^{13}C NMR (101 MHz, $CDCl_3$) δ 165.0 (^{13}C labeled), 155.4, 142.1, 132.38 (d, J = 4.7 Hz, 2C), 130.3 (d, J = 2.4 Hz, 2C), 127.9, 124.7 (2C), 122.7 (2C), 119.5 (2C), 116.3, 111.8, 64.8 (d, J = 2.4 Hz), 23.4 (d, J = 2.0 Hz).
LC-MS (ESI) m/z $^{13}CC_{17}H_{13}NO_3$ [M-[$^{13}CC_{10}H_9O_3$]+Na]+H]$^+$ 124.1
IR (cm^{-1}): 2961, 2920, 1681, 1453, 1255, 1176, 1097, 857, 748, 683.
HRMS (ESI) ***m/z*** calcd for $^{13}CC_{17}H_{13}NO_3$ [M-[$C_{10}H_9O$]+H]$^+$: 145.0649, found 145.0647.

[^{13}C]-2-(piperidin-1-yl)ethyl 4-cyanobenzoate ([^{13}C]10)

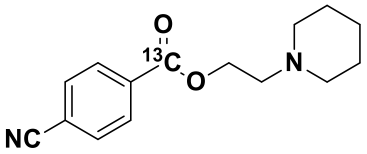

$C_{14}{}^{13}CH_{18}N_2O_2$
MW: 259.31 g.mol^{-1}
Yield: 82%
Yellow solid

The [^{13}C] 2-(piperidin-1-yl)ethyl 4-cyanobenzoate (**[^{13}C]10**) was prepared accordingly to the GP2, using Pd(dba)$_2$ (8.6 mg, 0.015 mmol, 5 mol%), cataCXium® A (10.7 mg, 0.03 mmol, 10 mol%), DMAP (9.1 mg, 0.075 mmol), $NaHCO_3$ (75.6 mg, 0.9 mmol), 4-iodobenzonitrile (68.7 mg, 0.3 mmol), 1-(2-hydroxyethyl)piperidine (0.079 mL, 0.6 mmol) under argon. The reaction mixture was heated to 80 °C for 20 hours then the solvent was evaporated under *vacuum*. The crude was purified by FC on SiO_2 gel (Eluent Hept : EtOAc 8/2), providing the compound (**[^{13}C]10**) as yellow solid (53.1 mg, 69% yield), m.p. 119-121 °C.

^{1}H NMR (400 MHz, $CDCl_3$) δ 8.13 – 8.06 (m, 2H), 7.77 – 7.66 (m, 2H), 4.44 (td, J = 6.1, 3.0 Hz, 2H), 2.72 (t, J = 6.1 Hz, 2H), 2.55 – 2.36 (m, 4H), 1.60 – 1.50 (m, 4H), 1.48 – 1.34 (m, 2H).
^{13}C NMR (100 MHz, $CDCl_3$) δ 164.9 (^{13}C labeled), 134.2 (d, J = 74.8 Hz), 132.3 (d, J = 4.6 Hz, 2C), 130.2 (d, J = 2.4 Hz, 2C), 118.0, 116.4, 63.6 (d, J = 2.3 Hz), 57.3 (d, J = 2.1 Hz), 54.9 (2C), 26.0 (2C), 24.2.
IR (cm^{-1}): 2934, 2226, 1679, 1248, 1089, 1016, 853, 770, 750, 684, 677, 568.
LCMS (ESI) *m/z* $C_{14}{}^{13}CH_{18}N_2O_2$ [M+H]$^+$ 260.3.
HRMS (ESI) *m/z* calcd for $C_{14}{}^{13}CH_{18}N_2O_2$ [M+H]$^+$: 260.1480, found: 260.1479.

[^{13}C] thiophen-2-ylmethyl 1H-indole-5-carboxylate ([^{13}C]11)

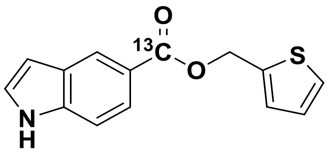

$C_{13}{}^{13}CH_{11}NO_2S$
MW: 258.31 g.mol^{-1}
Yield: 83%
Yellow oil

The [^{13}C] thiophen-2-ylmethyl 1H-indole-5-carboxylate (**[^{13}C]11**) was prepared accordingly to the **GP2**, using Pd(dba)$_2$ (8.6 mg, 0.015 mmol, 5 mol%), cataCXium® A (10.7 mg, 0.030 mmol, 10 mol%), DMAP (9.1 mg, 0.075 mmol), $NaHCO_3$ (75.6 mg, 0.9 mmol), 5-Iodoindole (73 mg, 0.3 mmol), 2-thiophenemethanol (0.057 mL, 0.6 mmol) under argon. The reaction mixture was heated to 80 °C for 20 hours then the solvent was evaporated under *vacuum*. The crude light brown oil was purified by FC on SiO_2 gel (Eluent EtOAc : MeOH 95/5, Rf: 0.25), the compound (**[^{13}C]11**) as a yellow oil (64.5 mg, 83% yield).

^{1}H NMR (400 MHz, $CDCl_3$) δ 8.44 (ddt, *J* = 4.2, 1.5, 0.7 Hz, 1H), 8.40 (s, 1H), 7.93 (ddd, *J* = 8.7, 3.7, 1.6 Hz, 1H), 7.39 (dd, *J* = 8.6, 0.9 Hz, 1H), 7.34 (dd, *J* = 5.1, 1.2 Hz, 1H), 7.26 (m, 1H), 7.19 (ddt, *J* = 3.5, 1.4, 0.8 Hz, 1H), 7.02 (dd, *J* = 5.1, 3.5 Hz, 1H), 6.64 (ddd, *J* = 3.1, 2.0, 1.0 Hz, 1H), 5.54 (dd, *J* = 3.2, 0.8 Hz, 2H).

^{13}C NMR (101 MHz, $CDCl_3$) δ 167.5 (^{13}C labeled), 138.7 (d, *J* = 2.2 Hz), 138.6, 128.0, 127.6 (d, *J* = 5.1 Hz), 126.9, 126.8, 125.71 (t, *J* = 6.1 Hz), 124.2 (d, *J* = 2.6 Hz), 123.7 (d, *J* = 3.1 Hz), 121.7 (d, *J* = 75.9 Hz), 110.9 (d, *J* = 4.9 Hz), 104.2, 60.9 (d, *J* = 2.3 Hz).

LC-MS (ESI) m/z $C_{13}{}^{13}CH_{11}NO_2S$ $[M+H]^+$ 259.2.

IR (cm^{-1}): 3418, 3337, 1649, 1614, 1293, 1240, 1171, 1094, 1074, 745, 703.

HRMS (ESI) *m/z* $C_{13}{}^{13}CH_{11}NO_2S$ $[M+H]^+$ calcd 259.0619, found 259.0616.

[^{13}C] (1-benzylpyrrolidin-2-yl)methyl 3-(4-(trifluoromethyl)benzoyl)benzoate ([^{13}C]12)

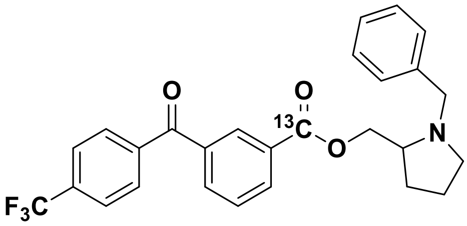

$C_{26}{}^{13}CH_{24}F_3NO_3$
MW: 468.48 g.mol^{-1}
Yield: 64%
Yellow oil

The [^{13}C] (1-benzylpyrrolidin-2-yl)methyl 3-(4-(trifluoromethyl)benzoyl)benzoate (**[^{13}C]12**) was prepared accordingly to the **GP2**, using $Pd(dba)_2$ (8.6 mg, 0.015 mmol, 5 mol%), cataCXium® A (10.7 mg, 0.030 mmol, 10 mol%), DMAP (9.1 mg, 0.075 mmol), $NaHCO_3$ (75.6 mg, 0.9 mmol,), 3-(iodophenyl)(4-(trifluoromethyl)phenyl)methanone (112.8 mg, 0.3 mmol), 1-benzylpyrrolidin-3-yl-methanol (0.600 mmol, 114.8 mg) under argon. The reaction mixture was heated to 80 °C for 20 hours then the solvent was evaporated under *vacuum*. The crude light brown oil was purified by FC on SiO_2 gel (Eluent Hept : EtOAc 8/2, Rf: 0.3), providing the compound (**[^{13}C]12**) as a yellow oil (90.0 mg, 64% yield).

^{1}H NMR (400 MHz, CDCl$_3$) δ 8.19 – 8.15 (m, 2H), 7.90 (d, J = 8.1 Hz, 2H), 7.84 (d, J = 7.7 Hz, 2H), 7.77 (d, J = 8.2 Hz, 2H), 7.36 – 7.21 (m, 5H), 4.37 (ddd, J = 5.7, 2.8, 1.2 Hz, 2H), 4.16 (d, J = 13.1 Hz, 1H), 3.49 (d, J = 13.1 Hz, 1H), 3.05 – 2.92 (m, 2H), 2.34 – 2.28 (m, 1H), 2.09 – 2.02 (m, 1H), 1.81 – 1.74 (m, 3H).

^{13}C NMR (101 MHz, CDCl$_3$) δ 194.9, 165.6 (^{13}C labeled), 140.3 (d, J = 0.6 Hz), 140.1 (d, J = 0.9 Hz), 139.6, 134.2 (d, J = 74.6 Hz), 134.3 (q, J = 32.8 Hz), 133.8, 130.3 (2C), 130.0, 129.9, 129.8 (2C), 128.9 (2C), 128.4 (2C), 127.0, 125.6 (CCF_3, q, J = 3.7 Hz), 123.7 (CF_3, q, J = 272.8 Hz), 68.2 (d, J = 2.3 Hz), 62.1 (d, J = 2.2 Hz), 59.7, 54.6, 28.7, 23.2.

^{19}F NMR (376 MHz, CDCl$_3$) δ -63.03.

LC-MS (ESI) m/z $^{13}CC_{26}H_{24}FNO_3$ $[M+H]^+$ 469.7.

IR (cm^{-1}): 2957, 2793, 1668, 1453, 1407, 1323, 1240, 1167, 1126, 1097, 1064, 1016, 929, 868, 851, 779, 724, 700, 668, 466.

[^{13}C] (1-ethyl-1H-imidazol-2-yl)methyl 3,4,5-trimethoxybenzoate ([^{13}C]13)

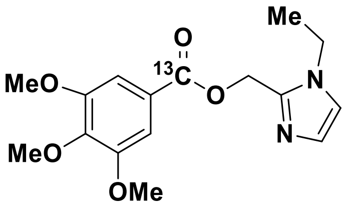

$C_{15}{}^{13}CH_{20}N_2O_5$
MW: 321.34 g.mol^{-1}
Yield: 73%
Yellow oil

The [^{13}C] (1-ethyl-1H-imidazol-2-yl)methyl 3,4,5-trimethoxybenzoate (**[^{13}C]13**) was prepared accordingly to the **GP2**, using Pd(dba)$_2$ (8.6 mg, 0.015 mmol, 5 mol%), cataCXium® A (10.7 mg, 0.030 mmol, 10 mol%), DMAP (9.1 mg, 0.075 mmol), $NaHCO_3$ (75.6 mg, 0.9 mmol,), 5-iodo-1,2,3-trimethoxybenzene (88.2 mg, 0.3 mmol), (1-ethyl-1H-imidazol-2-yl)methanol (75.7 mg, 0.6 mmol) under argon. The reaction mixture was heated to 80 °C for 20 hours then the solvent was evaporated under *vacuum*. The crude light brown oil was purified by FC on SiO_2 gel (Eluent DCM : MeOH : Et_3N 90/10/1, Rf: 0.35), providing the compound (**[^{13}C]13**) as a yellow oil (70 mg, 73% yield).

^{1}H NMR (400 MHz, $CDCl_3$) δ 7.29 (s, 1H), 7.28 (s, 1H), 7.08 (d, J = 1.3 Hz, 1H), 6.99 (d, J = 1.3 Hz, 1H), 5.42 (d, J = 2.7 Hz, 2H), 4.06 (q, J = 7.3 Hz, 2H), 3.89 (s, 3H), 3.87 (s, 6H), 1.42 (t, J = 7.3 Hz, 3H).

^{13}C NMR (101 MHz, $CDCl_3$) δ 165.9 (^{13}C labeled), 153.1 (d, J = 6.6 Hz, 2C), 142.7 (d, J = 1.0 Hz), 142.0 (d, J = 2.6 Hz), 128.8, 124.5 (d, J = 76.4 Hz), 120.4, 107.2 (d, J = 2.8 Hz, 2C), 60.9, 58.3 (d, J = 2.0 Hz), 56.4 (2C), 41.3, 16.7.

LC-MS (ESI) m/z $^{13}CC_{15}H_{20}N_2O_5$ [M+H]$^+$ 322.5.

IR (cm^{-1}): 2938, 1671, 1586, 1502, 1456, 1414, 1353, 1328, 1230, 1203, 1169, 1124, 1104, 1000, 969, 859, 743, 720.

HRMS (ESI) *m/z* calcd for $^{13}CC_{15}H_{20}N_2O_5$ [M+H]$^+$: 322.1482, found 322.1478.

Reductive Carbonylation

[13C] 4-(benzyloxy)benzaldehyde ([13C]14)

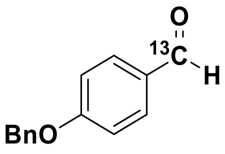

C_{13}13CH$_{12}$O$_2$
MW: 213.24 g.mol-1
Yield: 52%
White solid

The [^{13}C] 4-(benzyloxy)benzaldehyde (**[^{13}C]14**) was prepared accordingly to the **GP4c**, using Pd(dba)$_2$ (17 mg, 0.03 mmol, 5 mol%), PCy$_3$HBF$_4$ (11 mg, 0.03 mmol, 5 mol%), 1-(benzyloxy)-4-iodobenzene (186 mg, 0.6 mmol), potassium formate (101 mg, 1.2 mmol) and TBAI (66 mg, 0.18 mmol) in dry acetonitrile (3.5 mL) under argon. The reaction mixture was stirred 80 °C for 18 hours then the solvent was evaporated under *vacuum*. The crude was purified by FC on SiO$_2$ gel (eluent Hept : EtOAc 90:10), providing the compound (**[^{13}C]14**) as white solid (40 mg, 52% yield, [^{13}C]CO$_2$ = 0.365 mmol as limiting reagent); mp 68-70 °C.

^{1}H NMR (400 MHz, CDCl$_3$) δ 9.89 (d, J = 172.3 Hz, 1H), 7.90 – 7.78 (m, 2H), 7.47 – 7.32 (m, 5H), 7.08 (d, J = 8.6 Hz, 2H), 5.16 (s, 2H).
^{13}C NMR (100 MHz, CDCl$_3$) δ 191.0 (^{13}C labeled), 163.9 (d, J = 0.6 Hz), 136.0, 132.1 (d, J = 4.4 Hz), 130.2 (d, J = 54 Hz), 128.9 (2C), 128.5, 127.6, 115.3 (d, J = 4.9 Hz, 2C), 70.40.
HRMS (ESI) *m/z* calcd for $C_{13}{}^{13}CH_{12}O_2$ $[M+H]^+$: 214.0944; found: 214.0943.
IR (cm^{-1}): 3035, 2920, 2823, 2737, 1650, 1597, 1571, 1508, 1249, 1161, 1118, 829, 734, 695, 651, 514.

[^{13}C] 4-chlorobenzaldehyde ([^{13}C]15)

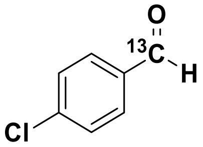

$C_6{}^{13}CH_5ClO$
MW: 141.56 g.mol^{-1}
Yield: 31%
White solid

The [^{13}C] 4-chlorobenzaldehyde (**[^{13}C]15**) was prepared accordingly to the **GP4c**, using Pd(dba)$_2$ (17 mg, 0.03 mmol, 5 mol%), PCy_3HBF_4 (11 mg, 0.03 mmol, 5 mol%), 1-chloro-4-iodobenzene (143 mg, 0.6 mmol), potassium formate (101 mg, 1.2 mmol) and TBAI (66 mg, 0.18 mmol) in dry acetonitrile (3.5 mL) under argon. The reaction mixture was stirred 80 °C for 18 hours then the solvent was evaporated under *vacuum*. The crude was purified by FC on SiO_2 gel (eluent pentane : Et_2O 90:10), providing the compound (**[^{13}C]15**) as white solid (16 mg, 31% yield, [^{13}C]CO_2 = 0.366 mmol as limiting reagent); mp 44-46 °C.

^{1}H NMR (400 MHz, $CDCl_3$) δ 9.98 (d, *J* = 175.3 Hz, 1H), 7.89 – 7.76 (m, 2H), 7.54 – 7.47 (m, 2H).
^{13}C NMR (100 MHz, $CDCl_3$) δ 191.0 (^{13}C labeled), 141.1, 134.8 (d, *J* = 54.1 Hz), 131.0 (d, *J* = 4.3 Hz, 2C), 129.6 (d, *J* = 4.9 Hz, 2C).
HRMS (ESI) *m/z* calcd for $C_6{}^{13}CH_5ClO$ $[M+H]^+$:142.0141, found 142.0140
IR (cm^{-1}): 2925, 2852, 1659, 1587, 1574, 1202, 1091, 813, 539, 479.

[^{13}C] 3,4,5-trimethoxybenzaldehyde ([^{13}C]16)

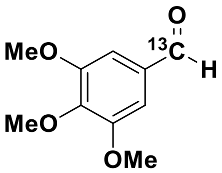

$C_9{}^{13}CH_{12}O_4$
MW: 197.19 g.mol^{-1}
Yield: 59%
White solid

The [^{13}C] 3,4,5-trimethoxybenzaldehyde (**[^{13}C]16**) was prepared accordingly to the **GP4c**, using $Pd(dba)_2$ (17 mg, 0.03 mmol, 5 mol%), PCy_3HBF_4 (11 mg, 0.03 mmol, 5 mol%), 5-iodo-1,2,3-trimethoxybenzene (176 mg, 0.6 mmol), potassium formate (101 mg, 1.2 mmol) and TBAI (66 mg, 0.18 mmol) in dry acetonitrile (3.5 mL) under argon. The reaction mixture was stirred 80 °C for 18 hours then the solvent was evaporated under *vacuum*. The crude was purified by FC on SiO_2 gel (Eluent Hept : EtOAc 70:30), providing the compound (**[^{13}C]16**) as white solid (42 mg, 59% yield, [^{13}C]CO_2 = 0.365 mmol as limiting reagent); mp 72-74 °C

^{1}H NMR (400 MHz, $CDCl_3$) δ 9.85 (d, J = 174.3 Hz, 1H), 7.11 (d, J = 5.4 Hz, 2H), 3.92 (s, 3H), 3.91 (s, 6H).
^{13}C NMR (100 MHz, $CDCl_3$) δ 191.1 (^{13}C labeled), 153.6 (d, J = 6.4 Hz, 2C), 143.5 (d, J = 0.5 Hz), 131.7 (d, J = 54.3 Hz), 106.7 (d, J = 4.5 Hz, 2C), 61.0, 56.2 (2C).
HRMS (ESI) *m/z* calcd for $C_9{}^{13}CH_{12}O_4$ $[M+H]^+$: 198.0842; found: 198.0841.
IR (cm^{-1}): 2942, 2840, 1645, 1582, 1421, 1385, 1326, 1231, 1122, 990, 845, 725, 623.

[^{13}C] 4-((trimethylsilyl)ethynyl)benzaldehyde ([^{13}C]17)

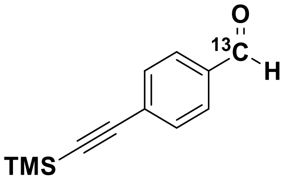

$C_{11}{}^{13}CH_{14}OSi$
MW: 203.32 g.mol^{-1}
Yield: 35%
Brown solid

The [^{13}C] 4-((trimethylsilyl)ethynyl)benzaldehyde (**[^{13}C]17**) was prepared accordingly to the **GP4b**, using Pd(dba)$_2$ (2.9 mg, 0.005 mmol, 5 mol%), PCy$_3$HBF$_4$ (1.9 mg, 0.005 mmol, 5 mol%), ((4-iodophenyl)ethynyl)trimethylsilane (31 mg, 0.1 mmol), potassium formate (17 mg, 0.2 mmol) and TBAI (11 mg, 0.03 mmol) in dry acetonitrile (1.5 mL) under argon. The reaction mixture was stirred 80 °C for 18 hours then the solvent was evaporated under *vacuum*. The crude was purified by FC on SiO$_2$ gel (Eluent Hept : EtOAc 90:10), providing the compound (**[^{13}C]17**) as brown solid (7 mg, 35% yield, [^{13}C]CO$_2$ = 0.365 mmol used in excess); mp 63-64 °C.

^{1}H NMR (400 MHz, CDCl$_3$) δ 10.0 (d, J = 174.9 Hz, 1H), 7.86 – 7.77 (m, 2H), 7.60 (d, J = 8.3 Hz, 2H), 0.27 (s, 9H).
^{13}C NMR (100 MHz, CDCl$_3$) δ 191.6 (^{13}C labeled), 135.7 (d, J = 53.1 Hz), 132.6 (d, J = 4.8 Hz, 2C), 129.6 (d, J = 4.1 Hz, 2C), 129.4 (d, J = 1.0 Hz), 103.9, 99.17, -0.07 (3C).
HRMS (ESI) *m/z* calcd for $C_{11}{}^{13}CH_{14}OSi$ [M+H]$^+$: 204.0919; found: 204.0920.
IR (cm^{-1}): 2957, 2925, 2730, 2156, 1660, 1599, 1249, 1202, 1151, 838, 759, 656, 536.

[^{13}C] 4-formylbenzonitrile ([^{13}C]18)

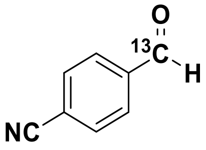

$C_7{}^{13}CH_5NO$
MW: 132.13 g.mol^{-1}
Yield: 38%
White solid

The [^{13}C] 4-formylbenzonitrile (**[^{13}C]18**) was prepared accordingly to the **GP4b**, using Pd(dba)$_2$ (2.9 mg, 0.005 mmol, 5 mol%), PCy_3HBF_4 (1.9 mg, 0.005 mmol, 5 mol%), 4-iodobenzonitrile (22.9 mg, 0.1 mmol), potassium formate (17 mg, 0.2 mmol) and TBAI (11 mg, 0.03 mmol) in dry acetonitrile (1.5 mL) under argon. The reaction mixture was stirred 80 °C for 18 hours then the solvent was evaporated under *vacuum*. The crude was purified by FC on SiO_2 gel (Eluent Hept : EtOAc 80:20), providing the compound (**[^{13}C]18**) as white solid (5 mg, 38% yield, [^{13}C]CO_2 = 0.365 mmol used in excess); mp 74-76 °C.

^{1}H NMR (400 MHz, $CDCl_3$) δ 10.1 (d, J = 178.3 Hz, 1H), 8.04 – 7.96 (m, 2H), 7.85 (d, J = 8.4 Hz, 2H).
^{13}C NMR (100 MHz, $CDCl_3$) δ 190.8 (^{13}C labeled), 160.2, 138.8 (d, J = 52.7 Hz), 133.1 (d, J = 4.7 Hz, 2C), 130.0 (d, J = 4.0 Hz; 2C), 117.9 (s), 117.8 (d, J = 1.0 Hz).
HRMS (ESI) *m/z* calcd for $C_7{}^{13}CH_5NO$ $[M+H]^+$: 133.0481, found 133.0483
IR (cm^{-1}): 2921, 2851, 2228, 1658, 1380, 1198, 1170, 827, 729, 545.

[^{13}C] 4-formylbenzoic acid ([^{13}C]19)

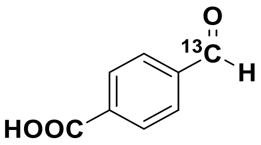

$C_7{}^{13}CH_6O_3$
MW: 151.13 g.mol^{-1}
Yield: 50%
White solid

The [^{13}C] 4-formylbenzoic acid (**[^{13}C]19**) was prepared accordingly to the **GP4a**, using Pd(dba)$_2$ (5.75 mg, 0.01 mmol), PCy_3HBF_4 (3.68 mg, 0.01 mmol), 4-iodoethylbenzoate (55.2 mg, 0.2 mmol), potassium formate (33.6 mg, 0.4 mmol) and TBAI (22 mg, 0.06 mmol) in dry acetonitrile (1.5 mL) under argon. The reaction mixture was stirred 80 °C for 18 hours then the solvent was evaporated under *vacuum*. The crude was dissolved in THF : Methanol (1 mL, 1:1) and *aq*. LiOH 5% (0.14 mL, 0.3 mmol) was added and the reaction was stirred for 2 hours at room temperature then the solution was basified with NaOH, the basic aqueous phase was separated, acidified and extracted with EtOAc providing the compound (**[^{13}C]19**) as white solid (15 mg, 50% yield, [^{13}C]CO_2 = 0.365 mmol used in excess); mp 244-247 °C.

^{1}H NMR (400 MHz, THF-*d*$_8$) δ 9.95 (d, *J* = 177.2 Hz, 1H), 8.07 (d, *J* = 8.3 Hz, 2H), 7.89 – 7.82 (m, 2H).

^{13}C NMR (100 MHz, THF-*d*$_8$) δ 192.2 (^{13}C labeled), 167.2, 140.6 (d, *J* = 52.4 Hz), 137.0, 131.1 (d, *J* = 4.6 Hz), 130.1 (d, *J* = 4.1 Hz).

HRMS (ESI) *m/z* calcd for $C_{14}{}^{13}CH_{18}N_2O$ [M-H]$^+$: 150.0276; found: 150.0277.

IR (cm^{-1}): 2924, 1717, 1691, 1663, 1637, 1423, 1290, 1109, 760.

Carbonylative Suzuki Coupling

[^{13}C]-(3,5-bis(trifluoromethyl)phenyl)(3,4,5-trimethoxyphenyl)methanone ([^{13}C]20)

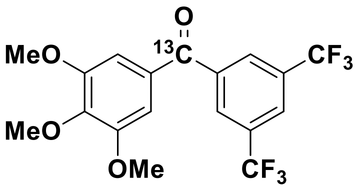

$C_{17}{}^{13}CH_{14}F_6O_4$
MW: 409.28 g.mol^{-1}
Yield: 65%
White solid

The [^{13}C] (3,5-bis(trifluoromethyl)phenyl)(3,4,5-trimethoxyphenyl)methanone (**[^{13}C]20**) was prepared accordingly to the **GP3**, using $PdCl_2$ (1.33 mg, 0.0075 mmol. 3 mol%), PPh_3 (3.93 mg, 0.015 mmol, 6 mol%), K_2CO_3 (103.5 mg, 0.75 mmol), 5-iodo-1,2,3-trimethoxybenzene (73.5 mg, 0.25 mmol), (3,5-bis(trifluoromethyl)phenyl)boronic acid (96.7 mg, 0.375 mmol) in dry anisole (2.0 mL) under argon. The reaction mixture was stirred at 80 °C for 18 hours then the solvent was evaporated under *vacuum*. The crude was purified by FC on SiO_2 gel (eluent Hept : EtOAc from 95:5 to 85:15), providing the compound (**[^{13}C]20**) as white solid (66.5 mg, 65% yield, [^{13}C]CO_2 = 0.445 mmol); m.p. 99-101 °C.

^{1}H NMR (400 MHz, $CDCl_3$) δ 8.24 (d, J = 3.2 Hz, 2H), 8.10 (s, 1H), 7.03 (d, J = 4.4 Hz, 2H), 3.98 (s, 3H), 3.88 (s, 6H).

^{13}C NMR (100 MHz, $CDCl_3$) δ 192.4 (^{13}C labeled), 153.2 (d, J = 5.9 Hz, 2C), 143.1 (d, J = 0.5 Hz), 139.5 (d, J = 54.9 Hz), 132.0 (dq, J = 34.1, 4.0 Hz, 2C), 130.6 (d, J = 57.5 Hz), 129.8 (2C), 125.45 (dt, J = 7.8, 4.0 Hz), 122.92 (q, J = 273.0 Hz, 2C), 107.73 (d, J = 3.3 Hz, 2C), 61.09, 56.3 (2C).

^{19}F NMR (376 MHz, $CDCl_3$) δ -62.9 (s, 6F).

IR (cm^{-1}):2926, 1576, 1414, 1318, 1278, 1221, 1167, 1171, 1123, 997, 909, 750.

LCMS (ESI) *m/z* $C_{17}{}^{13}CH_{14}F_6O_4$ [M+H]$^+$ 410.4.

HRMS (ESI) *m/z* calcd for $C_{17}{}^{13}CH_{14}F_6O_4$ [M+H]$^+$: 410.0908 ; found: 410.0905.

[^{13}C]- (2,4-difluorophenyl)(1H-indol-5-yl)methanone ([^{13}C]21)

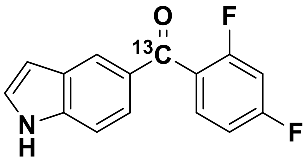

$C_{14}{}^{13}CH_9F_2NO$
MW: 258.23 g.mol^{-1}
Yield: 34%
Brown solid

The [^{13}C] (2,4-difluorophenyl)(1H-indol-5-yl)methanone (**[^{13}C]21**) was prepared accordingly to the **GP3**, using $PdCl_2$ (1.33 mg, 0.0075 mmol, 3 mol%), PPh_3 (3.93 mg, 0.015 mmol, 6 mol%), K_2CO_3 (103.5 mg, 0.75 mmol), 5-iodoindole (60.8 mg, 0.25 mmol), (2,4-difluorophenyl)boronic acid (59.2 mg, 0.375 mmol) in dry anisole (2.0 mL) under argon. The reaction mixture was stirred at 80 °C for 18 hours then the solvent was evaporated under *vacuum*. The crude was purified by FC on SiO_2 gel (Eluent Hept : EtOAc from 95:5 to 85:15), providing the compound (**[^{13}C]21**) as white solid (21.9 mg, 34% yield, [^{13}C]CO_2 = 0.445 mmol); m.p. 119-121 °C.

The [^{13}C] (2,4-difluorophenyl)(1H-indol-5-yl)methanone (**[^{13}C]21**) was also prepared using 0.6 mmol of [^{13}C]CO following the **GP0bis** in chamber 1 and **GP3** in chamber 2. The crude was purified by FC on SiO_2 gel (Eluent Hept : EtOAc from 95:5 to 80:20), providing the compound **[^{13}C]21** (57.2 mg, 88% yield, [^{13}C]CO_2 = 0.600 mmol).

^{1}H NMR (400 MHz, $CDCl_3$) δ 8.54 (brs, 1H), 8.12 – 8.09 (m, 1H), 7.83 – 7.78 (m, 1H), 7.61 – 7.54 (m, 1H), 7.47 – 7.42 (m, 1H), 7.29 (dd, J = 3.2, 2.4 Hz, 1H), 7.05 – 6.97 (m, 1H), 6.97 – 6.88 (m, 1H), 6.66 – 6.64 (m, 1H).

^{13}C NMR (100 MHz, $CDCl_3$) δ 192.7 (^{13}C labeled), 164.5 (dd, J = 252.8, 11.9 Hz), 160.7 (dd, J = 254.4, 12.3 Hz), 138.9, 132.2 (ddd, J = 10.1, 4.7, 1.4 Hz), 129.9 (d, J = 58.4 Hz), 127.5 (d, J = 5.1 Hz), 126.1, 125.4 (d, J = 2.9 Hz), 124.6 (ddd, J = 55.9, 15.6, 3.9 Hz), 123.7 (d, J = 3.3 Hz), 111.7 (dt, J = 21.4, 3.7 Hz), 111.3 (d, J = 4.6 Hz), 104.7 (td, J = 25.6, 2.4 Hz), 104.6.

^{19}F NMR (376 MHz, $CDCl_3$) δ -105.40 (d, J = 9.4 Hz), -107.24 (d, J = 9.4 Hz).

IR (cm^{-1}): 3292, 1594, 1560, 1307, 1264, 1250, 1095, 971, 818, 752, 732.

LCMS (ESI) *m/z* $C_{14}{}^{13}CH_9F_2NO$ $[M+H]^+$ 259.3.

HRMS (ESI) *m/z* calcd for $C_{14}{}^{13}CH_9F_2NO$ $[M+H]^+$: 259.0764 ; found: 259.0766.

[^{13}C]-furan-3-yl(4-methoxyphenyl)methanone ([^{13}C]22)

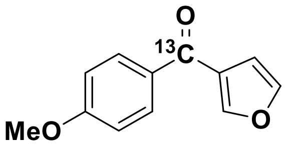

$C_{11}{}^{13}CH_{10}O_3$
MW: 203.20 g.mol^{-1}
Yield: 55%
White solid

The [^{13}C] furan-3-yl(4-methoxyphenyl)methanone (**[^{13}C]22**) was prepared accordingly to the **GP3**, using $PdCl_2$ (1.33 mg, 0.0075 mmol, 3 mol%), PPh_3 (3.93 mg, 0.015 mmol, 6 mol%), K_2CO_3 (103.5 mg, 0.75 mmol), 4-iodoanisole (58.5 mg, 0.25 mmol), furan-3-ylboronic acid (42 mg, 0.375 mmol) in dry anisole (2.0 mL) under argon. The reaction mixture was stirred at 80 °C for 18 hours then the solvent was evaporated under *vacuum*. The crude was purified by FC on SiO_2 gel (Eluent Hept : EtOAc 90:10), providing the compound (**[^{13}C]22**) as white solid (28 mg, 55% yield, [^{13}C]CO_2 = 0.445 mmol); m.p. 75-77 °C.

^{1}H NMR (400 MHz, $CDCl_3$) δ 7.91 (d, J = 0.5 Hz, 1H), 7.90 – 7.86 (m, 2H), 7.49 (t, J = 1.6 Hz, 1H), 6.97 (d, J = 8.9 Hz, 2H), 6.88 (d, J = 0.8 Hz, 1H), 3.88 (s, 3H).
^{13}C NMR (100 MHz, $CDCl_3$) δ 188.2 (^{13}C labeled), 163.4, 147.8 (d, J = 7 Hz), 143.8 (d, J = 4 Hz), 131.8, 131.3 (d, J = 4 Hz), 131.2, 126.9, 126.3, 113.9 (d, J = 4 Hz), 110.5 (d, J = 2 Hz), 55.6.
IR (cm^{-1}): 1595, 1502, 1317, 1259, 1165, 1156, 1014, 870, 839, 824, 787, 751, 702.
LCMS (ESI) *m/z* $C_{11}{}^{13}CH_{10}O_3$ $[M+H]^+$ 204.3.
HRMS (ESI) *m/z* calcd for $C_{11}{}^{13}CH_{10}O_3$ $[M+H]^+$: 204.0742 ; found: 204.0743.

[^{13}C] (4-methoxyphenyl)(phenyl)methanone ([^{13}C]23)

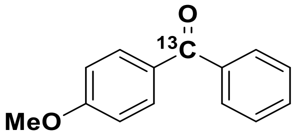

$C_{13}{}^{13}CH_{12}O_2$
MW: 213.24 g.mol^{-1}
Yield: 91%
White solid

The [^{13}C] (4-methoxyphenyl)(phenyl)methanone (**[^{13}C]23**) was prepared accordingly to the **GP3**, using $PdCl_2$ (1.33 mg, 0.0075 mmol, 3 mol%), PPh_3 (3.93 mg, 0.015 mmol, 6 mol%), K_2CO_3 (103.5 mg, 0.75 mmol), 4-iodoanisole (58.5 mg, 0.25 mmol), benzenboronic acid (45.8 mg, 0.375 mmol) in dry anisole (2.5 mL) under argon. The reaction mixture was stirred 80 °C for 18 hours then the solvent was evaporated under *vacuum*. The crude was purified by FC on SiO_2 gel (Eluent Hept : EtOAc 90:10), providing the compound (**[^{13}C]23**) as white solid (48 mg, 91% yield, [^{13}C]CO_2 = 0.445 mmol); mp 60-62 °C.

^{1}H NMR (400 MHz, $CDCl_3$) δ 7.60 – 7.51 (m, 4H), 7.47 – 7.39 (m, 2H), 7.35 – 7.29 (m, 1H), 7.04 – 6.96 (m, 2H), 3.86 (s, 3H).
^{13}C NMR (100 MHz, $CDCl_3$) δ 195.6 (^{13}C labeled), 163.3 (d, J = 0.6 Hz), 138.3 (d, J = 54.8 Hz), 132.6 (d, J = 3.2 Hz, 2C), 132.0 (d, J = 0.4 Hz), 130.2 (d, J = 57.0 Hz), 129.8 (d, J = 2.6 Hz, 2C), 128.3 (d, J = 4.0 Hz, 2C), 113.6 (d, J = 4.3 Hz, 2C), 55.6.
HRMS (ESI) *m/z* calcd for $C_{13}{}^{13}CH_{12}O_2$ $[M+H]^+$: 214.0946 ; found: 214.0943.
IR (cm^{-1}): 1592, 1444, 1310, 1254, 1168, 1148, 1021, 840, 692, 670, 597.

[^{13}C] (4-chlorophenyl)(4-fluorophenyl)methanone ([^{13}C]24)

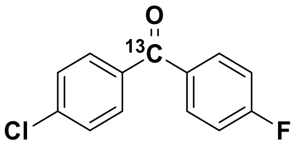

$C_{12}{}^{13}CH_8ClFO$
MW: 235.65 g.mol^{-1}
Yield: 78%
White solid

The [^{13}C] (4-chlorophenyl)(4-fluorophenyl)methanone (**[^{13}C]24**) was prepared accordingly to the **GP3**, using $PdCl_2$ (1.33 mg, 0.0075 mmol, 3 mol%), PPh_3 (3.93 mg, 0.015 mmol, 6 mol%), K_2CO_3 (103.5 mg, 0.75 mmol), 4-chloroiodobenzene (59.5 mg, 0.25 mmol), 4-fluorophenylboronic acid (52.5 mg, 0.375 mmol) in dry anisole (2.5 mL) under argon. The reaction mixture was stirred 80 °C for 18 hours then the solvent was evaporated under *vacuum*. The crude was purified by FC on SiO_2 gel (Eluent Hept : EtOAc 90:10), providing the compound (**[^{13}C]24**) as white solid (46 mg, 78% yield, [^{13}C]CO_2 = 0.445 mmol); mp 114-115 °C.

^{1}H NMR (400 MHz, $CDCl_3$) δ 7.86 – 7.76 (m, 2H), 7.76 – 7.68 (m, 2H), 7.51 – 7.42 (m, 2H), 7.22 – 7.10 (m, 2H).
^{13}C NMR (100 MHz, $CDCl_3$) δ 194.2 (^{13}C labeled), 165.6 (dd, J = 254.7, 0.9 Hz), 139.1 (d, J = 1.0 Hz), 135.9 (d, J = 55.7 Hz), 133.6 (dd, J = 56.3, 3.1 Hz), 132.7 (dd, J = 9.2, 3.1 Hz, 2C), 131.4 (d, J = 2.9 Hz, 2C), 128.8 (d, J = 4.2 Hz, 2C), 115.5 (dd, J = 21.9, 4.4 Hz, 2C).
^{19}F NMR (376 MHz, $CDCl_3$) δ -105.4.
HRMS (ESI) *m/z* calcd for $C_{12}{}^{13}CH_8ClFO$ $[M+H]^+$: 236.0358 ; found: 236.0354.
IR (cm^{-1}): 1582, 1504, 1482, 1226, 1145, 1085, 1012, 837, 750, 664, 515, 470.

[^{13}C]-4-(4-phenoxybenzoyl)benzonitrile ([^{13}C]25)

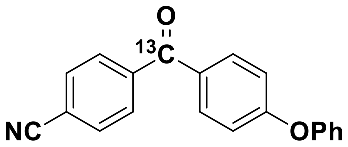

$C_{19}{}^{13}CH_{13}NO_2$
MW: 300.32 g.mol^{-1}
Yield: 49%
Yellow solid

The [^{13}C] 4-(4-phenoxybenzoyl)benzonitrile (**[^{13}C]25**) was prepared accordingly to the **GP3**, using $PdCl_2$ (1.33 mg, 0.0075 mmol, 3 mol%), PPh_3 (3.93 mg, 0.015 mmol, 6 mol%), K_2CO_3 (103.5 mg, 0.75 mmol), 4-iodobenzonitrile (57.2 mg, 0.25 mmol), (4-phenoxyphenyl)boronic acid (80.2 mg, 0.375 mmol) in dry anisole (2.0 mL) under argon. The reaction mixture was stirred at 80 °C for 18 hours then the solvent was evaporated under *vacuum*. The crude was purified by FC on SiO_2 gel (Eluent Hept : EtOAc from 95:5 to 85:15), providing the compound (**[^{13}C]25**) as a light yellow solid (36.8 mg, 49% yield, [^{13}C]CO_2 = 0.445 mmol); m.p. 115-118 °C.

^{1}H NMR (400 MHz, $CDCl_3$) δ 7.87 – 7.81 (m, 2H), 7.82 – 7.75 (m, 4H), 7.45 – 7.39 (m, 2H), 7.25 – 7.20 (m, 1H), 7.12 – 7.08 (m, 2H), 7.06 – 7.01 (m, 2H).
^{13}C NMR (100 MHz, $CDCl_3$) δ 193.8 (^{13}C labeled), 162.6, 155.3, 141.8 (d, J = 54.0 Hz), 132.7 (d, J = 3.2 Hz, 2C), 132.3 (d, J = 4.0 Hz, 2C), 130.6 (d, J = 57.6 Hz), 130.3, 130.1 (d, J = 2.6 Hz, 2C), 125.1 (2C), 120.5 (2C), 118.2, 117.3 (d, J = 4.4 Hz, 2C), 115.5.
IR (cm^{-1}): 2233, 1600, 1593, 1584, 1490, 1267, 1252, 1164, 918, 854, 841, 758, 670.
LCMS (ESI) *m/z* $C_{19}{}^{13}CH_{13}NO_2$ [M+H]$^+$ 301.7.
HRMS (ESI) *m/z* calcd for $C_{19}{}^{13}CH_{13}NO_2$ [M+H]$^+$: 301.1058; found: 301.1058.

Carbon Isotope Exchange

[^{13}C] benzoic acid ([^{13}C]26)

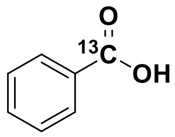

$C_6{}^{13}CH_6O_2$
MW: 123.12 g.mol^{-1}
Yield: 50%
White solid

The [^{13}C] benzoic acid (**[^{13}C]26**) was prepared accordingly to the **GP6**, using $Pd_2(dba)_3$ (9.1 mg, 0.01 mmol, 5 mol%), P(*o*-tol)$_3$ (12.2 mg, 0.04 mmol, 20 mol%), benzoyl chloride (28 mg, 0.2 mmol) in dry toluene (1.5 mL) under argon. The solution is injected *via* syringe in Chamber 2. The reaction mixture was stirred at 75 °C for 16 hours and quenched using 2 mL of NaOH 1M. A basic work-up is followed by the acidification of the aqueous phase to provide the compound (**[^{13}C]26**) as a white solid (12 mg, 50% yield, [^{13}C]CO_2 = 0.365 mmol)

^{1}H NMR (400 MHz, $CDCl_3$) δ 8.14-8.12 (m, 2H), 7.66-7.61 (m, 1H), 7.51-7.47 (m, 2H).
^{13}C NMR (100 MHz, $CDCl_3$) δ 172.2 (^{13}C labeled), 134.0, 130.4 (2C), 129.4, 128.6 (2C).
IR (cm^{-1}): 2919, 2850, 1672, 1645, 1601, 1581, 1452, 1410, 1272, 1184, 1124, 931, 800, 700, 667.
Isotopic Enrichment: +1 ^{13}C, 49.2%.

[^{13}C]-4-methoxybenzoic acid ([^{13}C]27)

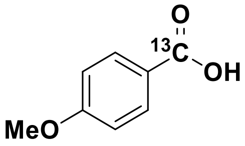

$C_7{}^{13}CH_8O_3$
MW: 153.14 g.mol^{-1}
Yield: 73%
White solid

The [^{13}C] 4-methoxybenzoic acid (**[^{13}C]27**) was prepared accordingly to the **GP6**, using $Pd_2(dba)_3$ (9.1 mg, 0.01 mmol, 5 mol%), P(*o*-tol)$_3$ (12.2 mg, 0.04 mmol, 20 mol%), 4-methoxybenzoyl chloride (27 µL, 0.2 mmol), in dry toluene (1.0 mL) under argon. The solution is injected *via* syringe in Chamber 2. The reaction mixture was stirred at 75 °C for 16 hours and quenched using 2 mL of NaOH 1M. A basic work-up is followed by the acidification of the aqueous phase to provide the compound (**[^{13}C]27**) as a white solid (22.5 mg, 73% yield, [^{13}C]CO_2 = 0.365 mmol); m.p. 181-183 °C.

^{1}H NMR (400 MHz, CDCl$_3$) δ 8.01 – 7.96 (m, 2H), 7.02 – 6.97 (m, 2H), 3.88 (s, 3H).
^{13}C NMR (100 MHz, CDCl$_3$) δ 169.8 (^{13}C labeled), 165.1, 132.8 (2C), 124.1, 114.7 (2C), 56.0.
IR (cm^{-1}): 1661, 1600, 1295, 1257, 1164, 1127, 1103, 1024, 923, 841, 773, 761.
HRMS (ESI) *m/z* calcd for $C_7{}^{13}CH_8O_3$ $[M+H]^+$ 154.0580; found: 154.0579.
Isotopic Enrichment: +1 ^{13}C, 47.2%.

[^{13}C]- 2-fluorobenzoic acid ([^{13}C]28)

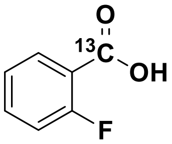

$C_6{}^{13}CH_5FO_2$
MW: 141.10 g.mol^{-1}
Yield: 51%
White solid

The [^{13}C] 2-fluorobenzoic acid (**[^{13}C]28**) was prepared accordingly to the **GP6**, using $Pd_2(dba)_3$ (9.1 mg, 0.01 mmol, 5 mol%), P(*o*-tol)$_3$ (12.2 mg, 0.04 mmol, 20 mol%), 2-fluorobenzoyl chloride (31.7 mg, 0.2 mmol), in dry toluene (1.0 mL) under argon. The solution is injected *via* syringe in Chamber 2. The reaction mixture was stirred at 75 °C for 16 hours and quenched using 2 mL of NaOH 1M. A basic work-up is followed by the acidification of the aqueous phase to provide the compound (**[^{13}C]28**) as a white solid (14.3 mg, 51% yield, [^{13}C]CO_2 = 0.365 mmol); m.p. 121-123 °C.

^{1}H NMR (400 MHz, CDCl$_3$) δ 8.04 (td, J = 7.6, 1.9 Hz, 1H), 7.62 – 7.55 (m, 1H), 7.27 – 7.22 (m, 1H), 7.21 – 7.15 (m, 1H).
^{13}C NMR (100 MHz, CDCl$_3$) δ 169.8 (d, J_{C-F} = 3 Hz, ^{13}C labeled), 162.8 (d, J_{C-F} = 261 Hz), 135.7 (d, J_{C-F} = 10 Hz), 132.9, 124.3 (d, J_{C-F} = 4 Hz), 117.7 (d, J_{C-F} = 9 Hz), 117.3, (d, J_{C-F} = 22 Hz).
^{19}F NMR (376 MHz, CDCl$_3$) δ -108.31.
IR (cm^{-1}): 1676, 1612, 1464, 1283, 1230, 1087, 915, 844, 750, 650.
HRMS (ESI) *m/z* calcd for $C_6{}^{13}CH_5FO_2$ [M+H]$^+$ 142.0380; found: 142.0381.
Isotopic Enrichment: +1 ^{13}C, 19.4%.

[^{13}C]- 4-(tert-butyl)benzoic acid ([^{13}C]29)

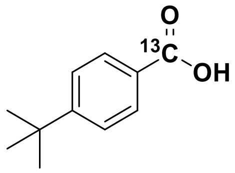

$C_{10}{}^{13}CH_{14}O_2$
MW: 179.22 g.mol^{-1}
Yield: 16%
White solid

The [^{13}C] 4-(tert-butyl)benzoic acid (**[^{13}C]29**) was prepared accordingly to the **GP6**, using $Pd_2(dba)_3$ (9.1 mg, 0.01 mmol, 5 mol%), P(*o*-tol)$_3$ (12.2 mg, 0.04 mmol, 20 mol%), 4-(*tert*-butyl)benzoyl chloride (39.3 mg, 0.2 mmol), in dry toluene (1.0 mL) under argon. The solution is injected *via* syringe in Chamber 2. The reaction mixture was stirred at 75 °C for 16 hours and quenched using 2 mL of NaOH 1M. A basic work-up is followed by the acidification of the aqueous phase to provide the compound (**[^{13}C]29**) as a white solid (5.9 mg, 16% yield, [^{13}C]CO_2 = 0.365 mmol); m.p. 157-159 °C.

^{1}H NMR (400 MHz, $CDCl_3$) δ 8.04 (d, *J* = 8 Hz, 2H), 7.49 (d, *J* = 8 Hz, 2H), 1.35 (s, 9H).
^{13}C NMR (100 MHz, $CDCl_3$) δ 171.8 (^{13}C labeled), 157.6, 135.0, 130.2 (2C), 125.6 (2C), 35.3, 31.3 (3C).
IR (cm^{-1}): 2922, 2853, 1668, 1643, 1609, 1278, 1185, 1131, 1013, 936, 853, 770, 699.
HRMS (ESI) *m/z* calcd for $C_{10}{}^{13}CH_{14}O_2$ [M-H]$^-$ 178.0955; found: 178.0954.
Isotopic Enrichment: +1 ^{13}C, 56.8%.

[^{13}C]- 2-phenylacetic acid ([^{13}C]30)

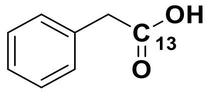

$C_7{}^{13}CH_8O_2$
MW: 137.14 g.mol^{-1}
Yield: 57%
White solid

The [^{13}C] 2-phenylacetic acid (**[^{13}C]30**) was prepared accordingly to the **GP6**, using $Pd_2(dba)_3$ (9.1 mg, 0.01 mmol, 5 mol%), P(*o*-tol)$_3$ (12.2 mg, 0.04 mmol, 20 mol%), 2-phenylacetyl chloride (30.9 mg, 0.2 mmol), in dry toluene (1.0 mL) under argon. The solution is injected *via* syringe in Chamber 2. The reaction mixture was stirred at 75 °C for 16 hours and quenched using 2 mL of NaOH 1M. A basic work-up is followed by the acidification of the aqueous phase to provide the compound (**[^{13}C]30**) as a white solid (15.6 mg, 57% yield, [^{13}C]CO_2 = 0.365 mmol); m.p. 75-76 °C.

^{1}H NMR (400 MHz, $CDCl_3$) δ 7.38 – 7.32 (m, 2H), 7.32 – 7.27 (m, 3H), 3.66 (t, J = 4 Hz, 2H).
^{13}C NMR (100 MHz, $CDCl_3$) δ 178.1 (^{13}C labeled), 133.4 (t, J = 2 Hz), 129.5 (2C), 128.8 (2C), 127.5, 41.2 (s^{12C} + d^{13C}, J = 28 Hz).
IR (cm^{-1}):1686, 1662, 1406, 1335, 1221, 1181, 891, 838, 751, 698, 675, 602.
HRMS (ESI) ***m/z*** calcd for $C_7{}^{13}CH_8O_2$ [M-H]$^-$ 136.0485; found: 136.0485.
Isotopic Enrichment: +1 ^{13}C, 56.5%.

[^{13}C]- terephthalic acid ([^{13}C]31)

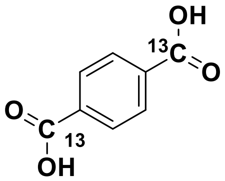

$C_6{}^{13}C_2H_6O_4$
MW: 168.12 g.mol^{-1}
Yield: 54%
White solid

The [^{13}C] terephthalic acid (**[^{13}C]31**) was prepared accordingly to the **GP6**, using $Pd_2(dba)_3$ (9.1 mg, 0.01 mmol, 5 mol%), P(*o*-tol)$_3$ (12.2 mg, 0.04 mmol, 20 mol%), terephthaloyl dichloride (20.2 mg, 0.1 mmol), in dry toluene (1.0 mL) under argon. The solution is injected *via* syringe in Chamber 2. The reaction mixture was stirred at 75 °C for 16 hours and quenched using 2 mL of NaOH 1M. A basic work-up is followed by the acidification of the aqueous phase to provide the compound (**[^{13}C]31**) as a white solid (9.1 mg, 54% yield, [^{13}C]CO_2 = 0.365 mmol); mp.> 350 °C.

^{1}H NMR (400 MHz, DMSO-*d$_6$*) δ 13.30 (brs, 2H), 8.04 (t, J = 1.2 Hz, 4H).
^{13}C NMR (100 MHz, DMSO-*d$_6$*) δ 166.7 (2C, ^{13}C labeled), 134.5 (2C), 129.5 (4C).
IR (cm^{-1}): 2921, 1667, 1647, 1410, 1277, 1133, 1111, 1018, 931, 876, 779, 717, 610.
HRMS (ESI) *m/z* calcd for $C_6{}^{13}C_2H_6O_4$ [M-H]$^-$ 167.0260; found: 167. 0258.
Isotopic Enrichment: +1 ^{13}C, 43.5%, +2 ^{13}C 12.3%.

Carbonylative Sonogashira Coupling

[^{13}C]-4-(3-(4-methoxyphenyl)propioloyl)benzonitrile ([^{13}C]32)

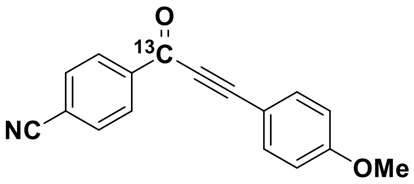

$C_{16}{}^{13}CH_{11}NO_2$
MW: 262.27 g.mol^{-1}
Yield: 32%
Light yellow solid

The [^{13}C] 4-(3-(4-methoxyphenyl)propioloyl)benzonitrile (**[^{13}C]32**) was prepared accordingly to the **GP5**, using $PdCl_2$ (1.77 mg, 0.01 mmol, 5 mol%), XantPhos (5.79 mg, 0.01 mmol, 5 mol%), Et_3N (84 µL, 0.6 mmol), 4-bromobenzonitrile (36.4 mg, 0.2 mmol), 1-ethynyl-4-methoxybenzene (39 µL, 0.3 mmol) in dry dioxane (1.0 mL) under argon. The reaction mixture was stirred at 75 °C for 16 hours then the solvent was evaporated under *vacuum*. The crude was purified by FC on SiO_2 gel (Eluent Hept : EtOAc from 95:5 to 85:15), providing the compound (**[^{13}C]32**) as white solid (16.8 mg, 32% yield, [^{13}C]CO_2 = 0.365 mmol); m.p. 144-146 °C.

^{1}H NMR (400 MHz, $CDCl_3$) δ 8.32 – 8.27 (m, 2H), 7.86 – 7.78 (m, 2H), 7.68 – 7.63 (m, 2H), 6.98 – 6.93 (m, 2H), 3.87 (s, 3H).
^{13}C NMR (100 MHz, $CDCl_3$) δ 176.3 (^{13}C labeled), 162.3, 140.0 (d, J = 60.5 Hz), 135.6 (2C), 132.6 (d, J = 4.7 Hz, 2C), 129.9 (d, J = 3.4 Hz, 2C), 118.1, 117.0, 114.7 (2C), 111.3 (d, J = 2.0 Hz), 96.7 (d, J = 14.7 Hz), 86.8 (d, J = 95.2 Hz), 55.6.
IR (cm^{-1}): 2922, 2190, 1596, 1284, 1258, 1166, 1109, 1018, 853, 828, 804, 729, 666.
LCMS (ESI) *m/z* $C_{16}{}^{13}CH_{11}NO_2$ $[M+H]^+$ 263.7.
HRMS (ESI) *m/z* calcd for $C_{16}{}^{13}CH_{11}NO_2$ $[M+H]^+$: 263.0901 ; found: 263.0903.

[^{13}C]- 1-(4-acetylphenyl)-3-(6-methoxynaphthalen-2-yl)prop-2-yn-1-one ([^{13}C]33)

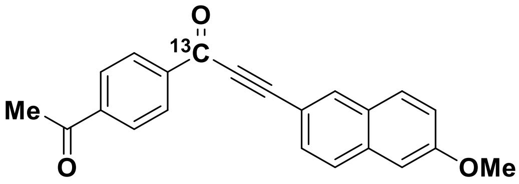

$C_{21}{}^{13}CH_{16}O_3$
MW: 329.35 g.mol^{-1}
Yield: 32%
Yellow solid

The [^{13}C] 1-(4-acetylphenyl)-3-(6-methoxynaphthalen-2-yl)prop-2-yn-1-one (**[^{13}C]33**) was prepared accordingly to the **GP5**, using $PdCl_2$ (1.77 mg, 0.01 mmol, 5 mol%), XantPhos (5.79 mg, 0.01 mmol, 5 mol%), Et_3N (84 µL, 0.6 mmol), 1-(4-iodophenyl)ethan-1-one (49.2 mg, 0.2 mmol), 2-ethynyl-6-methoxynaphthalene (54.6 mg, 0.3 mmol) in dry dioxane (1.0 mL) under argon. The reaction mixture was stirred at 75 °C for 16 hours then the solvent was evaporated under *vacuum*. The crude was purified by FC on SiO_2 gel (Eluent Hept : EtOAc from 9:1 to 8:2), providing the compound (**[^{13}C]33**) as yellow solid (21 mg, 32% yield, [^{13}C]CO_2 = 0.365 mmol); m.p. 159-161 °C.

^{1}H NMR (400 MHz, $CDCl_3$) δ 8.37 – 8.31 (m, 2H), 8.19 (s, 1H), 8.10 (d, J = 8.1 Hz, 2H), 7.77 (dd, J = 8.7, 5.1 Hz, 2H), 7.65 (dd, J = 8.5, 1.6 Hz, 1H), 7.22 (dd, J = 9.0, 2.5 Hz, 1H), 7.15 (d, J = 2.4 Hz, 1H), 3.95 (s, 3H), 2.68 (s, 3H).

^{13}C NMR (100 MHz, $CDCl_3$) δ 197.6, 177.3 (^{13}C labeled), 159.7, 140.8, 140.2 (d, J = 60.6 Hz), 135.9, 134.7, 130.0, 129.8 (d, J = 3.5 Hz, 2C), 129.3, 128.6 (d, J = 4.7 Hz, 2C), 128.3, 127.5, 120.3, 114.5, 106.1, 95.9 (d, J = 14.0 Hz), 87.2 (d, J = 93.5 Hz), 55.6, 27.1.

IR (cm^{-1}): 2182, 1687, 1596, 1259, 1166, 1121, 1019, 1007, 849, 841, 744, 669.

LCMS (ESI) *m/z* $C_{21}{}^{13}CH_{16}O_3$ [M+H]$^+$ 330.7.

HRMS (ESI) *m/z* calcd for $C_{21}{}^{13}CH_{16}O_3$ [M+H]$^+$: 330.1211 ; found: 330.1212.

[^{13}C]-1,3-diphenylprop-2-yn-1-one ([^{13}C]34)

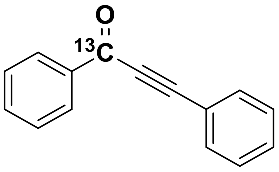

$C_{14}{}^{13}CH_{10}O$
MW: 207.23 g.mol^{-1}
Yield: 66%
White solid

The [^{13}C] 1,3-diphenylprop-2-yn-1-one (**[^{13}C]34**) was prepared accordingly to the **GP5**, using $PdCl_2$ (1.77 mg, 0.01 mmol, 5 mol%), XantPhos (5.79 mg, 0.01 mmol, 5 mol%), DiPEA (104 µL, 0.6 mmol), iodobenzene (22.3 µL, 0.2 mmol), phenylacetylene (33 µL, 0.3 mmol) in dry dioxane (1.0 mL) under argon. The reaction mixture was stirred at 75 °C for 16 hours then the solvent was evaporated under *vacuum*. The crude was purified by FC on SiO_2 gel (Eluent Hept : EtOAc from 95:5 to 85:15), providing the compound (**[^{13}C]-34**) as white solid (27.7 mg, 66% yield, [^{13}C]CO_2 = 0.365 mmol).

^{1}H NMR (400 MHz, $CDCl_3$) δ 8.26 – 8.21 (m, 2H), 7.72 – 7.67 (m, 2H), 7.66 – 7.61 (m, 1H), 7.56 – 7.47 (m, 3H), 7.46 – 7.40 (m, 2H).

^{13}C NMR (100 MHz, $CDCl_3$) δ 178.2 (^{13}C labeled), 137.0 (d, J = 61 Hz), 134.3, 133.2 (2C), 131.0, 129.7 (d, J = 3Hz, 2C), 128.8 (2C), 128.7 (d, J = 5 Hz, 2C), 120.3 (d, J = 2 Hz), 93.3 (d, J = 14 Hz), 87.0 (d, J = 91 Hz).

IR (cm^{-1}): 2196, 1595, 1577, 1478, 1458, 1205, 1195, 1150, 1067, 991, 820, 758, 694.

LCMS (ESI) *m/z* $C_{14}{}^{13}CH_{10}O$ [M+H]$^+$ 208.6.

HRMS (ESI) *m/z* calcd for $C_{14}{}^{13}CH_{10}O$ [M+H]$^+$: 208.0844 ; found: 208.0845.

[^{13}C]- 1,3,5-triphenyl-1H-pyrazole ([^{13}C]35)

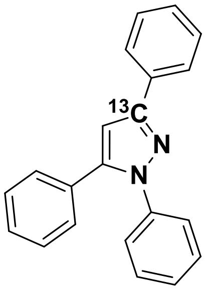

$C_{20}{}^{13}CH_{16}N_2$
MW: 297.36 g.mol^{-1}
Yield: 73%
Yellow solid

To a solution of [^{13}C] 1,3-diphenylprop-2-yn-1-one (10.4 mg, 0.05 mmol) (**[^{13}C]34**) in DMF (0.5 mL) was added phenylhydrazine (7.4 µL, 0.075 mmol), the solution was then stirred at 80 °C for 12 hours. The solvent was evaporated under *vacuum* and the crude was purified by FC on SiO_2 gel (Eluent Hept : EtOAc from 95:5 to 9:1), providing the compound (**[^{13}C]35**) as yellow solid (10.9 mg, 73% yield); m.p. 130-131 °C.[20]

^{1}H NMR (400 MHz, $CDCl_3$) δ 7.96 – 7.90 (m, 2H), 7.47 – 7.41 (m, 2H), 7.40 – 7.28 (m, 11H), 6.84 (d, *J* = 7.9 Hz, 1H).

^{13}C NMR (100 MHz, $CDCl_3$) δ 152.1, 144.5 (^{13}C labeled), 140.3 (d, *J* = 3 Hz), 133.2 (d, *J* = 2 Hz), 130.7 (d, *J* = 64 Hz), 129.1 (2C), 128.9 (d, *J* = 2 Hz, 2C), 128.8 (2C), 128.6 (d, *J* = 4 Hz, 2C), 128.4, 128.1, 127.6, 125.8 (2C) , 125.3 (2C), 105.3 (d, *J* = 68 Hz).

IR (cm^{-1}): 2924, 1728, 1595, 1574, 1494, 1481, 1455, 1205, 1152, 1066, 971, 763, 691.

LCMS (ESI) *m/z* $C_{20}{}^{13}CH_{16}N_2$ $[M+H]^+$ 298.6.

[^{13}C]- phenyl(2-phenylbenzofuran-3-yl)methanone ([^{13}C]36)

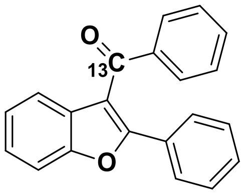

$C_{20}{}^{13}CH_{14}O_2$
MW: 299.33 g.mol^{-1}
Yield: 56%
Yellow solid

To a solution of [^{13}C] 1,3-diphenylprop-2-yn-1-one (10.4 mg, 0.05 mmol) (**[^{13}C]34**) in DMF (0.5 mL) was added 2-iodophenol (11 mg, 0.05 mmol), K_2CO_3 (35 mg, 0.25 mmol), LiCl (2.1 mg, 0.05 mmol), $Pd(OAc)_2$ (1.1 mg, 0.005 mmol, 10 mol%), PPh_3 (1.3 mg, 0.005 mmol, 10 mol%), the solution was then stirred at 100 °C for 12 hours. The solvent was evaporated under *vacuum* and the crude was purified by FC on SiO_2 gel (Eluent Hept : EtOAc from 95:5 to 9:1), providing the compound (**[^{13}C]36**) as yellow solid (8.4 mg, 56% yield); m.p. 94-97 °C.[21]

^{1}H NMR (400 MHz, $CDCl_3$) δ 7.86 – 7.81 (m, 2H), 7.71 – 7.64 (m, 2H), 7.61 – 7.54 (m, 2H), 7.52 – 7.46 (m, 1H), 7.40 – 7.34 (m, 2H), 7.33 – 7.27 (m, 5H).

^{13}C NMR (100 MHz, $CDCl_3$) δ 192.5 (^{13}C labeled), 157.8 (t, J = 3.0 Hz), 154.0, 137.9 (d, J = 56.2 Hz), 133.3 (d, J = 0.8 Hz), 130.0, 129.9, 129.9, 129.6, 128.6 (m, 7C), 125.5, 124.0, 121.6, 116.3 (d, J = 62.7 Hz), 111.4.

IR (cm^{-1}): 1232, 1120, 1058, 1024, 877, 789, 752, 720, 695, 685, 668.

LCMS (ESI) *m/z* $C_{20}{}^{13}CH_{14}O_2$ $[M+H]^+$ 300.6.

HRMS (ESI) *m/z* calcd for $C_{20}{}^{13}CH_{14}O_2$ $[M+H]^+$: 300.1106 ; found: 300.1104.

III.Supplementary Discussion

^{13}C Labeling of Pharmaceutical Relevant Compounds

[^{13}C]- (3-fluoro-4-methoxyphenyl)(3,4,5-trimethoxyphenyl)methanone ([^{13}C]37)

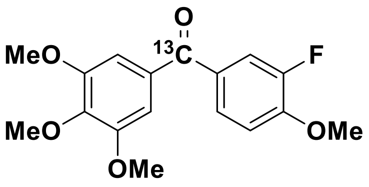

$C_{16}{}^{13}CH_{17}FO_5$
MW: 321.30 g.mol^{-1}
Yield: 48%
Yellow solid

The [^{13}C] (3-fluoro-4-methoxyphenyl)(3,4,5-trimethoxyphenyl)methanone (**[^{13}C]37**) was prepared accordingly to the **GP3**, using $PdCl_2$ (1.33 mg, 0.0075 mmol, 3 mol%), PPh_3 (3.93 mg, 0.015 mmol, 6 mol%), K_2CO_3 (103.5 mg, 0.75 mmol), 5-iodo-1,2,3-trimethoxybenzene (73.5 mg, 0.25 mmol), (3-fluoro-4-methoxyphenyl)boronic acid (63.7 mg, 0.375 mmol) in dry anisole (2.0 mL) under argon. The reaction mixture was stirred at 80 °C for 18 hours then the solvent was evaporated under *vacuum*. The crude was purified by FC on SiO_2 gel (eluent Hept : EtOAc from 95:5 to 80:20), providing the compound (**[^{13}C]37**) as a light yellow solid (38.6 mg, 48% yield, [^{13}C]CO_2 = 0.445 mmol); m.p. 124-126 °C.

^{1}H NMR (400 MHz, CDCl$_3$) δ 7.64 – 7.61 (m, 1H), 7.61 – 7.58 (m, 1H), 7.05 – 7.01 (m, 1H), 7.01 (d, J = 4 Hz, 2H), 3.97 (s, 3H), 3.93 (s, 3H), 3.88 (s, 6H).
^{13}C NMR (100 MHz, CDCl$_3$) δ 193.7 (d, J = 1.7 Hz, ^{13}C labeled), 153.1 (d, J = 5.7 Hz, 2C), 151.9 (dd, J = 248.1, 5.6 Hz), 151.6 (d, J = 10.8 Hz), 141.9 (d, J = 0.7 Hz), 132.8 (d, J = 55.8 Hz), 130.6 (dd, J = 57.0, 5.2 Hz), 127.6 (t, J = 3.2 Hz), 117.9 (dd, J = 19.2, 3.0 Hz), 112.3 (dd, J = 4.8, 1.8 Hz), 107.5 (d, J = 3.1 Hz, 2C), 61.1, 56.4 (3C).
^{19}F NMR (376 MHz, CDCl$_3$) δ -134.18 (d, J = 1.7 Hz).
IR (cm^{-1}): 1329, 1318, 1290, 1231, 1172, 1127, 1111, 1020, 997, 810, 765, 744.
HRMS (ESI) ***m/z*** calcd for $C_{16}{}^{13}CH_{17}FO_5$ $[M+H]^+$ 322.1166; found: 322.1165.

[^{13}C]-isoFCA-4 ([^{13}C]38)

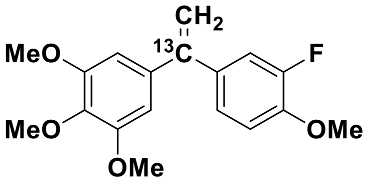

$C_{17}{}^{13}CH_{19}FO_4$
MW: 319.33 g.mol^{-1}
Yield: 78%
Yellow solid

A solution of methyltriphenylphosphonium bromide (32.2 mg; 0.09 mmol) in THF (1.5 mL) was cooled to 0 °C and slowly treated with LiHMDS 1M in THF (90 µL, 0.09 mmol). The yellow ylide solution was stirred at 0 °C for 1 h, then slowly treated with a solution of (3-fluoro-4-methoxyphenyl)(3,4,5-trimethoxyphenyl)methanone **[^{13}C]37** (16 mg, 0.05 mmol) in THF (2 mL) *via* syringe. The resulting mixture was allowed to warm to room temperature and stirred for another 1 h. The solution was poured in H_2O (3 mL) and extracted with CH_2Cl_2 (2x5 mL). The organic layers were combined, dried ($MgSO_4$), filtered and concentrated. The crude was purified by FC on SiO_2 gel (eluent Hept : EtOAc from 95:5 to 9:1), providing the compound (**[^{13}C]38**) as yellow solid (12.5 mg, 78% yield); m.p. 92-95 °C.

^{1}H NMR (400 MHz, $CDCl_3$) δ 7.14 – 7.05 (m, 2H), 6.92 (t, J = 8.5 Hz, 1H), 6.55 – 6.50 (m, 2H), 5.36 (dt, J = 14.9, 1.2 Hz, 2H), 3.91 (s, 3H), 3.88 (s, 3H), 3.82 (s, 6H).
^{13}C NMR (100 MHz, $CDCl_3$) δ 153.1 (d, J = 5.5 Hz, 2C), 152.2 (dd, J = 245.4, 5.5 Hz), 148.9 (d, J = 1.7 Hz, ^{13}C labeled), 147.5 (d, J = 10.8 Hz), 137.4 (d, J = 138.2 Hz), 137.3, 134.5 (dd, J = 55.5, 6.3 Hz), 124.2 (dd, J = 3.2, 1.8 Hz), 116.0 (dd, J = 18.8, 2.3 Hz), 113.5 (d, J = 72.6 Hz), 113.0 (dd, J = 4.7, 2.2 Hz), 105.8 (d, J = 2.1 Hz, 2C), 61.0, 56.4, 56.3 (2C).
^{19}F NMR (376 MHz, $CDCl_3$) δ -135.6.
IR (cm^{-1}): 1571, 1505, 1328, 1318, 1290, 1231, 1171, 1124, 1020, 997, 736, 745.
HRMS (ESI) *m/z* calcd for $C_{17}{}^{13}CH_{19}FO_4$ $[M+H]^+$ 320.1374; found: 320.1373.

[^{13}C] 3,4-diphenylfuran-2(5H)-one ([^{13}C]39)

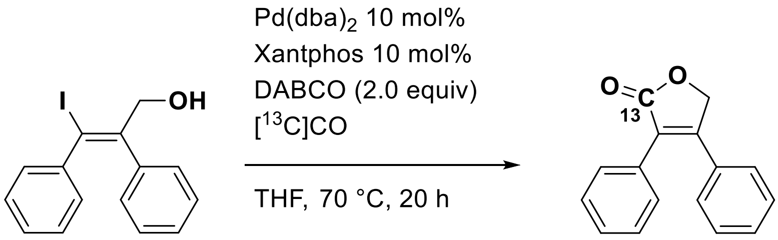

$C_{15}{}^{13}CH_{12}O_2$
MW: 237.26 g.mol^{-1}
Yield: 71%
Pale brown solid

Glassware Type 1 (Figure S1) was used. The [^{13}C] 3,4-diphenylfuran-2(5H)-one (**[^{13}C]39**) was prepared using Pd(dba)$_2$ (8.7 mg, 0.015 mmol, 10 mol%), Xantphos (8.7 mg, 0.015 mmol, 10 mol%), (Z)-3-iodo-2,3-diphenylprop-2-en-1-ol (*see preparation below*) (50.4 mg, 0.15 mmol) and DABCO (33.5 mg, 0.30 mmol) in dry THF (2.0 mL) under argon. The solution is then transferred *via* syringe (previously removing the argon from the syringe) into Chamber 2 of the Two-Chamber Glassware (*note*: this chamber is under *vacuum*). Stopcock between Chamber 1 (where [^{13}C]CO is previously produced by photoreduction following **GP0**) and 2 is then open. The reaction mixture was stirred at 70 °C for 20 h then the solvent was evaporated under *vacuum*. The crude was purified by FC on SiO_2 gel (Eluent Hept : EtOAc 80:20), providing the compound (**[^{13}C]39**) as pale brown solid (25 mg, 71% yield, [^{13}C]CO_2 = 0.365 mmol); mp 88-90 °C.[22]

^{1}H NMR (400 MHz, CDCl$_3$) δ 7.47 – 7.29 (m, 10H), 5.18 (d, J = 1.8 Hz, 2H).
^{13}C NMR (100 MHz, CDCl$_3$) δ 173.6 (^{13}C labeled), 156.2 (d, J = 7.1 Hz), 131.0 (d, J = 6.4 Hz), 130.7, 130.3 (d, J = 4.0 Hz), 129.4 (d, J = 1.8 Hz, 2C), 129.2 (2C), 129.0, 128.8 (2C), 127.6 (2C), 126.3 (d, J = 65.6 Hz), 70.7 (d, J = 3.2 Hz).
HRMS (ESI) *m/z* calcd for $C_{15}{}^{13}CH_{12}O_2$ $[M+H]^+$: 238.0946; found: 238.0943.
IR (cm^{-1}): 1696, 1441, 1333, 1059, 1025, 954, 772, 693, 644, 501.

(Z)-3-iodo-2,3-diphenylprop-2-en-1-ol

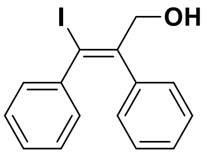

$C_{15}H_{13}IO$
MW: 336.17 g.mol^{-1}
Yield: 56%
White solid

To a solution of propargyl alcohol (1.0 g, 17.8 mmol) and CuI (338 mg, 1.7 mmol) in dry THF (20 mL) was added 3.0 M PhMgBr (15 mL, 44.5 mmol) at -10 °C. Upon complete addition of Grignard reagent, the reaction mixture was allowed to come at room temperature and stirred overnight. The resultant mixture was then cooled to -78 °C and then added to a solution of I_2 (9.0 g, 35.6 mmol) in THF (20 mL). The reaction mixture was allowed to cool at room temperature and stirred for 1 hour then cooling at 0 °C, the reaction mixture was quenched by saturated NH_4Cl. The reaction mixture was warmed up to room temperature and extracted with EtOAc, washed with brine dried over Na_2SO_4 and concentrated under reduced pressure. The crude was purified by flash chromatography 9 :1 Heptane : EtOAc providing the compound as white solid (3.35 g, 56% yield).[23]

^{1}H NMR (400 MHz, CDCl$_3$) δ. 7.10 – 6.92 (m, 10H), 4.68 (d, J = 6.4 Hz, 2H), 1.87 (t, J = 6.5 Hz, 1H).
^{13}C NMR (100 MHz, CDCl$_3$) δ. 146.7, 143.8 8, 137.9, 129.4 (2C), 129.0 (2C), 128.1 (2C), 127.7 (2C), 127.5, 127.2, 102.1, 73.1.

[^{13}C] 2-(diethylamino)ethyl 4-butoxybenzoate ([^{13}C]40)

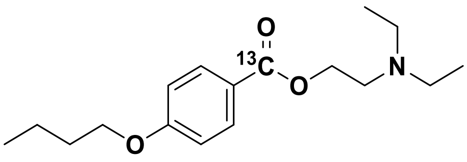

Butoxycaine

$C_{16}{}^{13}CH_{27}NO_3$
MW: 294.40 g.mol^{-1}
Yield: 96%
Orange oil

The [^{13}C] 2-(diethylamino)ethyl 4-butoxybenzoate (**[^{13}C]40**) was prepared accordingly to the **GP2**, using Pd(dba)$_2$ (8.6 mg, 0.015 mmol, 5 mol%), cataCXium® A (10.7 mg, 0.03 mmol, 10 mol%), DMAP (9.1 mg, 0.075 mmol), $NaHCO_3$ (75.6 mg, 0.9 mmol), 1-bromo-4-butoxybenzene (0.051 mL, 0.3 mmol), 2-(diethylamino)ethanol (0.079 mL, 0.6 mmol) under argon. The reaction mixture was heated to 80 °C for 20 hours then the solvent was evaporated under *vacuum*. The crude light brown oil was purified by FC on SiO_2 gel (Eluent EtOAc : MeOH 95 : 5), providing the compound (**[^{13}C]40**) as an orange oil (85 mg, 96% yield).

^{1}H NMR (400 MHz, $CDCl_3$) δ 7.99 – 7.95 (m, 2H), 6.89 (d, J = 8.3 Hz, 2H), 4.36 (td, J = 6.3, 3.0 Hz, 2H), 4.01 (t, J = 6.5 Hz, 2H), 2.84 (t, J = 6.3 Hz, 2H), 2.63 (q, J = 7.1 Hz, 4H), 1.82 – 1.72 (m, 2H), 1.55 – 1.44 (m, 2H), 1.06 (t, J = 7.1 Hz, 6H), 0.98 (t, J = 7.4 Hz, 3H).
^{13}C NMR (100 MHz, $CDCl_3$) δ 166.47 (^{13}C labeled), 163.0, 131.6 (d, J = 2.8 Hz, 2C), 122.5 (d, J = 77.2 Hz), 114.1 (d, J = 4.8 Hz, 2C), 67.9, 63.1 (d, J = 2.3 Hz), 51.1 (d, J = 2.0 Hz), 47.9 (2C), 31.2, 19.3, 13.9, 12.2 (2C).
HRMS (ESI) *m/z* calcd for $C_{16}{}^{13}CH_{27}NO_3$ $[M+H]^+$: 295.2099; found: 295.2097.

[^{13}C] 4-chloro-N-(2-morpholinoethyl)benzamide ([^{13}C]41)

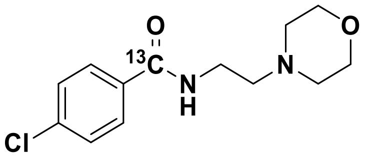

Moclobemide

$C_{12}{}^{13}CH_{17}ClN_2O_2$
MW: 269.73 g.mol^{-1}
Yield: 61%
White solid

The [^{13}C] 4-chloro-*N*-(2-morpholinoethyl)benzamide (**[^{13}C]41**) was prepared accordingly to the **GP1**, using Pd(dba)$_2$ (8.7 mg, 0.015 mmol, 2 mol%), P(*t*Bu)$_3$HBF$_4$ (8.7 mg, 0.030 mmol, 4 mol%), 4-chloroIodobenzene (172 mg, 0.724 mmol), 2-morpholinoethylamine (0.19 mL, 1.45 mmol), and DABCO (162 mg, 1.45 mmol) in dry THF (3.0 mL) under argon. The solution is injected *via* syringe in Chamber 2. The reaction mixture was stirred at room temperature for 48 hours then the solvent was evaporated under *vacuum*. The crude was purified by FC on SiO$_2$ gel (Eluent DCM : MeOH 98:2), providing the compound (**[^{13}C]41**) as white solid (60 mg, 61% yield, [^{13}C]CO$_2$ = 0.365 mmol); mp 136-138 °C

^{1}H NMR (400 MHz, CDCl$_3$) δ 7.76 – 7.66 (m, 2H), 7.46 – 7.36 (m, 2H), 6.76 (s, 1H), 3.76 – 3.67 (m, 4H), 3.58 – 3.49 (m, 2H), 2.64 – 2.56 (m, 2H), 2.54 – 2.43 (m, 4H).
^{13}C NMR (100 MHz, CDCl$_3$) δ 166.4 (^{13}C labeled), 137.7, 133.0 (d, J = 65.1 Hz, 2C), 128.9 (d, J = 4.4 Hz, 2C), 128.4 (d, J = 2.5 Hz), 67.1 (2C), 56.9 (d, J = 1.7 Hz), 53.4 (2C), 36.2.
HRMS (ESI) *m/z* calcd for $C_{12}{}^{13}CH_{17}ClN_2O_2$ [M+H]$^+$: 270.1085; found: 270.1084.
IR (cm^{-1}): 3273, 2972, 2942, 2858, 2810, 1602, 1529, 1294, 1116, 1090, 1008, 864, 838, 724, 681, 525.

[^{13}C]-Probenecid ([^{13}C]42)

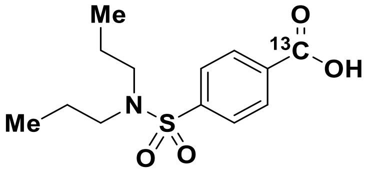

$C_{12}{}^{13}CH_{19}NO_4S$
MW: 286.35 g.mol^{-1}
Yield: 73%
Beige solid

The [^{13}C] Probenecid (**[^{13}C]42**) was prepared accordingly to the general procedure GP6, using $Pd_2(dba)_3$ (9.1 mg, 0.01 mmol, 5 mol%), P(*o*-tol)$_3$ (12.2 mg, 0.04 mmol, 20 mol%), 4-(N,N-dipropylsulfamoyl)benzoyl chloride (60.7 mg, 0.2 mmol), in dry toluene (1.0 mL) under argon. The solution is injected *via* syringe in Chamber 2. The reaction mixture was stirred at 75 °C for 16 hours and quenched using 2 mL of NaOH 1M. A basic work-up is followed by the acidification of the aqueous phase to provide the desired compound (**[^{13}C]42**) as a beige solid (41.8 mg, 73% yield, [^{13}C]CO_2 = 0.365 mmol); m.p. 195-197 °C.

^{1}H NMR (400 MHz, CDCl$_3$) δ 8.26 – 8.21 (m, 2H), 7.94 – 7.86 (m, 2H), 3.15 – 3.09 (m, 4H), 1.62 – 1.50 (m, 4H), 0.87 (t, *J* = 7.4 Hz, 6H).
^{13}C NMR (100 MHz, CDCl$_3$) δ 170.4 (^{13}C labeled), 145.3, 132.6, 131.0 (2C), 127.3 (t, *J* = 2.1 Hz, 2C), 50.1 (2C), 22.1 (2C), 11.3 (2C).
IR (cm^{-1}): 1678, 1344, 1284, 1155, 995, 776, 736, 710, 605, 561.
HRMS (ESI) *m/z* calcd for $C_{12}{}^{13}CH_{19}NO_4S$ $[M+H]^+$ 287.1141; found: 287.1138.
Isotopic Enrichment: +1 ^{13}C, 36.1%.

Other Carbonylations Reactions with Aryl Chlorides and Alkyl Iodide

Besides the reactions of carbonylation reported previously, a practical question was rised on the reactivity of aryl chlorides as starting materials in the carbonylative processes. From the reaction scope, it was indeed noticed that aryl chloride substitutions are tolerated in presence of aryl iodides, which react selectively. See for example compounds **[^{13}C]15** and **[^{13}C]24**. These results seemed to indicate that the ArCl are ineffective substrates.

In order to answer to the question about the reactivity of Ar-Cl in these carbonylative couplings, the following aryl chlorides were used in the amino- and Suzuki- carbonylations. We expressly selected reaction partners that worked effectively from the identical Ar-I derivatives. Under otherwise identical reaction conditions, no formation labeled products was observed.

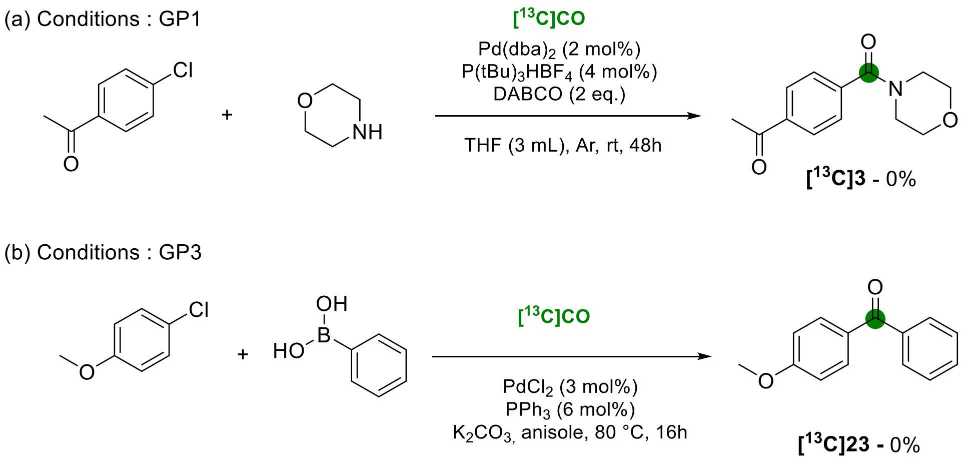

Supplementary Figure 27 Reactions with Aryl Chlorides

A question was raised about the suitability of this method on alkyl halide derivatives. We have thus performed an aminocarbonylation between cyclohexyl iodide and morpholine according to the following report: *J. Org. Chem*. **2019**, *84*, 16076–16085. The procedure was implemented but it was not optimised further. The result highlighted that 18% yield (IS NMR) of the desired labeled amide was formed. This result, which was not optimised further, show that the carbonylation of alkyl substrates is possible, as well.

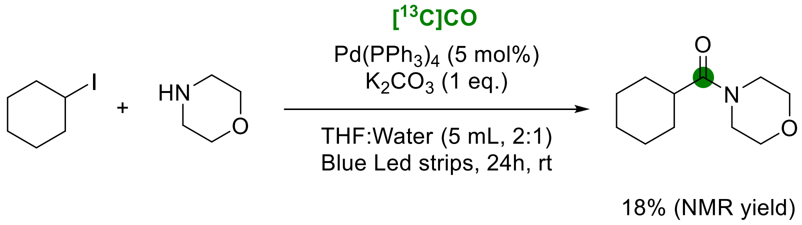

Supplementary Figure 28 Reactions with alkyl iodies

Chamber 1 *(Glassware Type 1)* A suspension of $Ru(bpy)_3Cl_2.6H_2O$ stock solution (1.20 mL), $Re(CO)_3(bpy)Br$ stock solution (0.81 mL), ACN (0.8 mL), BIH (175 mg, 0.78 mmol), phosphine P2 (20.5 mg, 0.036 mmol), phenol (188 mg, 2.0 mmol) were transferred into Chamber 1 with a Pasteur pipette. The chambers were sealed with a screwcap fitted with a Teflon®. The adaptor was then connected to the RC Tritec® system. The solution in Chamber 1 was frozen with a liquid nitrogen bath and the chambers were degassed with vacuum pump connected with RC Tritec manifold for 10 min. The stopcock was closed between the two chambers. $[^{13}C]CO_2$ (365 μmol) was then loaded into Chamber 1 using the RC Tritec® system and the stopcock was closed between Chamber 1 and the adaptor. The loaded Two-Chamber Glassware was then disconnected from the RC Tritec® system and the suspension was warmed to room temperature. Chamber 1 was placed ca. 2 cm away from a 40 W A160WE Tuna Blue Kessil® LED lamp and photo- irradiated with the lower light intensity for 1 h. The $[^{13}C]CO$ produced is then used in the carbonylation reaction in Chamber 2.

Chamber 2 *(Glassware Type 1)* To chamber A were added $Pd(PPh_3)_4$ (5%, 0.03 mmol), K_2CO_3, (1 equiv, 0.60 mmol), THF (2 mL), iodocyclohexane (1 equiv, 0.60 mmol), morpholine (3 equiv, 1.80 mmol), and water (1 mL). The solution is then transferred *via* syringe (previously removing the argon

from the syringe) into Chamber 2 of the TwoChamber Glassware (note: this chamber is under vacuum). Stopcock between Chamber 1 (where [^{13}C]CO is previously produced by photoreduction following GP0, page S40) and Chamber 2 is then open. The solution is stirred for 48 h at room temperature under [^{13}C]CO atmosphere under Blue LED strips irradiation. The [^{13}C]CO is then evacuated from the Two-Chamber Glassware, the solution transferred into a round bottom flask and the solvent removed under reduced pressure. [^{13}C]-cyclohexyl(morpholino)methanone was obtained with 18% NMR yield. Yields was calculated on the [^{13}C]CO_2 loaded in Chamber 1 (limiting reagent, 0.365 mmol).

^{14}C Labeling of Pharmaceutically Relevant Compounds

[^{14}C] N-hexyl-4-methoxybenzamide ([^{14}C]1)

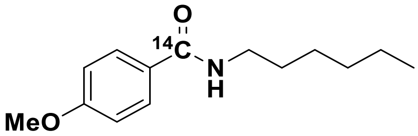

$C_{13}{}^{14}CH_{21}NO_2$
MW: 237.32 g.mol^{-1}

Glassware Type 1 (Figure S1) was used.

<u>*Photoreduction*</u> : A suspension of $Ru(bpy)_3Cl_2.6H_2O$ stock solution (1.20 mL), $Re(CO)_3(bpy)Br$ stock solution (0.81 mL), ACN (0.8 mL), BIH (194 mg, 0.78 mmol), phosphine P**2** (20.5 mg, 0.036 mmol), phenol (188 mg, 2.0 mmol) were transferred into Chamber 1 with a Pasteur pipette. The chambers were sealed with a screwcap fitted with a Teflon®. The adaptor was then connected to the ^{14}C Tritec® system. The solution in Chamber 1 was frozen with a liquid nitrogen bath and the chambers were degassed with *vacuum* pump connected with Tritec manifold for 10 min. The stopcock was closed between the two chambers. **[^{14}C]CO$_2$** (0.35 mmol, 764.79 MBq, **A$_m$**: 2171.9 MBq mmol^{-1}) was then loaded into Chamber 1 using the RC Tritec® system and the stopcock was closed between Chamber 1 and the adaptor. The loaded Two-Chamber Glassware was then disconnected from the RC Tritec® system and the suspension was warmed to room temperature. Chamber 1 was placed *ca.* 2 cm away from a 40 W A160WE Tuna Blue Kessil® LED lamp and photo-irradiated with the lower light intensity for 1 h. The **[^{14}C]CO** produced is then used in the carbonylation reaction in Chamber 2.

<u>*Carbonylation*</u>: [^{14}C] *N*-hexyl-4-methoxybenzamide (**[^{14}C]1**) was prepared accordingly to the **GP1**, using $Pd(dba)_2$ (8.7 mg, 0.015 mmol, 2 mol%), $P(tBu)_3HBF_4$ (8.7 mg, 0.030 mmol, 4 mol%), 4-iodoanisole (169 mg, 0.724 mmol), *n*-hexylamine (0.19 mL, 1.45 mmol), and DABCO (162 mg, 1.45 mmol) in dry THF (3.0 mL) under argon. The solution is injected *via* syringe in Chamber 2. The reaction mixture was stirred at room temperature for 48 hours then the solvent was evaporated under *vacuum*. The crude was purified by FC on SiO_2 gel (Eluent Hept : EtOAc 60:40), providing the compound (**[^{14}C]1**) (46% RCY, 0.162 mmol, 337.25 MBq).

Molar activity (MS (ESI)): 2077.92 MBq mmol^{-1}

TLC (silicagel 60F254, Heptane/ EtOAc (60/40)); **Radiochemical purity**: >99%

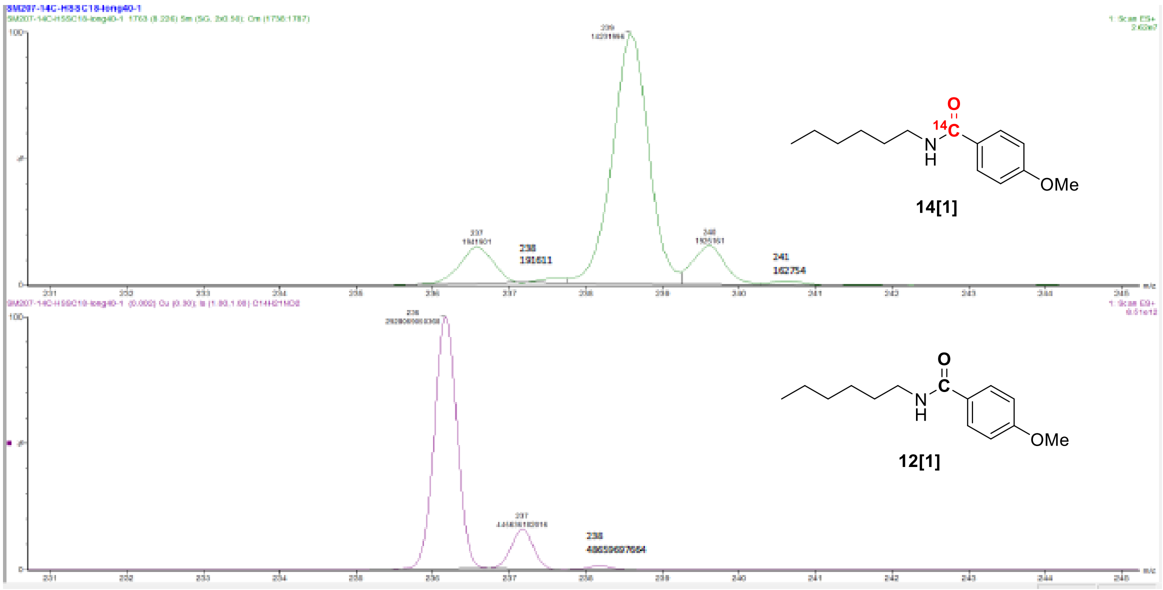

Mesure SM207-14C-Purifié_02.rta raytest GmbH Page 1/1

C:\PROGRA-1\raytest\Rita Control\list\OLIVIER\C14\SM207-14C-PURIFIé_02.RTADate d'impression : 1

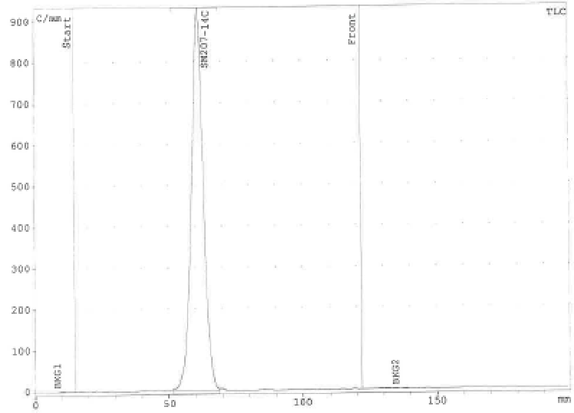

Description de l'échantillon

Etude: OLIVIER
Mesure: SM207-14C-Purifié_02.rta, commencé: 17/10/2019 11:20
Méthode: C14
Origine: 15 mm Front 122 mm
Meas. time: 0,1 min Résolution: 0,4 mm
Tray number: 1,0 Position de scan: 175,0 mm
Haute tension: 1620,0 V

SM207-14C purifié
Silicagel Merck 60F254 - Heptane 60 AcOEt 40
Détecteur de radioactivité: raytest RITA
Autre Square flow cell #0
Cell volume 0 ul

Intégration TLC

Substance	R/F	Type	Aire	%Aire
			Counts	%
SM207-14C	0,432	DD	4786,499	100,00
Sum in ROI			4786,499	
Aire totale			4775,831	
Aire RF			4788,765	
BKG1			0,1136	
BKG2			0,3209	
Remainder RF			2,27	0,05
Remainder (Tot)			-10,67	-0,22

Supplementary Figure 29 MS (ESI) and radio-TLC for compound **[^{14}C]1**

[^{14}C] 2-(diethylamino)ethyl 4-butoxybenzoate ([^{14}C]40)

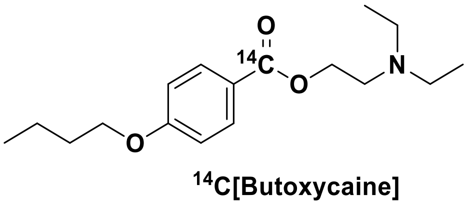

$C_{16}{}^{14}CH_{27}NO_3$
MW: 295.40 g.mol^{-1}

Glassware Type 1 (Figure S1) was used.

Photoreduction: A suspension of $Ru(bpy)_3Cl_2.6H_2O$ stock solution (1.20 mL), $Re(CO)_3(bpy)Br$ stock solution (0.81 mL), ACN (0.8 mL), BIH (194 mg, 0.78 mmol), phosphine P**2** (20.5 mg, 0.036 mmol), phenol (188 mg, 2.0 mmol) were transferred into Chamber 1 with a Pasteur pipette. The chambers were sealed with a screwcap fitted with a Teflon®. The adaptor was then connected to the 14C Tritec® system. The solution in Chamber 1 was frozen with a liquid nitrogen bath and the chambers were degassed with *vacuum* pump connected with Tritec manifold for 10 min. The stopcock was closed between the two chambers. **[^{14}C]CO_2** (0.35 mmol, 754.06 MBq, **A_m**: 2171.9 MBq mmol^{-1}) was then loaded into Chamber 1 using the Tritec® system and the stopcock was closed between Chamber 1 and the adaptor. The loaded Two-Chamber Glassware was then disconnected from the Tritec® system and the suspension was warmed to room temperature. Chamber 1 was placed *ca.* 2 cm away from a 40 W A160WE Tuna Blue Kessil® LED lamp and photo-irradiated with the lower light intensity for 1 h. The **[^{14}C]CO** produced is then used in the carbonylation reaction in Chamber 2.

Carbonylation: [^{14}C] 2-(diethylamino)ethyl 4-butoxybenzoate (**[^{14}C]40**) was prepared accordingly to the **GP2**, using $Pd(dba)_2$ (8.6 mg, 0.015 mmol), cataCXium® A (10.7 mg, 0.03 mmol), DMAP (9.1 mg , 0.075 mmol), $NaHCO_3$ (75.6 mg, 0.9 mmol), 1-bromo-4-butoxybenzene (0.051 mL, 0.3 mmol), 2-(diethylamino)ethanol (0.079 mL, 0.6 mmol) under Argon. The chamber was heated to 80 °C for 20 hours then the solvent was evaporated under *vacuum*. The crude light brown oil was purified by FC on SiO_2 gel (Eluent DCM : MeOH 90 : 10), providing the compound **[^{14}C]40** (40%, 0.120 mmol, 243.09 MBq).

Molar activity (MS (ESI)): 2020.2 MBq mmol^{-1}

TLC (silicagel 60F254, DCM / MeOH (90/ 10)); **Radiochemical purity**: >99%

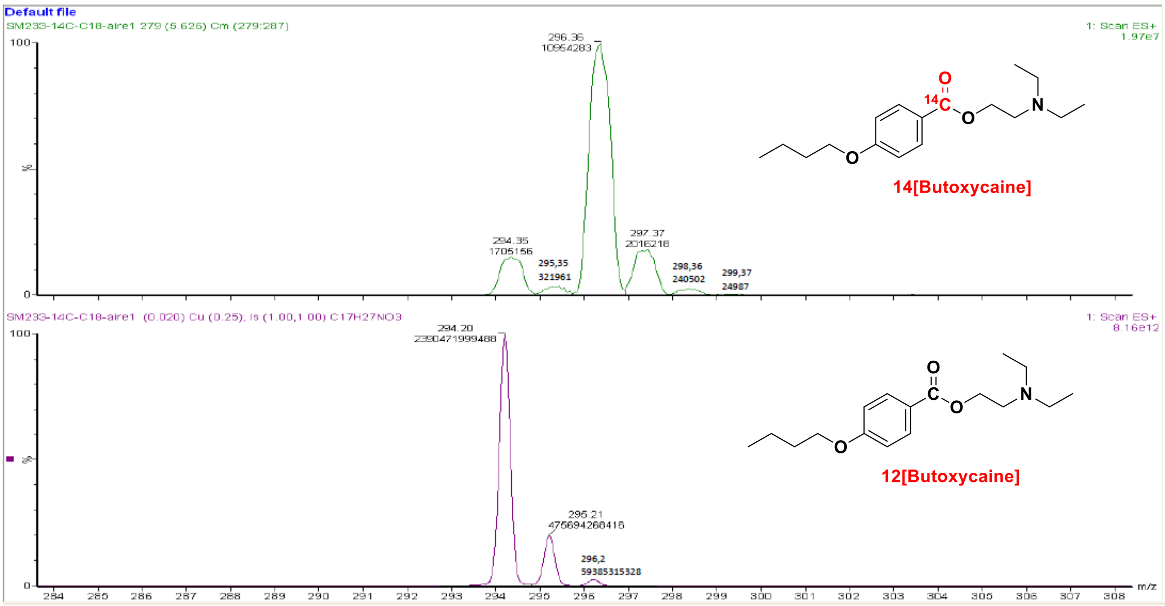

Supplementary Figure 30 MS (ESI) for compound **[^{14}C]40**

Mesure SM233-14C purA.rta raytest GmbH Page 1/1
C:\PROGRA~1\raytest\Rita Control\list\OLIVIER\C14\SM233-14C PURA.RTADate d'impression : 10/12/2[illegible]

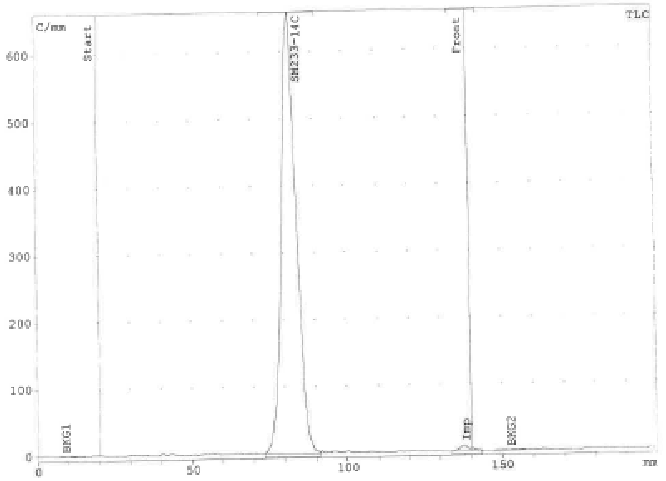

Description de l'échantillon

Etude:	OLIVIER	
Mesure:	SM233-14C purA.rta, commencé:	10/12/2019 10:37
Méthode:	C14	
Origine:	20 mm	Front 140 mm
Meas. time:	0,3 min	Résolution: 0,4 mm
Haute tension:	1620,0 V	

SM233-14C- purifié
Silicagel Merck 60F254 - CH2Cl2 90 MeOH 10
Détecteur de radioactivité: raytest RITA
Autre Square flow cell #0
Cell volume 0 ul

Intégration TLC

Substance	R/F	Type	Aire	%Aire
			Counts	%
SM233-14C	0,520	DD	3625,392	99,21
Imp	0,982	DD	28,958	0,79
Sum in ROI			3654,350	
Aire totale			3691,797	
Aire RF			3674,529	
BKG1			0,3030	
BKG2			0,3209	
Remainder RF			20,18	0,55
Remainder (Tot)			37,45	1,01

Supplementary Figure 31 radio-TLC for compound **[^{14}C]40**

[^{14}C]- (3-fluoro-4-methoxyphenyl)(3,4,5-trimethoxyphenyl)methanone ([^{14}C]37)

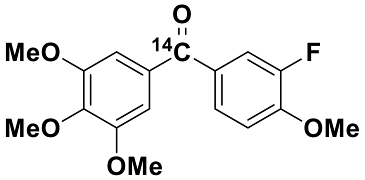

$C_{16}{}^{14}CH_{17}FO_5$
MW: 322.31 g.mol^{-1}

Glassware Type 1 (Figure S1) was used.

<u>Photoreduction</u>: A suspension of $Ru(bpy)_3Cl_2.6H_2O$ stock solution (1.20 mL), $Re(CO)_3(bpy)Br$ stock solution (0.81 mL), ACN (0.8 mL), BIH (194 mg, 0.78 mmol), phosphine P**2** (20.5 mg, 0.036 mmol), phenol (188 mg, 2.0 mmol) were transferred into Chamber 1 with a Pasteur pipette. The chambers were sealed with a screwcap fitted with a Teflon®. The adaptor was then connected to the 14C Tritec® system. The solution in Chamber 1 was frozen with a liquid nitrogen bath and the chambers were degassed with *vacuum* pump connected with Tritec manifold for 10 min. The stopcock was closed between the two chambers. **[^{14}C]CO_2** (0.35 mmol, 768.63 MBq, **A_m**: 2171.9 MBq.mmol^{-1}) was then loaded into Chamber 1 using the Tritec® system and the stopcock was closed between Chamber 1 and the adaptor. The loaded Two-Chamber Glassware was then disconnected from the Tritec® system and the suspension was warmed to room temperature. Chamber 1 was placed *ca.* 2 cm away from a 40 W A160WE Tuna Blue Kessil® LED lamp and photo-irradiated with the lower light intensity for 1 h. The **[^{14}C]CO** produced is then used in the carbonylation reaction in Chamber 2.

<u>Carbonylation</u>: [^{14}C] (3-fluoro-4-methoxyphenyl)(3,4,5-trimethoxyphenyl)methanone was prepared accordingly to the **GP3**, using $PdCl_2$ (1.06 mg, 0.006 mmol), PPh_3 (3.1 mg, 0.012 mmol), K_2CO_3 (83 mg, 0.6 mmol), 5-iodo-1,2,3-trimethoxybenzene (58.8 mg, 0.2 mmol), (3-fluoro-4-methoxyphenyl)boronic acid (51 mg, 0.3 mmol) in dry anisole (1.6 mL) under Argon. The chamber was stirred at 80 °C for 18 hours then the solvent was evaporated under *vacuum*. The crude was purified by FC on SiO_2 gel (Eluent Hept:EtOAc 80:20), providing the compound as a light yellow solid (180.39 MBq, 0.090 mmol, 45% yield calculated on the limiting reagent, (1.77 equiv of [^{14}C]CO_2)).

Molar activity (MS (ESI)): 2015.58 MBq.mmol^{-1}.

Radiochemical purity (TLC): >99% (Silicagel 60F254, Heptane/AcOEt (70/ 30)).

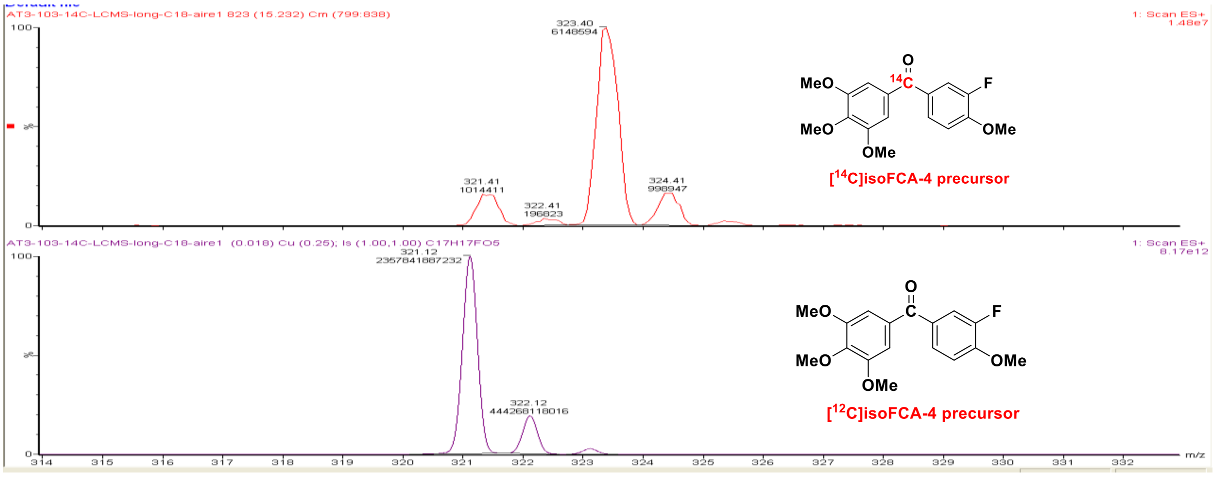

Mesure AT3-103 14C Pur_02.rta raytest GmbH Page 1/1
C:\PROGRA~1\raytest\Rita Control\list\OLIVIER\C14\AT3-103 14C PUR_02.RTADate d'impression : 19/

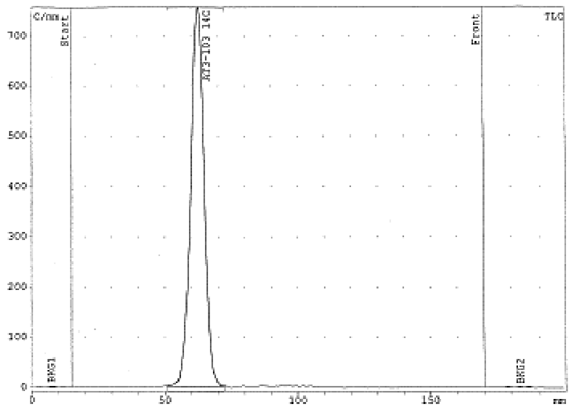

Description de l'échantillon

Etude:	OLIVIER		
Mesure:	AT3-103 14C Pur_02.rta, commencé:	29/09/2020	16:28
Méthode:	C14 de:	01/01/2000	
Origine:	15 mm	Front 170 mm	
Meas. time:	0,3 min	Résolution: 0,4 mm	

AT3-103 14C Pur
Silicagel Merck 60F254 - Heptane 70 AcOEt 30
Détecteur de radioactivité: raytest RITA
Autre Square flow cell #0
Cell volume 0 ul

Intégration TLC

Substance	R/F	Type	Aire	%Aire
			Counts	%
AT3-103 14C	0,307	DD	4431,619	100,00
Sum in ROI			4431,619	
Aire totale			4509,103	
Aire RF			4506,448	
BKG1			0,7524	
BKG2			0,6583	
Remainder RF			74,83	1,66
Remainder (Tot)			77,48	1,72

Supplementary Figure 32 MS (ESI) and radio-TLC for compound **[^{14}C]37**

Radiochemical purity (HPLC): >99% (Column: Xbridge C18 (100 x 4.6 mm) 3.5 µm, Flow: 1 mL/mn, Temperature: 20°C, Solvent A: H_2O with 0.1% HCOOH, Solvent B: CH_3CN with 0.1% HCOOH).

	Solvent A (%)	Solvent B (%)
t = 0	95	5
t = 24 mn	0	100
t = 30 mn	0	100

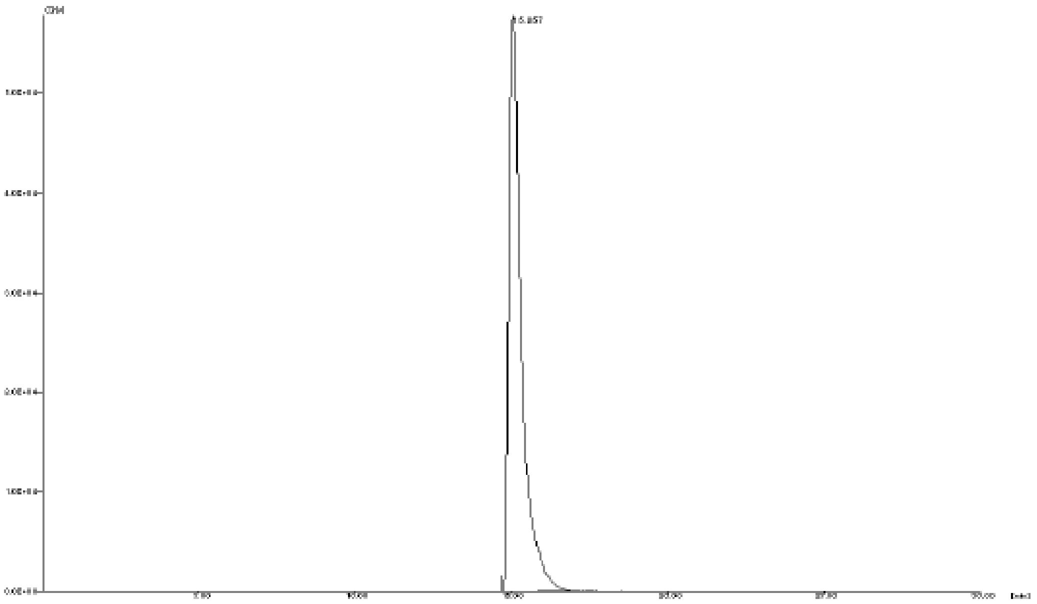

#	Nom	Tr[min]	Aire[CPM.Sec	%Aire
	1	15.057	1520670	100.0

Aire Totale des Pics = 1520670 [CPM.Sec]

Supplementary Figure 33 HPLC for compound **[^{14}C]37**

[^{14}C]-Probenecid ([^{14}C]42)

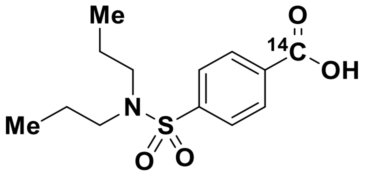

$C_{12}{}^{13}CH_{19}NO_4S$
MW: 287.35 g.mol^{-1}

Photoreduction : A suspension of $Ru(bpy)_3Cl_2.6H_2O$ stock solution (1.20 mL), $Re(CO)_3(bpy)Br$ stock solution (0.81 mL), ACN (0.8 mL), BIH (194 mg, 0.78 mmol), phosphine P**2** (20.5 mg, 0.036 mmol), phenol (188 mg, 2.0 mmol) were transferred into Chamber 1 with a Pasteur pipette. The chambers were sealed with a screwcap fitted with a Teflon®. The adaptor was then connected to the 14C Tritec® system. The solution in Chamber 1 was frozen with liquid nitrogen bath and the chambers were degassed with *vacuum* pump connected with Tritec manifold for 10 min. The stopcock was closed between the two chambers. **[^{14}C]CO_2** (0.36 mmol, 772.79 MBq, **A_m**: 2171.9 MBq.mmol^{-1}) was then loaded into Chamber 1 using the Tritec® system and the stopcock was closed between chamber 1 and the adaptor. The loaded Two-Chamber Glassware was then disconnected from the Tritec® system and the suspension was warmed to room temperature. Chamber 1 was placed *ca.* 2 cm away from a 40 W A160WE Tuna Blue Kessil® LED lamp and photo-irradiated with the lower light intensity for 1 h. The **^{14}CO** produced is then used in the exchange reaction in Chamber 2.

Carboxylation exchange: The [^{14}C] Probenecid (**[^{14}C]42**) was prepared accordingly to the general procedure, using $Pd_2(dba)_3$ (9.1 mg, 0.01 mmol), P(*o*-tol)$_3$ (12.2 mg, 0.04 mmol), 4-(N,N-dipropylsulfamoyl)benzoyl chloride (60.7 mg, 0.2 mmol), in dry toluene (1.0 mL) under Argon. The solution is injected *via* syringe in Chamber 2. The chamber was stirred at 75 °C for 16 hours before being quenched using 2 mL of NaOH 1M, a basic work-up followed by the acidification of the aqueous phase provides the compound (**[^{14}C]42**) as a beige solid (89.98 MBq, 0.146 mmol, 73% yield calculated on the limiting reagent (1.78 equiv of [^{14}C]CO_2)).

Molar activity (MS (ESI)): 616.45 MBq.mmol^{-1}.

Radiochemical purity (TLC): >99% (Silicagel 60F254, CH_2Cl_2/MeOH/AcOH (100/3/0.2).

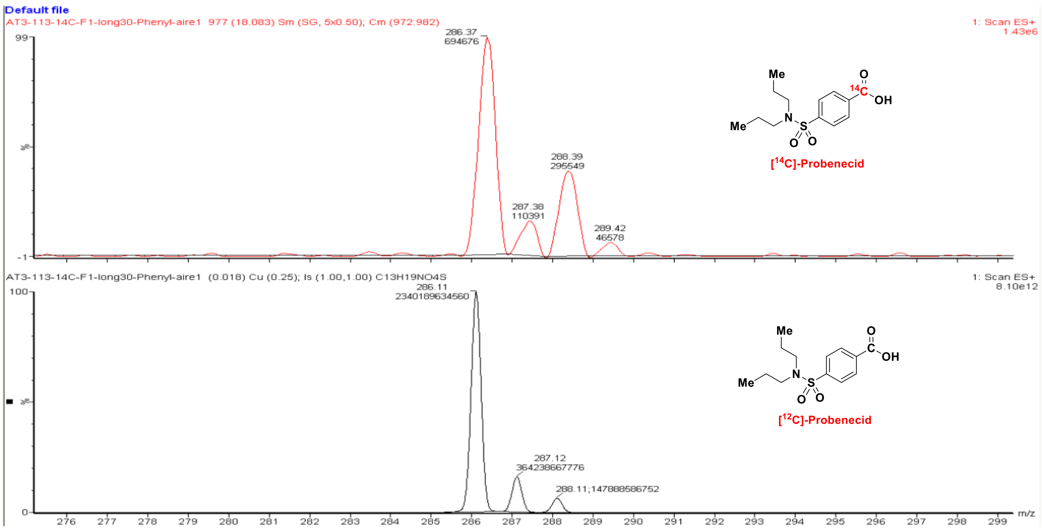

	M+1	M+2 ($1^{13}C$)	M+3 ($2^{13}C$)	M+4 ($3^{13}C$)	M+5 ($4^{13}C$)
			M+3 ($1^{14}C$)		
	286	287	288	289	
aire tot	694676	110391	295549	46578	
Σ contrib	0	108123	44253	39256	
aire D	0	2268	251296	7322	
% D	0	0,2%	26,7%	0,8%	
Activité spécifique			26,7%		

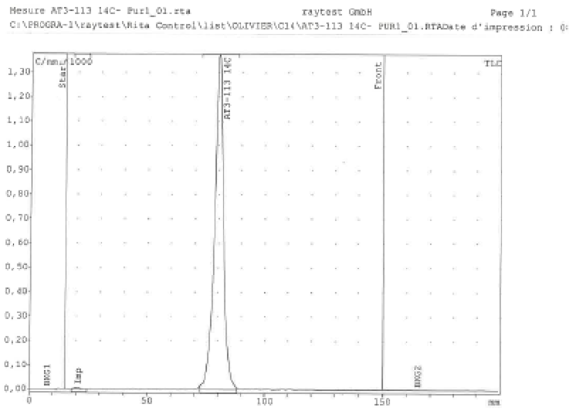

Description de l'échantillon

Etude: OLIVIER
Mesure: AT3-113 14C- Puri_01.rta, commencé: 08/10/2020 14:04
Méthode: C14 de: 01/01/2000
Origine: 15 mm Front 150 mm
Meas. time: 0.2 min Résolution: 0,4 mm

AT3-113 14C
Silicagel Merck 60F254
CH2Cl2 100 MeOH 3 AcOH 0.2
Détecteur de radioactivité: raytest RITA
Autre Square flow cell #0
Cell volume 0 ul

Intégration TLC

Substance	R/F	Type	Aire Counts	%Aire %
Imp	0,038	DD	20,917	0,34
AT3-113 14C	0,484	DD	6176,146	99,66
Sum in ROI			6197,063	
Aire totale			6317,209	
Aire RF			6308,667	
BKG1			0,5051	
BKG2			0,0826	
Remainder RF			111,60	1,77
Remainder (Tot)			120,15	1,90

Supplementary Figure 34 MS (ESI) and radio-TLC for compound **[^{14}C]42**

Radiochemical purity (HPLC): >99% (Column: Xbridge C18 (100 x 4.6 mm) 3.5 µm, Flow: 1 mL/mn, Temperature: 20°C, Solvent A: H_2O with 0.1% HCOOH, Solvent B: CH_3CN with 0.1% HCOOH).

	Solvent A (%)	Solvent B (%)
t = 0	95	5
t = 25 mn	0	100
t = 30 mn	0	100

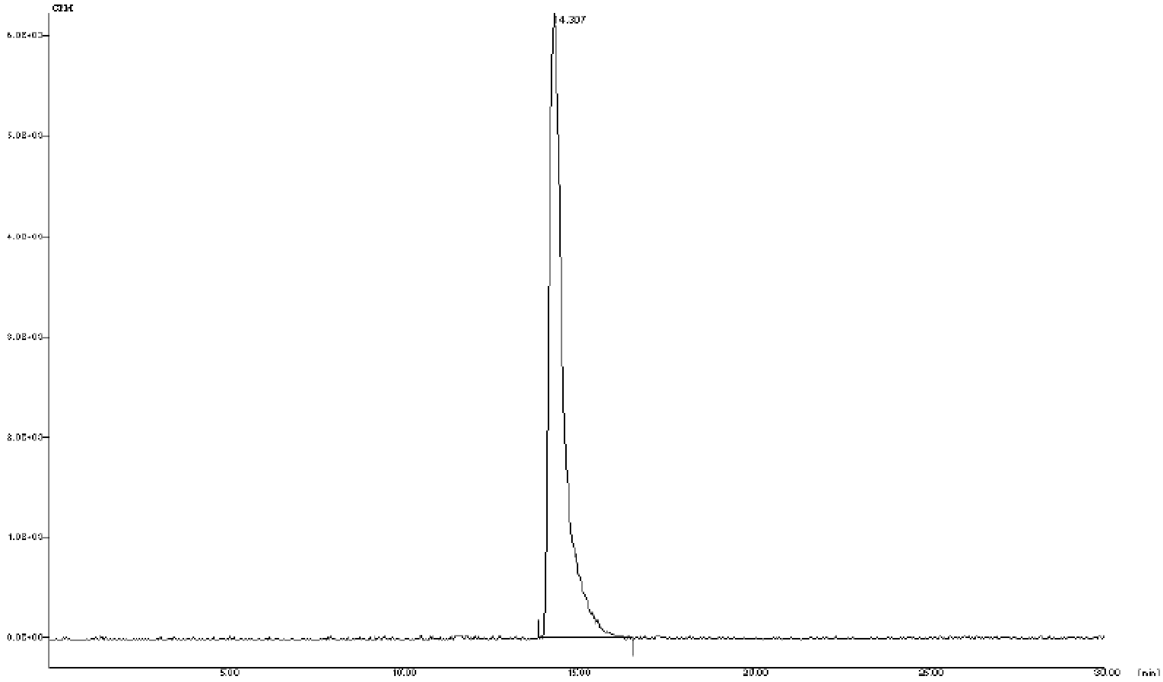

#	Nom	Tr[min]	Aire[CPM.Sec	%Aire
	1	14.307	161831	100.0

Aire Totale des Pics = 161831 [CPM.Sec]

Supplementary Figure 35 HPLC for compound **[^{14}C]42**

^{11}C Radio-synthesis of amide **[^{11}C]1**

Automated radiosynthesis with carbon-11 was performed using a MeI_{plus} research synthesizer (Synthra GmbH, Germany) with modifications to undergo direct bubbling of [^{11}C]CO_2 into the reaction vessel (Figure S27). No carrier-added [^{11}C]CO_2 (1.8-3.5 GBq) was produced *via* the ^{14}N(p, α)^{11}C nuclear reaction by irradiation of a [^{14}N]N_2 target containing 0.15-0.5% of O_2 on a cyclone 18/9 cyclotron (18 MeV, IBA, Belgium) and trapped at -180 °C. [^{11}C]CO_2 was then released at 0 °C under a stream of helium (8 mL/min) to bubble for 5 s into reactor 1 containing a solution of phenol (1.9 mg), BIH (1.8 mg), tri-*tert*-butyl phosphonium tetrafluoroborate (0.2 mg), [(bpm)Re$(CO)_3$Br] (8 µg) and [Ru$(bpm)_3(PF_6)_2$] (12 µg) in CH_3CN (400 µL) at -50 °C. The mixture was heated at 20 °C and submitted at irradiation under blue light (Kessil A160WE Tuna Blue, LED lamp® (40W)) for 5 min. By pressurizing reactor 1 with helium (2 mL/min), the generated [^{11}C]$CO_{(g)}$ was passed through an Ascarite® column to remove unreacted [^{11}C]CO_2 and bubbled in reactor 2 containing a solution of iodobenzene (5 µL), hexylamine (200 µL), Xantphos (16 mg) and $Pd_2(dba)_3$ (14 mg) in THF (2 mL). The mixture was heated at 70 °C for 2 min. Upon cooling to room temperature, the mixture was diluted with CH_3CN/H_2O (1 mL, 50/50 v/v) to afford **[^{11}C]1** in 21% RCC and 88% RCP.

Analysis of the crude was performed by HPLC using a 717_{plus} Autosampler system equipped with a 1525 binary pump and a 2996 photodiode array detector (Waters, USA) and a Flowstar LB 513 (Berthold, France) gamma detector. The system was monitored with the Empower 3 (Waters, USA) software. HPLC were realized on a reverse phase analytical Symmetry C18 (50 x 3.9 mm, 5 µm, Waters, USA) column using a mixture of H_2O/CH_3CN/PicB7® (2 mL/min) as eluent. UV detection was performed at 252 nm. Identification of the peak was assessed by comparing the retention time of [^{11}C]SM-108 with the retention time of the non-radioactive reference (t_R^{ref}). For acceptance, the retention time must be within the t_R^{ref} ± 10% range. Radiochemical purity (RCP) was calculated as the ratio of the area under the curve (AUC) of the peak over the sum of the AUCs of all other peaks on gamma chromatograms. Radiochemical purity is the mean value of three consecutive runs. The radiochemical conversion (RCC) of the labeling reaction was calculated as the ratio of the decay-corrected activity at

the end of the synthesis (A_{EOS}), measured in an ionization chamber (Capintec®, Berthold, France) over the starting activity of [^{11}C]CO_2 (A_{CO2}) measured by the calibrated detector of the synthesizer. This ratio was corrected for the radiochemical purity following the equation: RCC = (A_{EOS} / A_{CO2}) x RCP. Molar activity was calculated as the ratio of the activity of the collected peak of [^{11}C]**1** measured in an ionization chamber (Capintec®, Berthold) over the molar quantity of **1** determined using a calibration curve. Molar activity is calculated as the mean value of three consecutive runs.

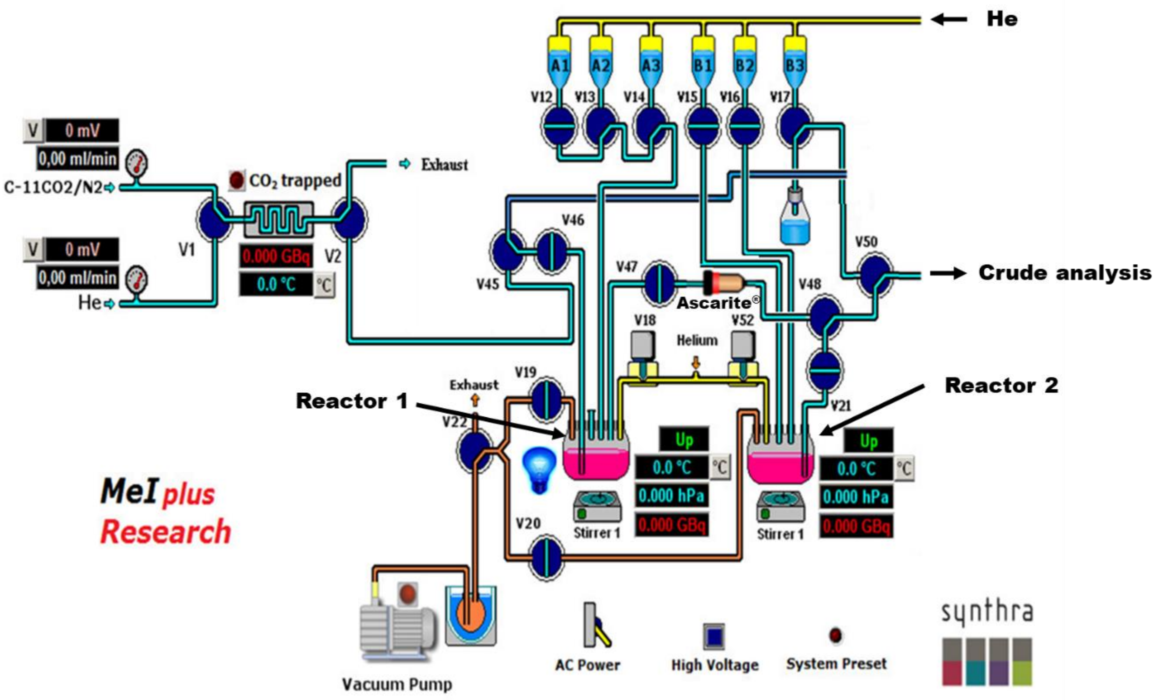

Supplementary Figure 36. Modified MeI_{plus} Research module for photocarbonylation.

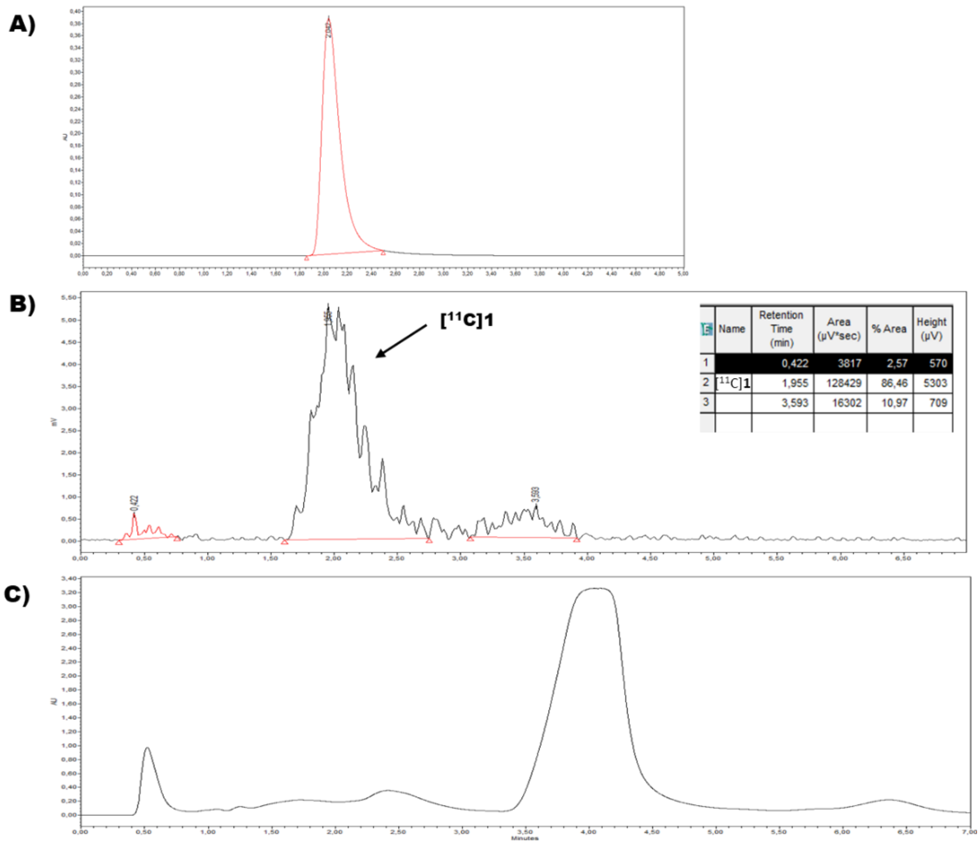

Supplementary Figure 37. Reverse phase analytical HPLC of crude [^{11}C]**1**. A) UV chromatogram (λ = 252 nm) of the reference compound **1**; B) Gamma chromatogram of crude [^{11}C]**1**; C) UV (λ = 252 nm) chromatogram of crude [^{11}C]**1**.

NMR Spectra

[^{13}C] N-hexyl-4-methoxybenzamide ([^{13}C]1)

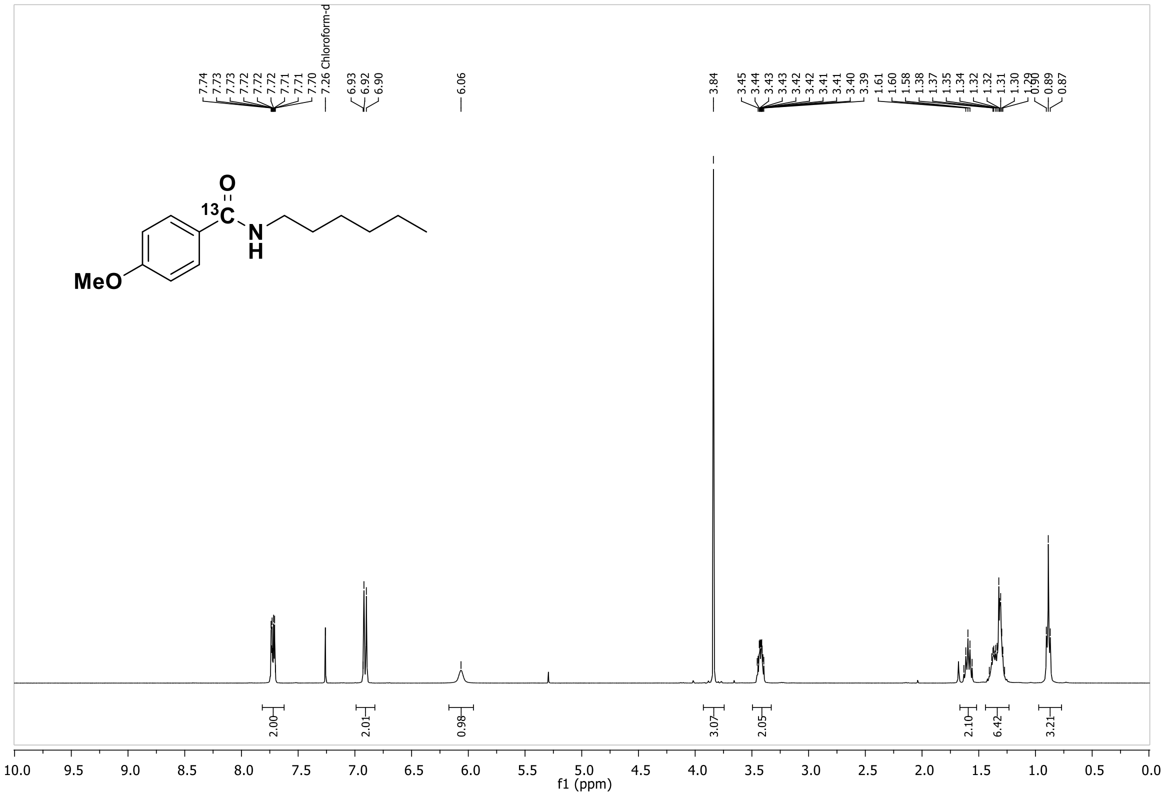

Supplementary Figure 38. ^{1}H NMR Spectrum (400 MHz, $CDCl_3$) of compound **[^{13}C]1**.

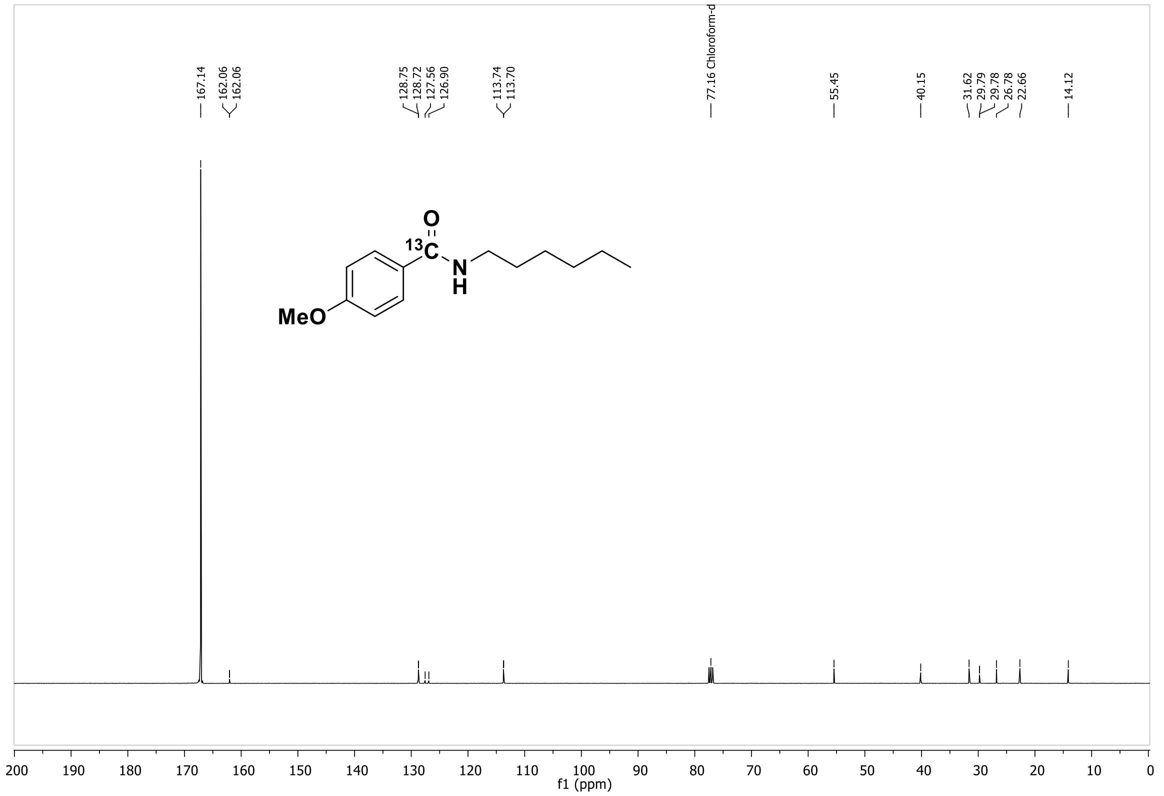

Supplementary Figure 39. ^{13}C NMR Spectrum (100 MHz, $CDCl_3$) of compound **[^{13}C]1**.

[^{13}C] N-benzylnicotinamide ([^{13}C]2)

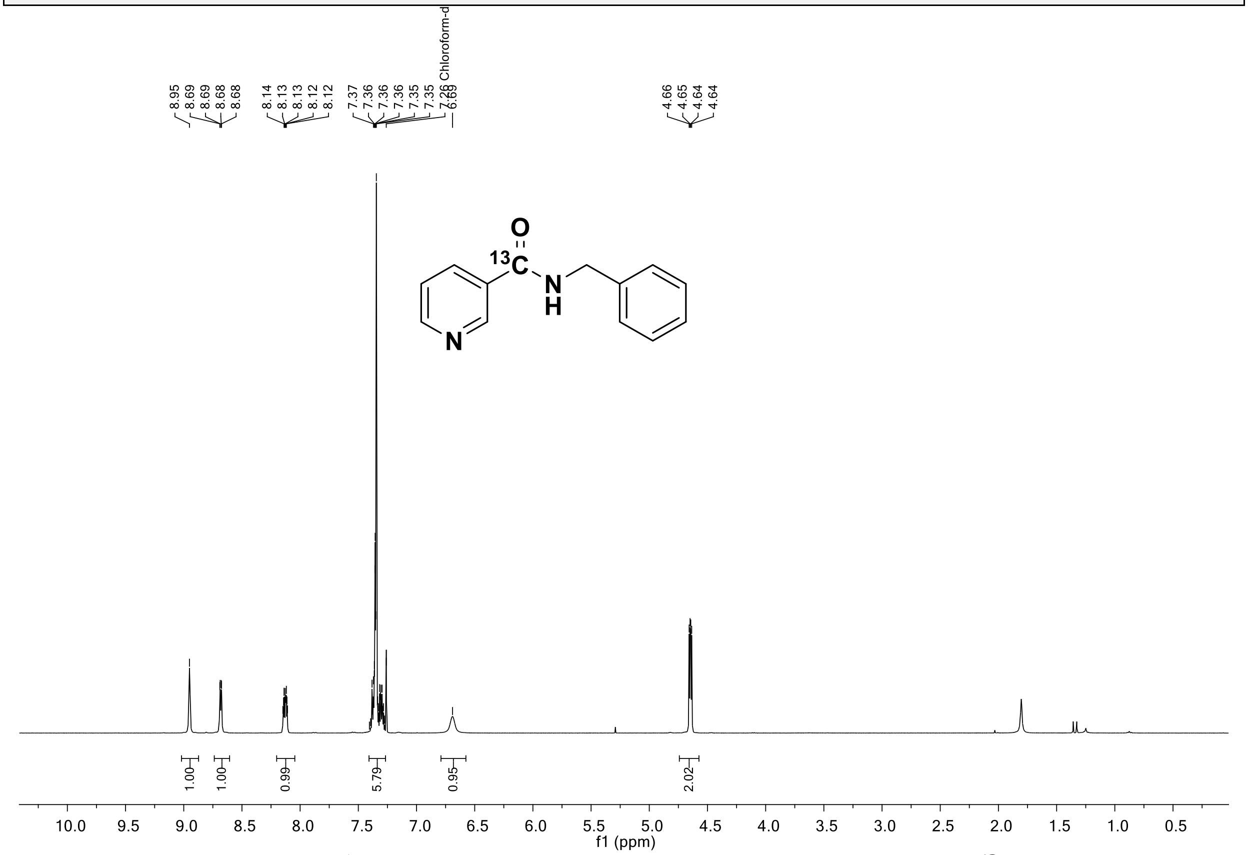

Supplementary Figure 40. ^{1}H NMR Spectrum (400 MHz, $CDCl_3$) of compound **[^{13}C]2**.

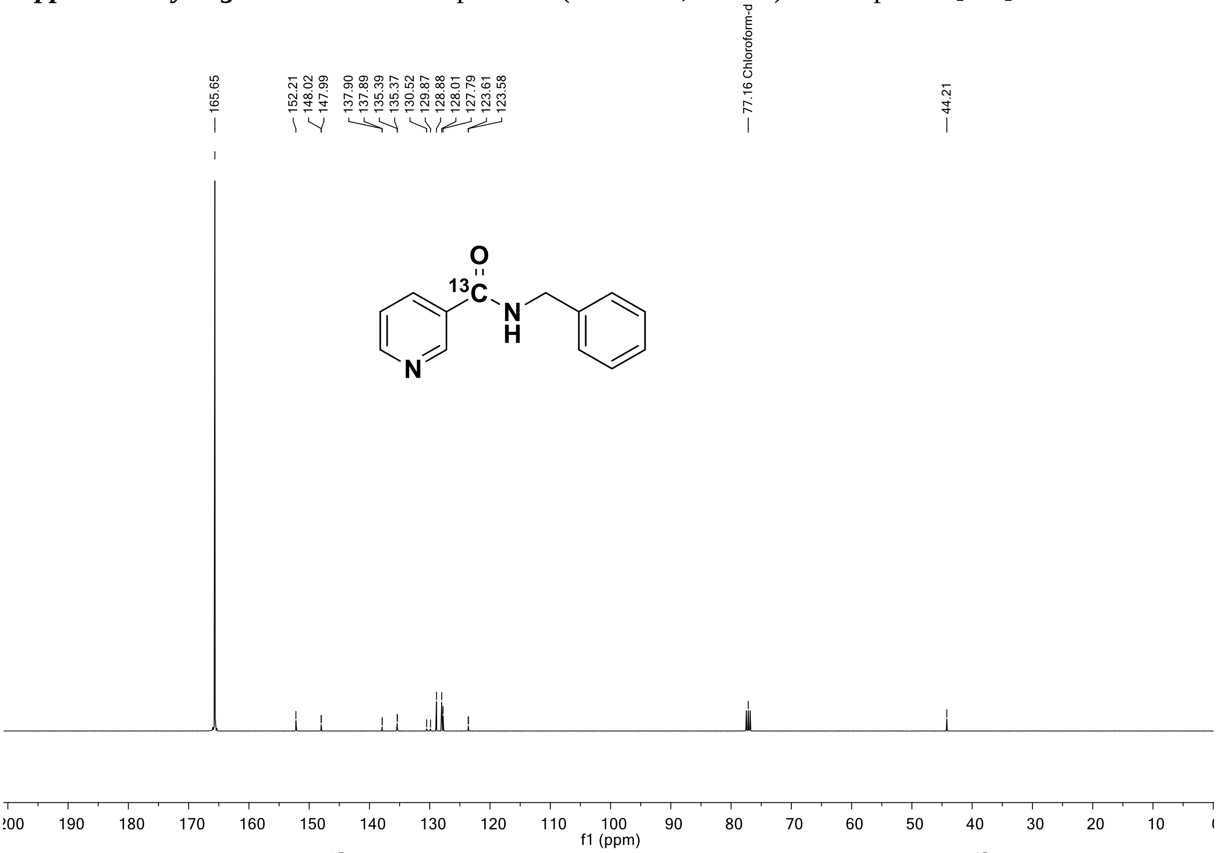

Supplementary Figure 41. ^{13}C NMR Spectrum (100 MHz, $CDCl_3$) of compound **[^{13}C]2**.

[^{13}C] 1-(4-(morpholine-4-carbonyl)phenyl)ethan-1-one ([^{13}C]3)

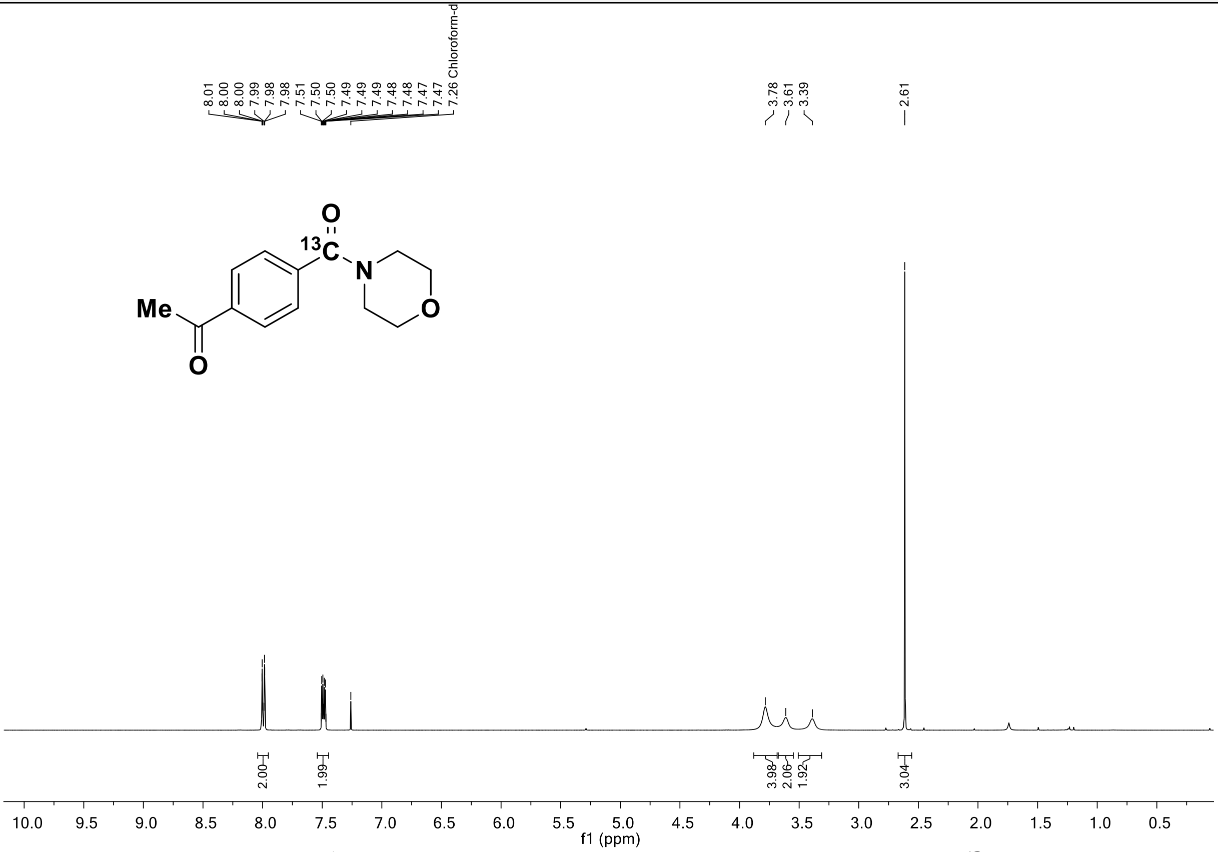

Supplementary Figure 42. ^{1}H NMR Spectrum (400 MHz, $CDCl_3$) of compound **[^{13}C]3**.

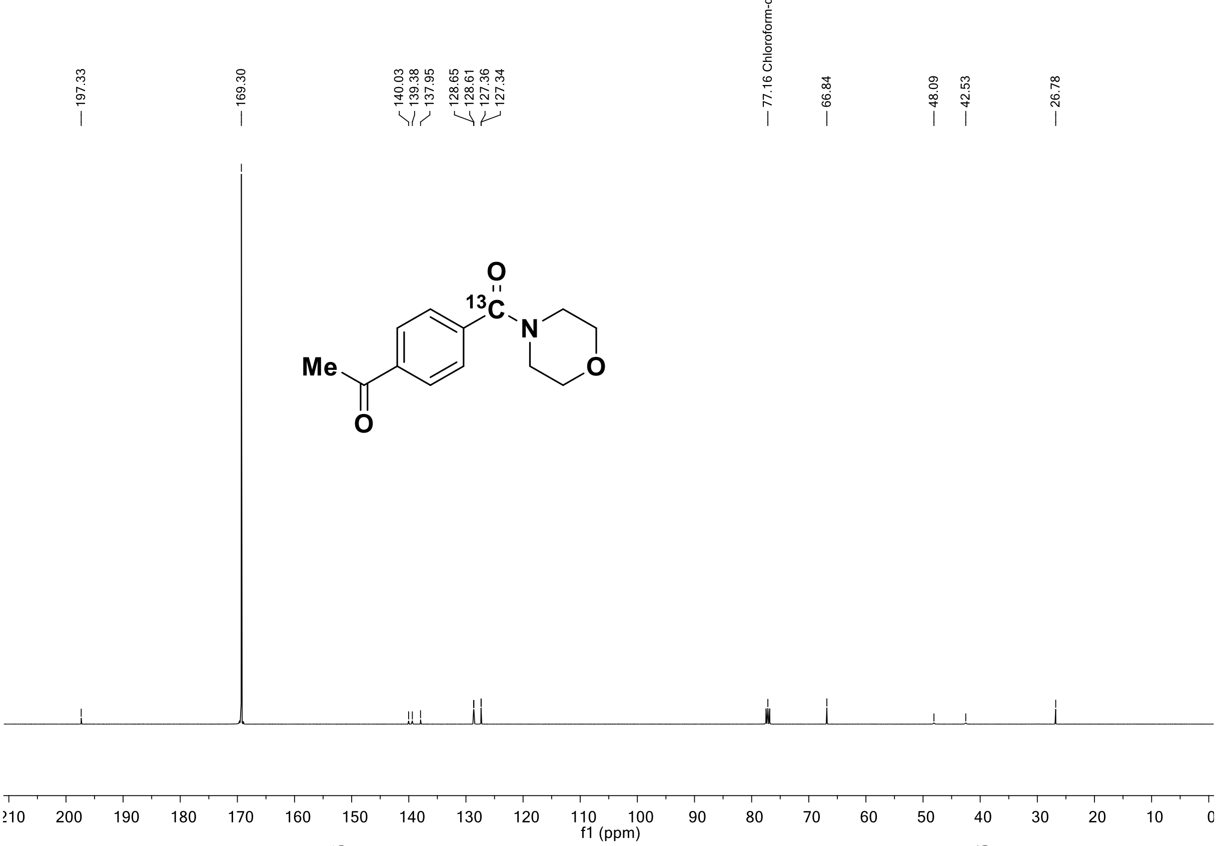

Supplementary Figure 43. ^{13}C NMR Spectrum (100 MHz, $CDCl_3$) of compound **[^{13}C]3**.

[^{13}C] N-cyclohexyl-4-fluorobenzamide ([^{13}C]4)

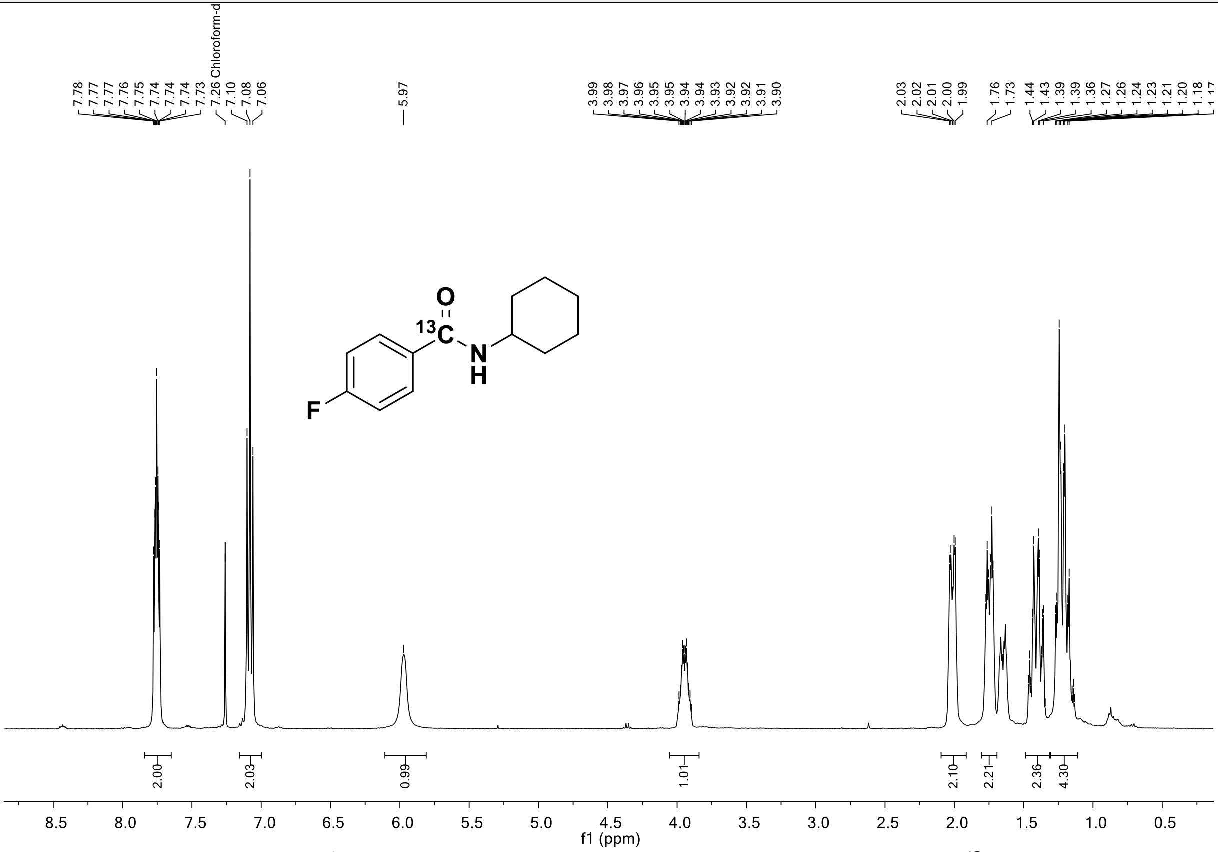

Supplementary Figure 44. ^{1}H NMR Spectrum (400 MHz, $CDCl_3$) of compound **[^{13}C]4**.

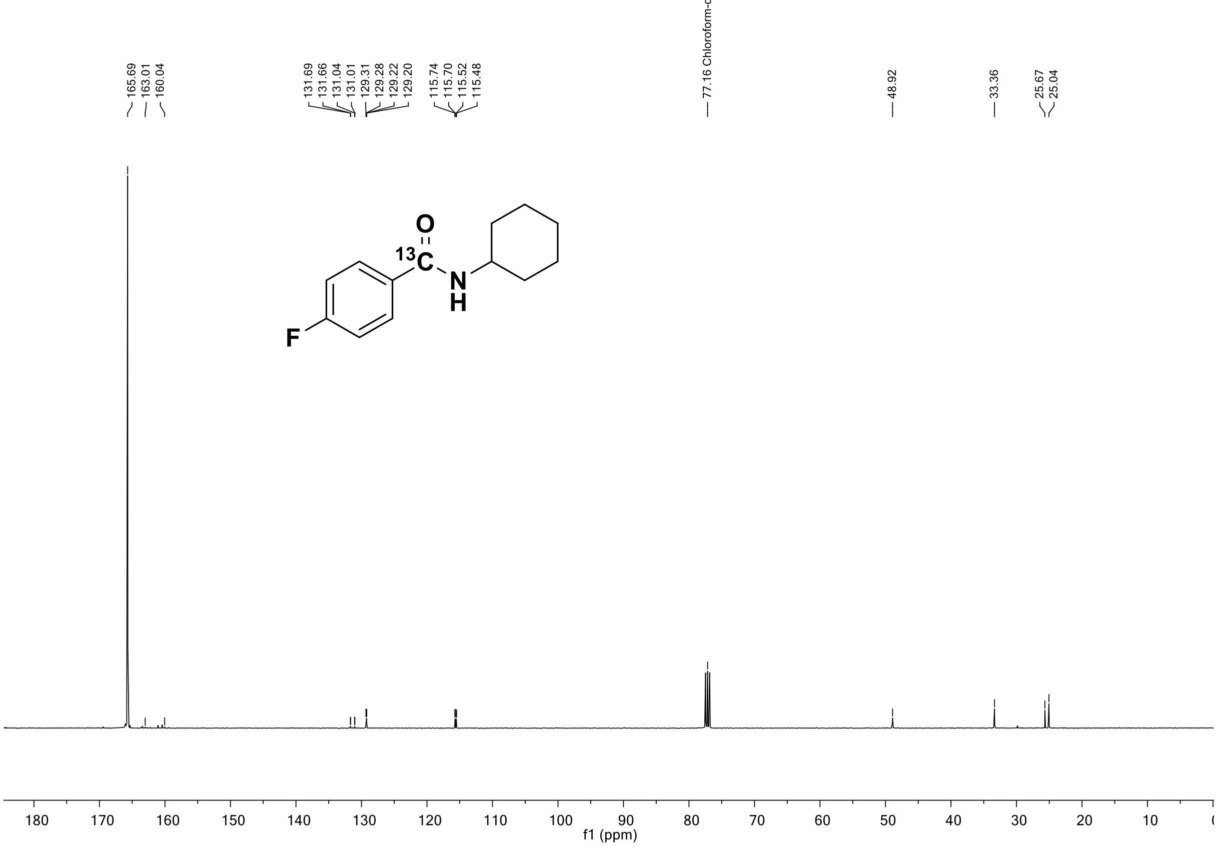

Supplementary Figure 45. ^{13}C NMR Spectrum (100 MHz, $CDCl_3$) of compound **[^{13}C]4**.

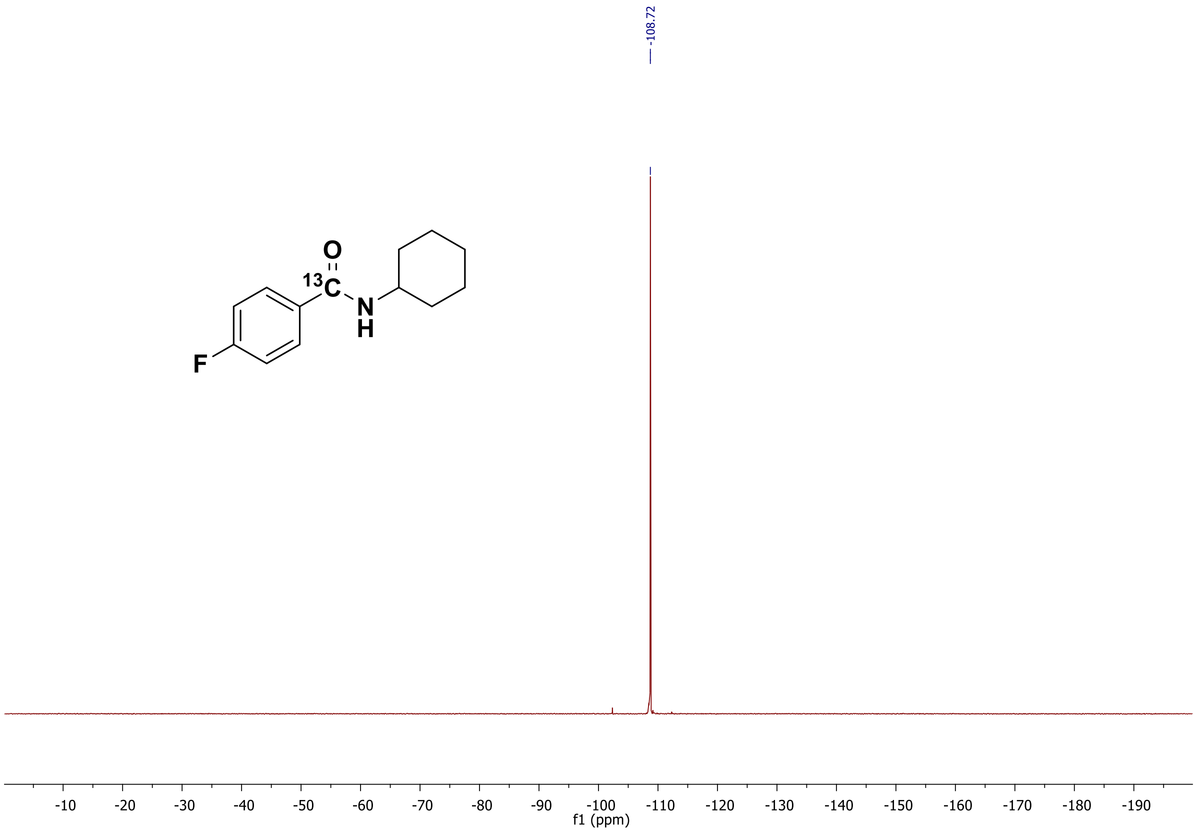

Supplementary Figure 46. ^{19}F NMR Spectrum (376 MHz, $CDCl_3$) of compound **[^{13}C]4**.

[^{13}C] N-(adamantan-1-yl)-2-(trifluoromethyl)benzamide ([^{13}C]5)

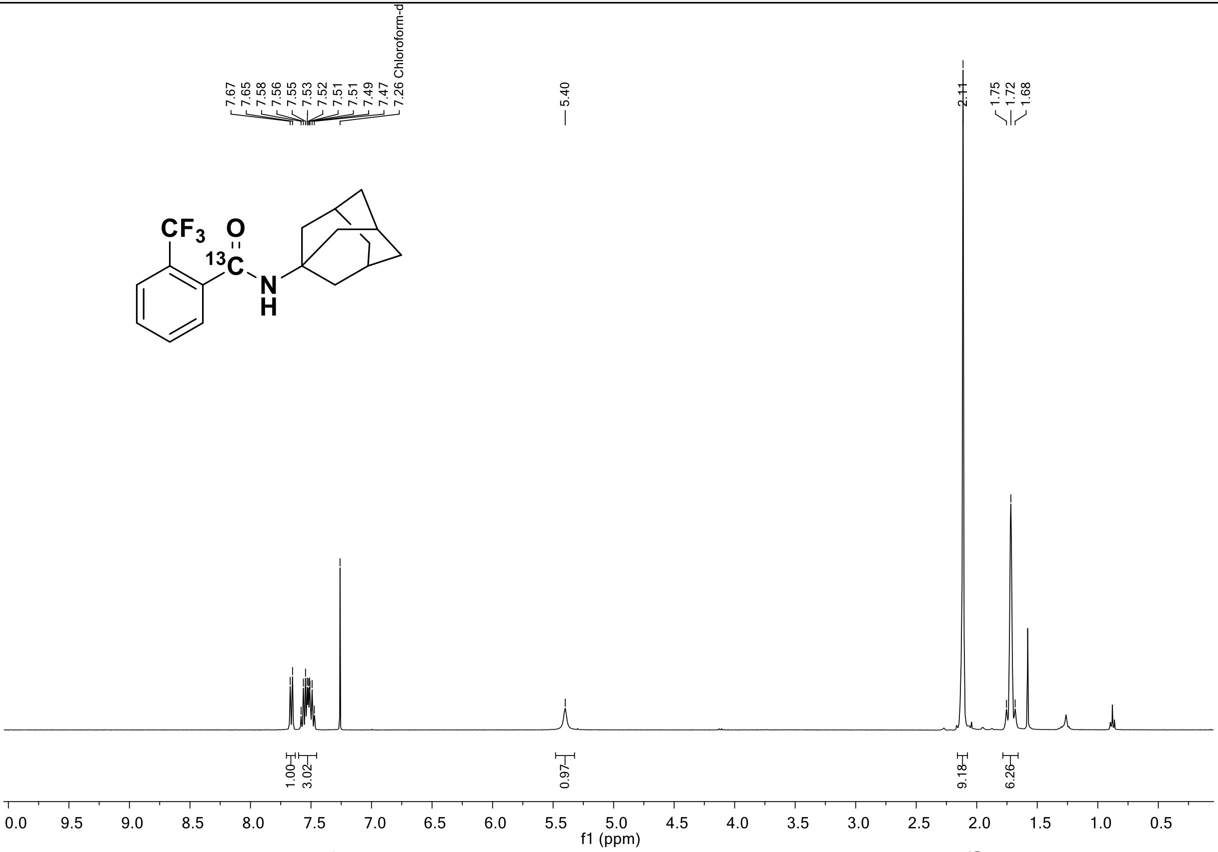

Supplementary Figure 47. ^{1}H NMR Spectrum (400 MHz, $CDCl_3$) of compound **[^{13}C]5**.

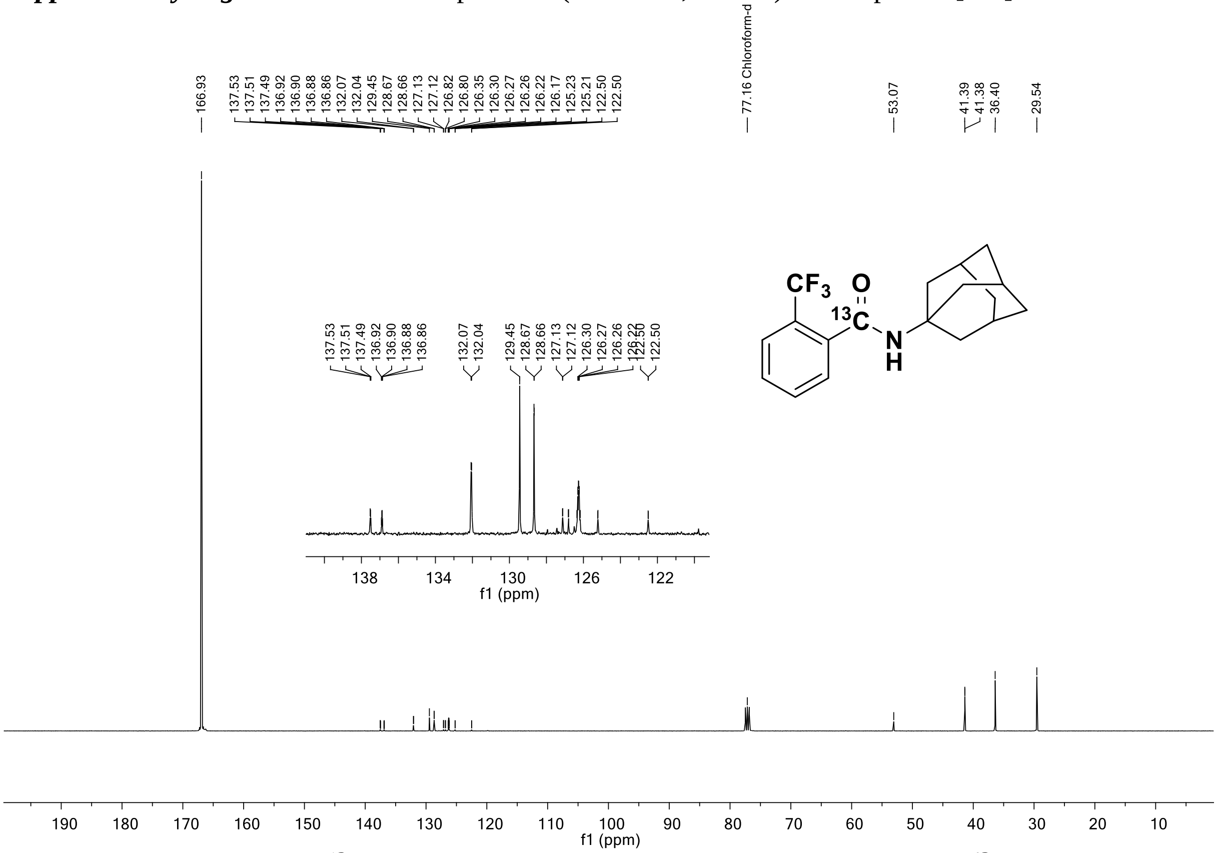

Supplementary Figure 48. ^{13}C NMR Spectrum (100 MHz, $CDCl_3$) of compound **[^{13}C]5**.

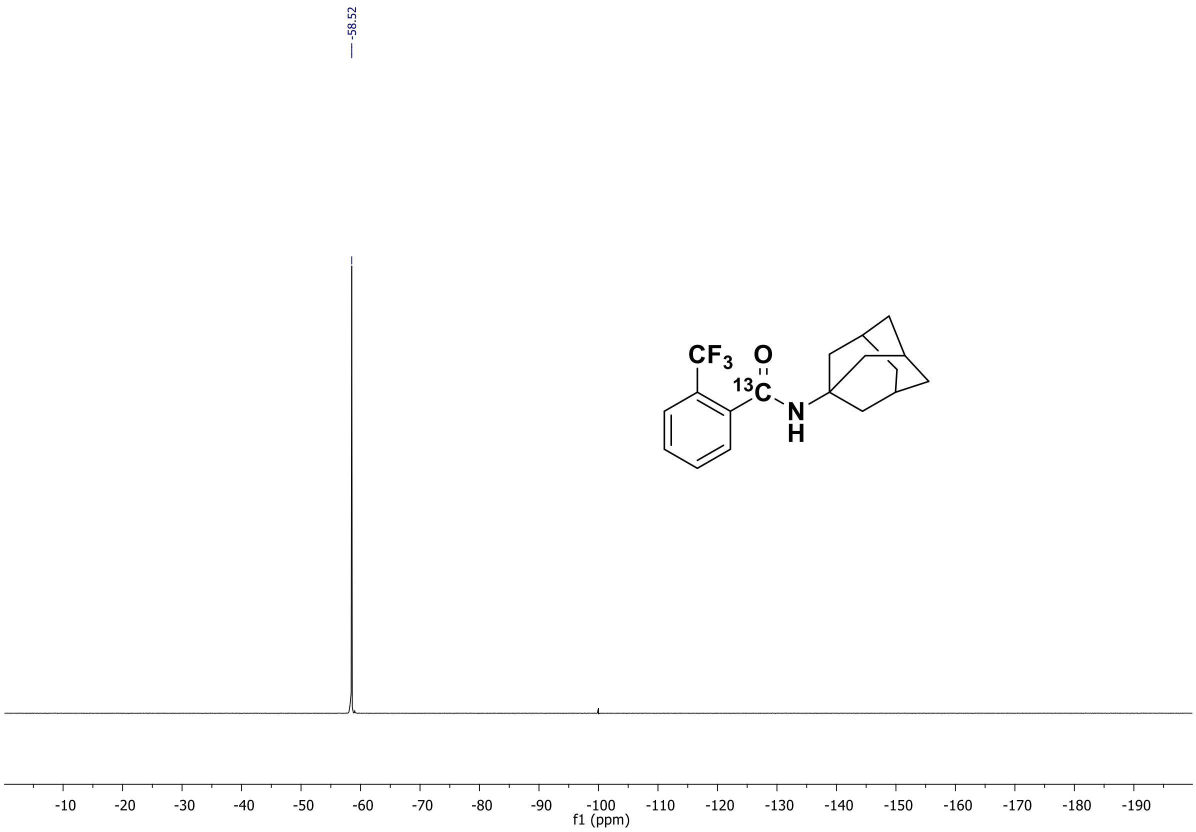

Supplementary Figure 49. ^{19}F NMR Spectrum (376 MHz, $CDCl_3$) of compound **[^{13}C]5**.

[13C] (1H-indol-5-yl)(4-methylpiperidin-1-yl)methanone ([13C]6)

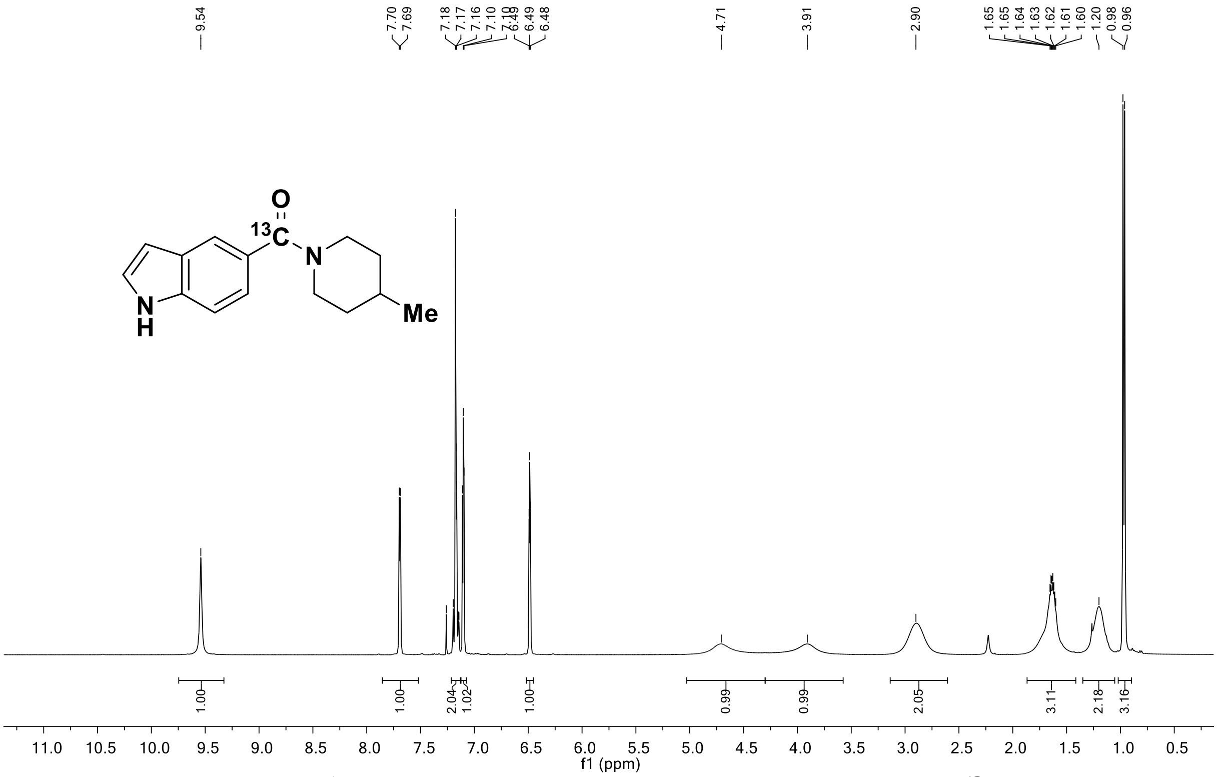

Supplementary Figure 50. ^{1}H NMR Spectrum (400 MHz, $CDCl_3$) of compound **[^{13}C]6**.

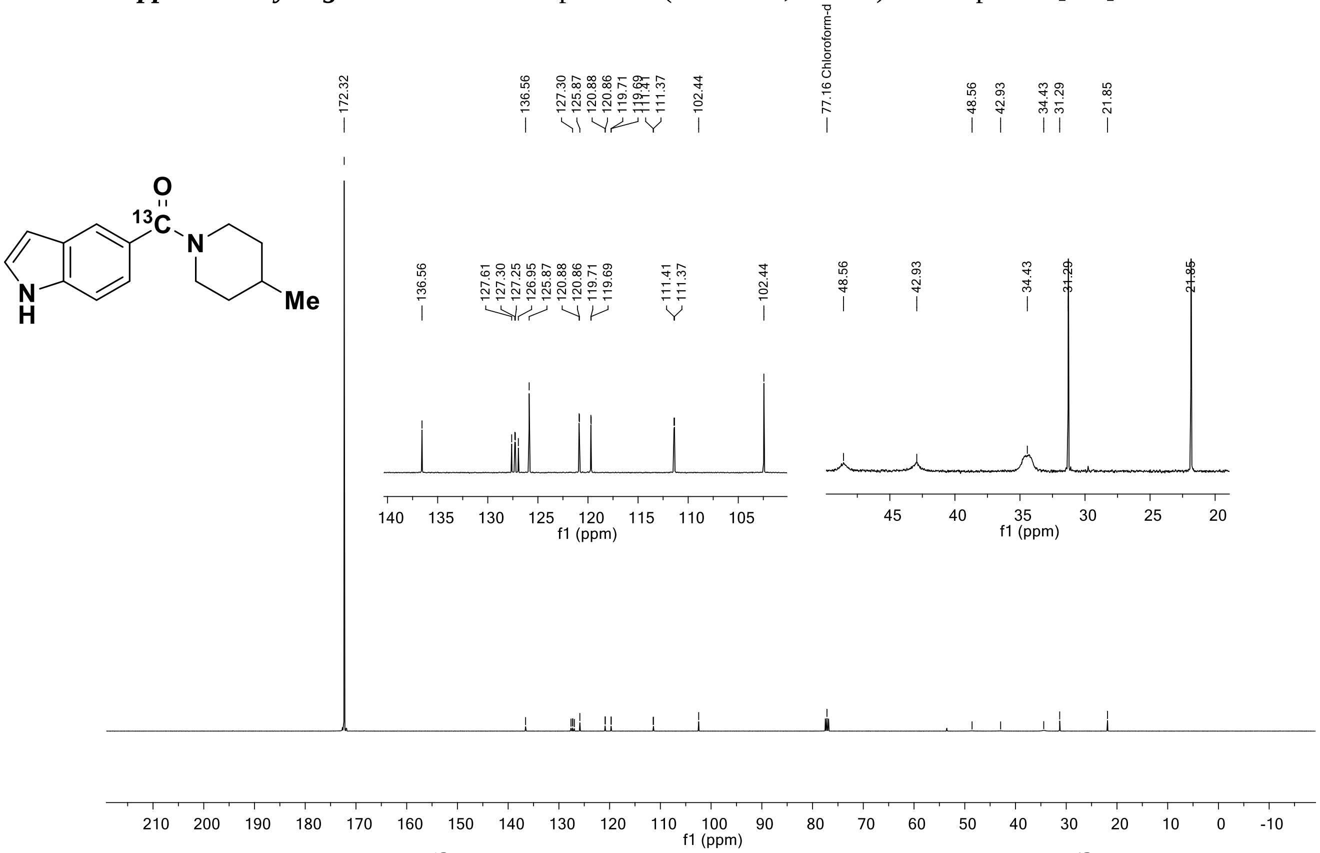

Supplementary Figure 51. ^{13}C NMR Spectrum (100 MHz, $CDCl_3$) of compound **[^{13}C]6**.

[^{13}C] adamantan-1-ylmethyl 4-butoxybenzoate ([^{13}C]7)

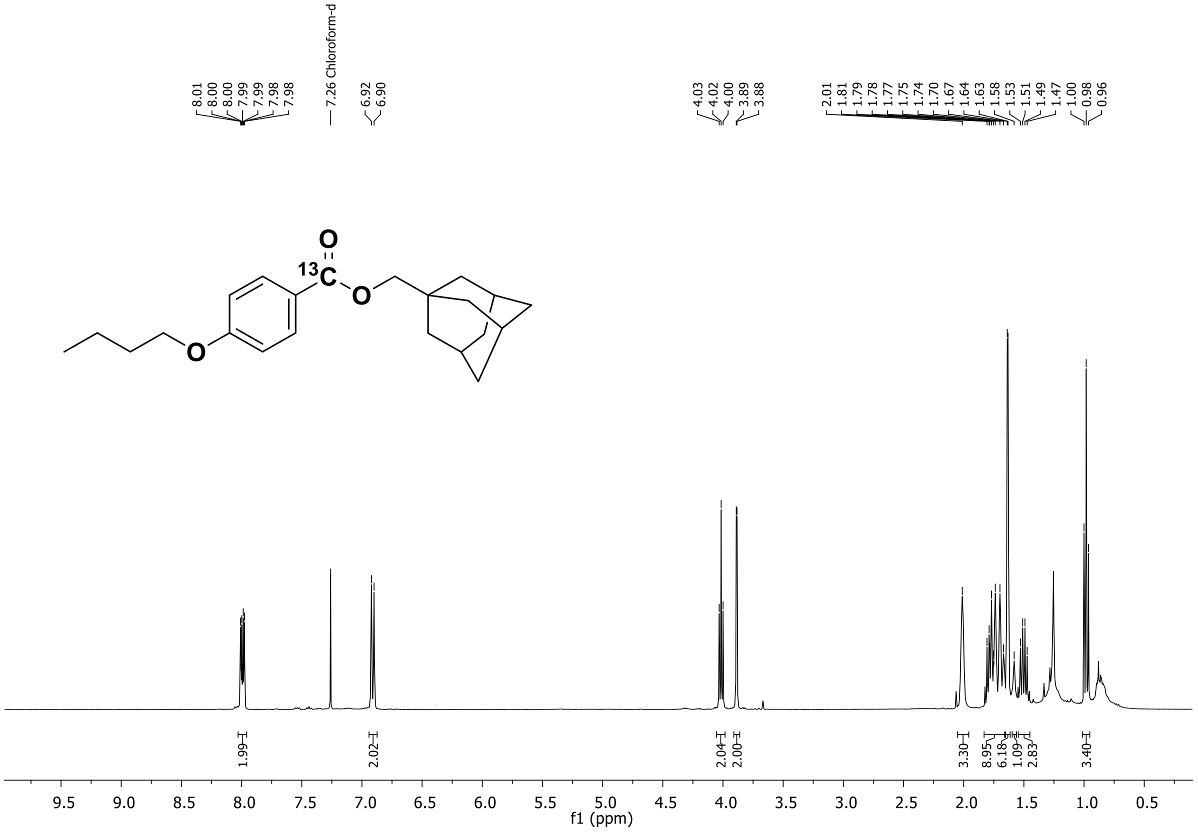

Supplementary Figure 52. ^{1}H NMR Spectrum (400 MHz, $CDCl_3$) of compound **[^{13}C]7**.

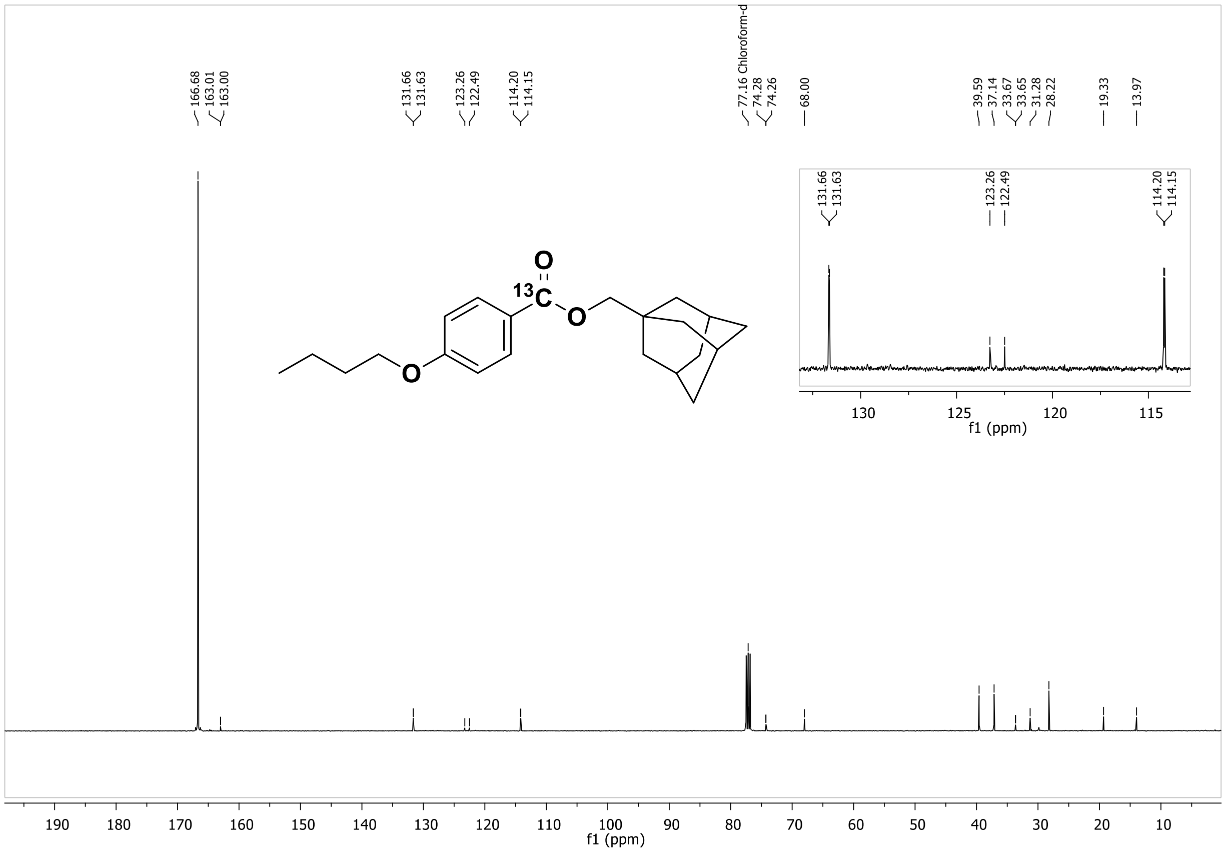

Supplementary Figure 53. ^{13}C NMR Spectrum (100 MHz, $CDCl_3$) of compound **[^{13}C]7**.

[^{13}C] 2-(piperidin-1-yl)ethyl 2,3-dihydrobenzo[b][1,4]dioxine-6-carboxylate ([^{13}C]8)

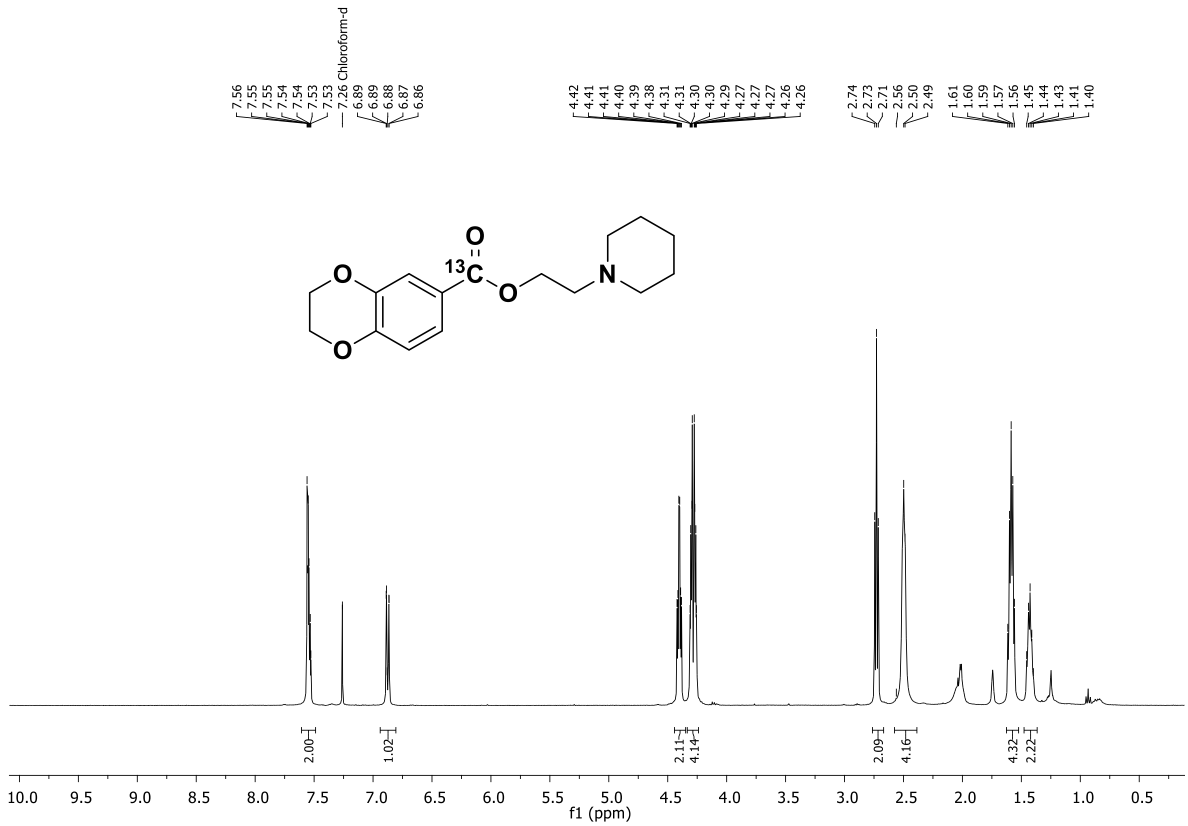

Supplementary Figure 54. ^{1}H NMR Spectrum (400 MHz, $CDCl_3$) of compound **[^{13}C]8**.

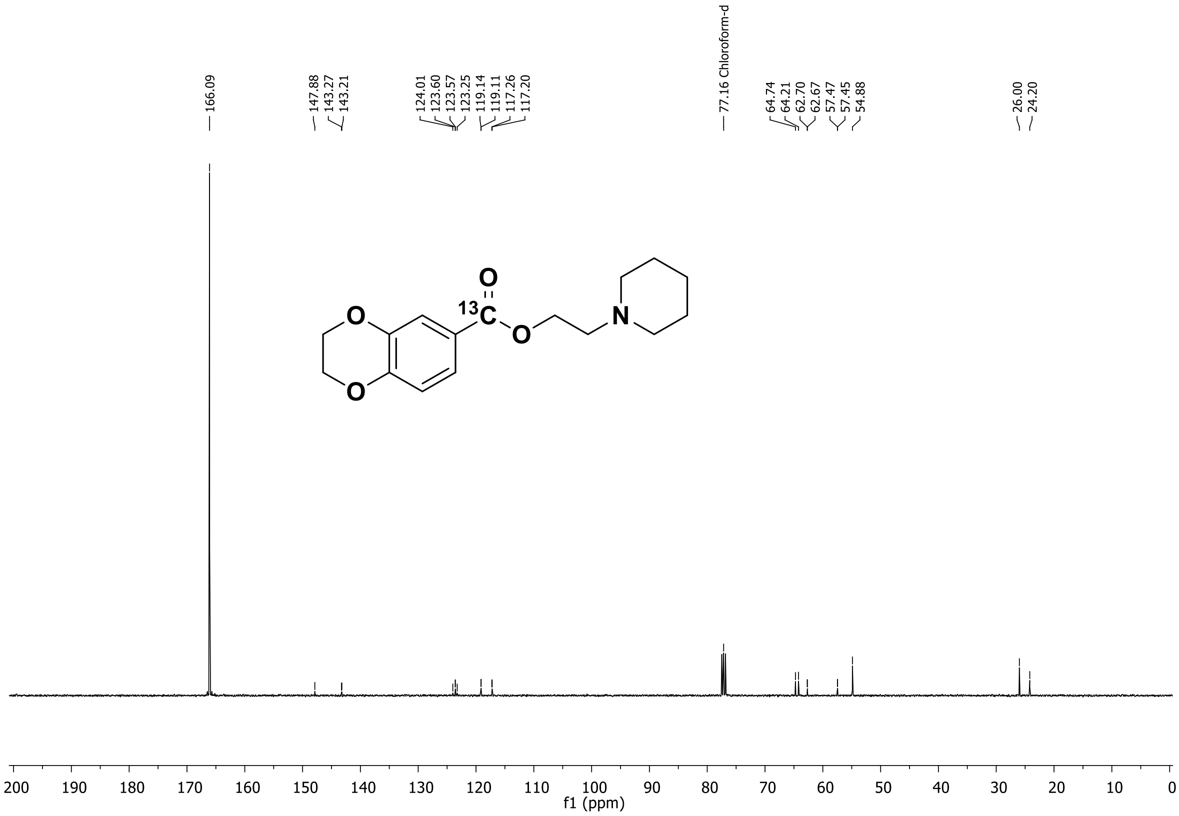

Supplementary Figure 55. ^{13}C NMR Spectrum (100 MHz, $CDCl_3$) of compound **[^{13}C]8**.

[^{13}C] 2-(benzofuran-3-yl)ethyl 4-cyanobenzoate ([^{13}C]9)

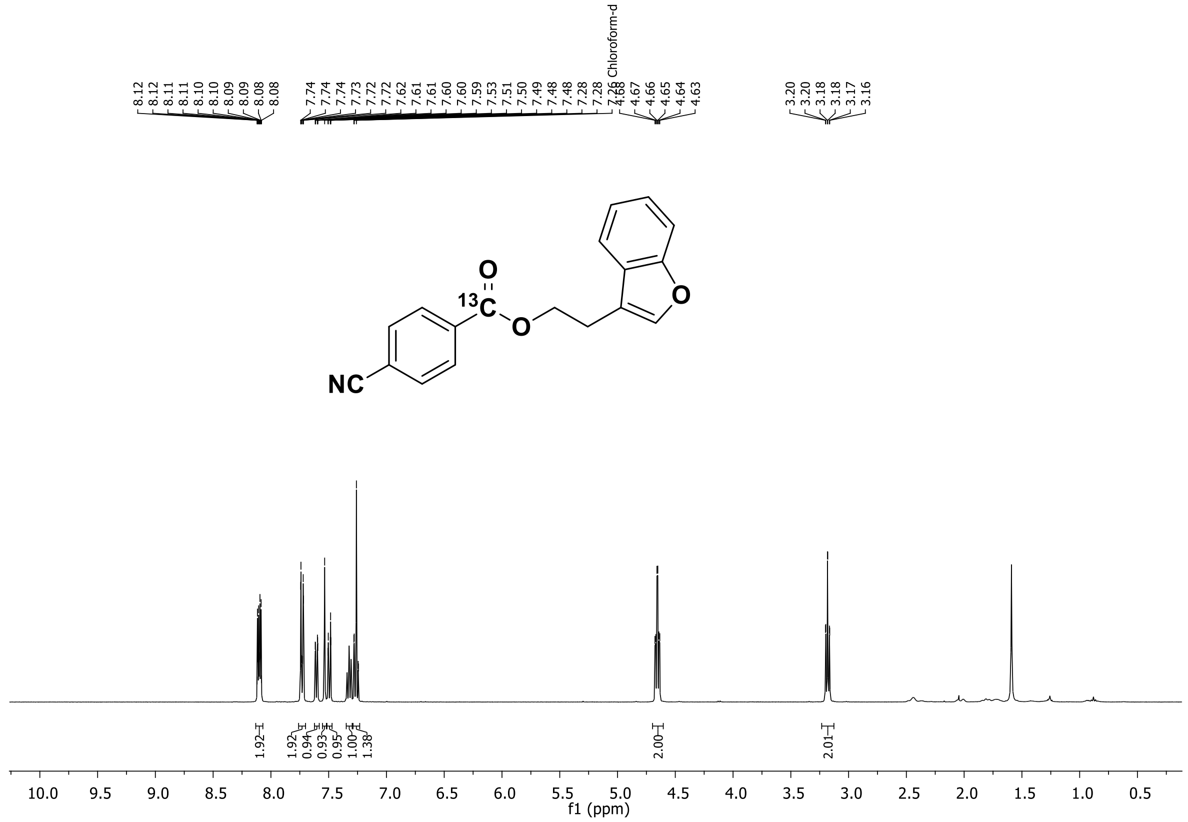

Supplementary Figure 56. ^{1}H NMR Spectrum (400 MHz, $CDCl_3$) of compound **[^{13}C]9**.

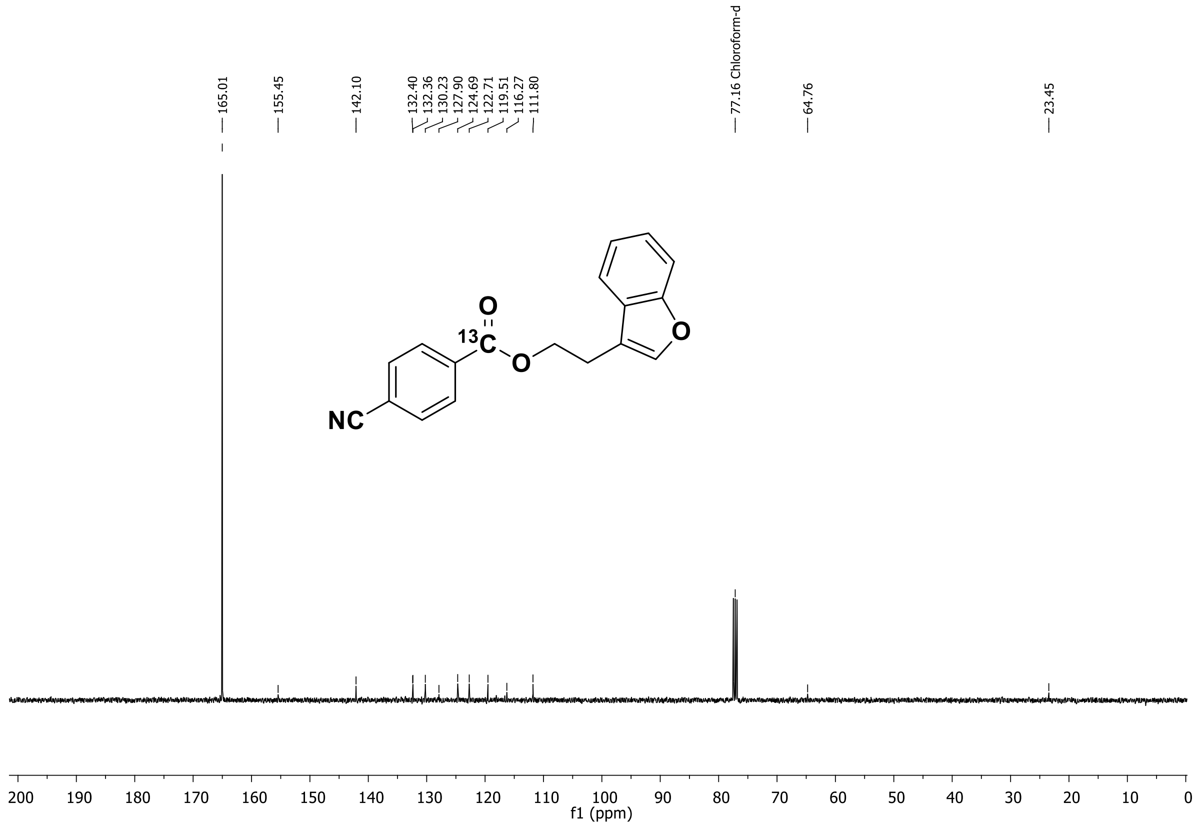

Supplementary Figure 57. ^{13}C NMR Spectrum (100 MHz, $CDCl_3$) of compound **[^{13}C]9**.

[^{13}C]-2-(piperidin-1-yl)ethyl 4-cyanobenzoate ([^{13}C]10)

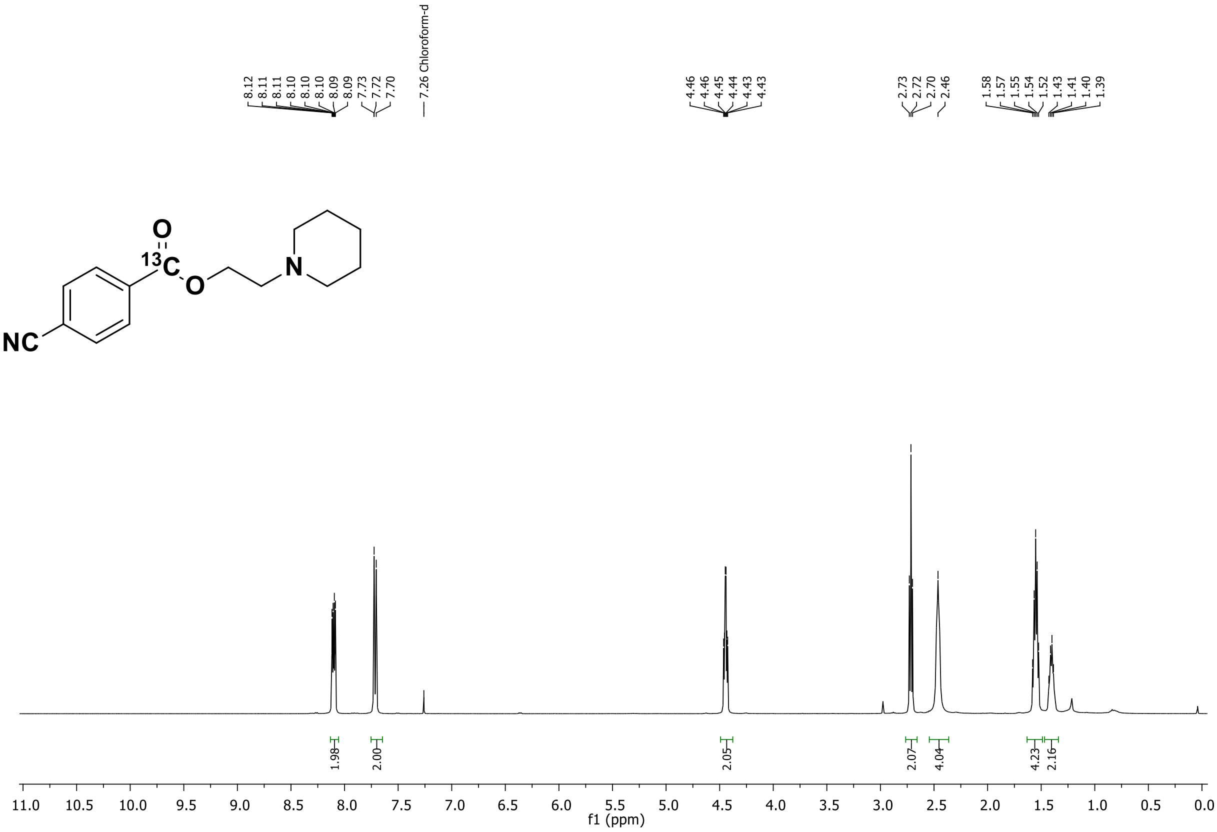

Supplementary Figure 58. ^{1}H NMR Spectrum (400 MHz, $CDCl_3$) of compound **[^{13}C]10**.

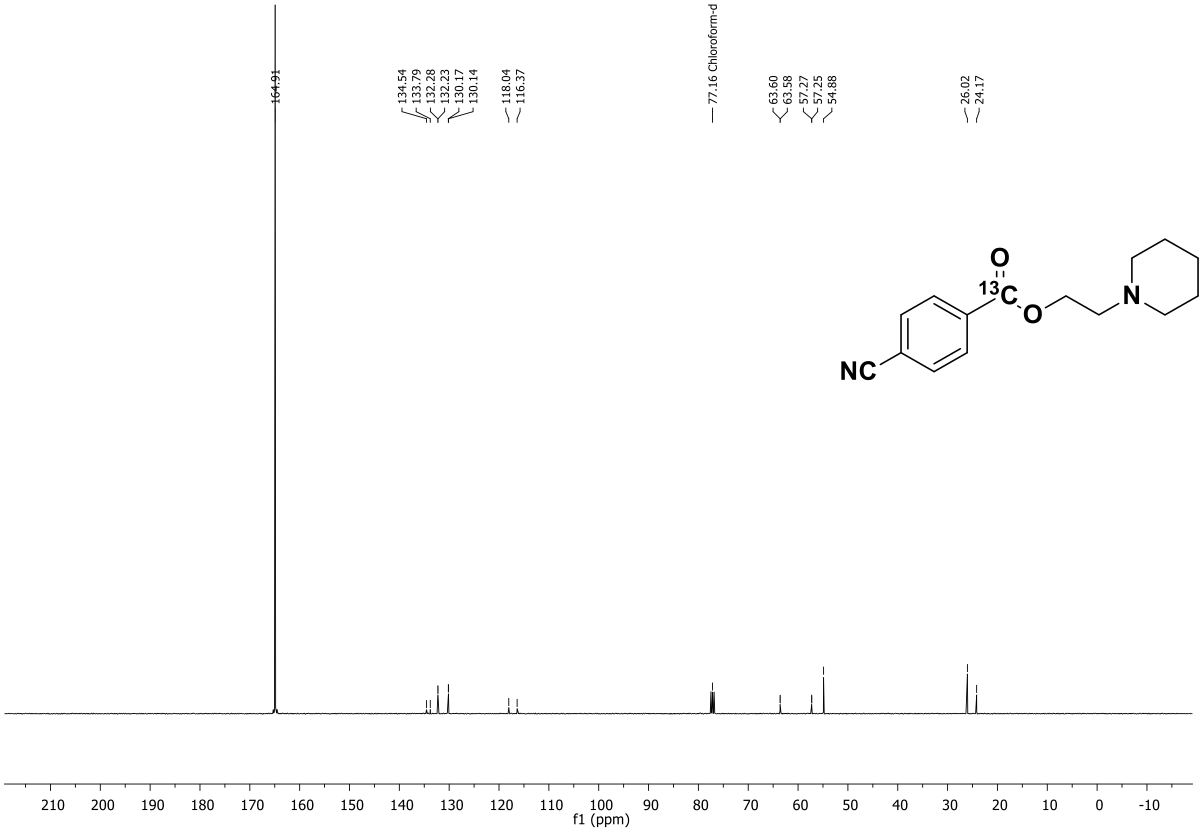

Supplementary Figure 59. ^{13}C NMR Spectrum (100 MHz, $CDCl_3$) of compound **[^{13}C]10**.

[13C] thiophen-2-ylmethyl 1H-indole-5-carboxylate ([13C]11)

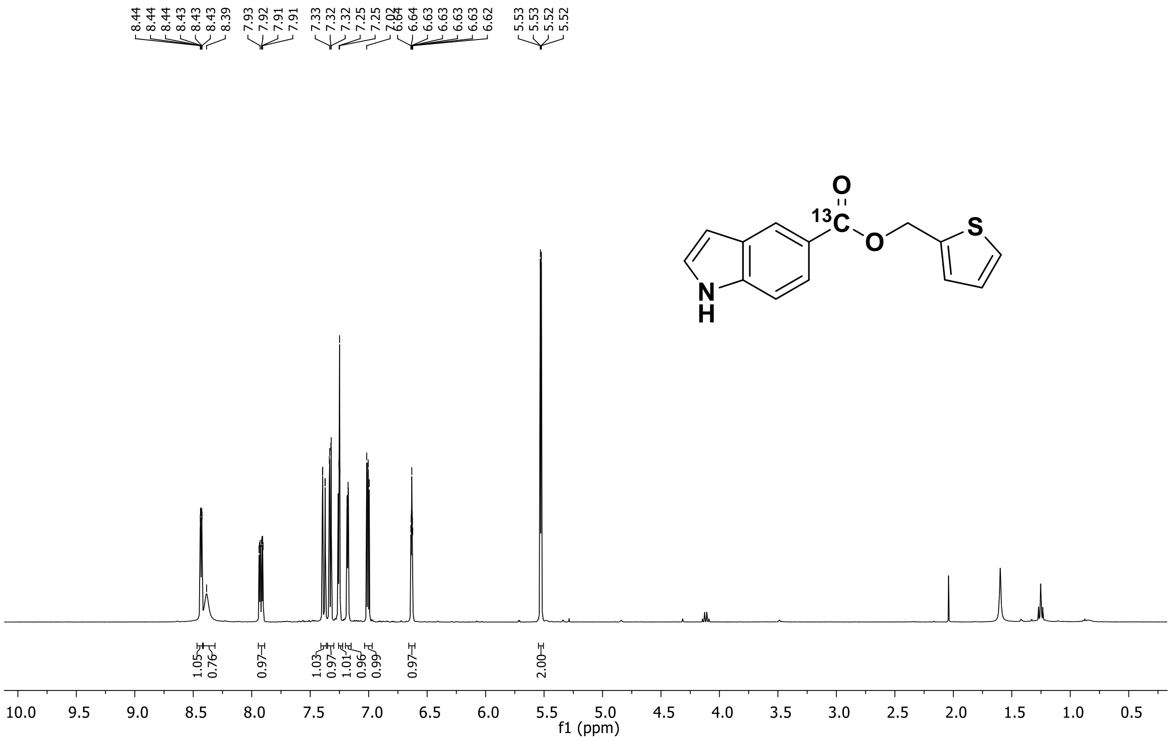

Supplementary Figure 60. ^{1}H NMR Spectrum (400 MHz, $CDCl_3$) of compound **[^{13}C]11**.

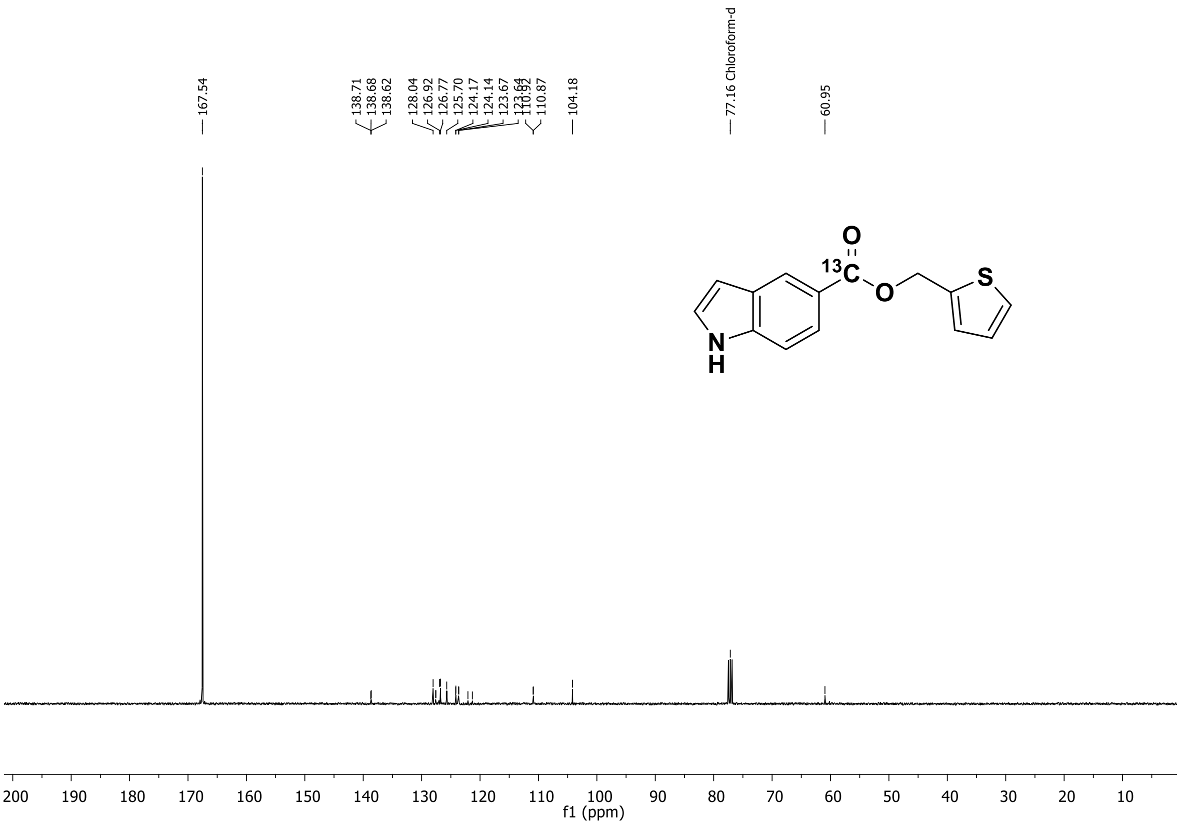

Supplementary Figure 61. ^{13}C NMR Spectrum (100 MHz, $CDCl_3$) of compound **[^{13}C]11**.

[^{13}C] (1-benzylpyrrolidin-2-yl)methyl 3-(4-(trifluoromethyl)benzoyl)benzoate ([^{13}C]12)

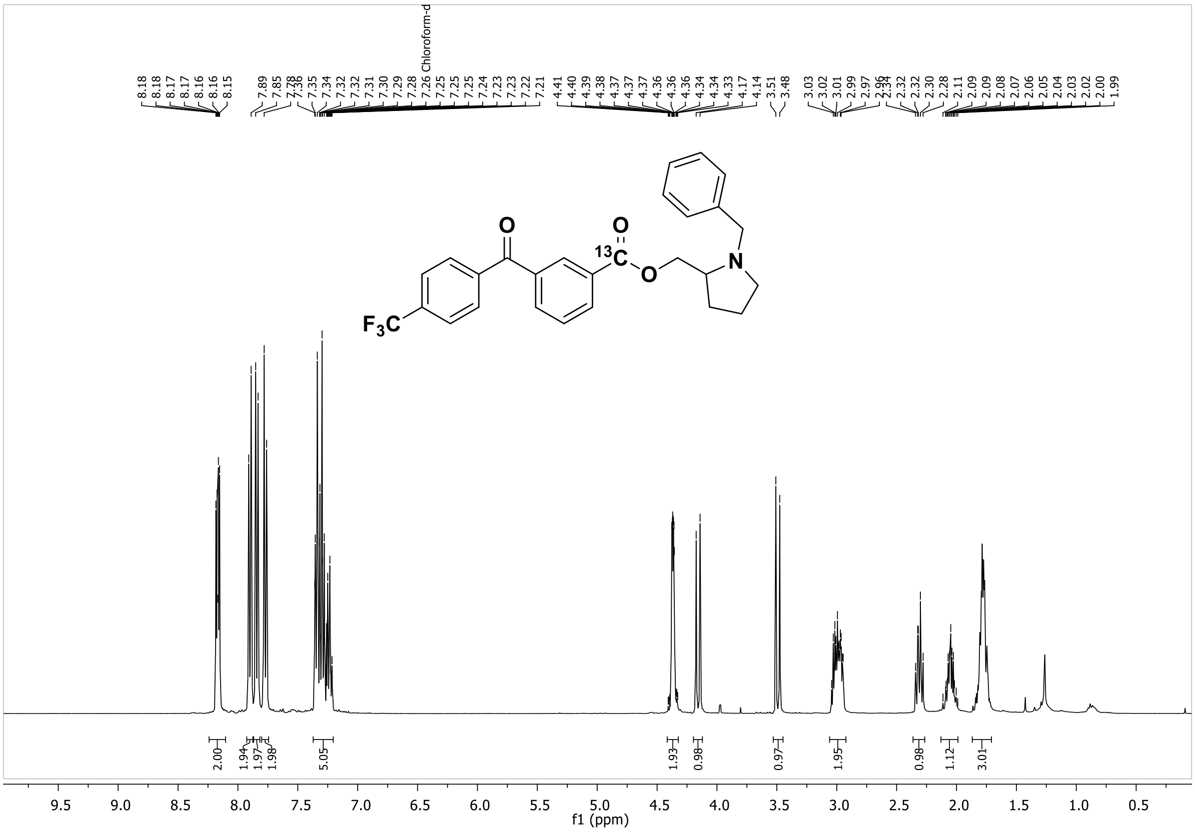

Supplementary Figure 62. ^{1}H NMR Spectrum (400 MHz, $CDCl_3$) of compound **[^{13}C]12**.

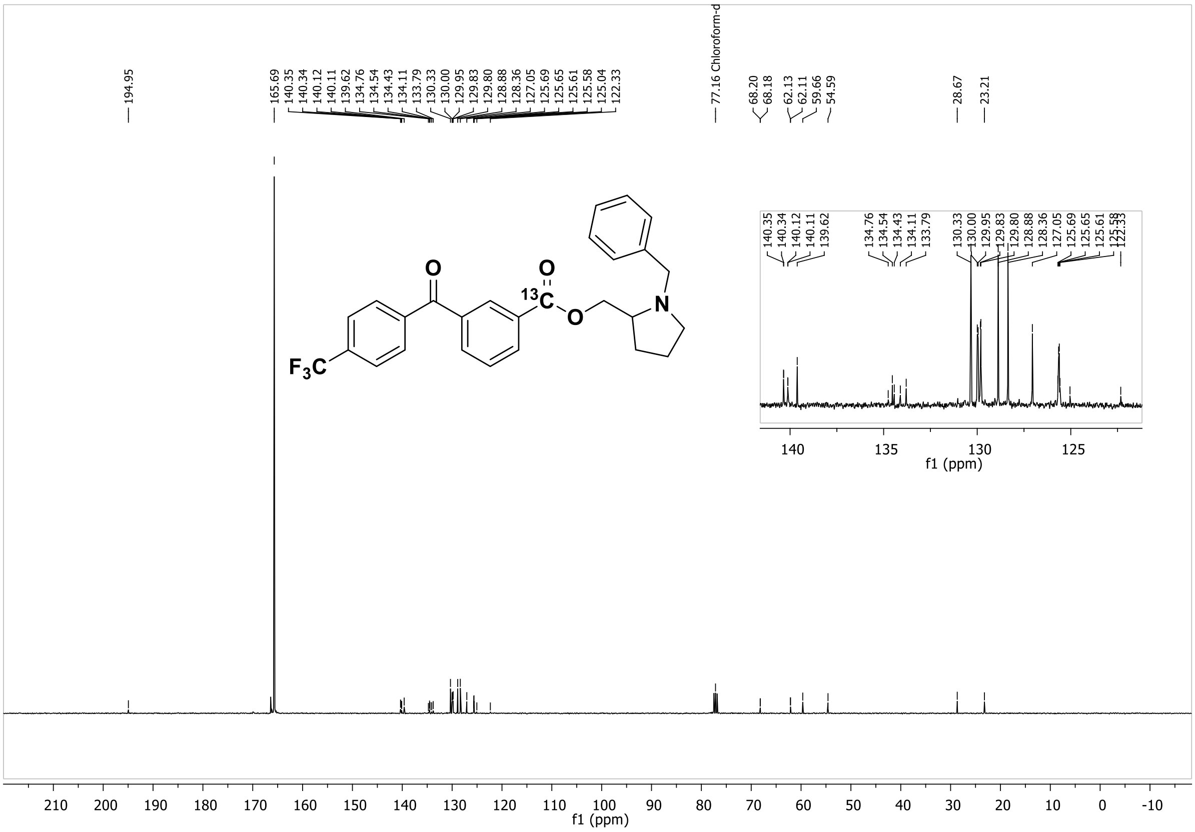

Supplementary Figure 63. ^{13}C NMR Spectrum (100 MHz, $CDCl_3$) of compound **[^{13}C]12**.

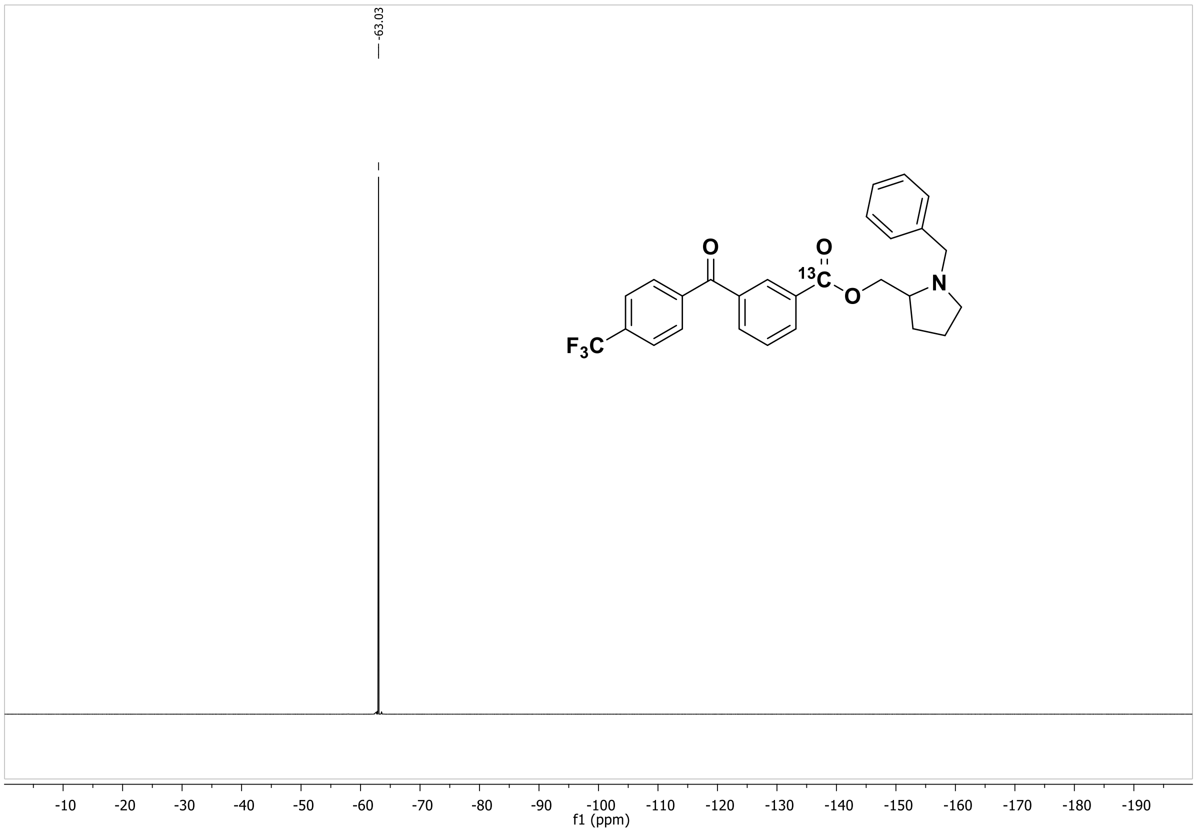

Supplementary Figure 64. ^{19}F NMR Spectrum (376 MHz, $CDCl_3$) of compound **[^{13}C]12**.

[^{13}C] (1-ethyl-1H-imidazol-2-yl)methyl 3,4,5-trimethoxybenzoate ([^{13}C]13)

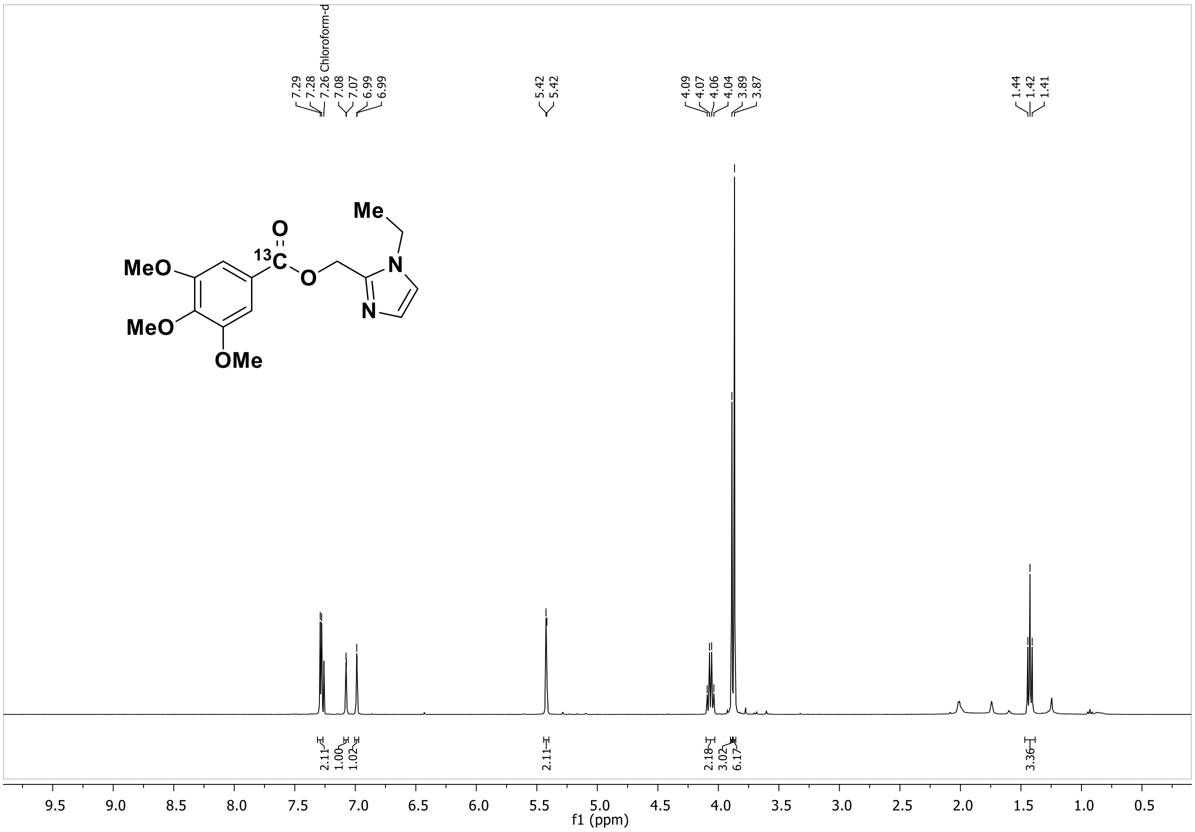

Supplementary Figure 65. ^{1}H NMR Spectrum (400 MHz, $CDCl_3$) of compound **[^{13}C]13**.

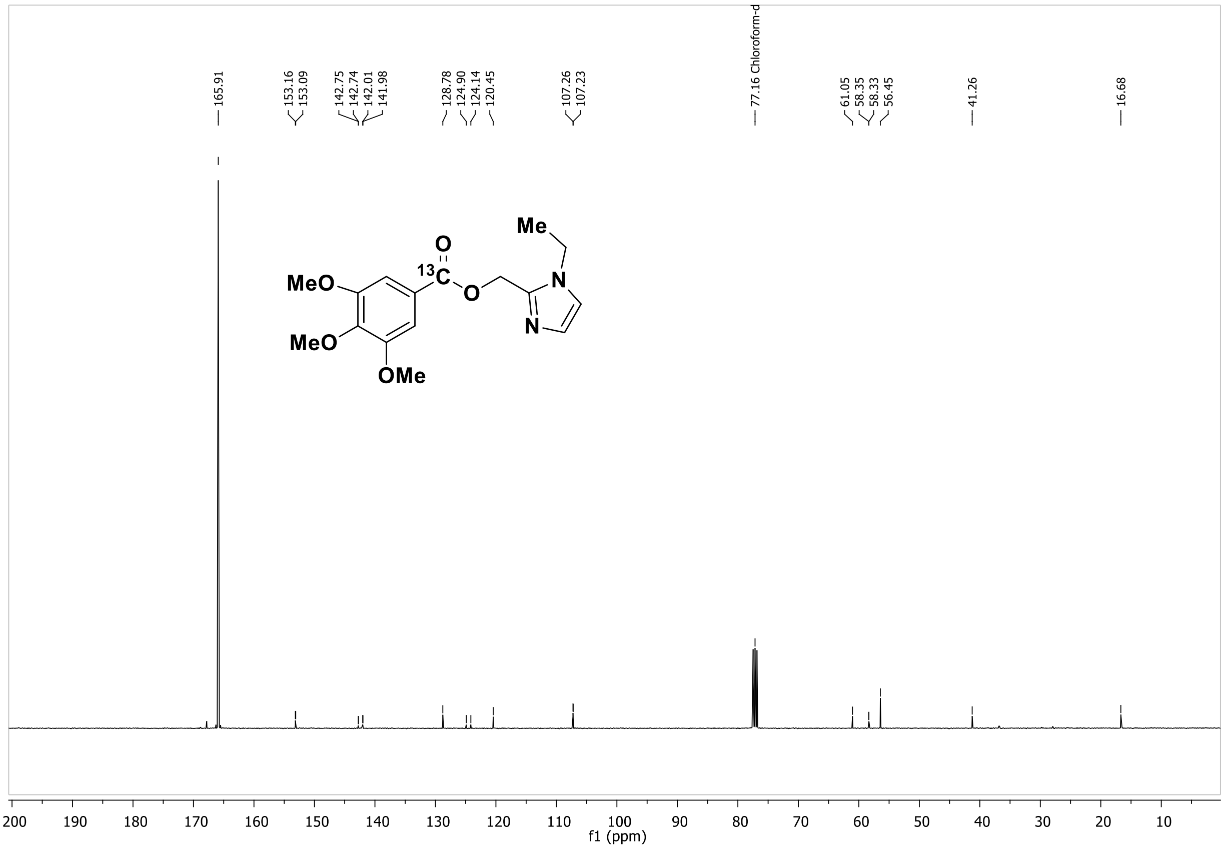

Supplementary Figure 66. ^{13}C NMR Spectrum (100 MHz, $CDCl_3$) of compound **[^{13}C]13**.

[13C] 4-(benzyloxy)benzaldehyde ([13C]14)

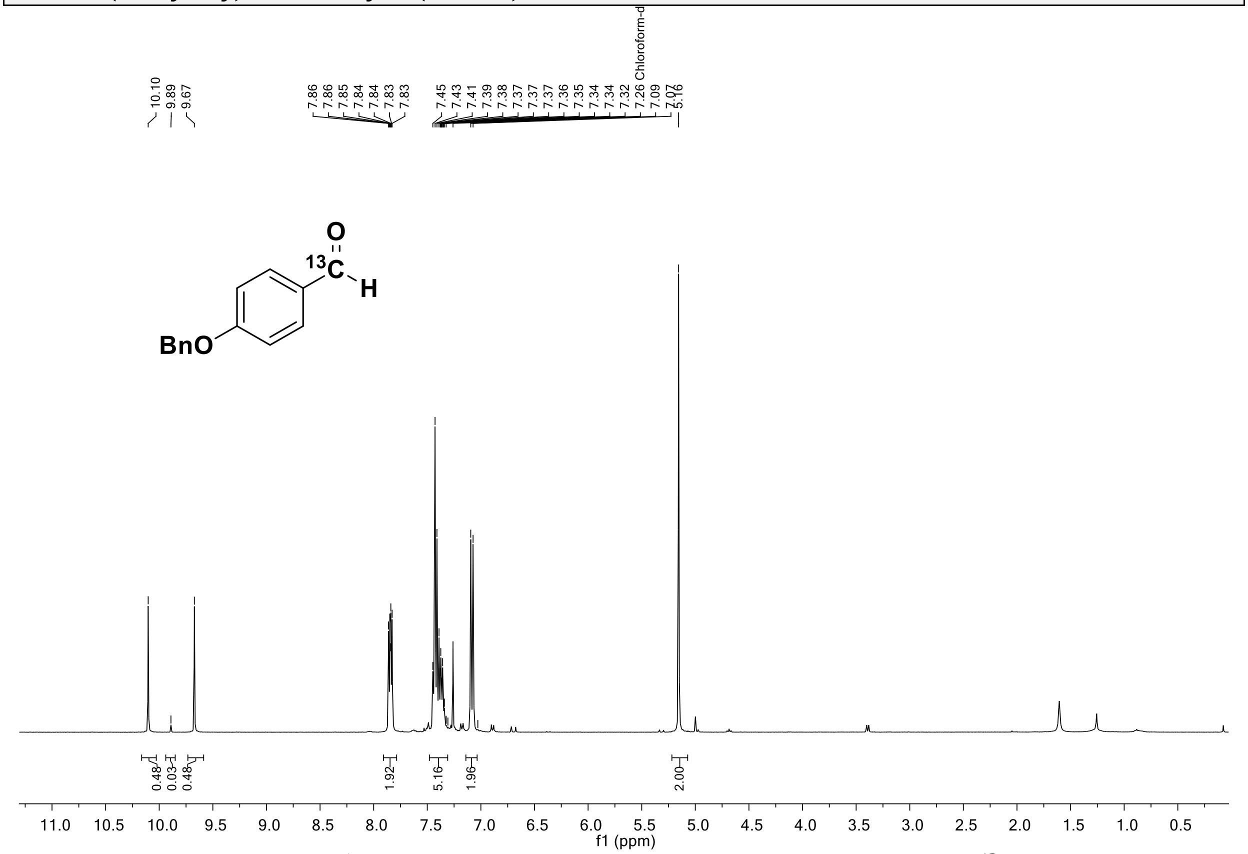

Supplementary Figure 67. ^{1}H NMR Spectrum (400 MHz, $CDCl_3$) of compound **[^{13}C]14**.

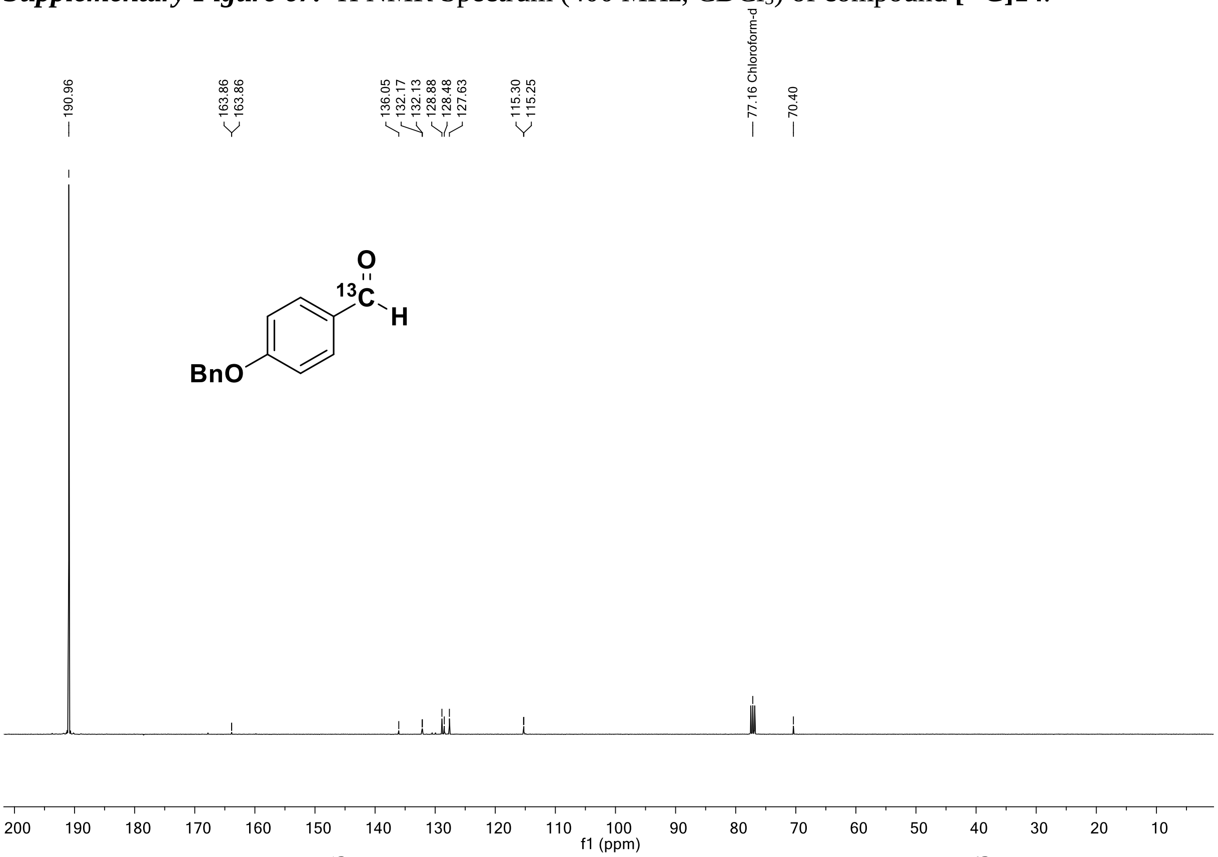

Supplementary Figure 68. ^{13}C NMR Spectrum (100 MHz, $CDCl_3$) of compound **[^{13}C]14**.

[13C] 4-chlorobenzaldehyde ([13C]15)

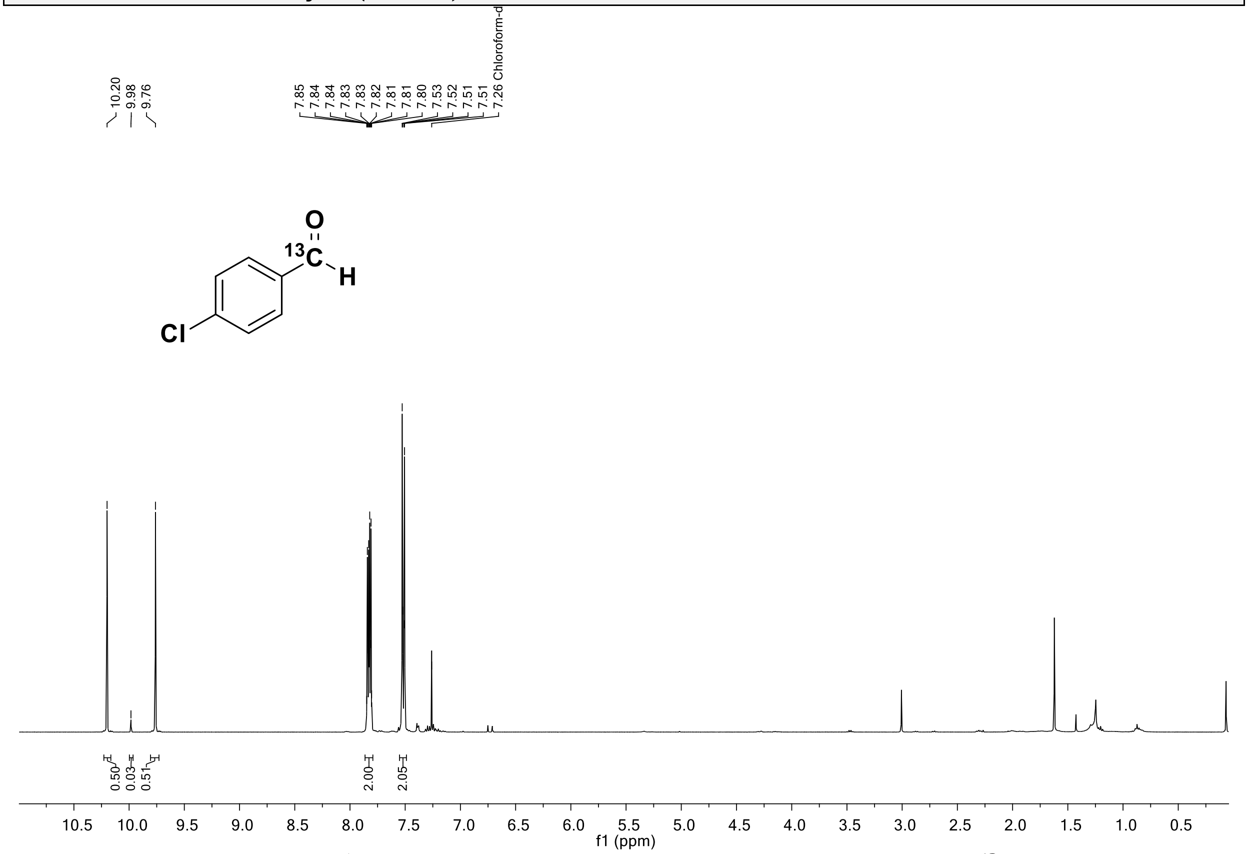

Supplementary Figure 69. ^{1}H NMR Spectrum (400 MHz, $CDCl_3$) of compound **[^{13}C]15**.

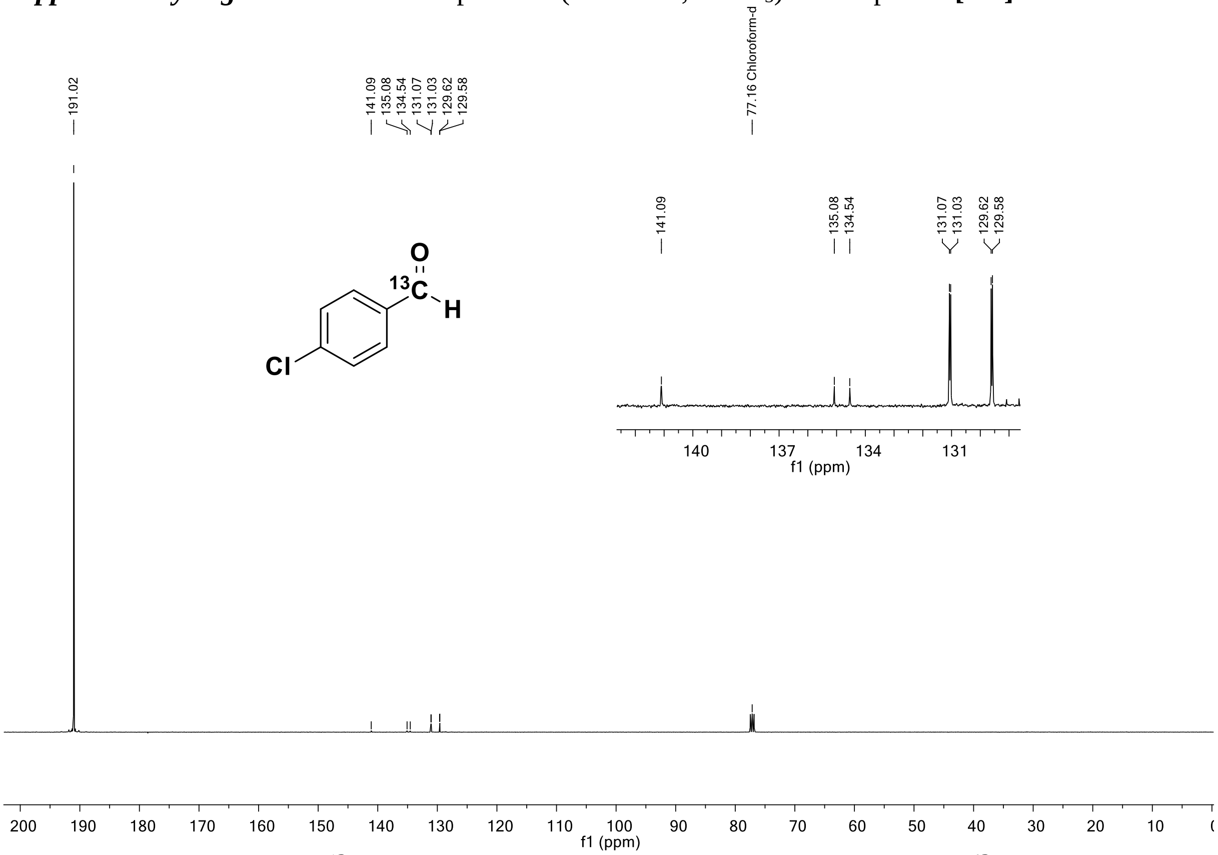

Supplementary Figure 70. ^{13}C NMR Spectrum (100 MHz, $CDCl_3$) of compound **[^{13}C]15**.

[^{13}C] 3,4,5-trimethoxybenzaldehyde ([^{13}C]16)

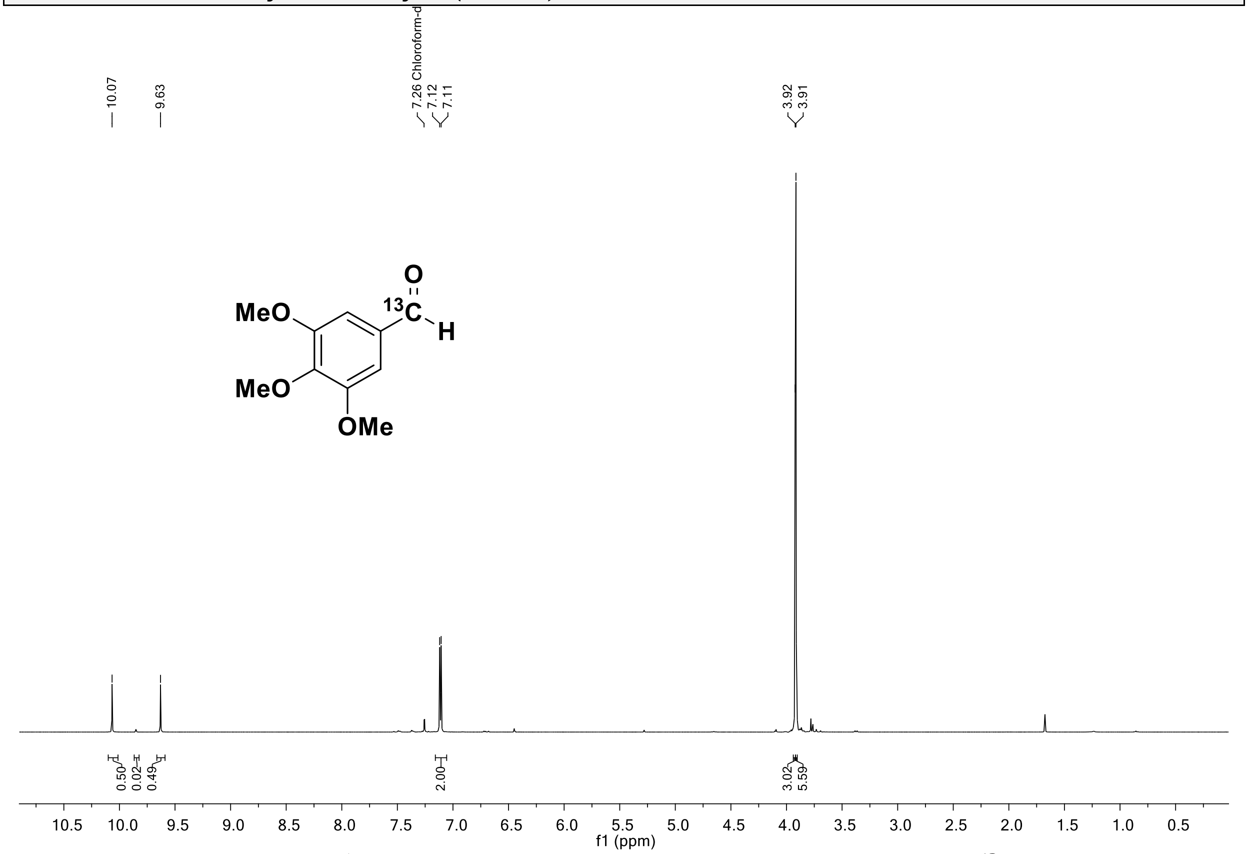

Supplementary Figure 71. ^{1}H NMR Spectrum (400 MHz, $CDCl_3$) of compound **[^{13}C]16**.

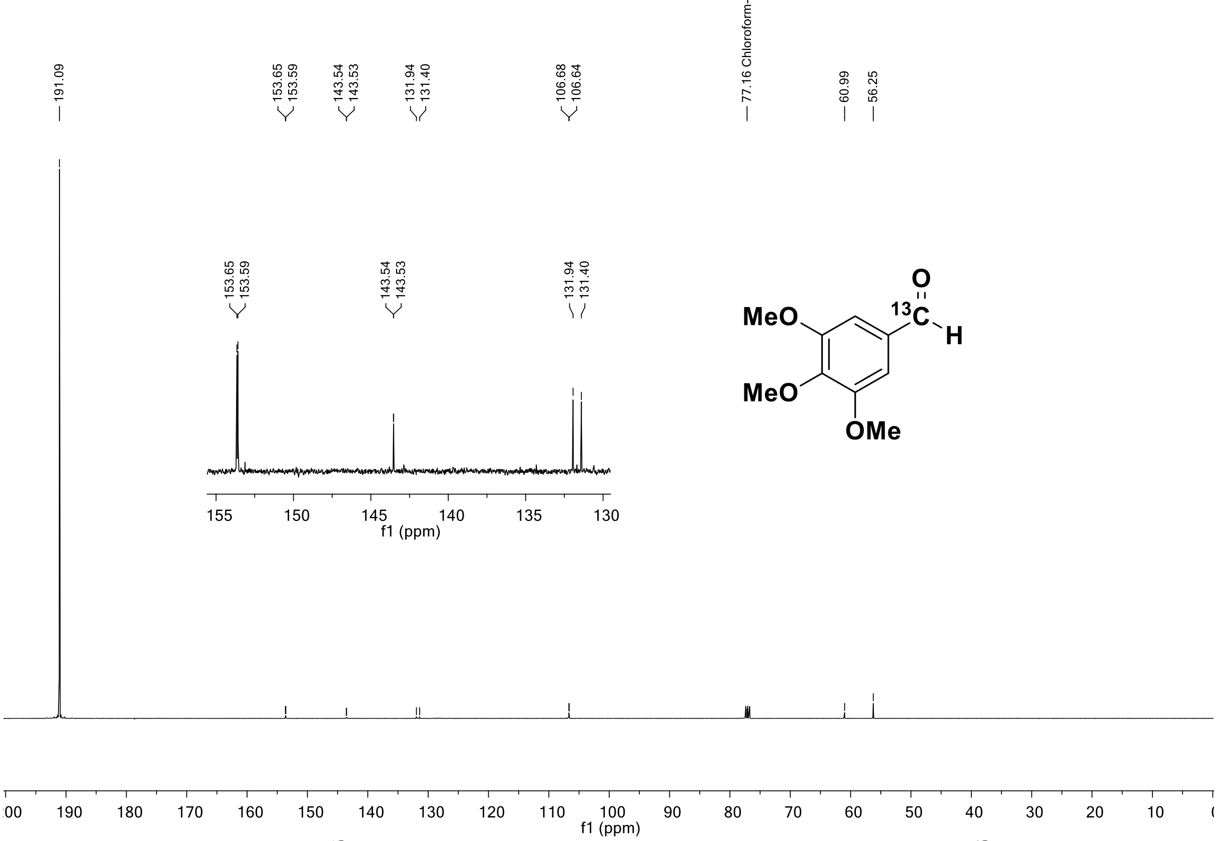

Supplementary Figure 72. ^{13}C NMR Spectrum (100 MHz, $CDCl_3$) of compound **[^{13}C]16**.

[13C] 4-((trimethylsilyl)ethynyl)benzaldehyde ([13C]17)

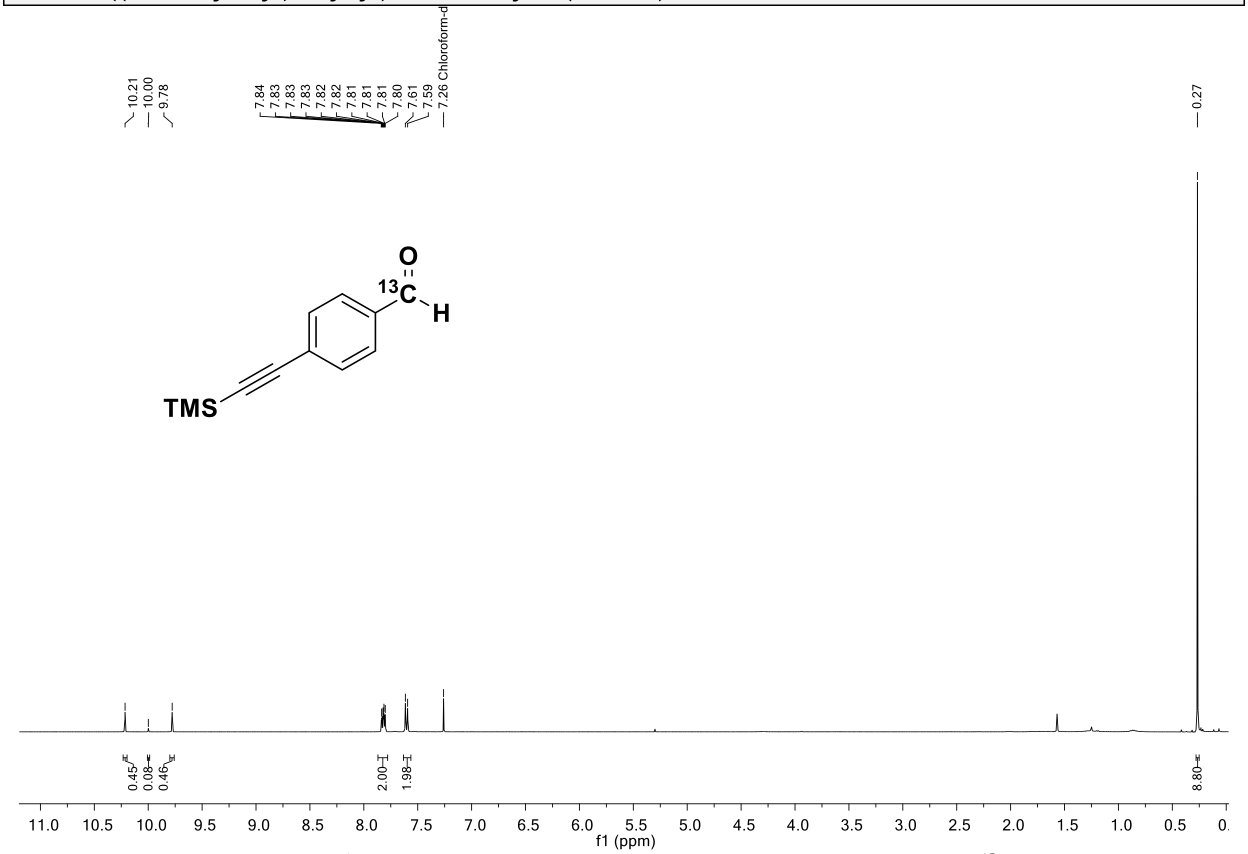

Supplementary Figure 73. ^{1}H NMR Spectrum (400 MHz, $CDCl_3$) of compound **[13C]17**.

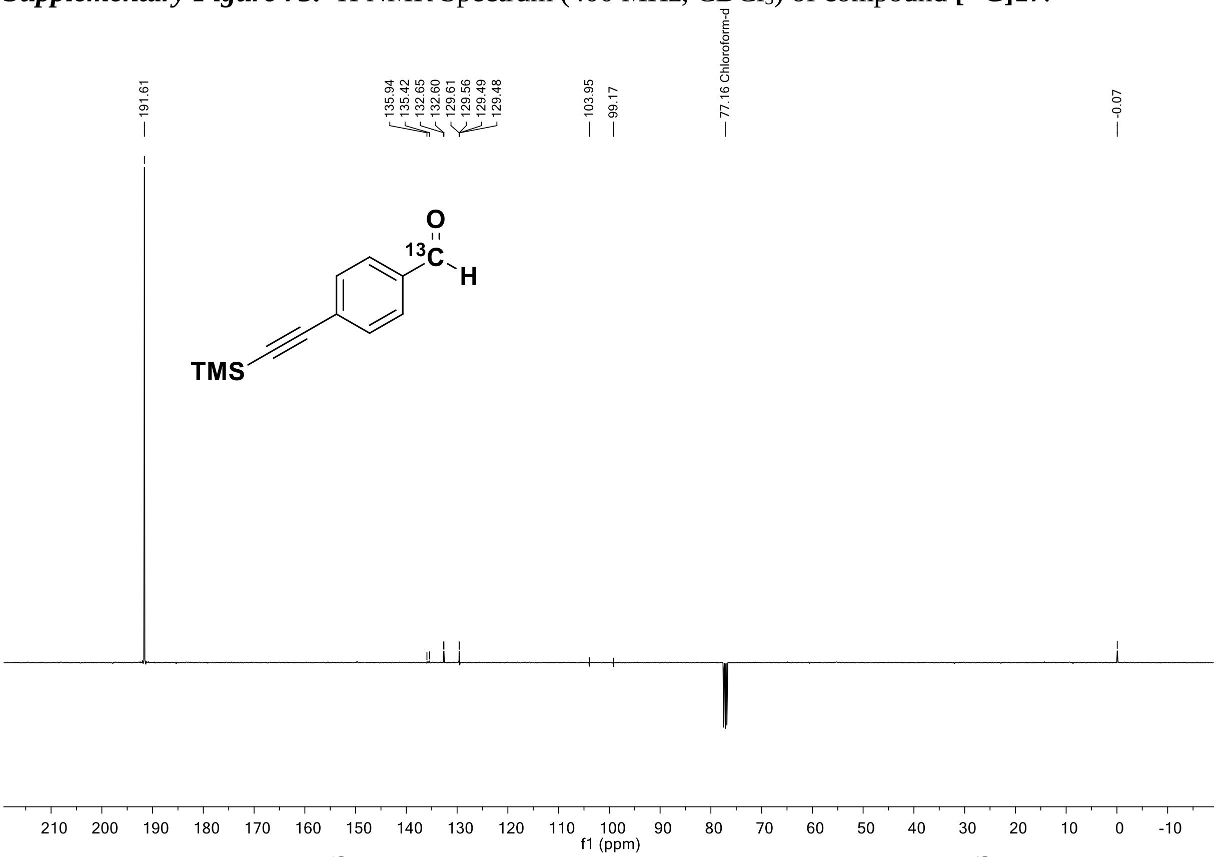

Supplementary Figure 74. ^{13}C NMR Spectrum (100 MHz, $CDCl_3$) of compound **[13C]17**.

[^{13}C] 4-formylbenzonitrile ([^{13}C]18)

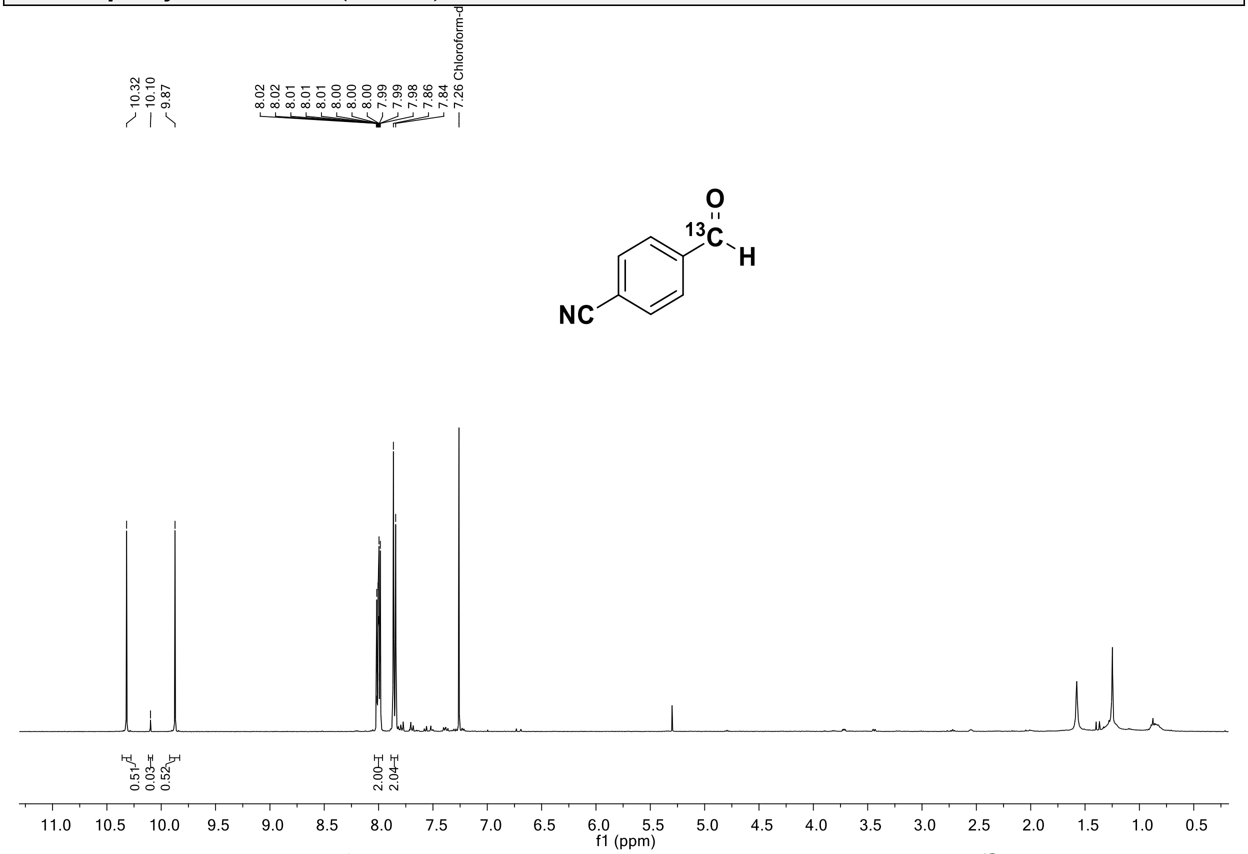

Supplementary Figure 75. ^{1}H NMR Spectrum (400 MHz, $CDCl_3$) of compound **[^{13}C]18**.

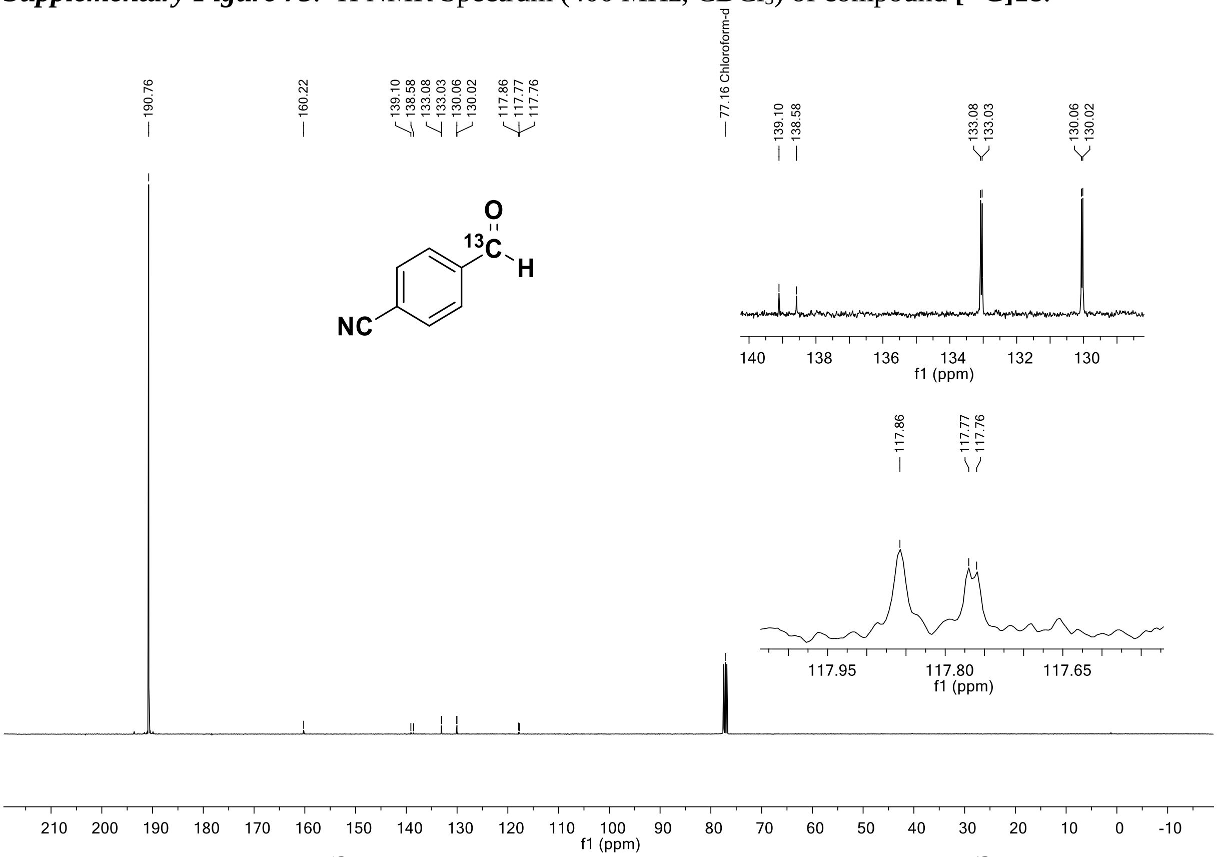

Supplementary Figure 76. ^{13}C NMR Spectrum (100 MHz, $CDCl_3$) of compound **[^{13}C]18**.

[^{13}C] 4-formylbenzoic acid ([^{13}C]19)

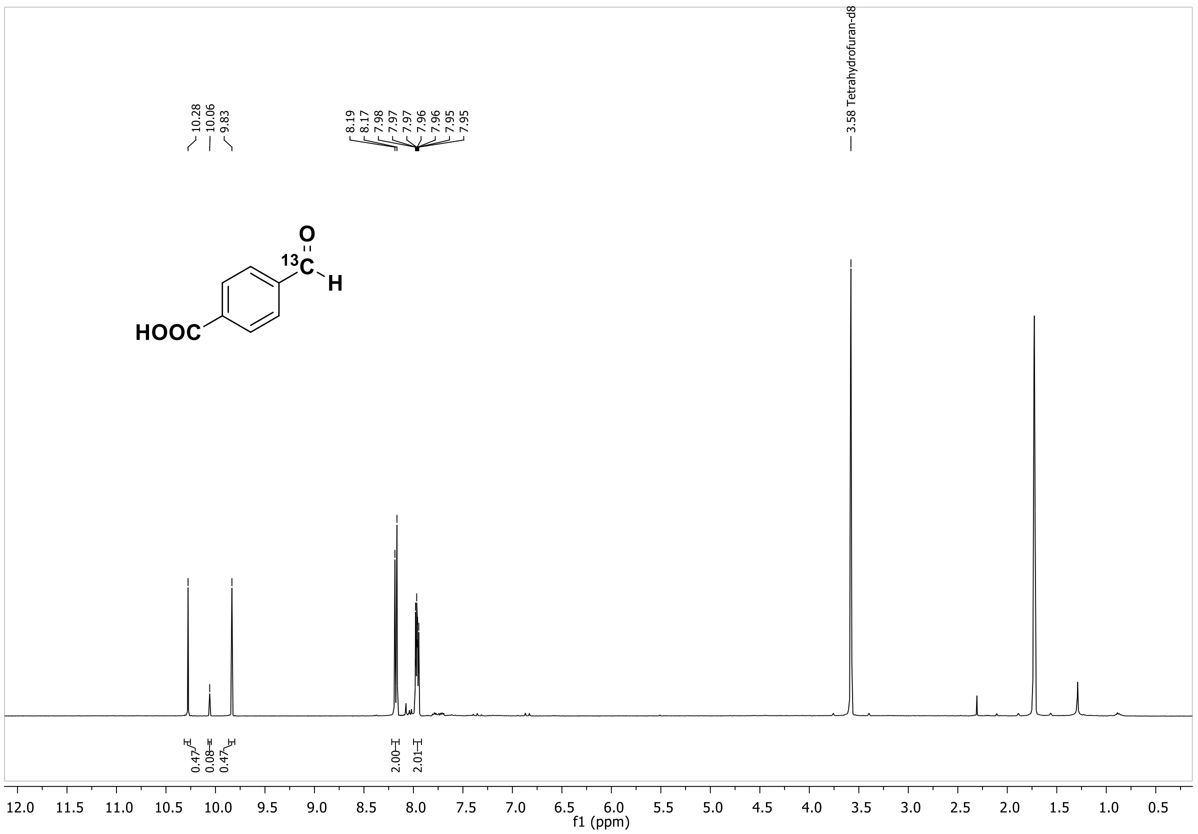

Supplementary Figure 77. ^{1}H NMR Spectrum (400 MHz, THF-d_8) of compound **[^{13}C]19**.

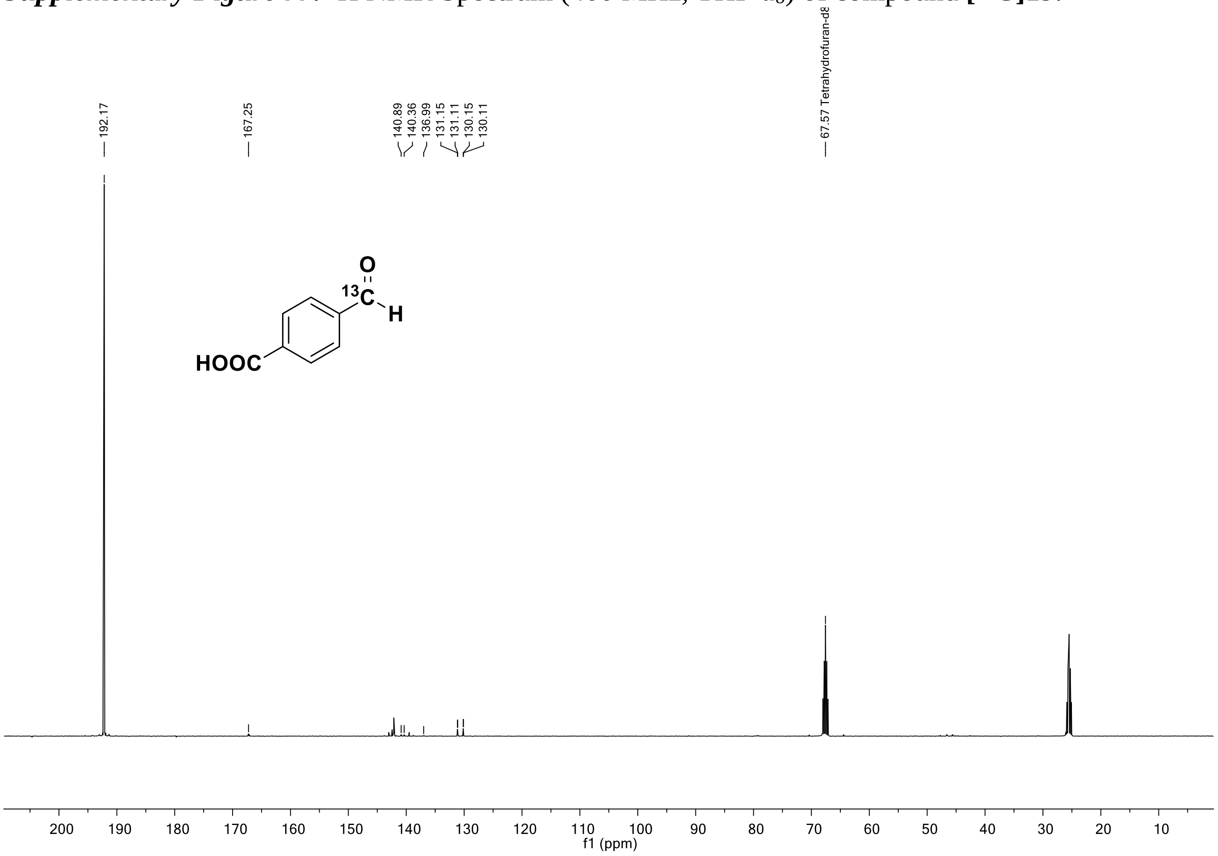

Supplementary Figure 78. ^{13}C NMR Spectrum (100 MHz, THF-d_8) of compound **[^{13}C]19**.

[13C]-(3,5-bis(trifluoromethyl)phenyl)(3,4,5-trimethoxyphenyl)methanone ([13C]20)

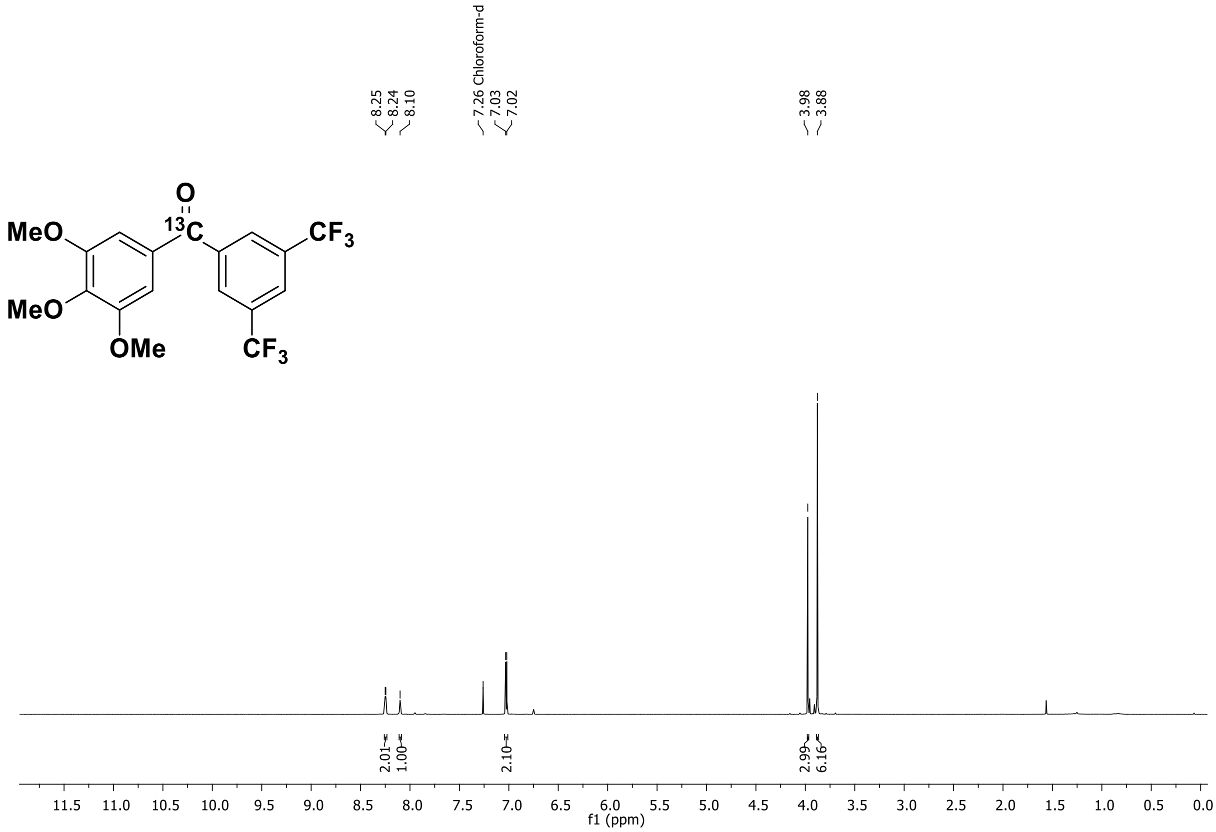

Supplementary Figure 79. ^{1}H NMR Spectrum (400 MHz, $CDCl_3$) of compound **[^{13}C]20**.

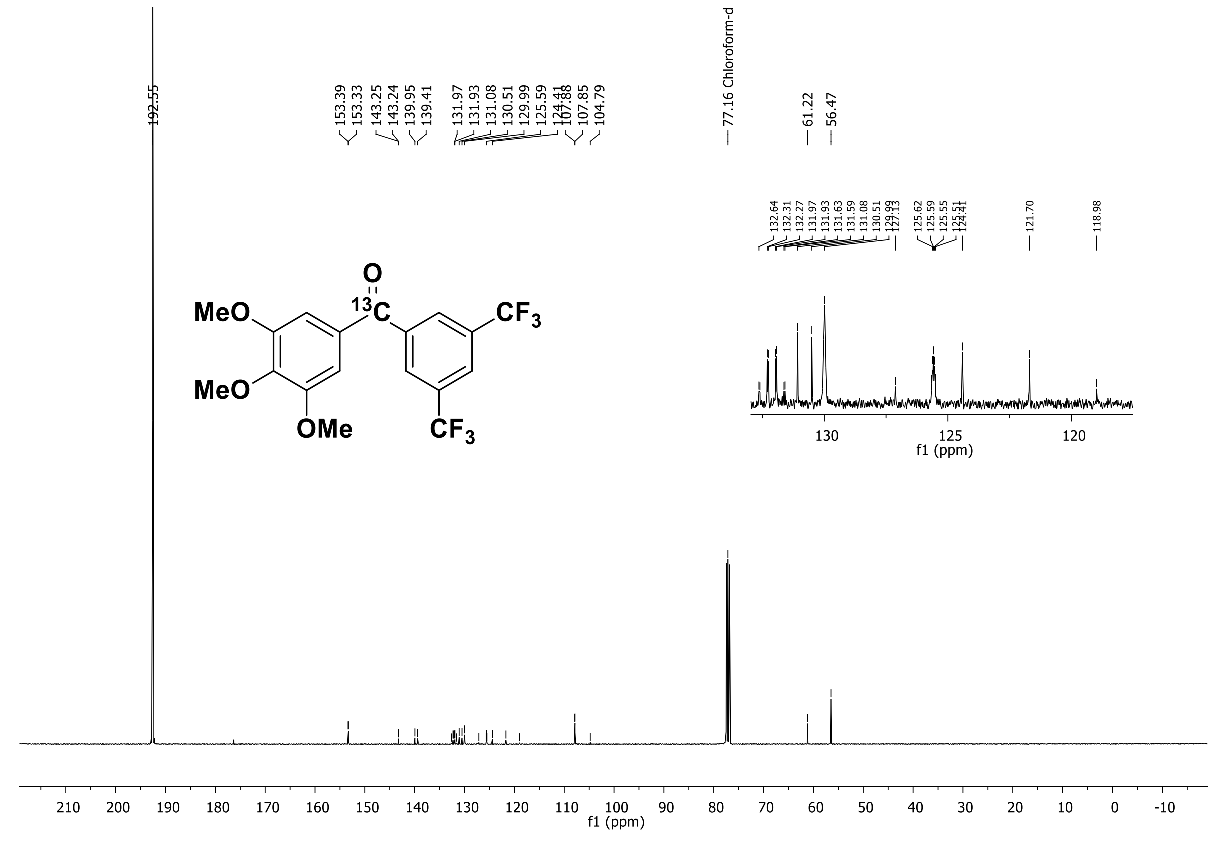

Supplementary Figure 80. ^{13}C NMR Spectrum (100 MHz, $CDCl_3$) of compound **[^{13}C]20**.

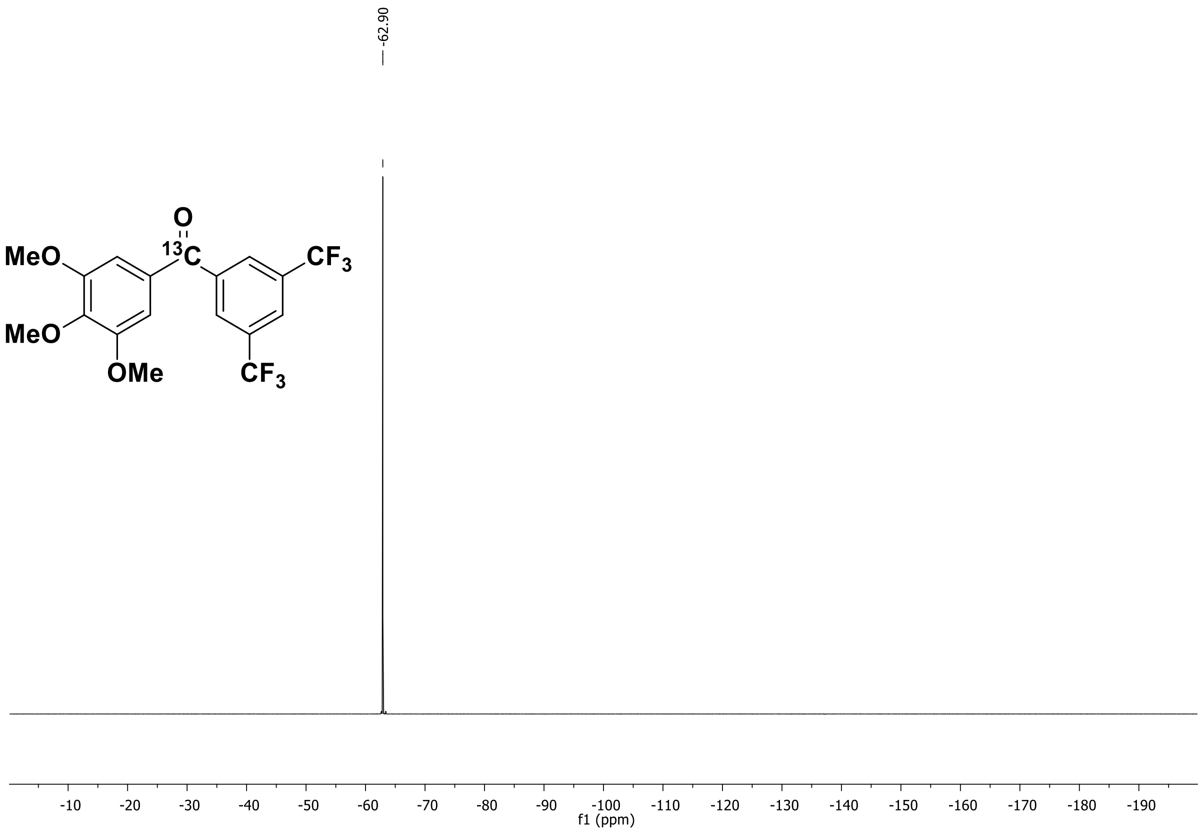

Supplementary Figure 81. ^{19}F NMR Spectrum (376 MHz, $CDCl_3$) of compound **[^{13}C]20**.

[13C]- (2,4-difluorophenyl)(1H-indol-5-yl)methanone ([13C]21)

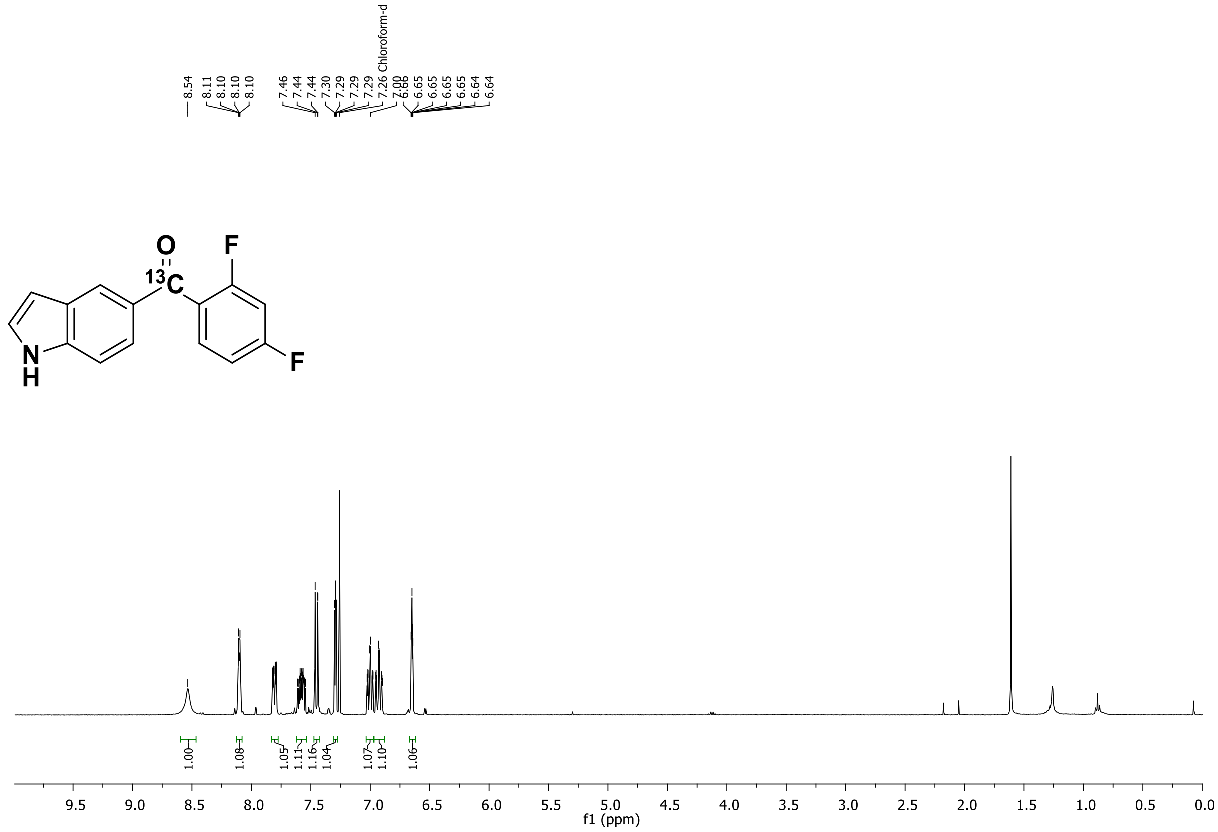

Supplementary Figure 82. ^{1}H NMR Spectrum (400 MHz, $CDCl_3$) of compound **[13C]21**.

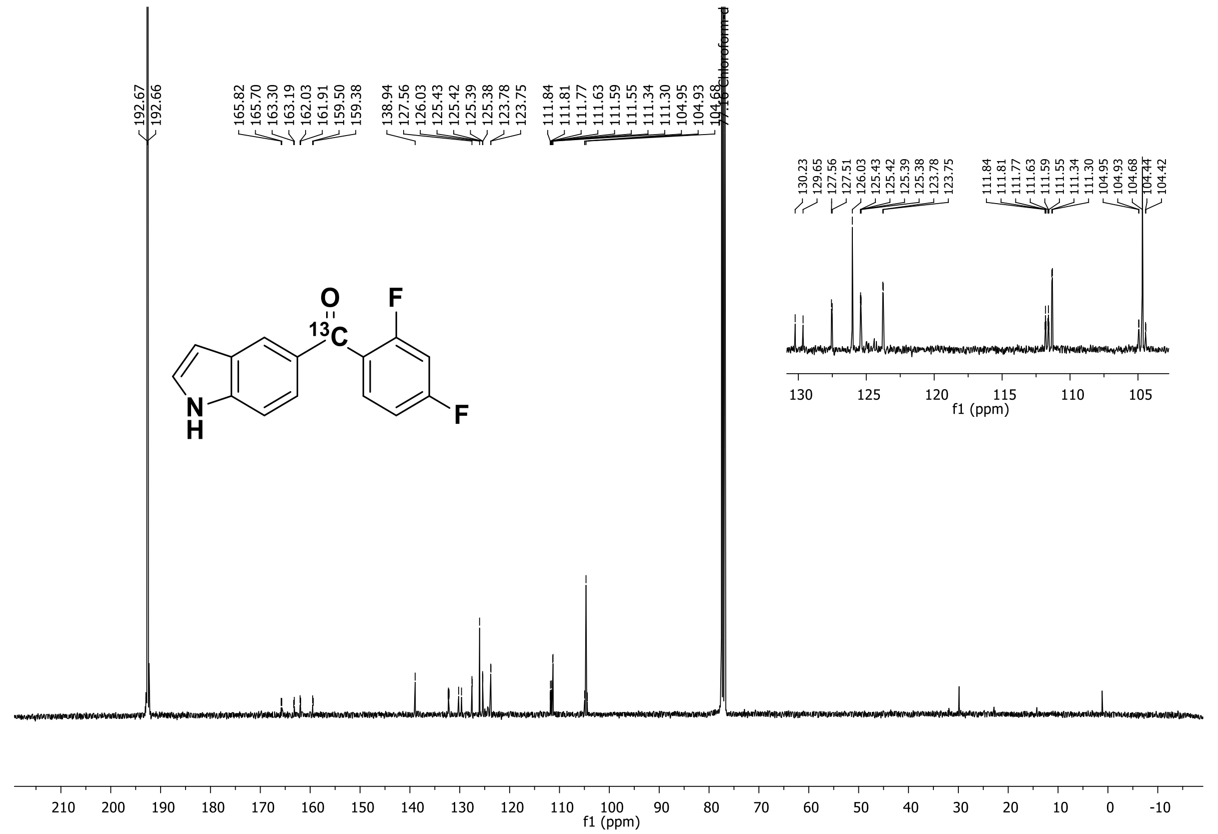

Supplementary Figure 83. ^{13}C NMR Spectrum (100 MHz, $CDCl_3$) of compound **[13C]21**.

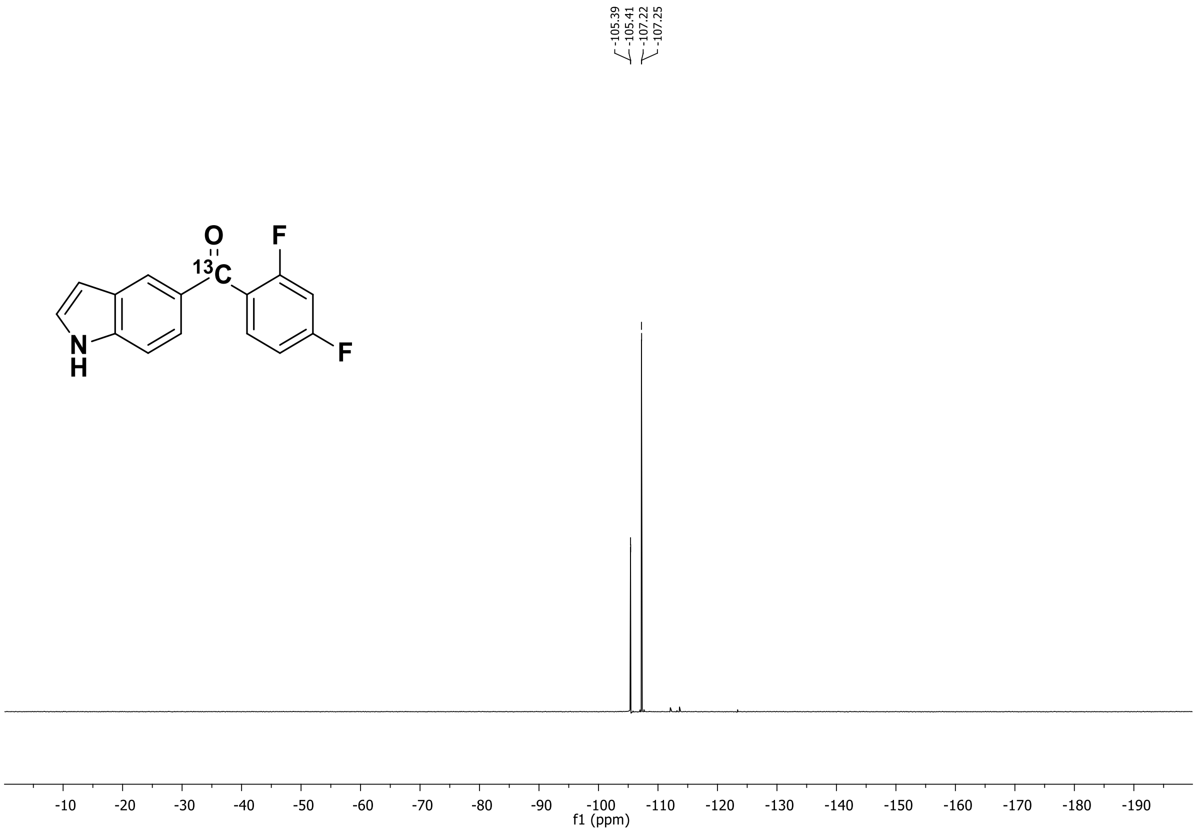

Supplementary Figure 84. ^{19}F NMR Spectrum (376 MHz, $CDCl_3$) of compound **[^{13}C]21**.

[13C]-furan-3-yl(4-methoxyphenyl)methanone ([13C]22)

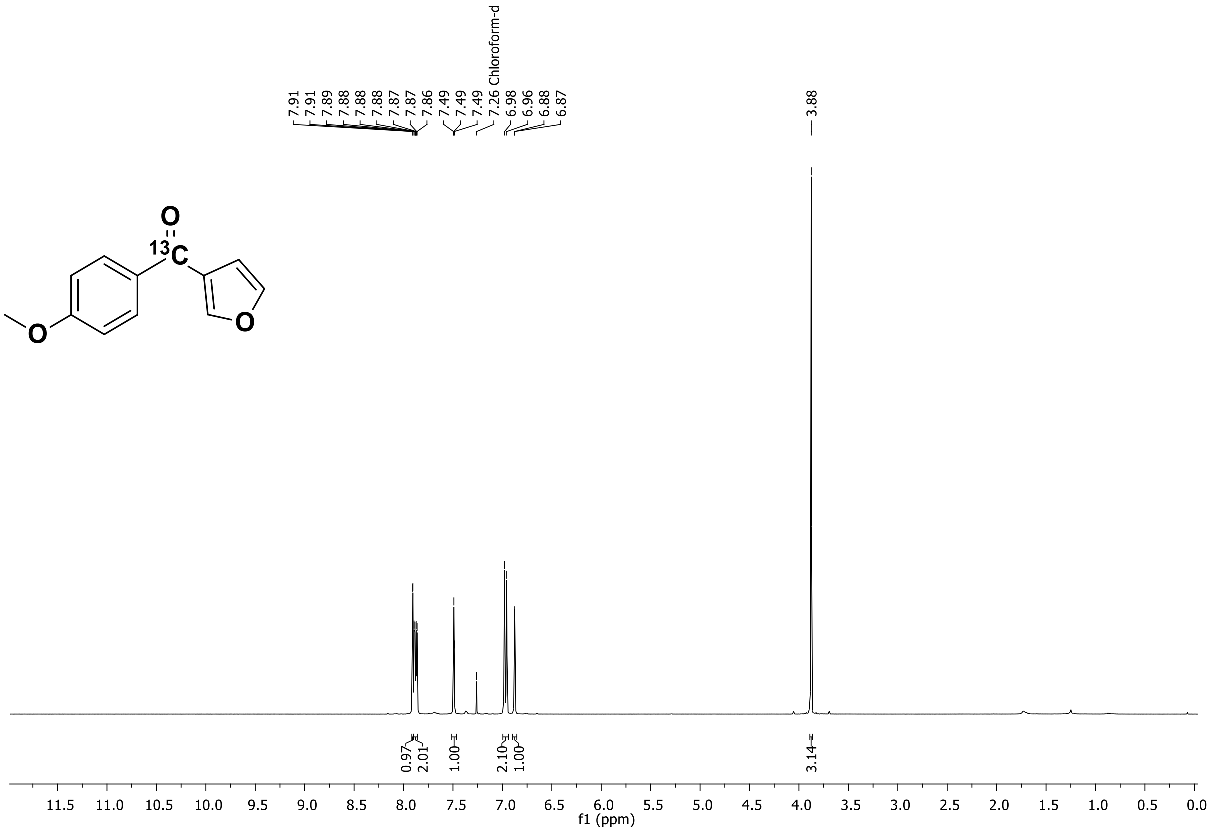

Supplementary Figure 85. ^{1}H NMR Spectrum (400 MHz, $CDCl_3$) of compound **[^{13}C]22**.

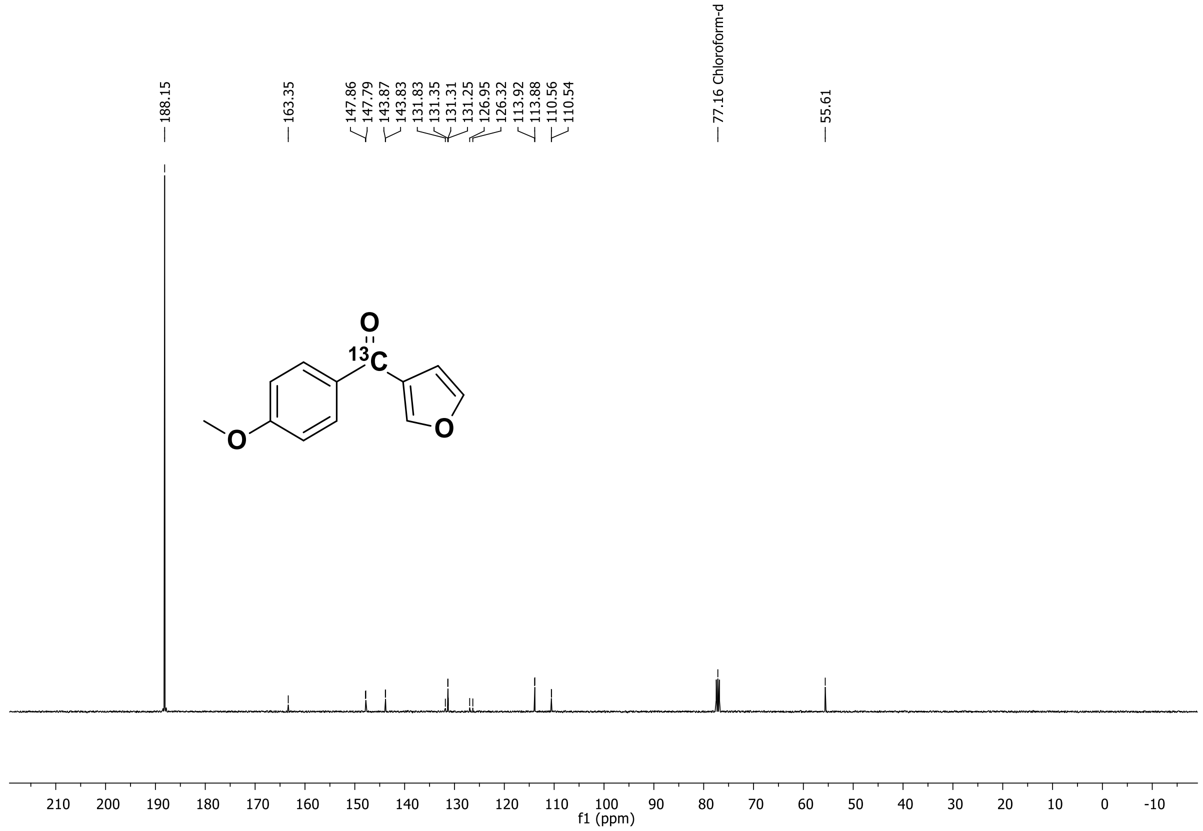

Supplementary Figure 86. ^{13}C NMR Spectrum (100 MHz, $CDCl_3$) of compound **[^{13}C]22**.

[^{13}C] (4-methoxyphenyl)(phenyl)methanone ([^{13}C]23)

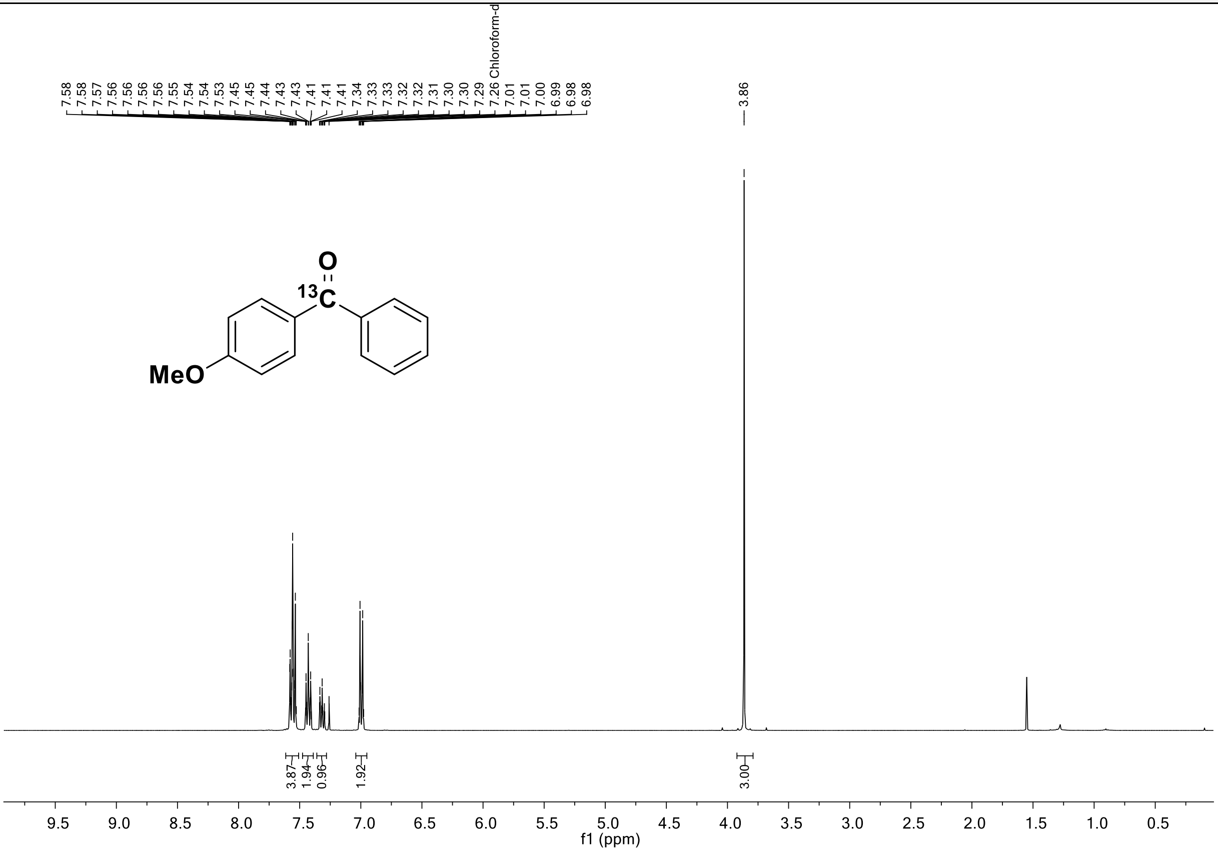

Supplementary Figure 87. ^{1}H NMR Spectrum (400 MHz, $CDCl_3$) of compound **[^{13}C]23**.

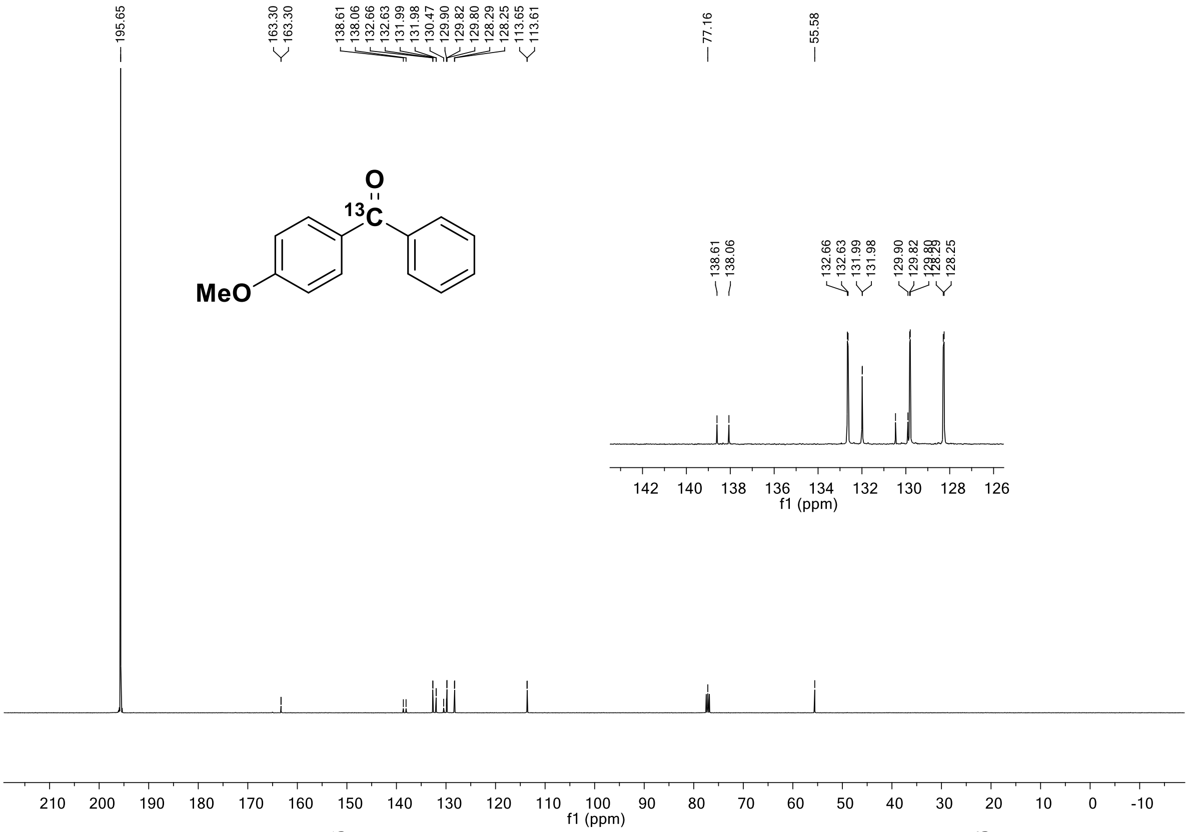

Supplementary Figure 88. ^{13}C NMR Spectrum (100 MHz, $CDCl_3$) of compound **[^{13}C]23**.

[¹³C] (4-chlorophenyl)(4-fluorophenyl)methanone ([¹³C]24)

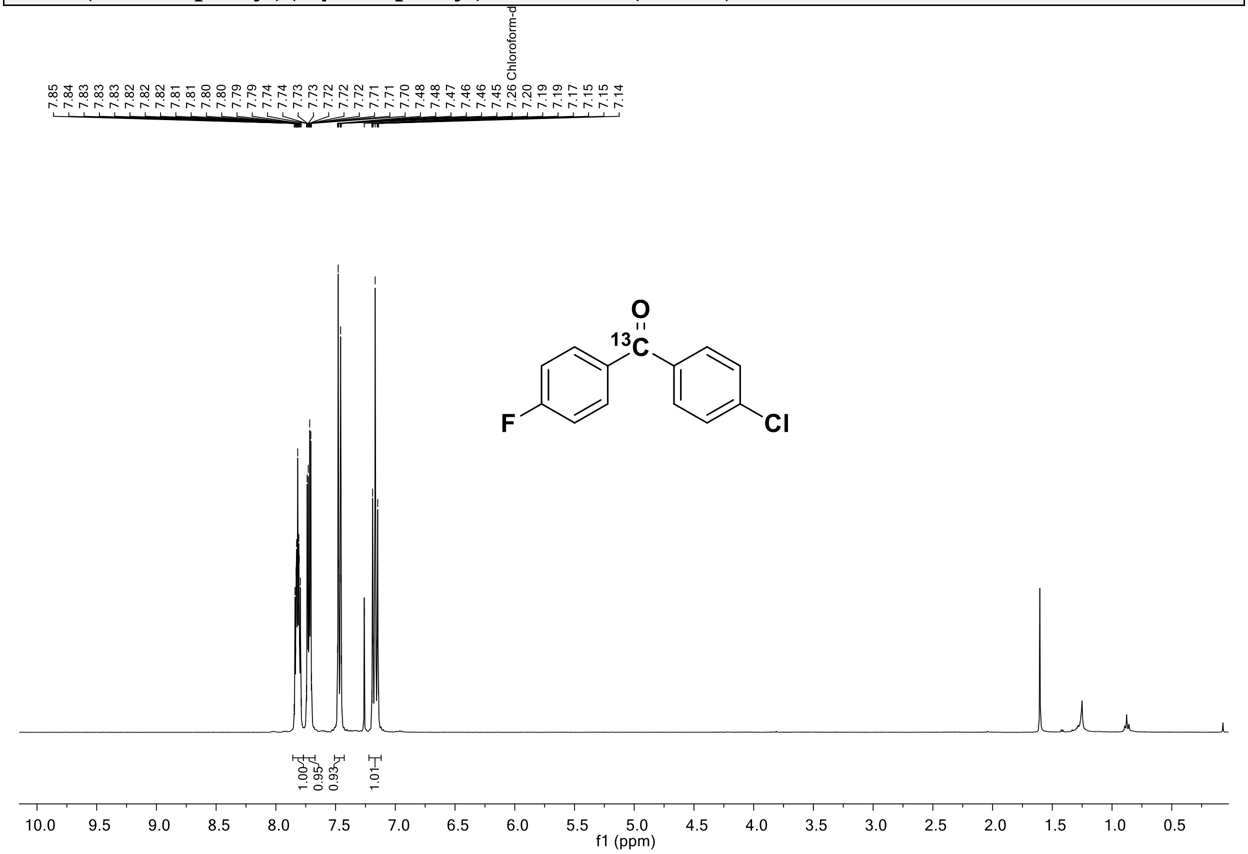

Supplementary Figure 89. ^{1}H NMR Spectrum (400 MHz, $CDCl_3$) of compound **[^{13}C]24**.

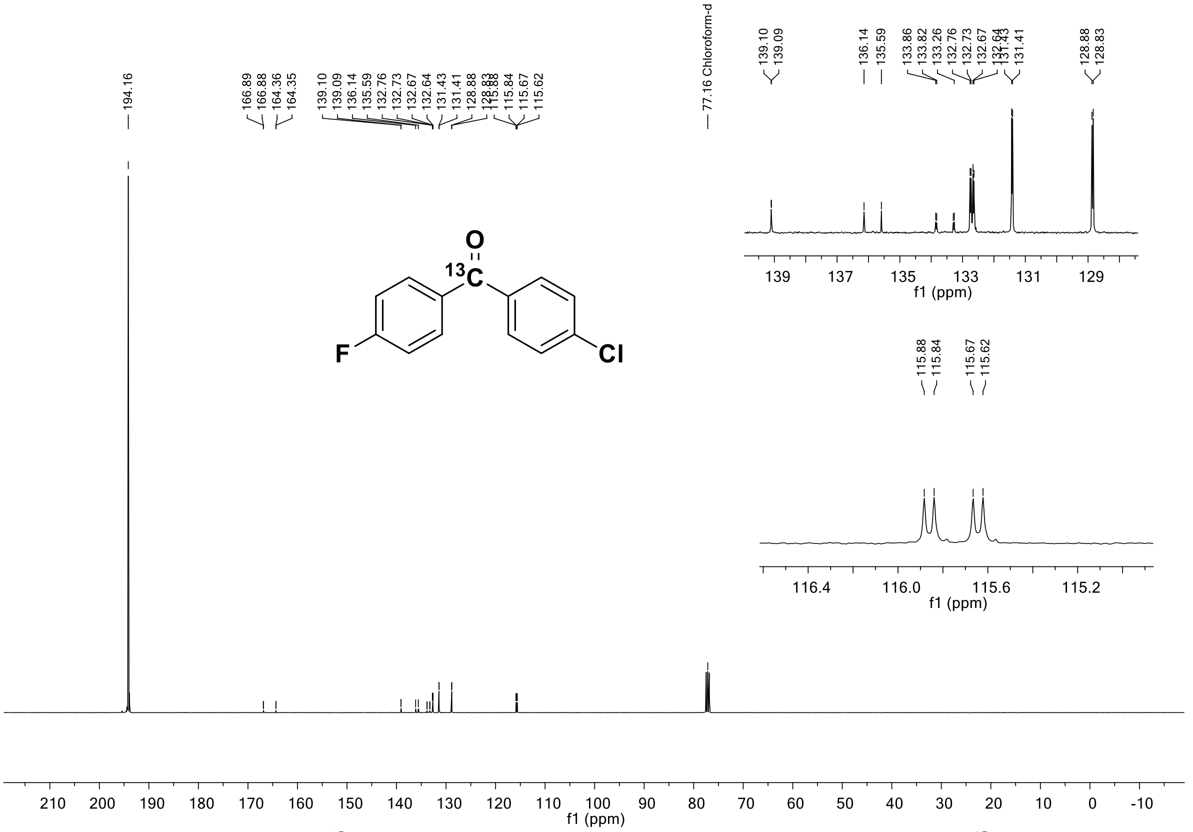

Supplementary Figure 90. ^{13}C NMR Spectrum (100 MHz, $CDCl_3$) of compound **[^{13}C]24**.

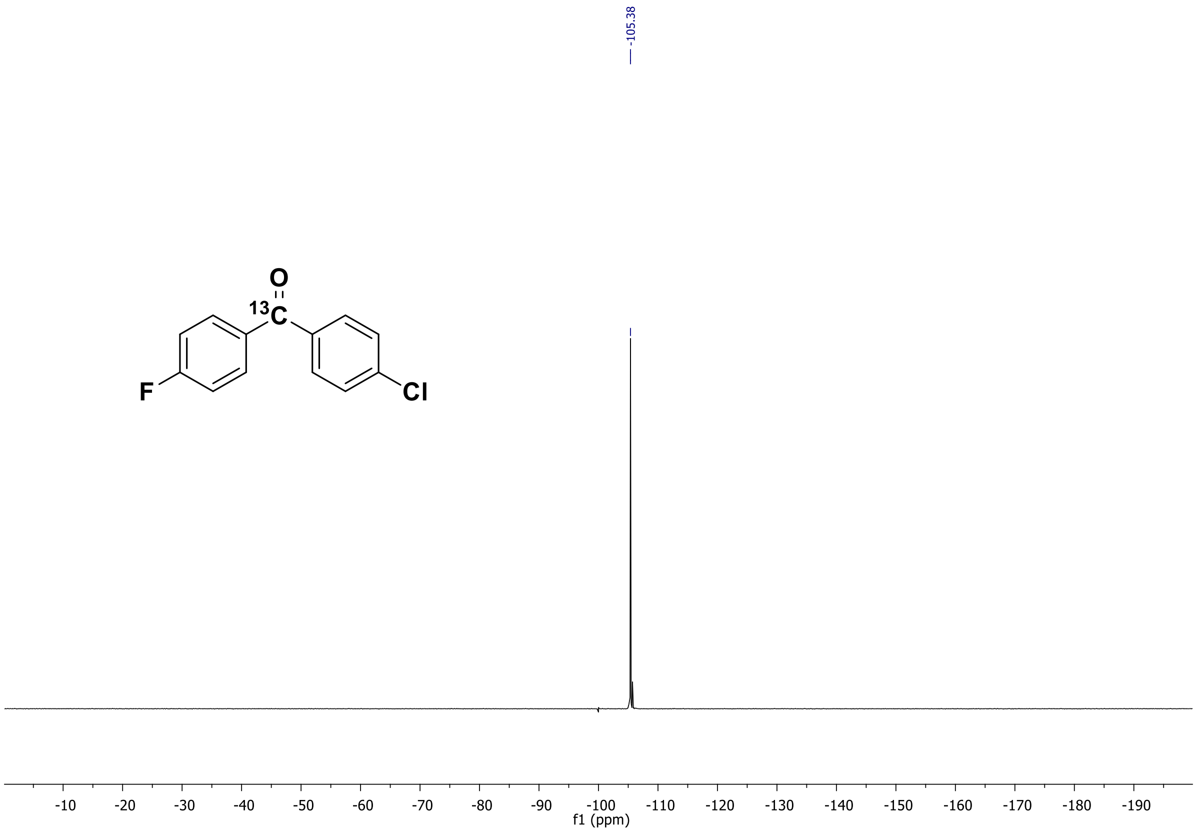

Supplementary Figure 91. ^{19}F NMR Spectrum (376 MHz, $CDCl_3$) of compound **[^{13}C]24**.

[^{13}C]-4-(4-phenoxybenzoyl)benzonitrile ([^{13}C]25)

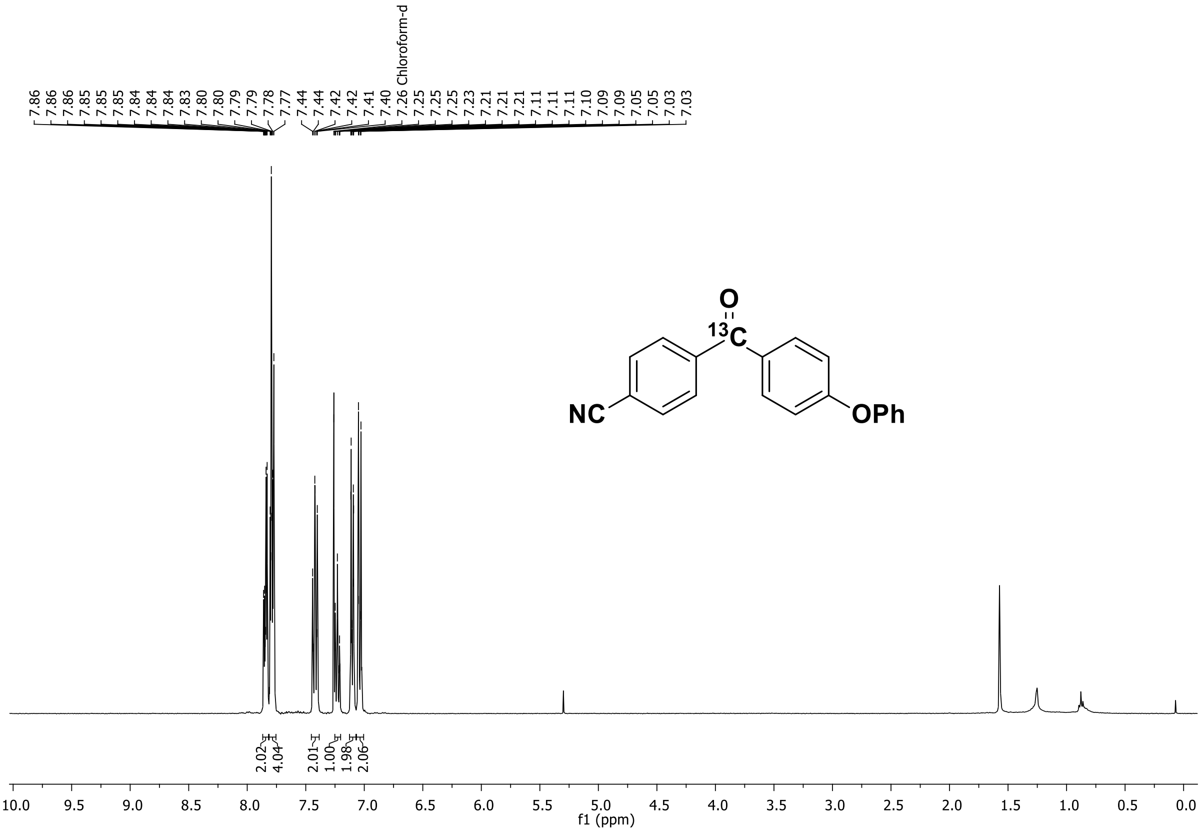

Supplementary Figure 92. ^{1}H NMR Spectrum (400 MHz, $CDCl_3$) of compound **[^{13}C]25**.

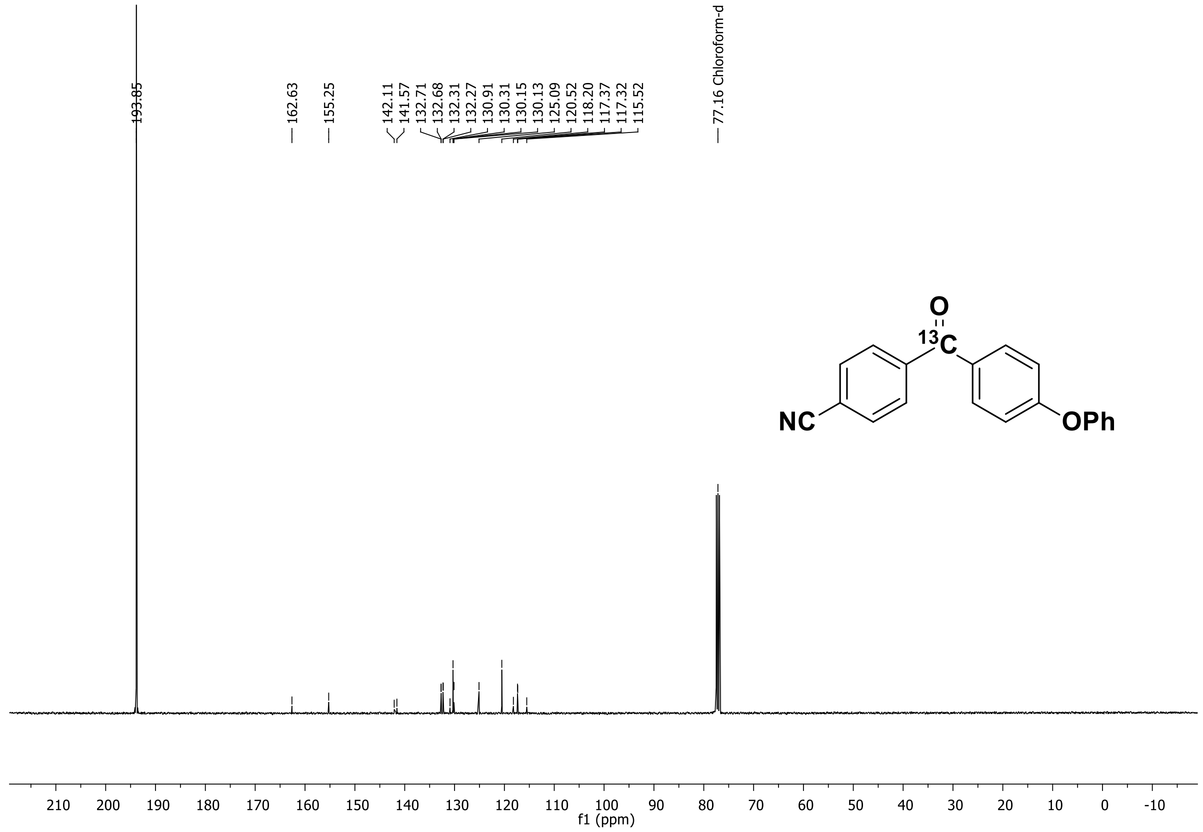

Supplementary Figure 93. ^{13}C NMR Spectrum (100 MHz, $CDCl_3$) of compound **[^{13}C]25**.

[^{13}C] benzoic acid ([^{13}C]26)

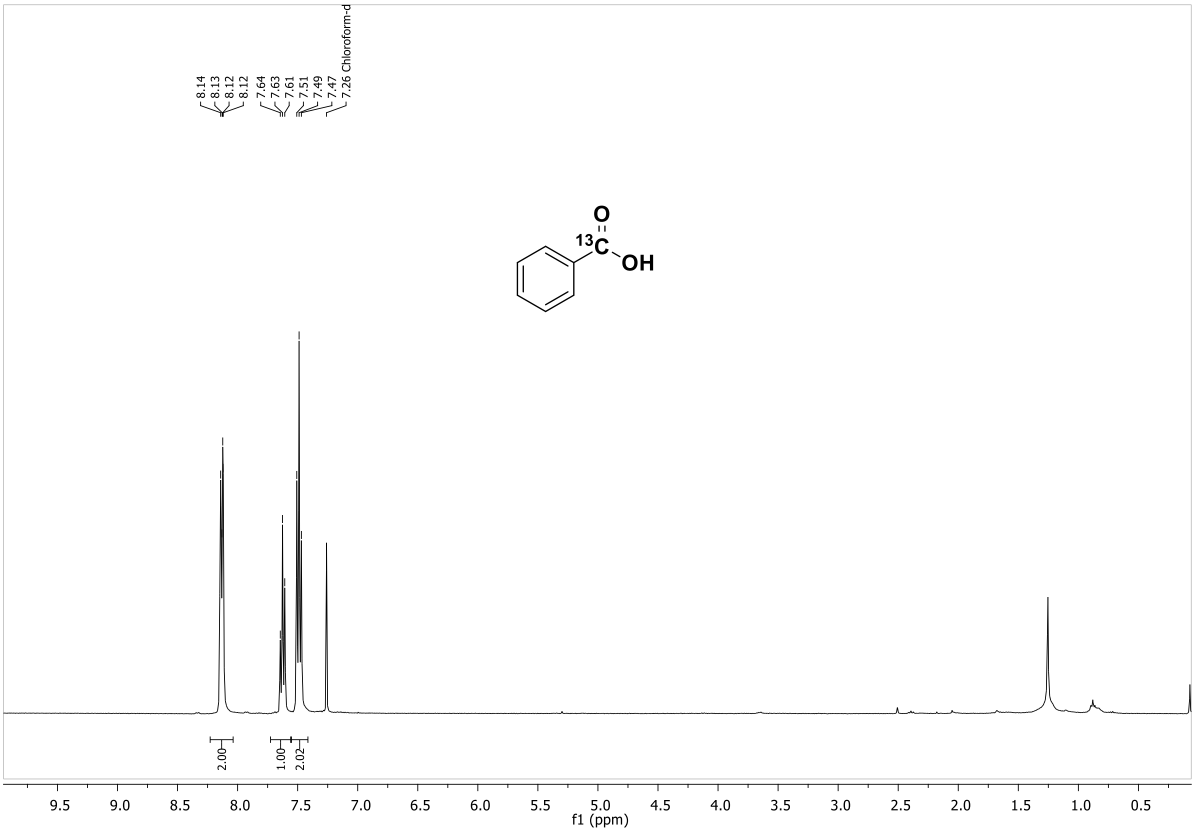

Supplementary Figure 94. ^{1}H NMR Spectrum (400 MHz, $CDCl_3$) of compound **[^{13}C]26**.

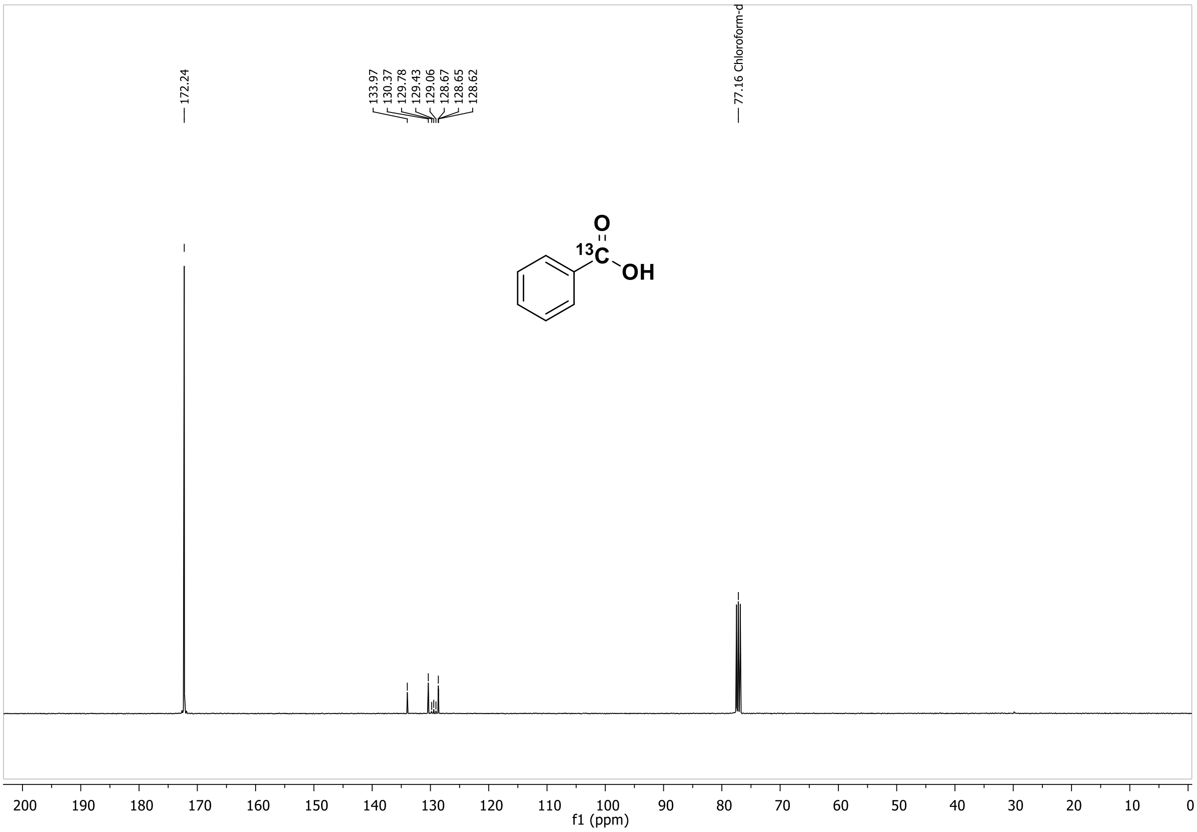

Supplementary Figure 95. ^{13}C NMR Spectrum (100 MHz, $CDCl_3$) of compound **[^{13}C]26**.

[^{13}C]-4-methoxybenzoic acid ([^{13}C]27)

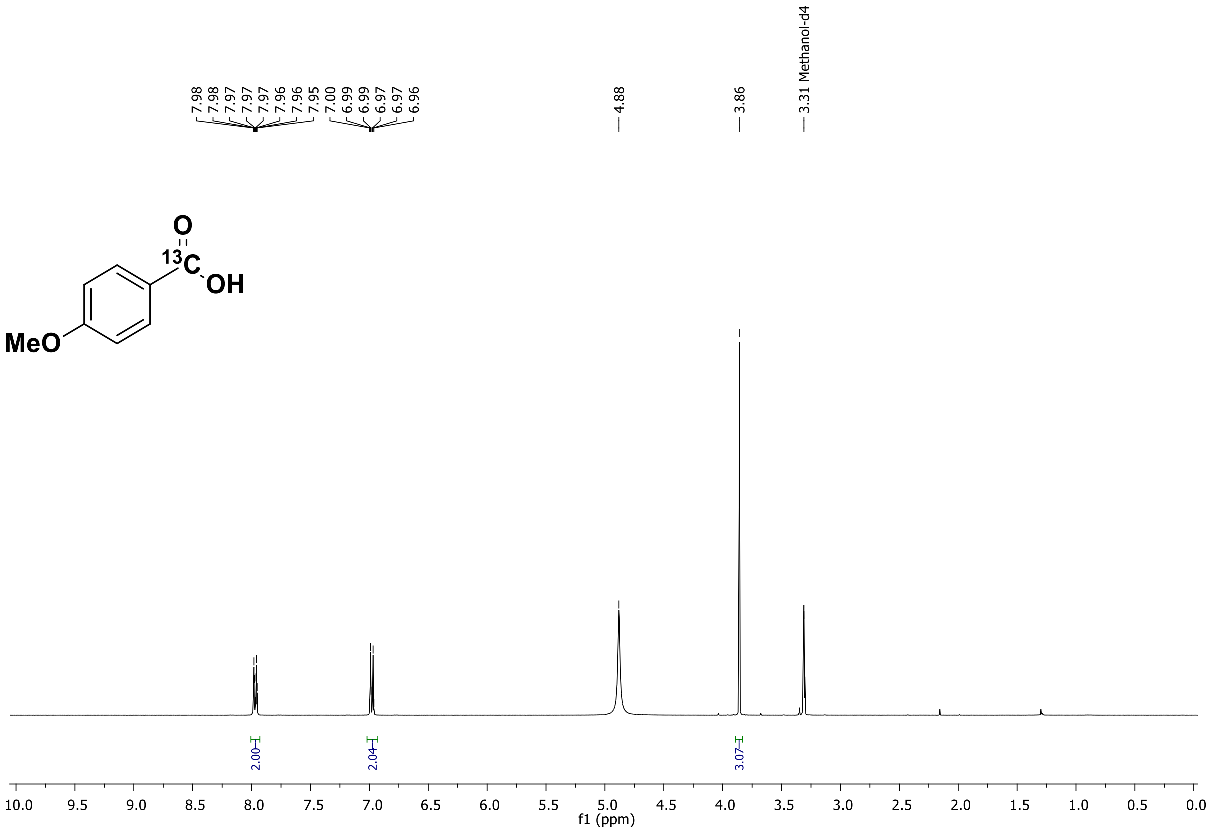

Supplementary Figure 96. ^{1}H NMR Spectrum (400 MHz, MeOD-d_4) of compound **[^{13}C]27**.

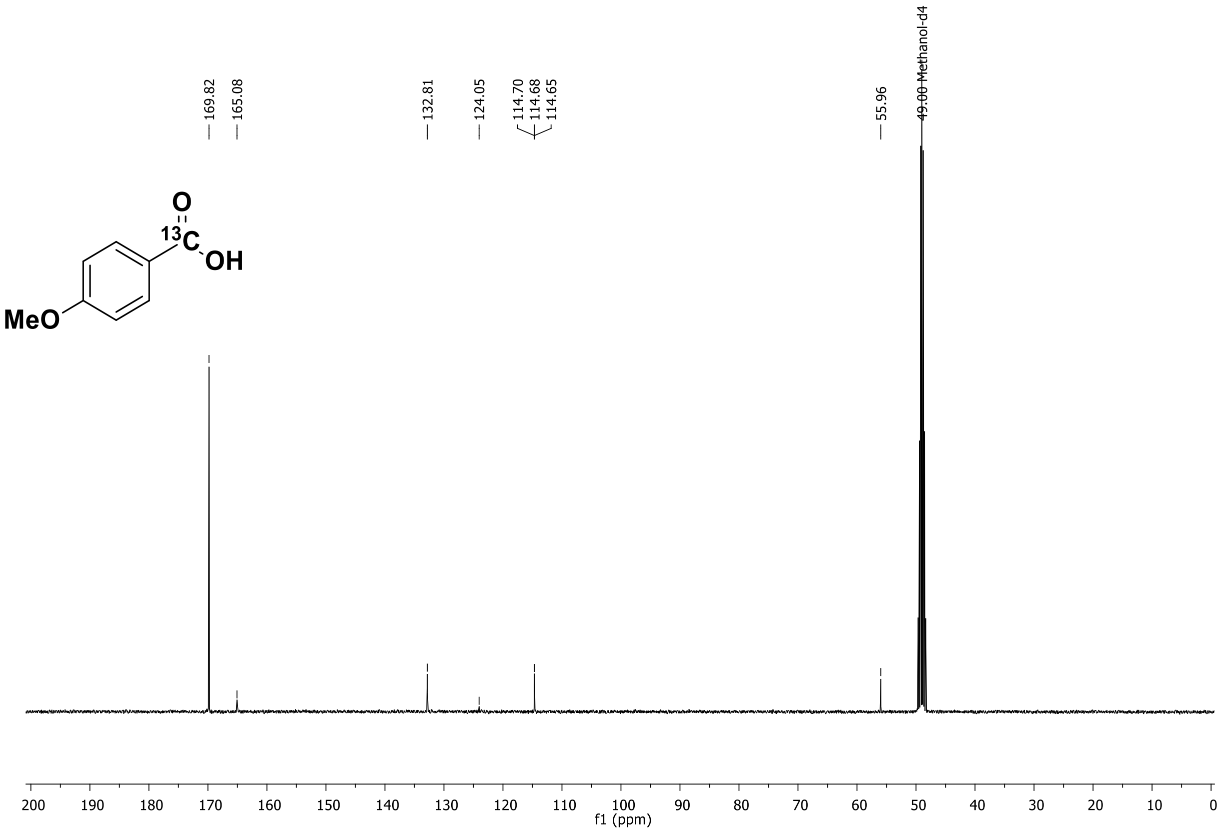

Supplementary Figure 97. ^{13}C NMR Spectrum (100 MHz, MeOD-d_4) of compound **[^{13}C]27**.

[13C]- 2-fluorobenzoic acid ([13C]28)

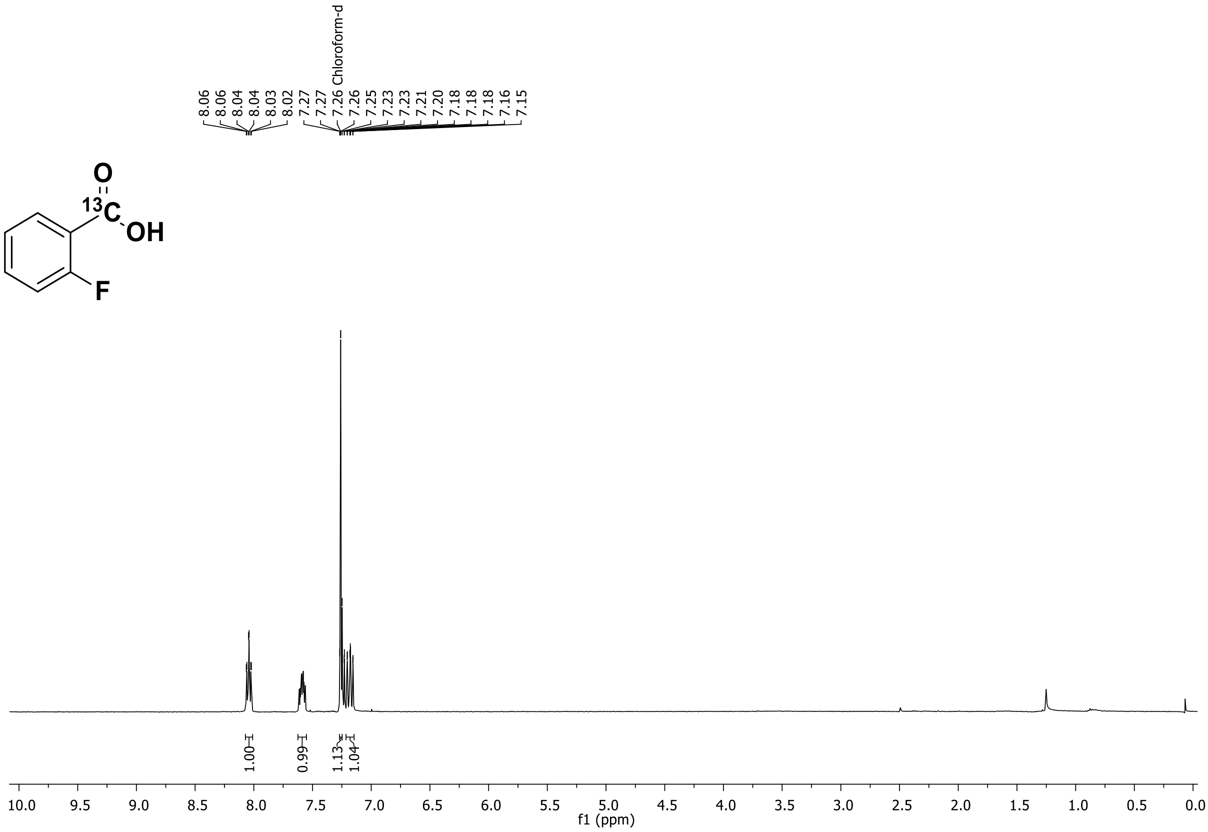

Supplementary Figure 98. ^{1}H NMR Spectrum (400 MHz, $CDCl_3$) of compound **[^{13}C]28**.

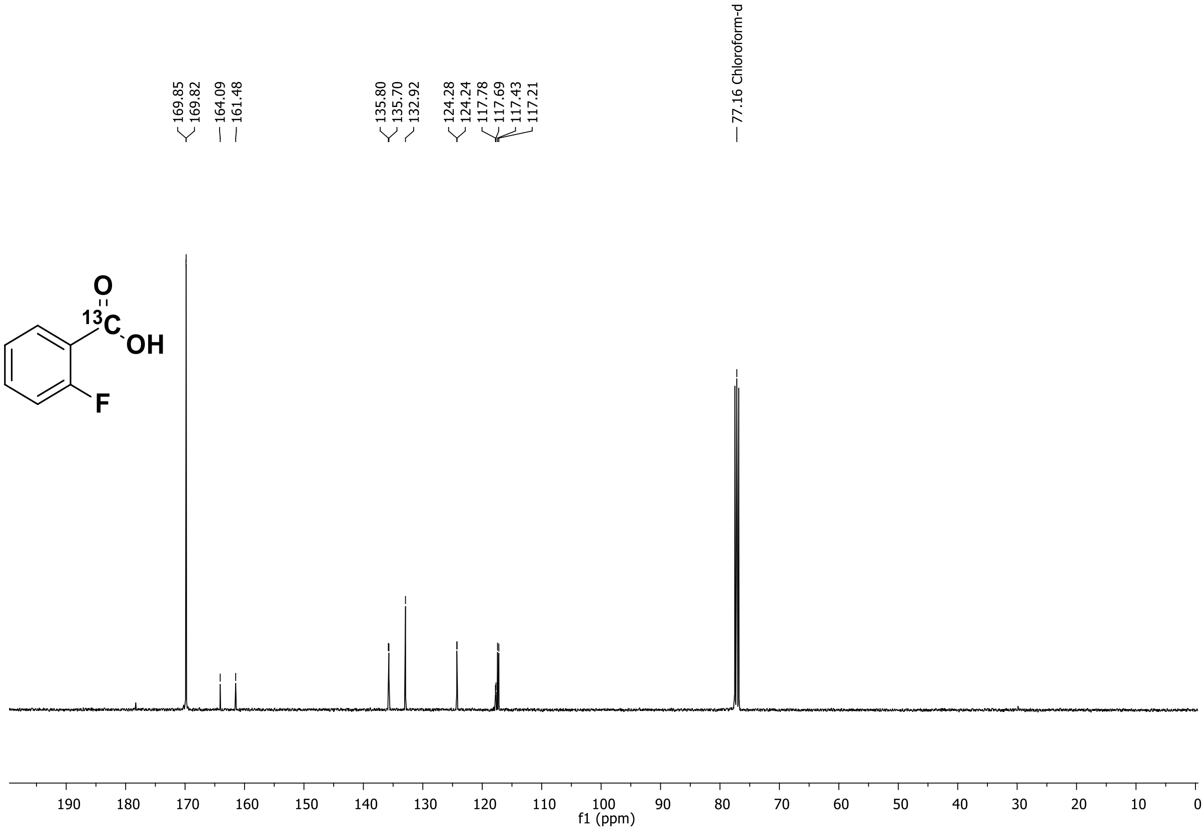

Supplementary Figure 99. ^{13}C NMR Spectrum (100 MHz, $CDCl_3$) of compound **[^{13}C]28**.

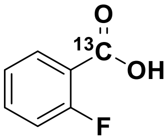

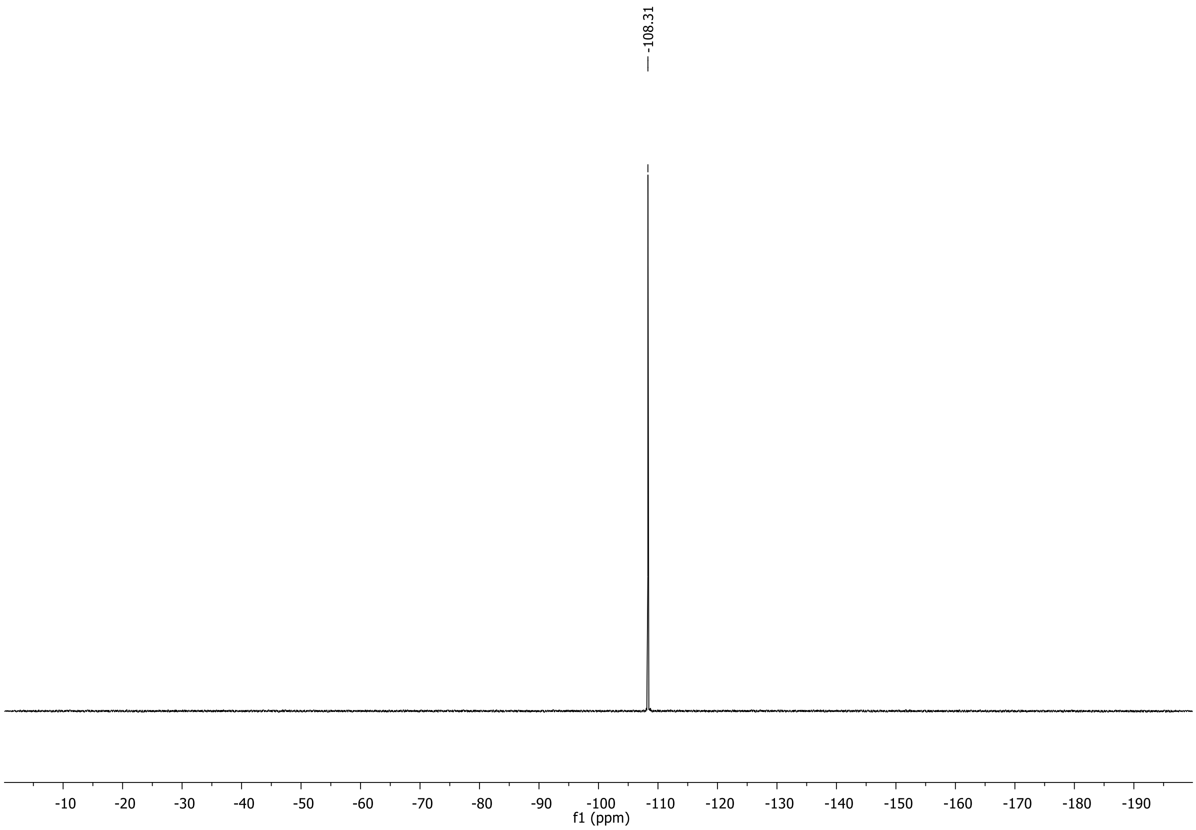

Supplementary Figure 100. ^{19}F NMR Spectrum (376 MHz, $CDCl_3$) of compound **[^{13}C]28**.

[^{13}C]- 4-(tert-butyl)benzoic acid ([^{13}C]29)

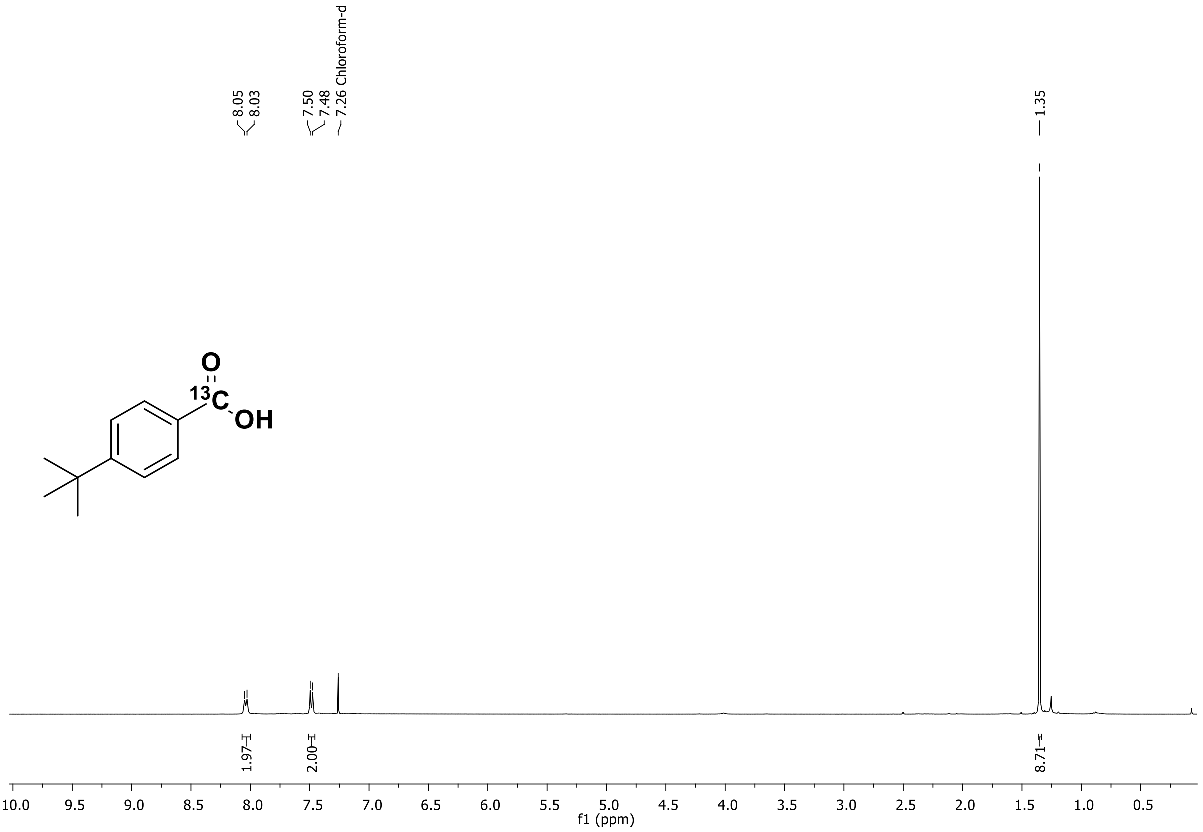

Supplementary Figure 101. ^{1}H NMR Spectrum (400 MHz, $CDCl_3$) of compound **[^{13}C]29**.

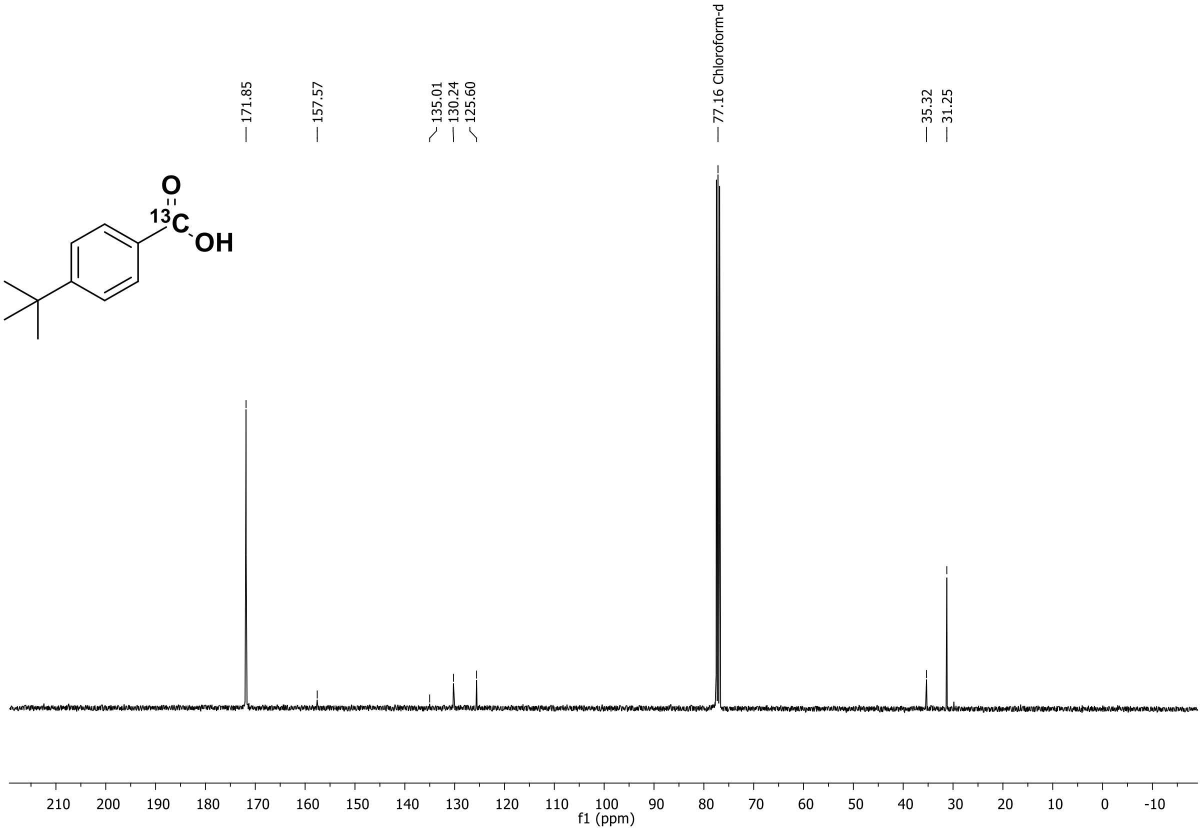

Supplementary Figure 102. ^{13}C NMR Spectrum (100 MHz, $CDCl_3$) of compound **[^{13}C]29**.

[^{13}C]-2-phenylacetic acid ([^{13}C]30)

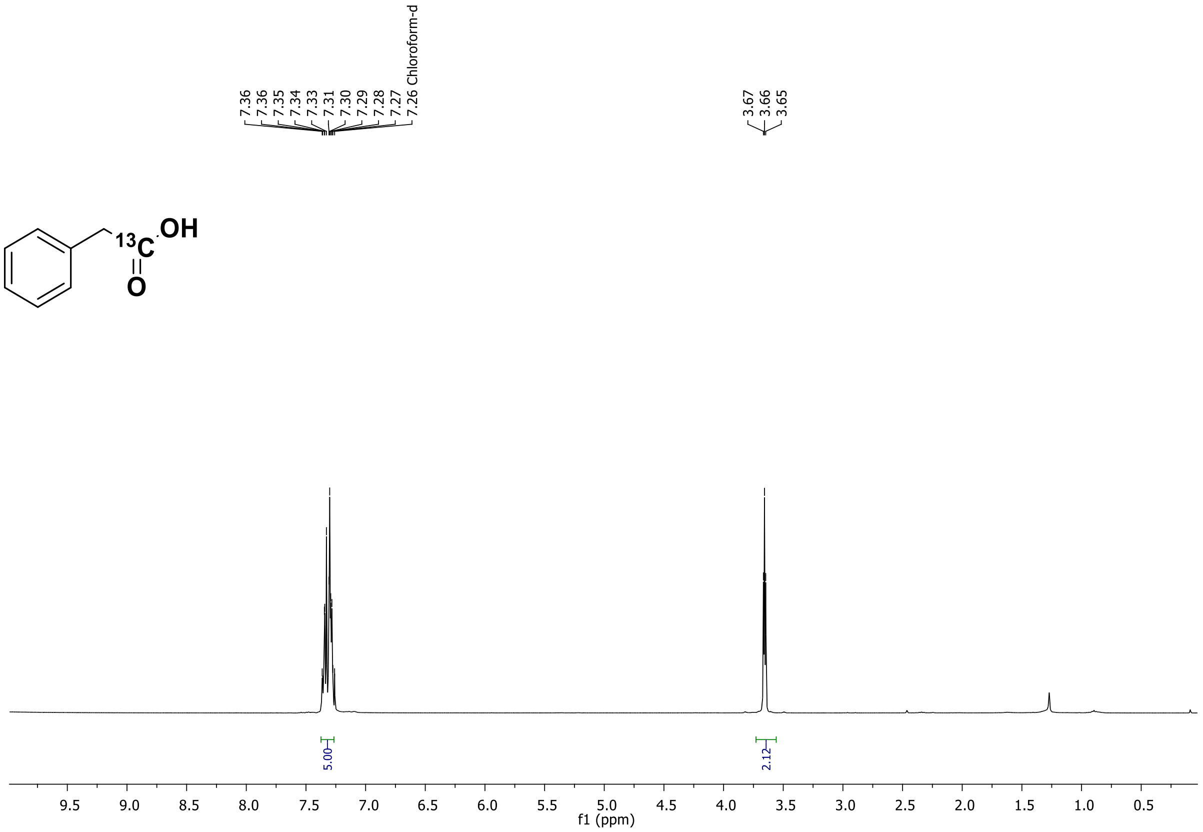

Supplementary Figure 103. ^{1}H NMR Spectrum (400 MHz, $CDCl_3$) of compound **[^{13}C]30**.

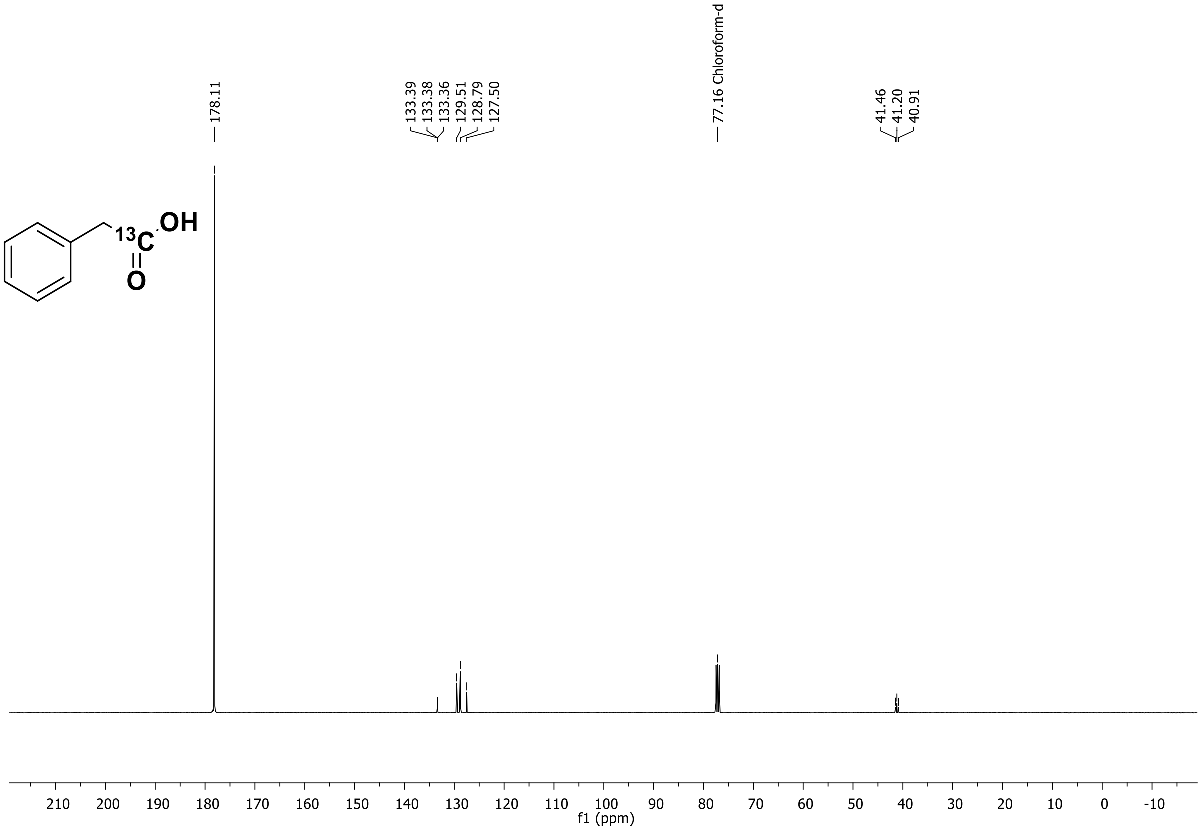

Supplementary Figure 104. ^{13}C NMR Spectrum (100 MHz, $CDCl_3$) of compound **[^{13}C]30**.

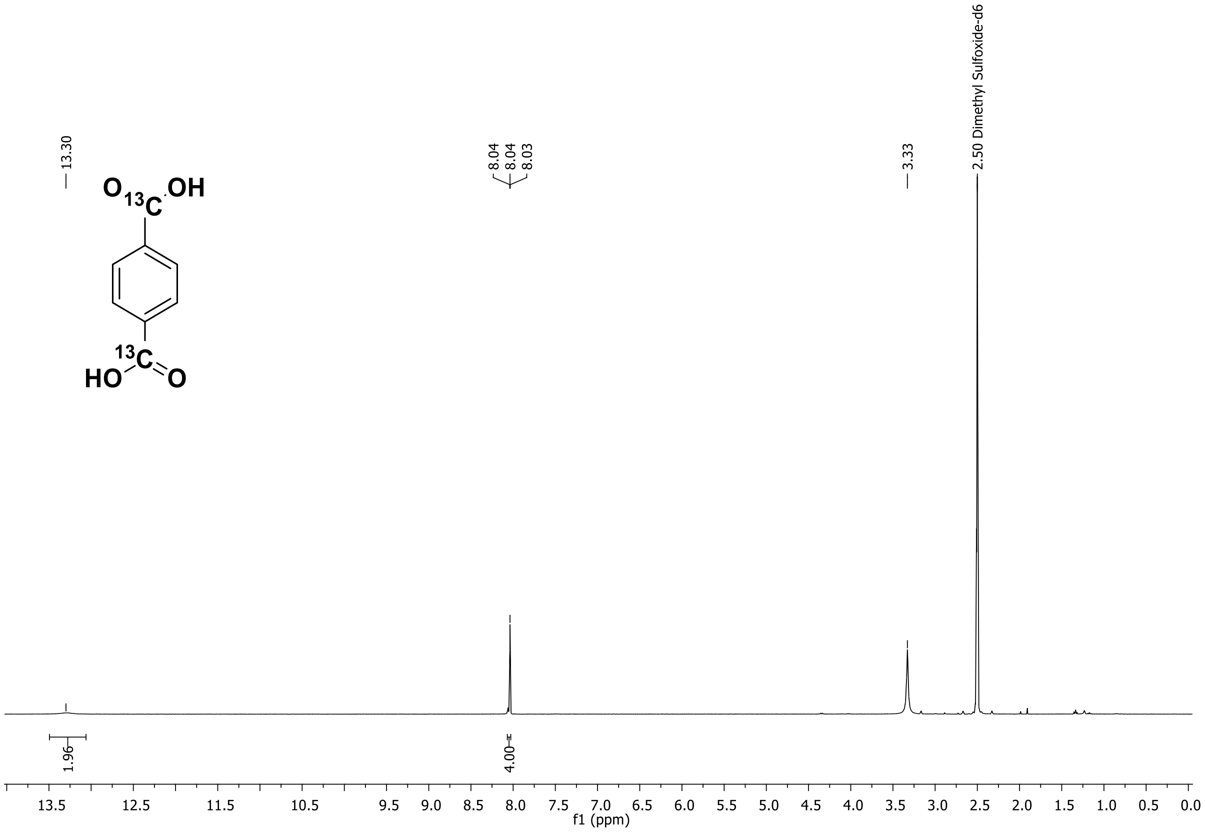

Supplementary Figure 105. ^{1}H NMR Spectrum (400 MHz, DMSO-d_6) of compound **[^{13}C]31**.

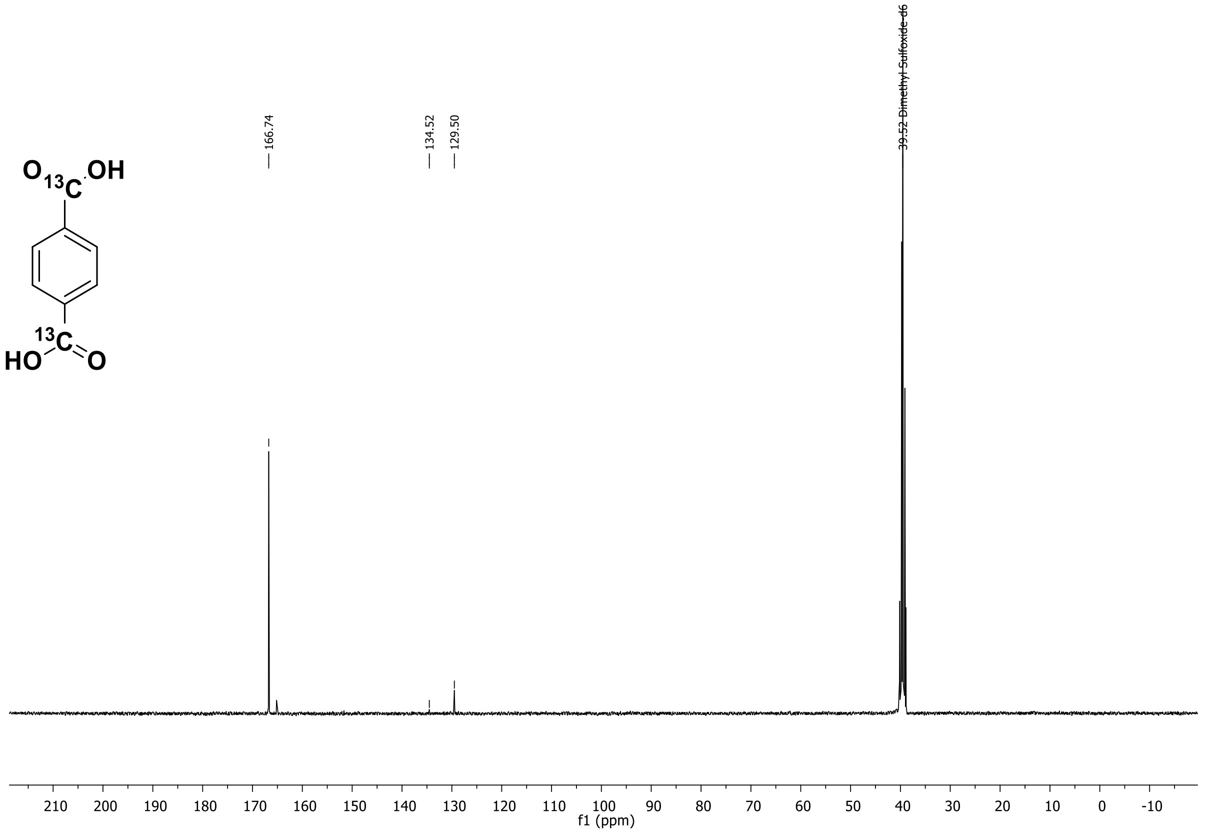

Supplementary Figure 106. ^{13}C NMR Spectrum (100 MHz, DMSO-d_6) of compound **[^{13}C]31**.

[^13C]-4-(3-(4-methoxyphenyl)propioloyl)benzonitrile ([^13C]32)

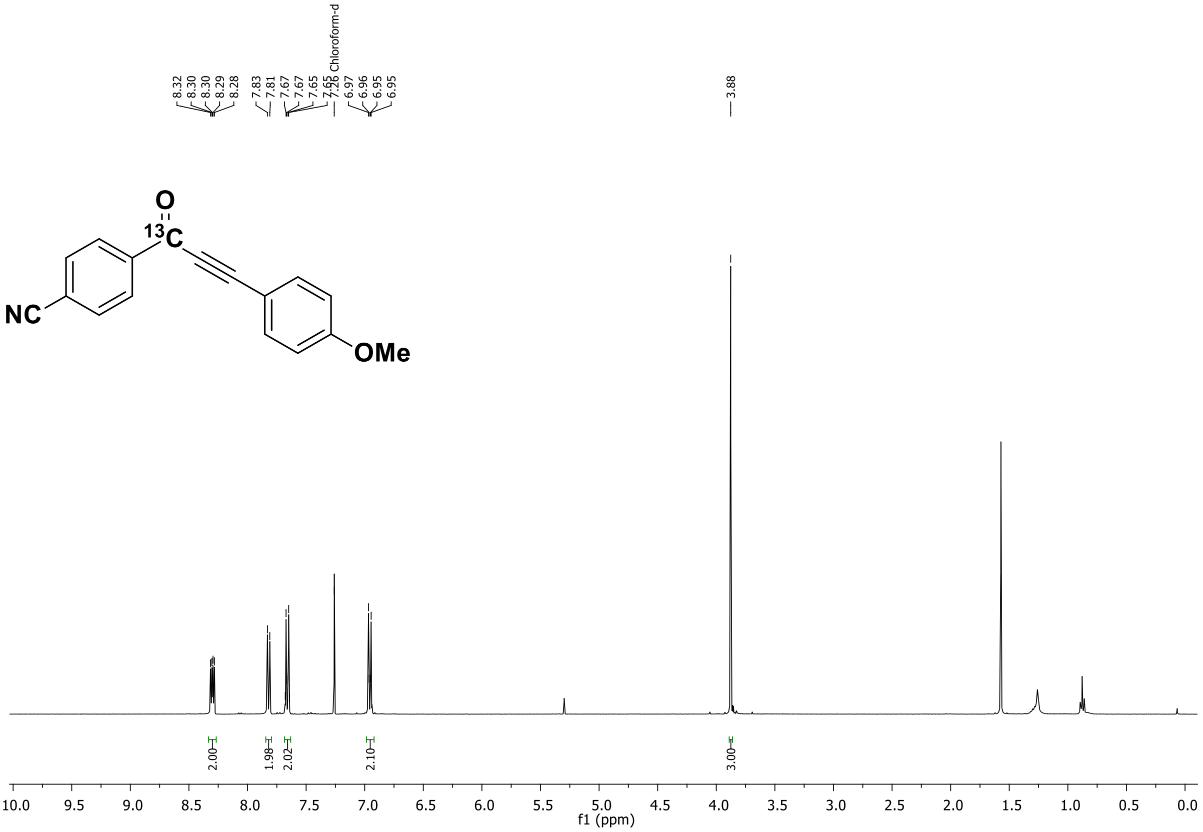

Supplementary Figure 107. ^{1}H NMR Spectrum (400 MHz, $CDCl_3$) of compound **[^{13}C]32**.

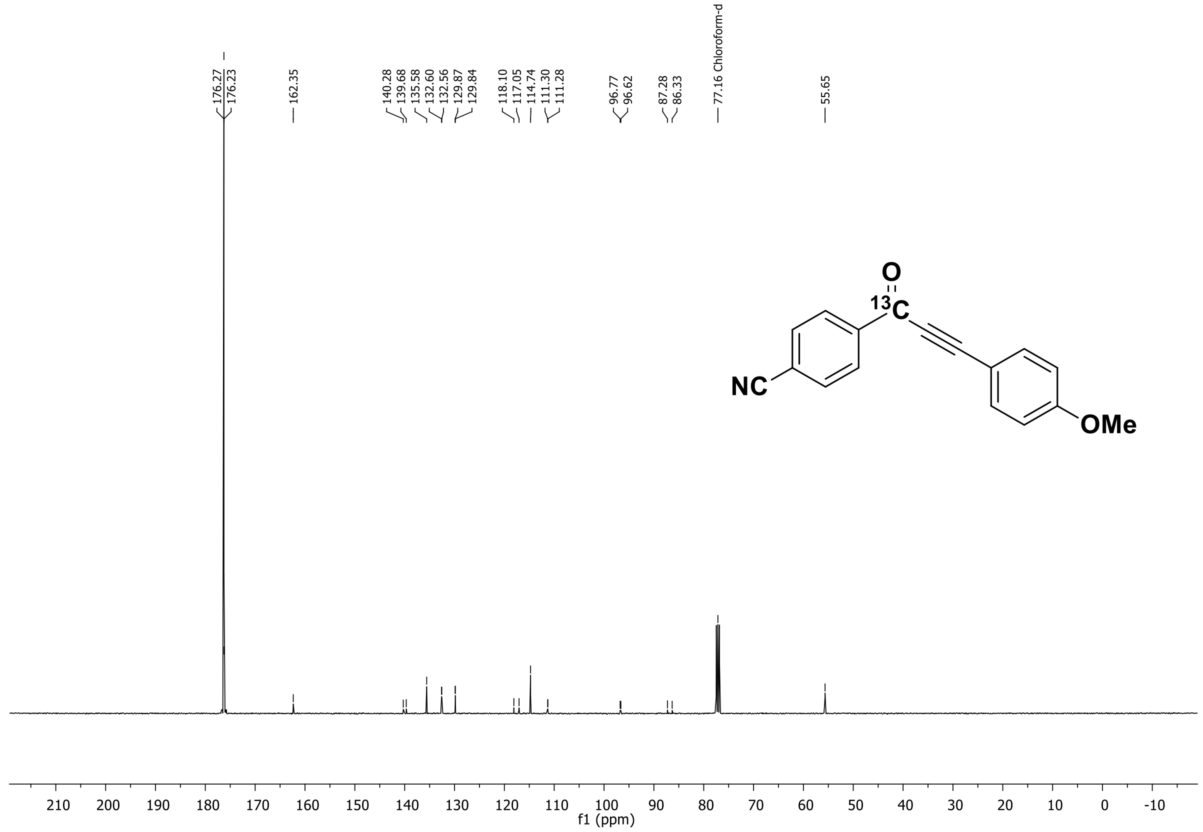

Supplementary Figure 108. ^{13}C NMR Spectrum (100 MHz, $CDCl_3$) of compound **[^{13}C]32**.

[^{13}C]- 1-(4-acetylphenyl)-3-(6-methoxynaphthalen-2-yl)prop-2-yn-1-one ([^{13}C]33)

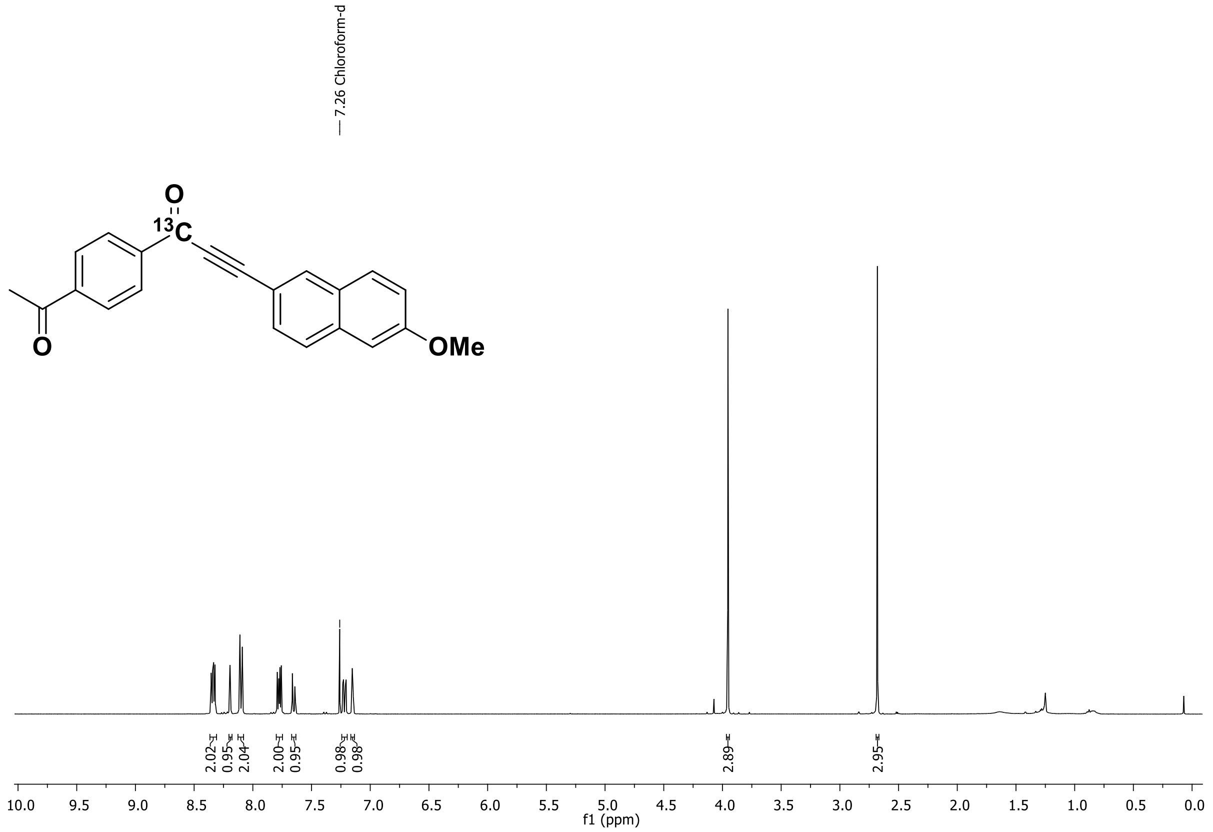

Supplementary Figure 109. ^{1}H NMR Spectrum (400 MHz, $CDCl_3$) of compound **[^{13}C]33**.

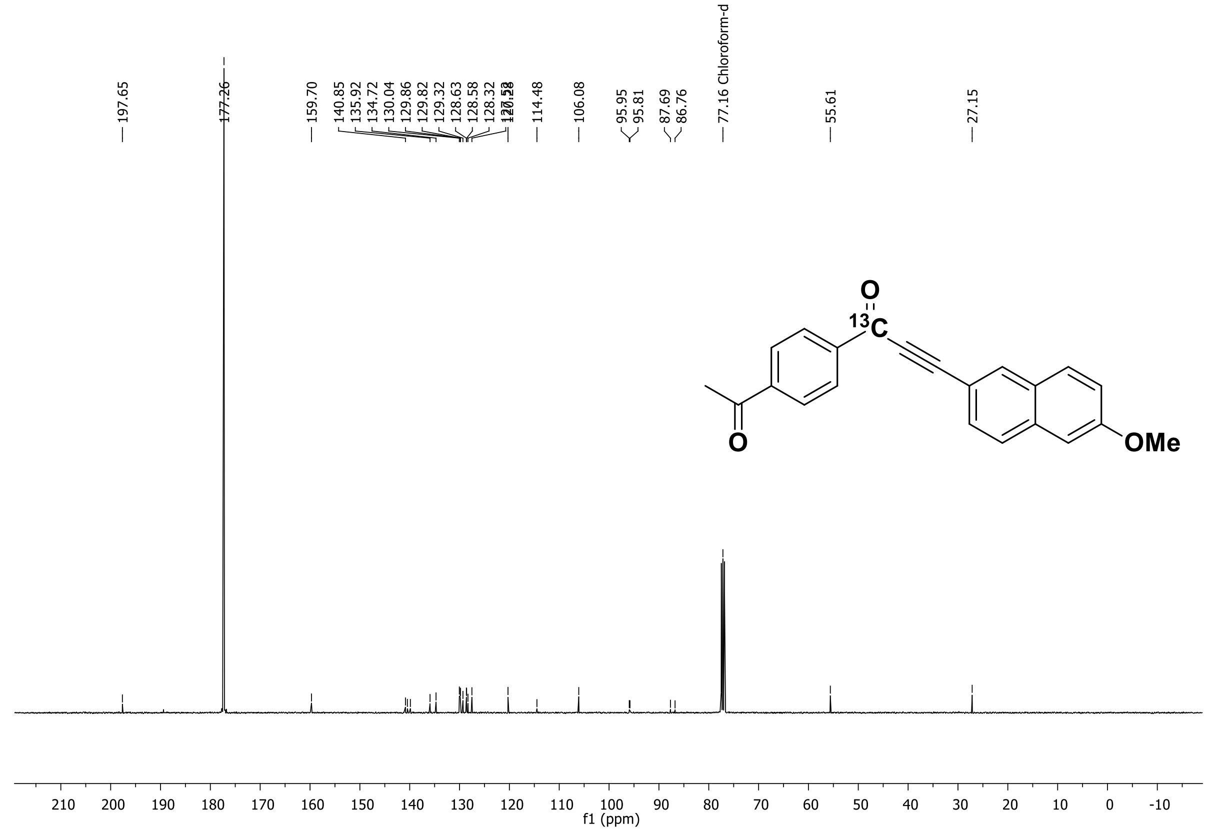

Supplementary Figure 110. ^{13}C NMR Spectrum (100 MHz, $CDCl_3$) of compound **[^{13}C]33**.

[^{13}C]-1,3-diphenylprop-2-yn-1-one ([^{13}C]34)

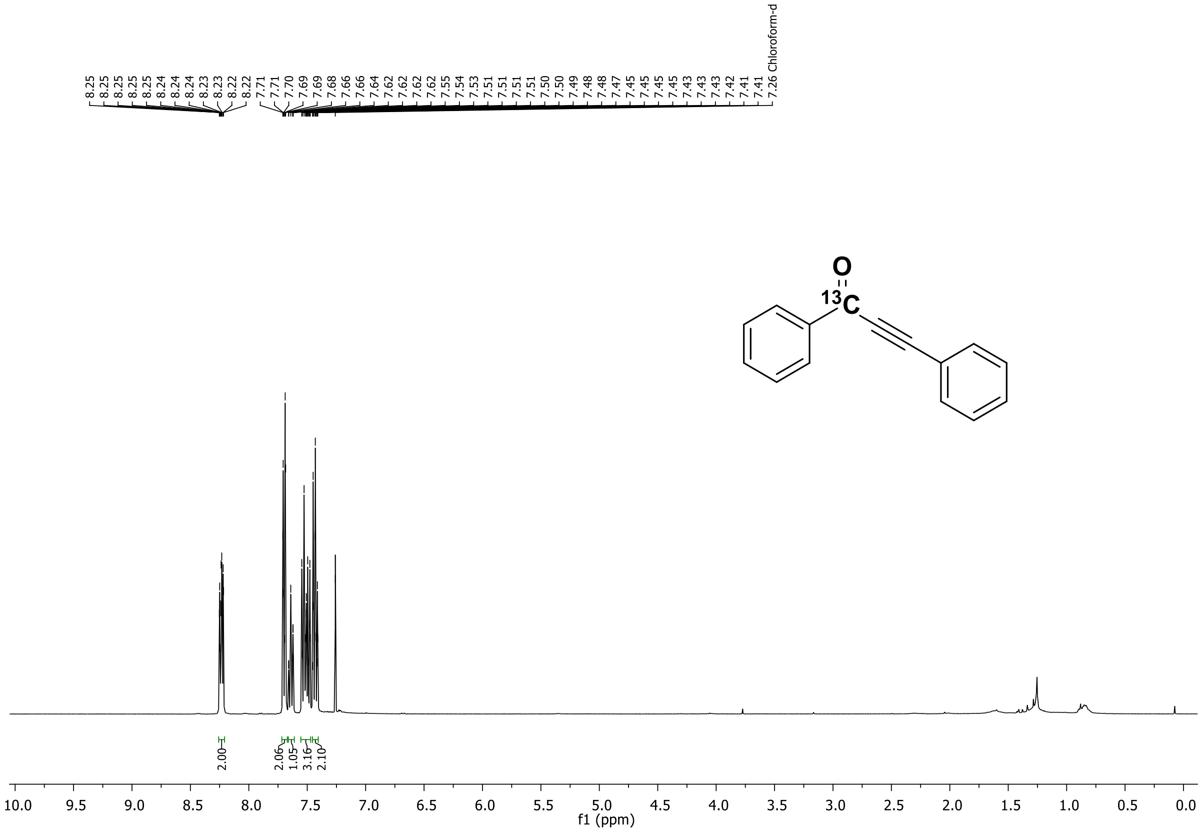

Supplementary Figure 111. ^{1}H NMR Spectrum (400 MHz, $CDCl_3$) of compound **[^{13}C]34**.

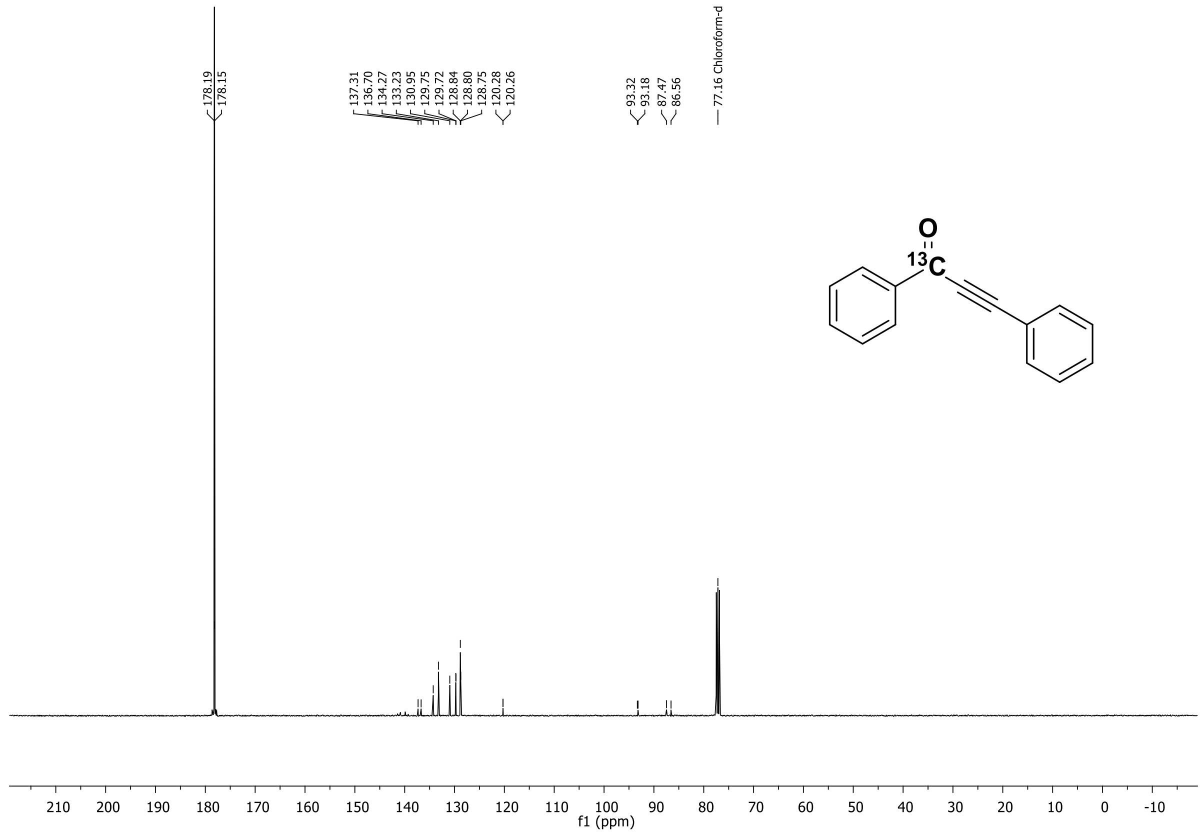

Supplementary Figure 112. ^{13}C NMR Spectrum (100 MHz, $CDCl_3$) of compound **[^{13}C]34**.

[^{13}C]- 1,3,5-triphenyl-1H-pyrazole ([^{13}C]35)

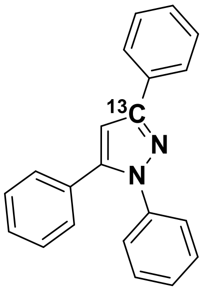

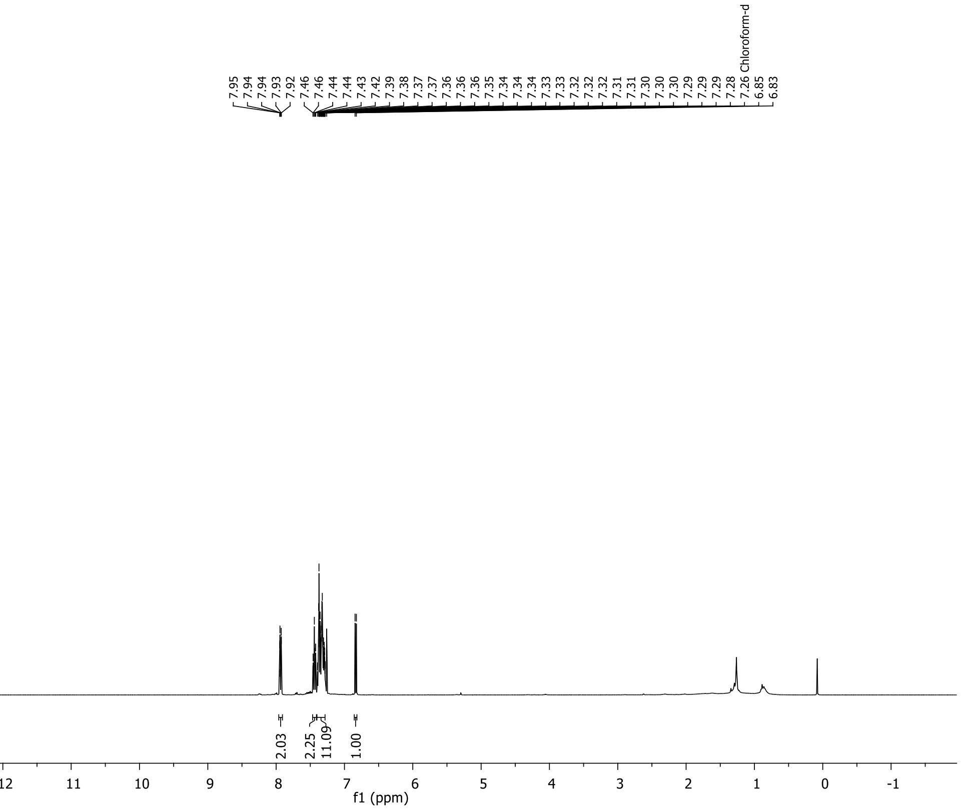

Supplementary Figure 113. ^{1}H NMR Spectrum (400 MHz, $CDCl_3$) of compound **[^{13}C]35**.

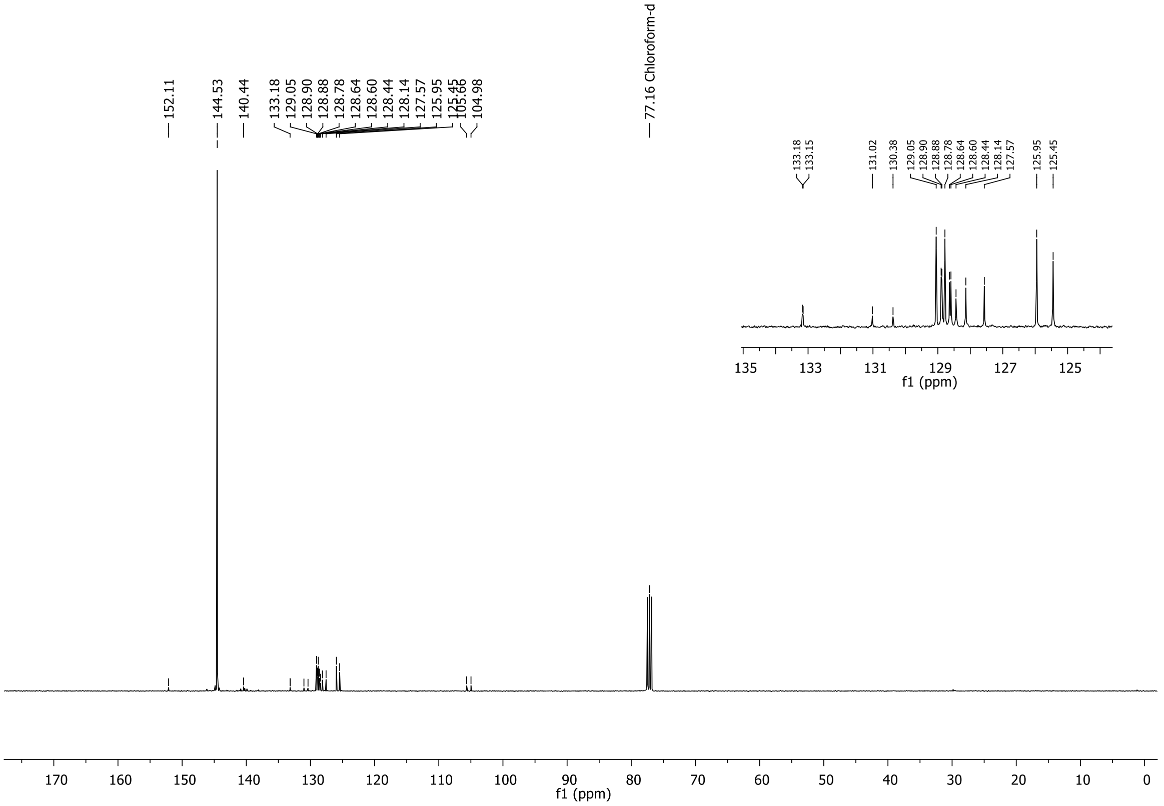

Supplementary Figure 114. ^{13}C NMR Spectrum (100 MHz, $CDCl_3$) of compound **[^{13}C]35**.

[13C]- phenyl(2-phenylbenzofuran-3-yl)methanone ([13C]36)

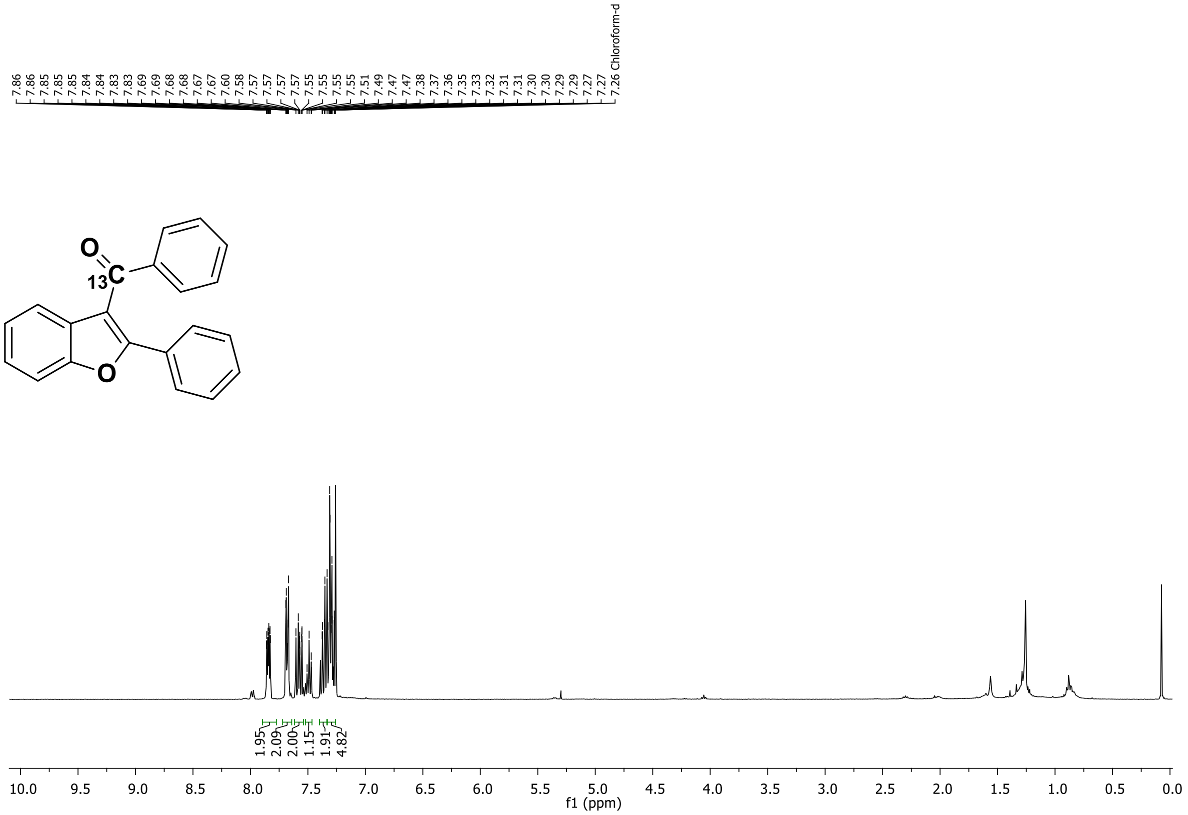

Supplementary Figure 115. ^{1}H NMR Spectrum (400 MHz, $CDCl_3$) of compound **[^{13}C]36**.

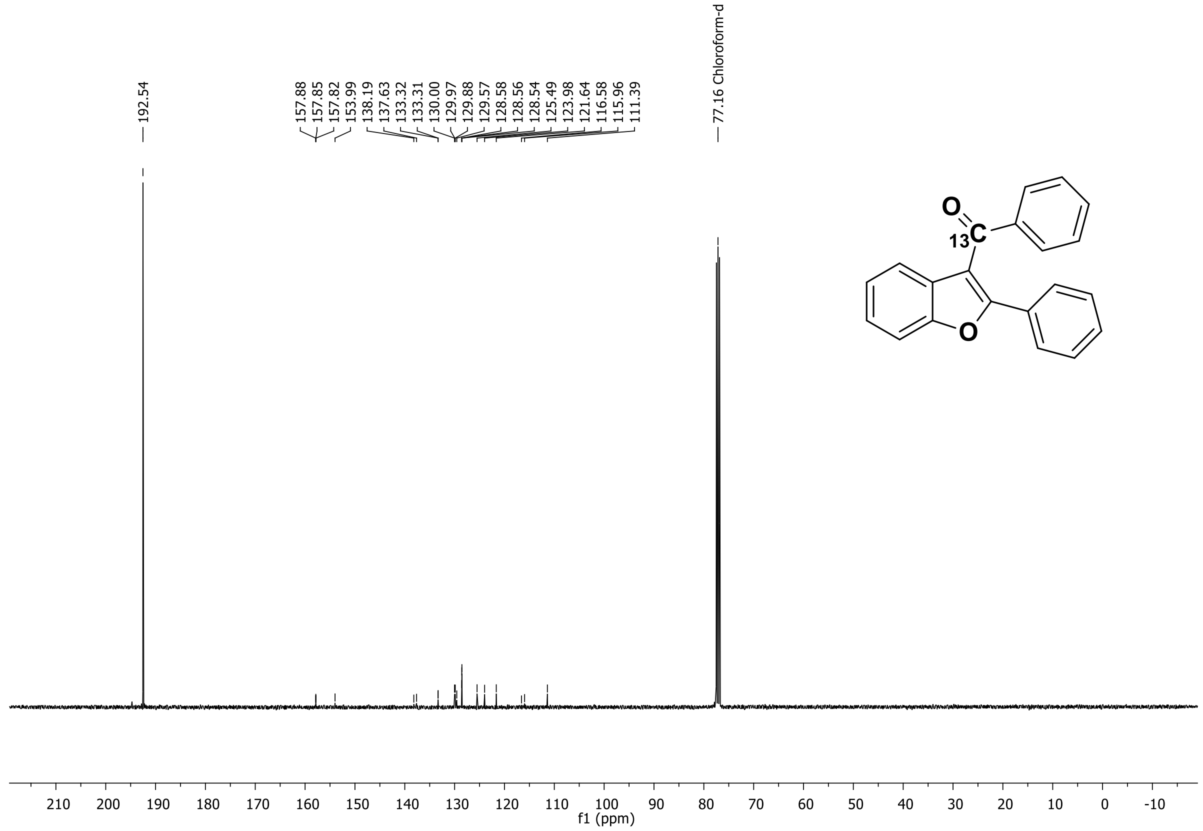

Supplementary Figure 116. ^{13}C NMR Spectrum (100 MHz, $CDCl_3$) of compound **[^{13}C]36**.

[^{13}C]- (3-fluoro-4-methoxyphenyl)(3,4,5-trimethoxyphenyl)methanone ([^{13}C]37)

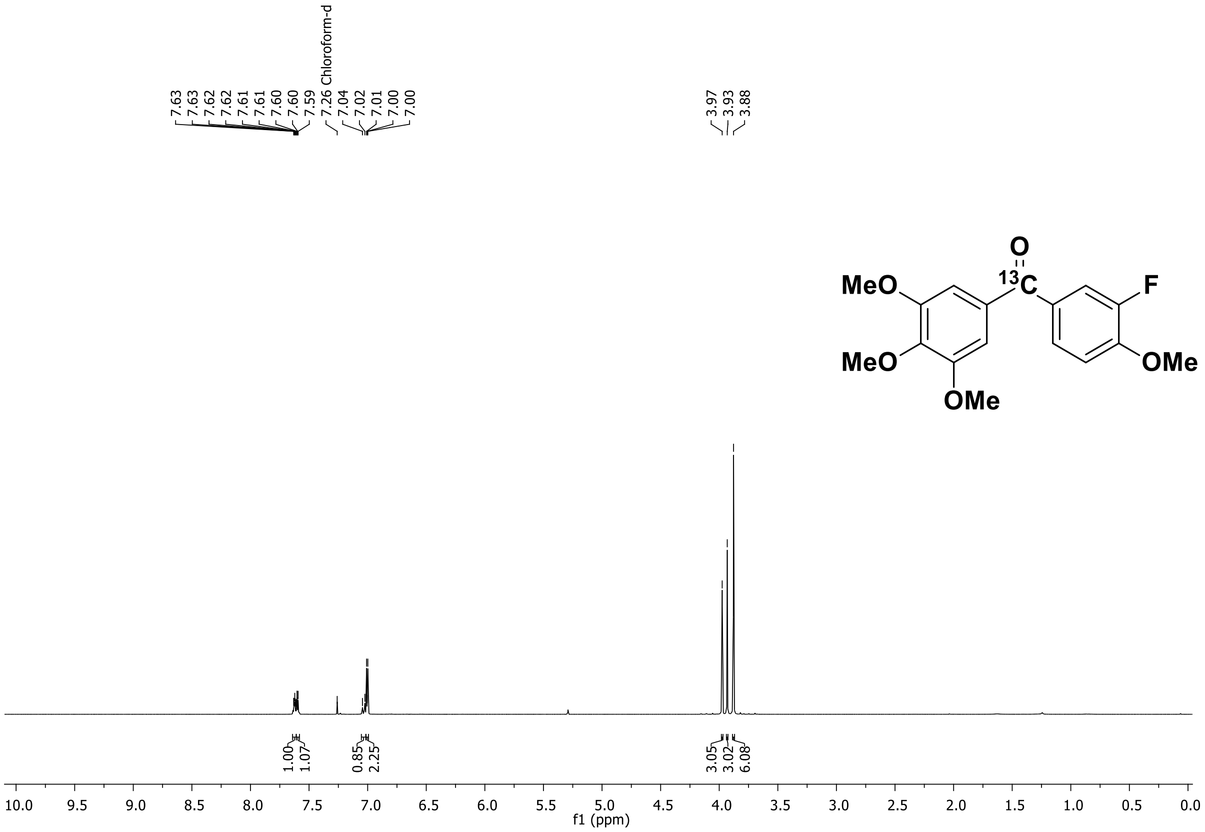

Supplementary Figure 117. ^{1}H NMR Spectrum (400 MHz, $CDCl_3$) of compound **[^{13}C]37**.

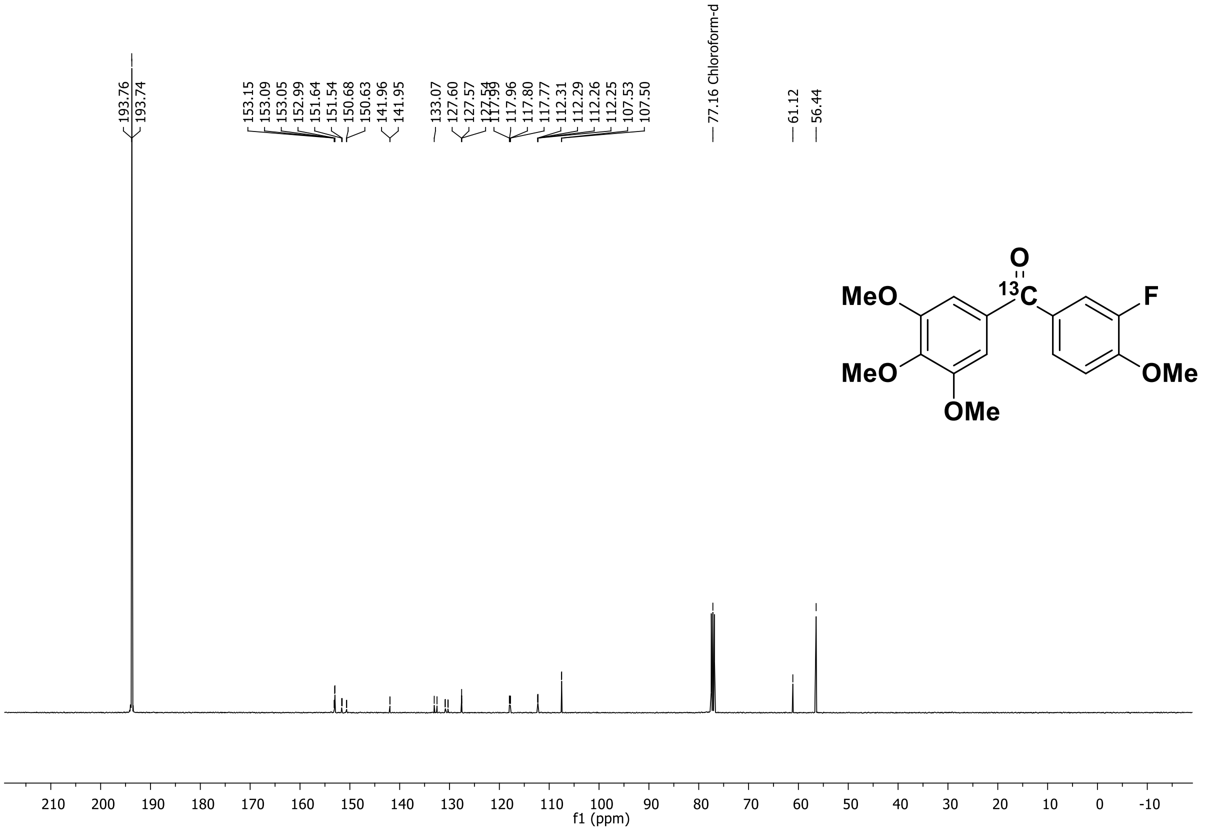

Supplementary Figure 118. ^{13}C NMR Spectrum (100 MHz, $CDCl_3$) of compound **[^{13}C]37**.

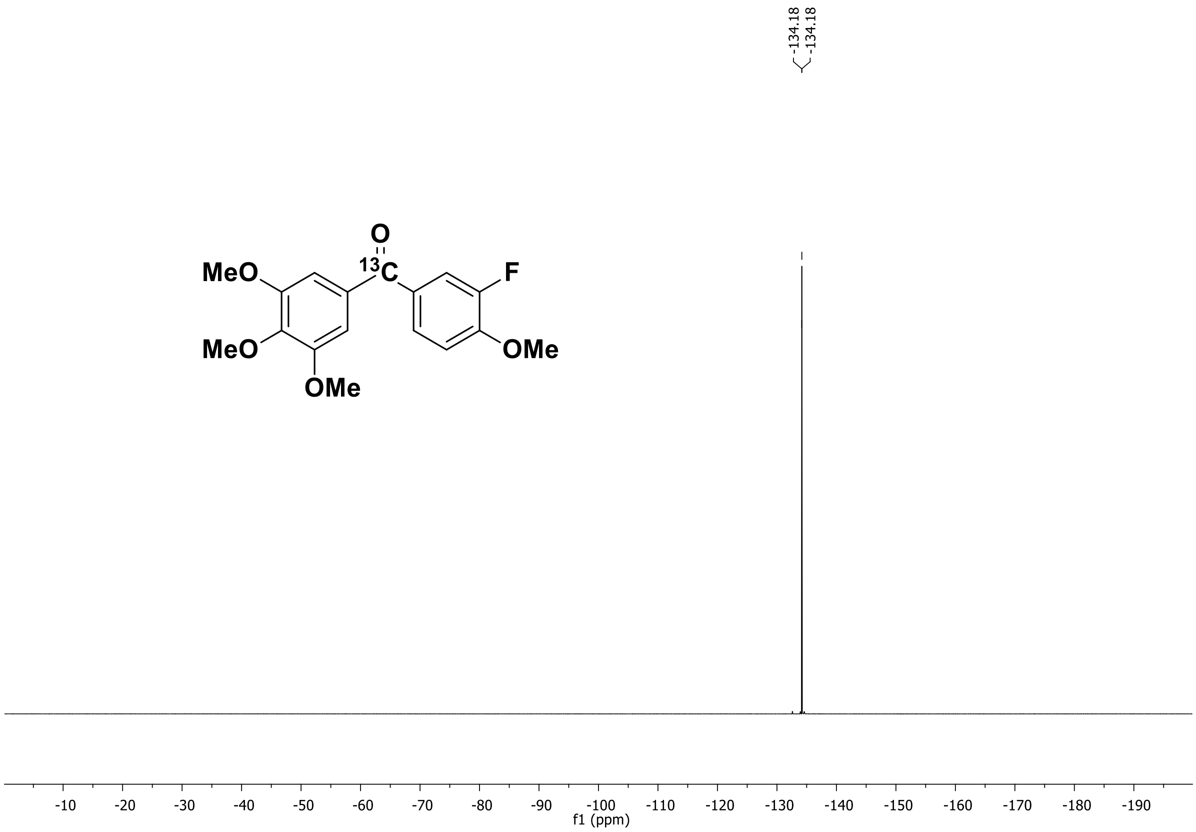

Supplementary Figure 119. ^{19}F NMR Spectrum (376 MHz, $CDCl_3$) of compound **[^{13}C]37**.

[^{13}C]-isoFCA-4 ([^{13}C]38)

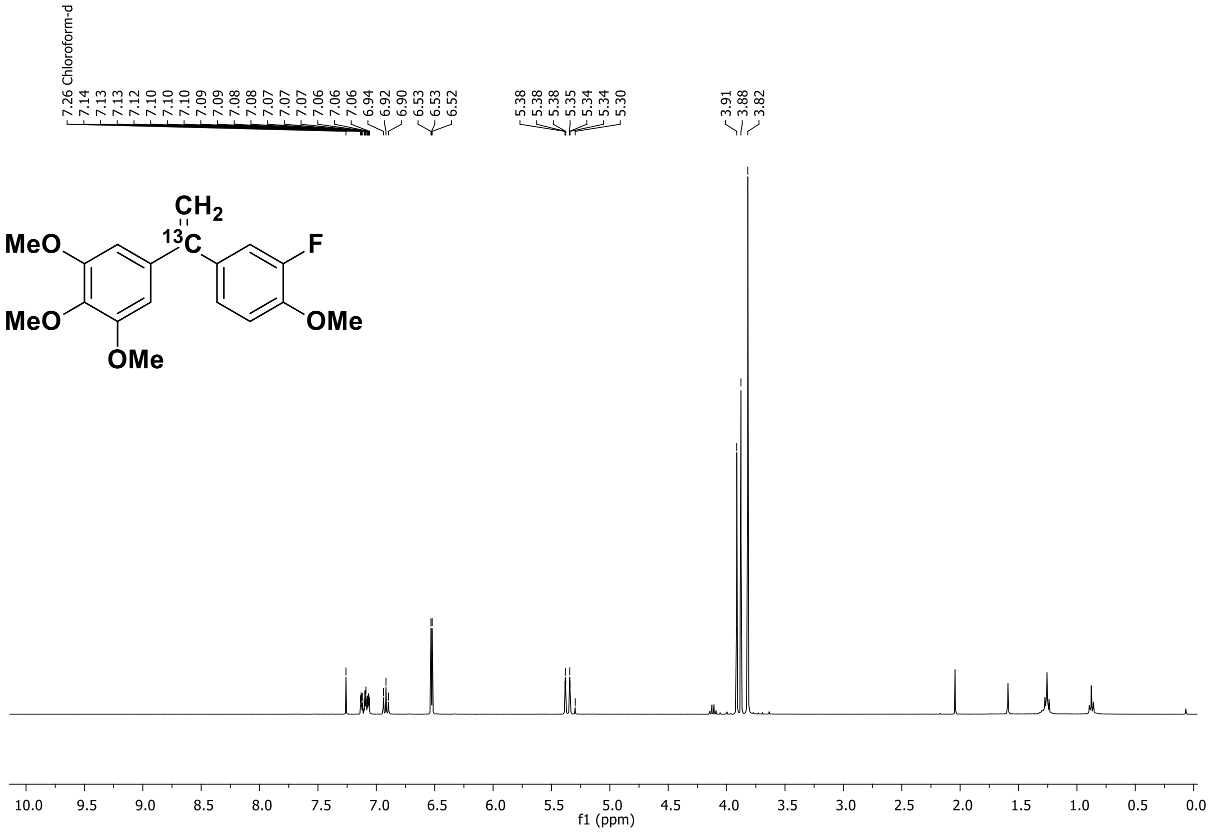

Supplementary Figure 120. ^{1}H NMR Spectrum (400 MHz, $CDCl_3$) of compound **[^{13}C]38**.

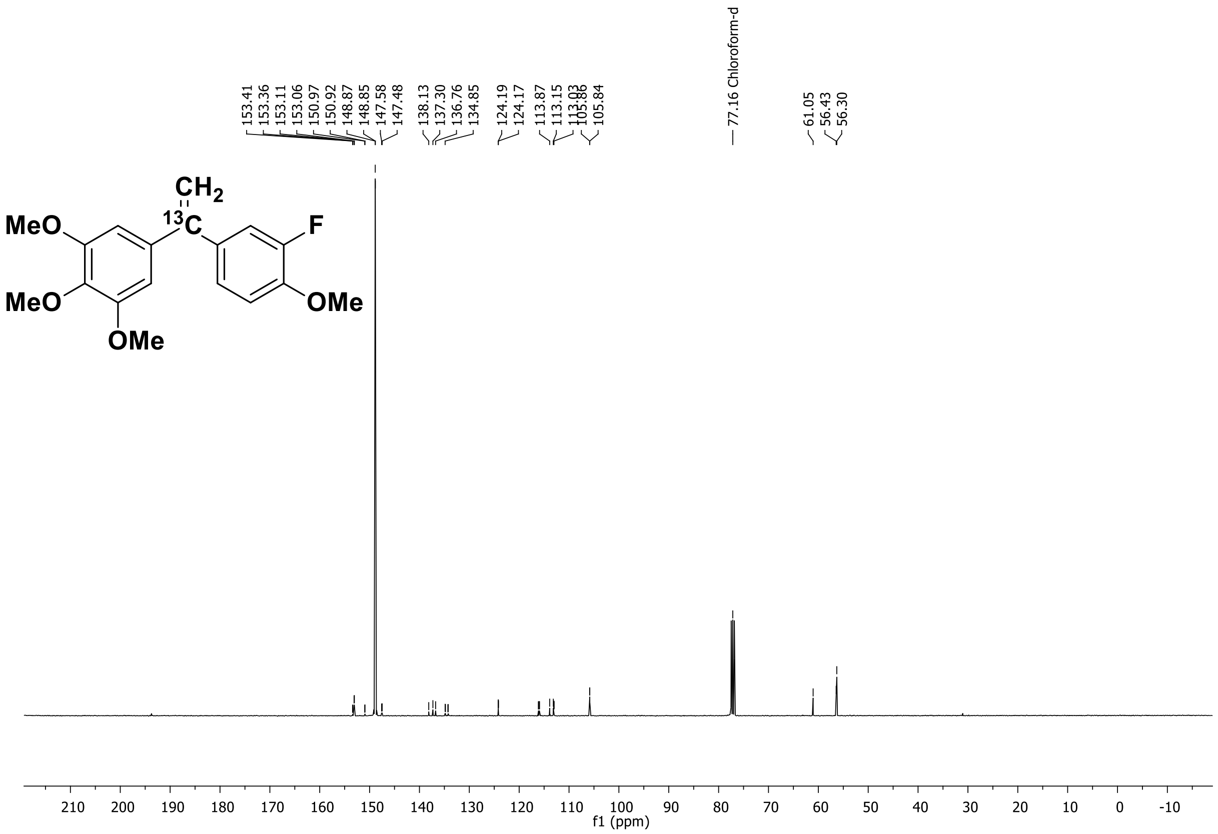

Supplementary Figure 121. ^{13}C NMR Spectrum (100 MHz, $CDCl_3$) of compound **[^{13}C]38**.

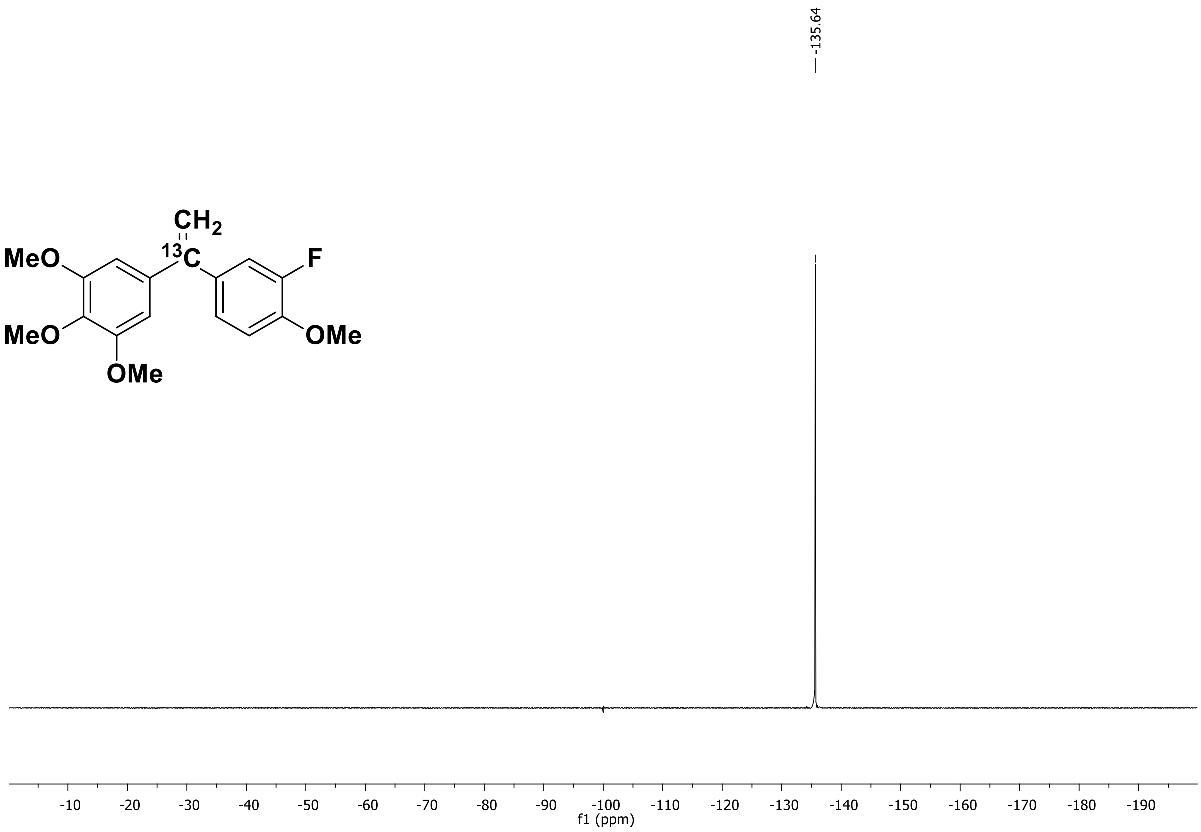

Supplementary Figure 122. ^{19}F NMR Spectrum (376 MHz, $CDCl_3$) of compound **[^{13}C]38**.

[^{13}C] 3,4-diphenylfuran-2(5H)-one ([^{13}C]39)

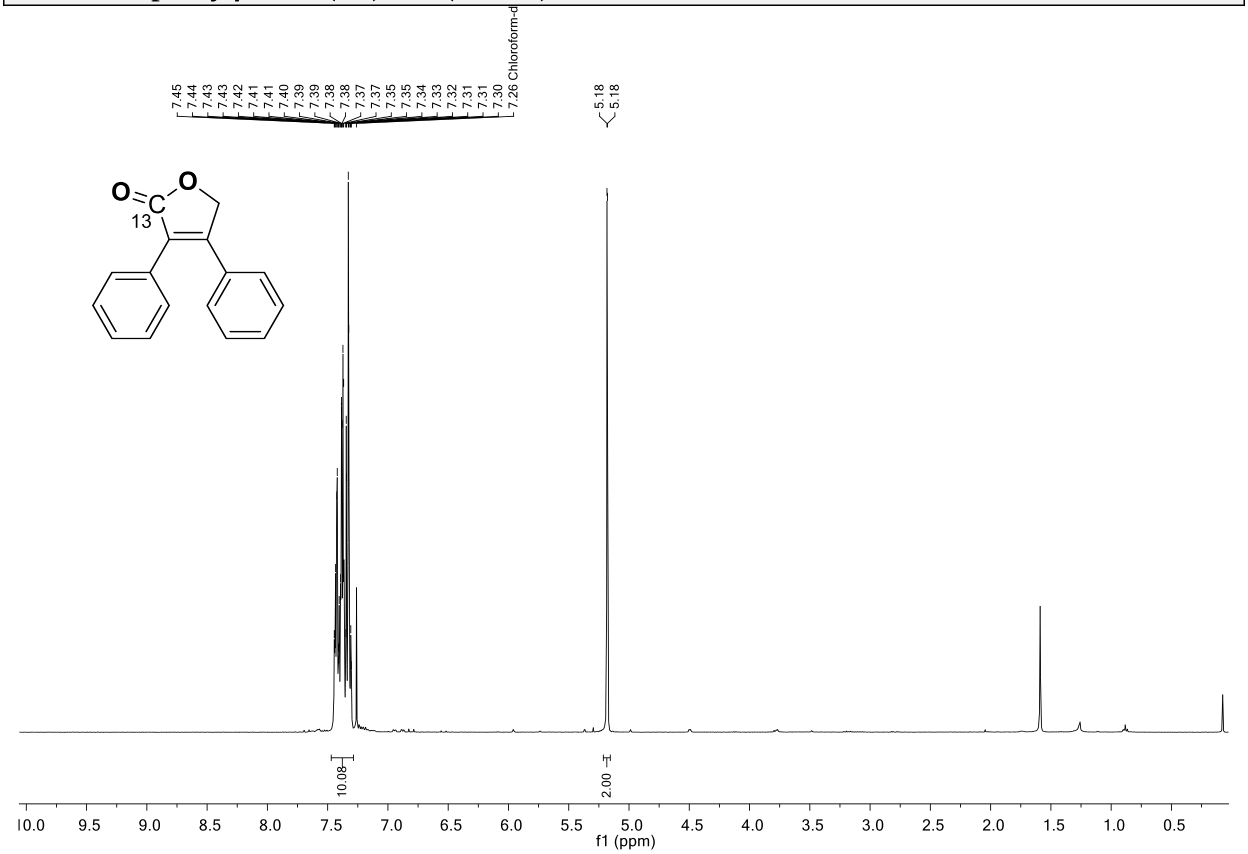

Supplementary Figure 123. ^{1}H NMR Spectrum (400 MHz, $CDCl_3$) of compound **[^{13}C]39**.

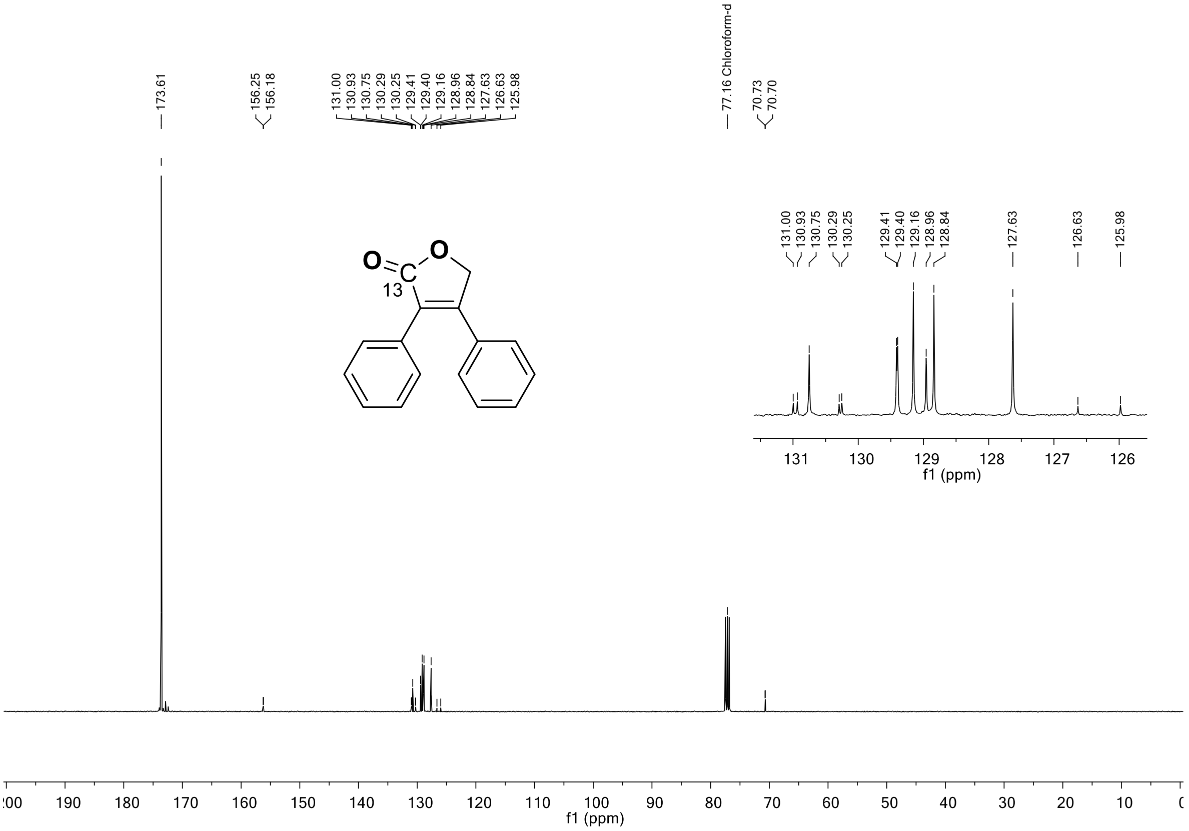

Supplementary Figure 124. ^{13}C NMR Spectrum (100 MHz, $CDCl_3$) of compound **[^{13}C]39**.

[^13C] 2-(diethylamino)ethyl 4-butoxybenzoate ([^13C]40)

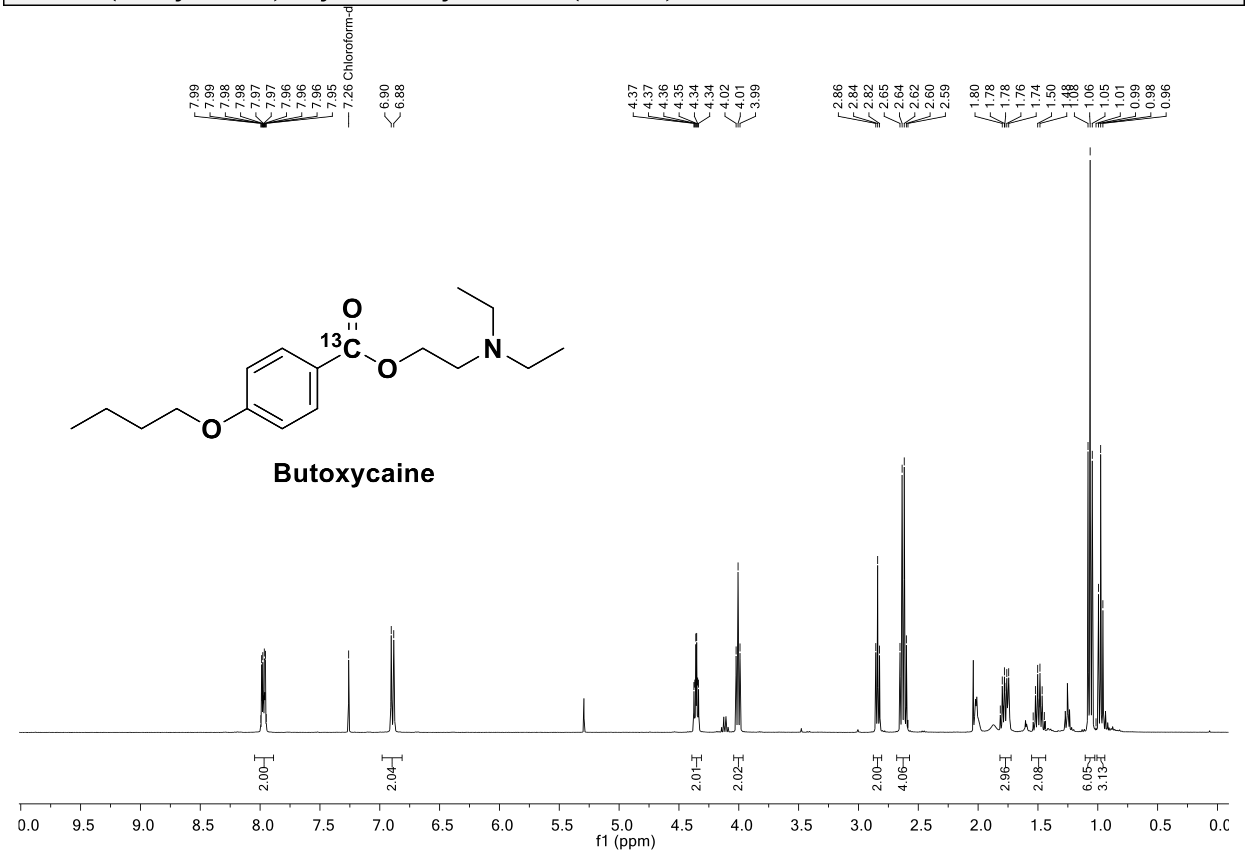

Supplementary Figure 125. ^{1}H NMR Spectrum (400 MHz, $CDCl_3$) of compound **[^{13}C]40**.

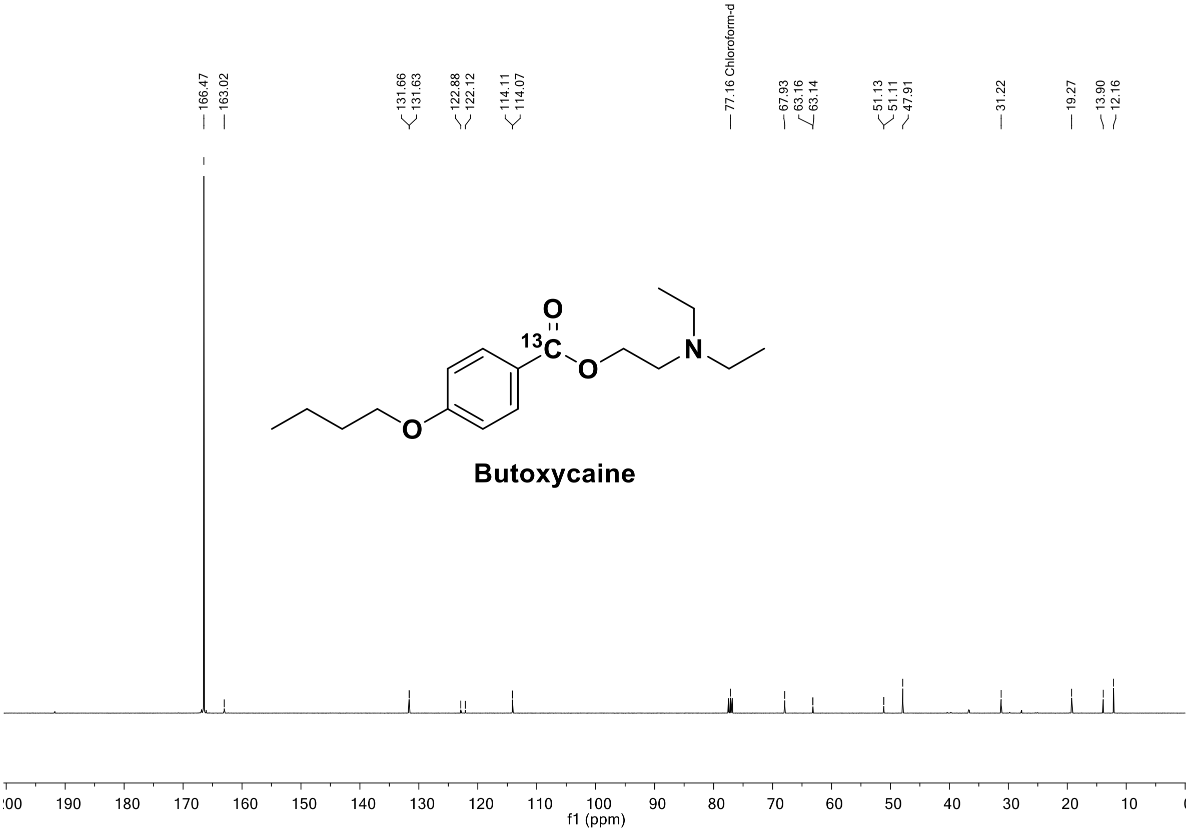

Supplementary Figure 126. ^{13}C NMR Spectrum (100 MHz, $CDCl_3$) of compound **[^{13}C]40**.

[13C] 4-chloro-N-(2-morpholinoethyl)benzamide ([13C]41)

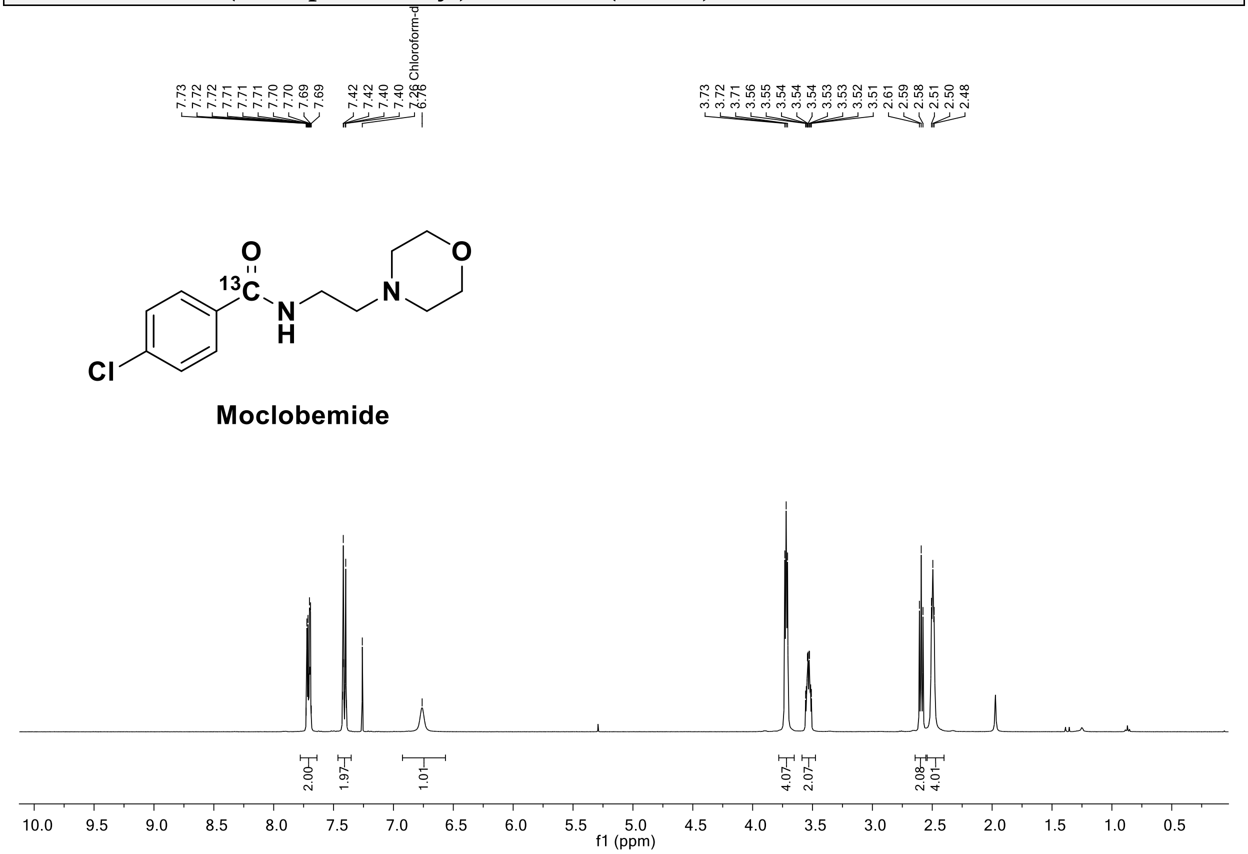

Supplementary Figure 127. ^{1}H NMR Spectrum (400 MHz, $CDCl_3$) of compound **[13C]41**.

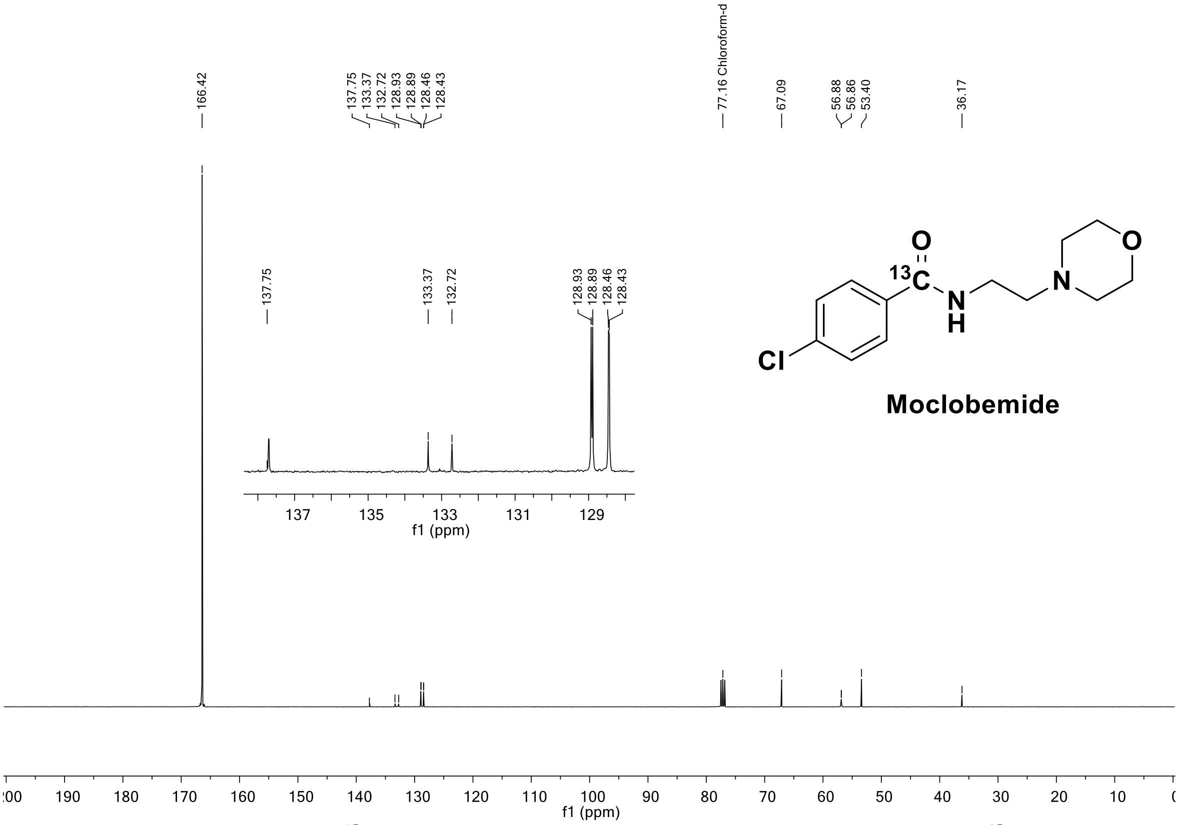

Supplementary Figure 128. ^{13}C NMR Spectrum (100 MHz, $CDCl_3$) of compound **[13C]41**.

[^{13}C]-Probenecid ([^{13}C]42)

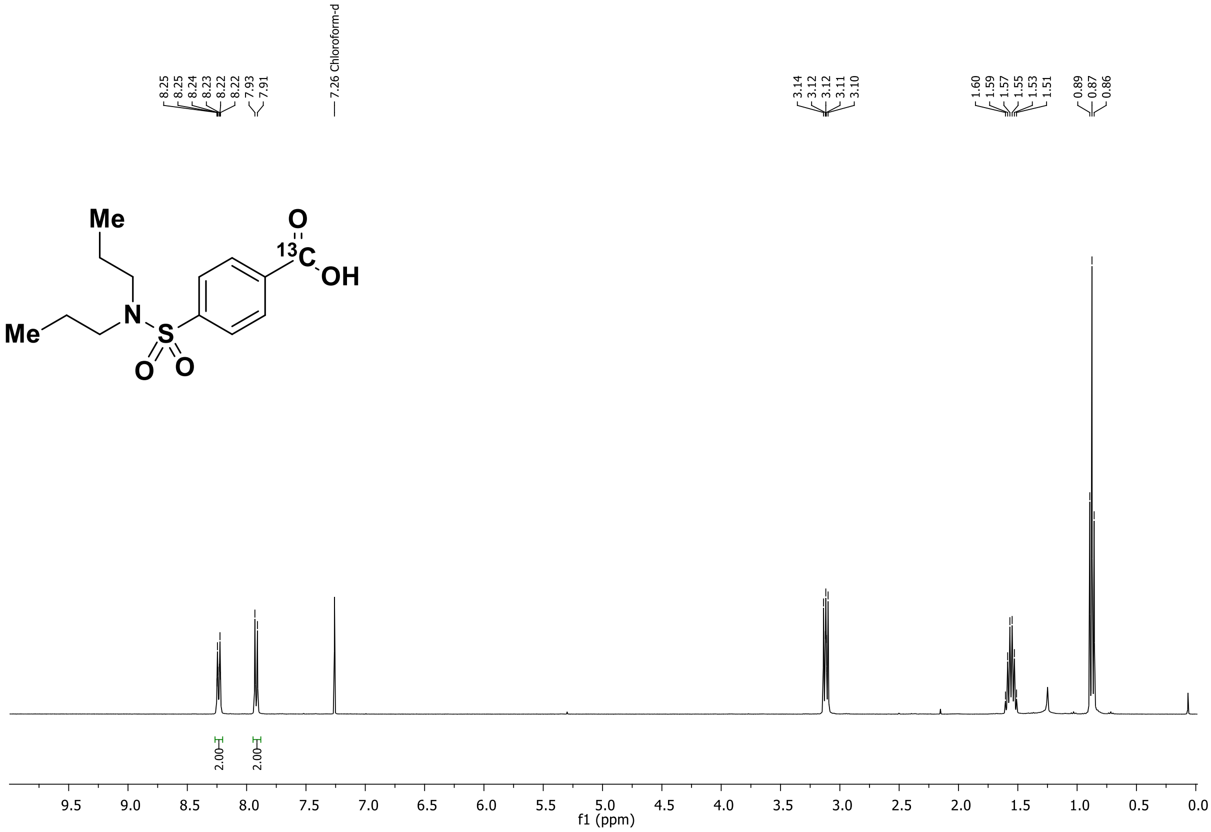

Supplementary Figure 129. ^{1}H NMR Spectrum (400 MHz, $CDCl_3$) of compound **[^{13}C]42**.

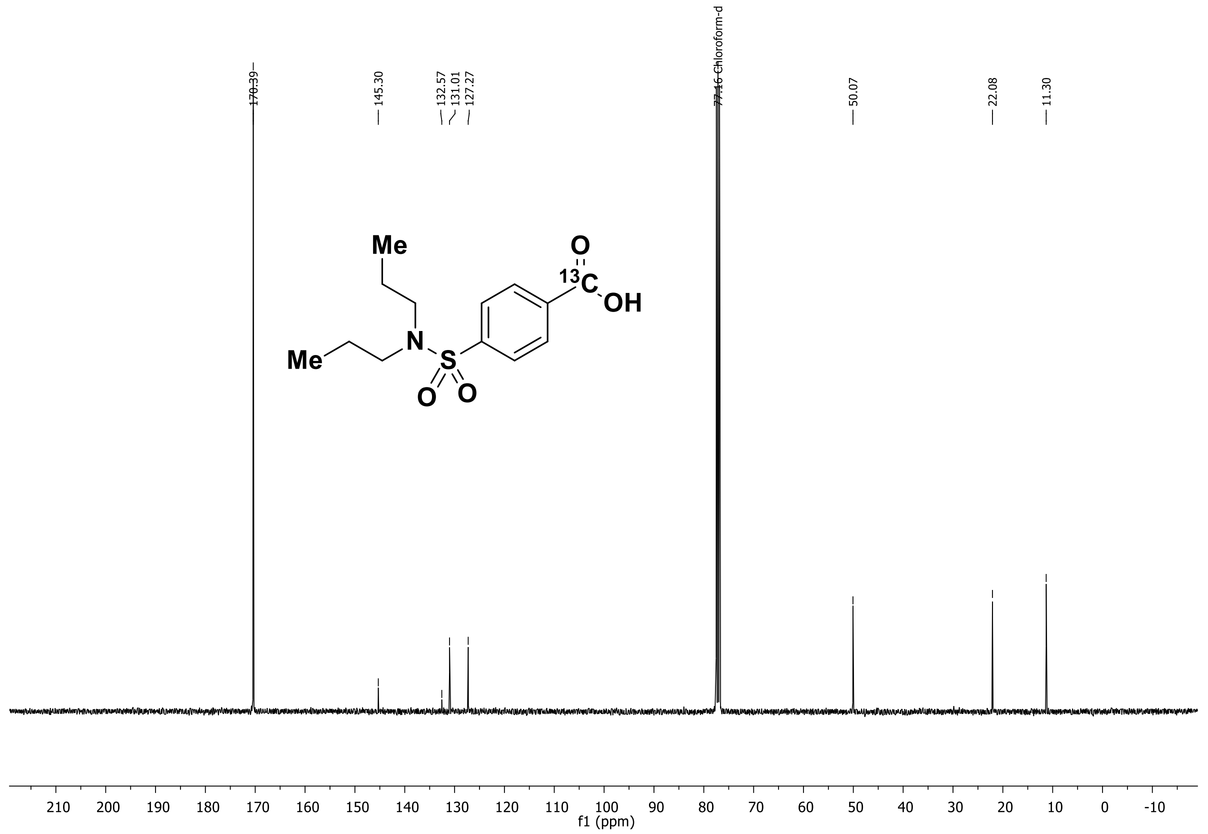

Supplementary Figure 130. ^{13}C NMR Spectrum (100 MHz, $CDCl_3$) of compound **[^{13}C]42**.

IV. Supplementary references

(1) Rountree, E. S.; McCarthy, B. D.; Eisenhart, T. T.; Dempsey, J. L. *Inorg. Chem.* **2014**, *53*, 9983–10002.
(2) Costentin, C.; Savéant, J.-M. *ChemElectroChem* **2014**, *1*, 1226–1236.
(3) Clark, M. L.; Cheung, P. L.; Lessio, M.; Carter, E. A.; Kubiak, C. P. *ACS Catal.* **2018**, *8*, 2021–2029.
(4) Johnson, K. A.; Simpson, Z. B.; Blom, T. *Anal. Biochem.* **2009**, *387*, 20–29.
(5) Johnson, K. A.; Simpson, Z. B.; Blom, T. *Anal. Biochem.* **2009**, *387*, 30–41.
(6) Gotico, P.; Del Vecchio, A.; Audisio, D.; Quaranta, A.; Halime, Z.; Leibl, W.; Aukauloo, A. *ChemPhotoChem* **2018**, *2*, 715–719.
(7) Tamaki, Y.; Koike, K.; Morimoto, T.; Ishitani, O. *J. Catal.* **2013**, *304*, 22–28.
(8) Keith, J. A.; Grice, K. A.; Kubiak, C. P.; Carter, E. A. *J. Am. Chem. Soc.* **2013**, *135*, 15823–15829.
(9) Riplinger, C.; Sampson, M. D.; Ritzmann, A. M.; Kubiak, C. P.; Carter, E. A. *J. Am. Chem. Soc.* **2014**, *136*, 16285–16298.
(10) Bhugun, Iqbal.; Lexa, Doris.; Saveant, J.-Michel. *Anal. Chem.* **1994**, *66*, 3994–3996.
(11) Jödecke, M.; Pérez-Salado Kamps, Á.; Maurer, G. *J. Chem. Eng. Data* **2012**, *57*, 1249–1266.
(12) Khan, M. M. T.; Halligudi, S. B.; Shukla, S. *J. Chem. Eng. Data* **1989**, *34*, 353–355.
(13) Hasegawa, E.; Takizawa, S.; Seida, T.; Yamaguchi, A.; Yamaguchi, N.; Chiba, N.; Takahashi, T.; Ikeda, H.; Akiyama, K. *Tetrahedron* **2006**, *62*, 6581–6588.
(14) Ching, H. Y. V.; Wang, X.; He, M.; Perujo Holland, N.; Guillot, R.; Slim, C.; Griveau, S.; Bertrand, H. C.; Policar, C.; Bedioui, F.; Fontecave, M. *Inorg. Chem.* **2017**, *56*, 2966–2976.
(15) Hermange, P.; Lindhardt, A. T.; Taaning, R. H.; Bjerglund, K.; Lupp, D.; Skrydstrup, T. *J. Am. Chem. Soc.* **2011**, *133*, 6061–6071.
(16) He, X.; Cao, Y.; Lang, X.-D.; Wang, N.; He, L.-N. *ChemSusChem* **2018**, *11*, 3382–3387.
(17) Korsager, S.; Taaning, R. H.; Lindhardt, A. T.; Skrydstrup, T. *J. Org. Chem.* **2013**, *78*, 6112–6120.
(18) Neumann, K. T.; Laursen, S. R.; Lindhardt, A. T.; Bang-Andersen, B.; Skrydstrup, T. *Org. Lett.* **2014**, *16*, 2216–2219.
(19) Gauthier, D. R. Jr.; Rivera, N. R.; Yang, H.; Schultz, D. M.; Shultz, C. S. *J. Am. Chem. Soc.* **2018**, *140*, 15596–15600.
(20) Lang, X.-D.; He, L.-N. *ChemSusChem* **2018**, *11*, 2062–2067.
(21) Gill, G. S.; Grobelny, D. W.; Chaplin, J. H.; Flynn, B. L. *J. Org. Chem.* **2008**, *73*, 1131–1134.
(22) Cornilleau, T.; Audrain, H.; Guillemet, A.; Hermange, P.; Fouquet, E. *Org. Lett.* **2015**, *17*, 354–357.
(23) Chauhan, D. P.; Varma, S. J.; Gudem, M.; Panigrahi, N.; Singh, K.; Hazra, A.; Talukdar, P. *Org. Biomol. Chem.* **2017**, *15*, 4822–4830.
